# Palladium(ii)-catalyzed synthesis of dibenzothiophene derivatives *via* the cleavage of carbon–sulfur and carbon–hydrogen bonds†
†Electronic supplementary information (ESI) available: Experimental procedures and characterization data for all new compounds. See DOI: 10.1039/c5sc04890g


**DOI:** 10.1039/c5sc04890g

**Published:** 2016-01-21

**Authors:** Mamoru Tobisu, Yoshihiro Masuya, Katsuaki Baba, Naoto Chatani

**Affiliations:** a Department of Applied Chemistry , Faculty of Engineering , Osaka University , Suita , Osaka 565-0871 , Japan . Email: tobisu@chem.eng.osaka-u.ac.jp ; Email: chatani@chem.eng.osaka-u.ac.jp; b Center for Atomic and Molecular Technologies , Graduate School of Engineering , Osaka University , Suita , Osaka 565-0871 , Japan

## Abstract

A new process has been developed for the palladium(ii)-catalyzed synthesis of dibenzothiophene derivatives *via* the cleavage of C–H and C–S bonds. In contrast to the existing methods for the synthesis of this scaffold by C–H functionalization, this new catalytic C–H/C–S coupling method does not require the presence of an external stoichiometric oxidant or reactive functionalities such as C–X or S–H, allowing its application to the synthesis of elaborate π-systems. Notably, the product-forming step of this reaction lies in an oxidative addition step rather than a reductive elimination step, making this reaction mechanistically uncommon.

## Abstract 2

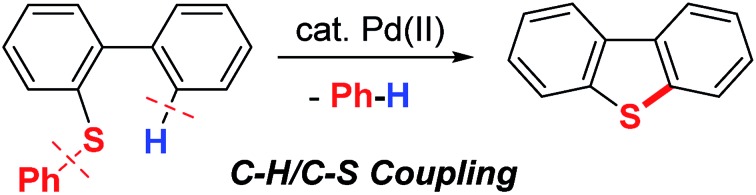
Pd(ii)-catalyzed synthesis of dibenzothiophene derivatives has been developed. The reaction proceeds through the cleavage of carbon–hydrogen and carbon–sulfur bonds.

## Introduction

**Scheme 1:**
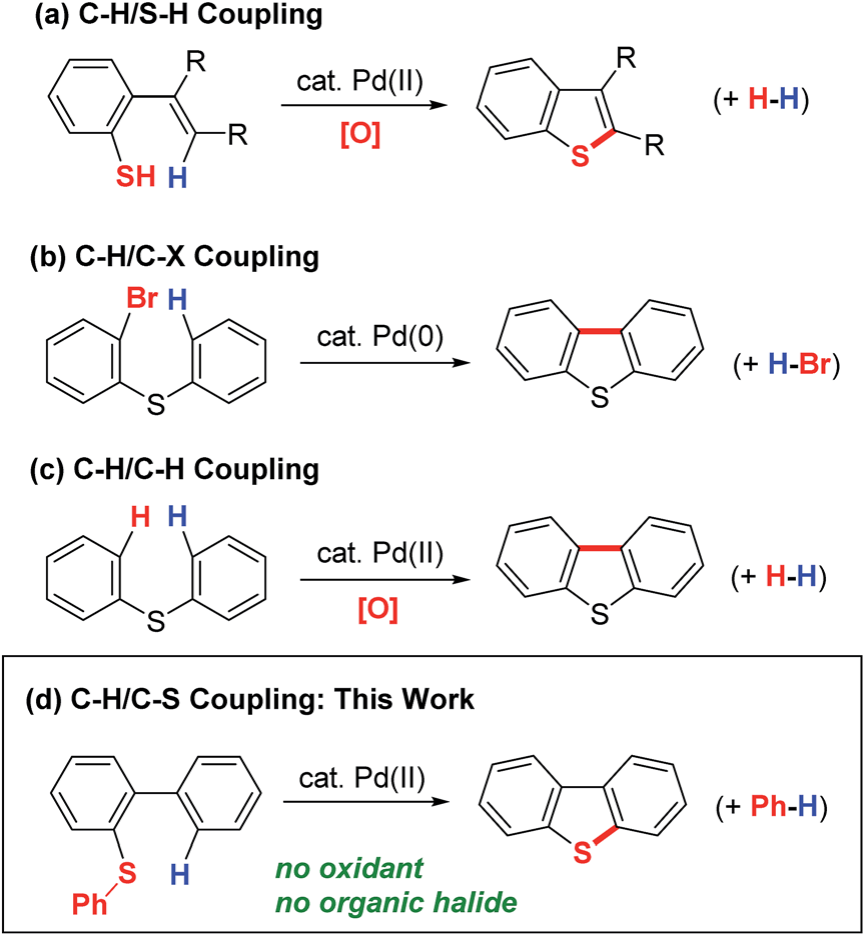
Catalytic synthetic approaches to benzo-fused thiophenes *via* C–H activation.

Thiophenes and their benzo-fused derivatives constitute a privileged class of scaffolds with numerous applications, in pharmaceuticals^1^ and advanced molecular materials.^2^ Although a wide range of methods are currently available for the synthesis of thiophene derivatives,^3^ recent research efforts have focused on the development of catalytic C–H functionalization reactions^4^ in the hope of achieving increasingly facile processes for the construction of elaborate thiophene derivatives. In this context, three different classes of catalytic reaction have been reported to date for the synthesis of thiophene derivatives. The first class involves a C–S bond-forming ring closure, which occurs *via* an oxidative C–H/S–H coupling reaction (Scheme 1a).^5^,^6^ However, the inherent instability and toxicity of thiophenol-based substrates have limited the practical application of this method. The second method is based on an intramolecular C–H/C–X coupling reaction under Pd(0)/Pd(ii) catalysis (Scheme 1b).^7^ However, the starting halogenated biaryl sulfides (or sulfoxides) required for this strategy can only be synthesized by S_N_Ar,7*a* Grignard,7*a* RLi7*b* or S_E_Ar-type C–H palladation7*c* reactions, which has limited the structural diversity of the thiophene derivatives that can be accessed by this protocol. The third of these three different methods for the synthesis of dibenzothiophene derivatives involves the intramolecular oxidative C–H/C–H coupling of simple diaryl sulfides (Scheme 1c).^6^,^8^ This approach is particularly interesting in the sense that it allows for the direct conversion of less functionalized substrates into the desired products. Despite this advantage, the application of this process has been limited by its requirement for the use of a large excess of a silver oxidant (2–4 equiv.). Herein, we report a unique C–H/C–S coupling strategy for the catalytic synthesis of fused thiophene derivatives (Scheme 1d). The notable features of the reaction are as follows: (1) it does not require any reactive functionalities, such as C–X or S–H bonds; (2) it does not require an external oxidant, such as a silver salt; and (3) the reaction proceeds through the cleavage of inert C–H and C–S bonds.^9^ Although strong acid-mediated reaction of biphenyl sulfoxides was reported to form dibenzothiophenes through formal C–H and S–Me cleavage, its mechanism involves classical Friedel–Crafts type C–H functionalization and S–Me cleavage *via* the S_N_2 mechanism.^10^

## Results and discussion

We initially selected biphenyl sulfide **1** as a test substrate to develop a catalytic synthesis of dibenzothiophene **2***via* the activation of its C–H and C–S bonds. It was envisioned that the required C–H activation of the 2′-position in **1** could be achieved by a sulfur-directed cyclometallation process.^11^,^12^ With this in mind, we focused our initial effort on the development of suitable conditions for this unprecedented cyclization process involving C–S activation.^9^ After several experiments, we found that Pd(OAc)_2_ performed as a potential catalyst for the cyclization of **1a** (Table 1, entry 1). Changing the leaving group on the sulfur atom from Me (**1a**) to Ph (**1b**) led to an increase in the yield of **3** from 5 to 15% (entry 2). Further improvements in yield were accomplished by adding a carboxylic acid ligand,^13^ with 2,6-Me_2_C_6_H_3_CO_2_H (**3**) being optimal (entry 5).

**Table 1:** Effect of ligands^*a*^

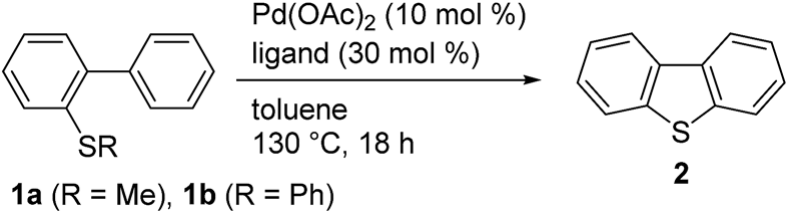			
Entry	Substrate	Ligand	NMR yield of **2** [%]
1	**1a**	None	5
2	**1b**	None	15
3	**1b**	PivOH	57
4	**1b**	2,6-Me_2_C_6_H_3_CO_2_H (**3**)	66
5^*b*^	**1b**	**3**	87 (79)^*c*^
6^*b*^	**1a**	**3**	7

^*a*^Reaction conditions: **1** (0.30 mmol), Pd(OAc)_2_ (0.030 mmol), and ligand (0.090 mmol) in toluene (1.0 mL) at 130 °C for 18 h.

^*b*^Pd(OAc)_2_ (0.045 mmol), and **3** (0.135 mmol) were used.

^*c*^Isolated yield.

**Fig. 1:**
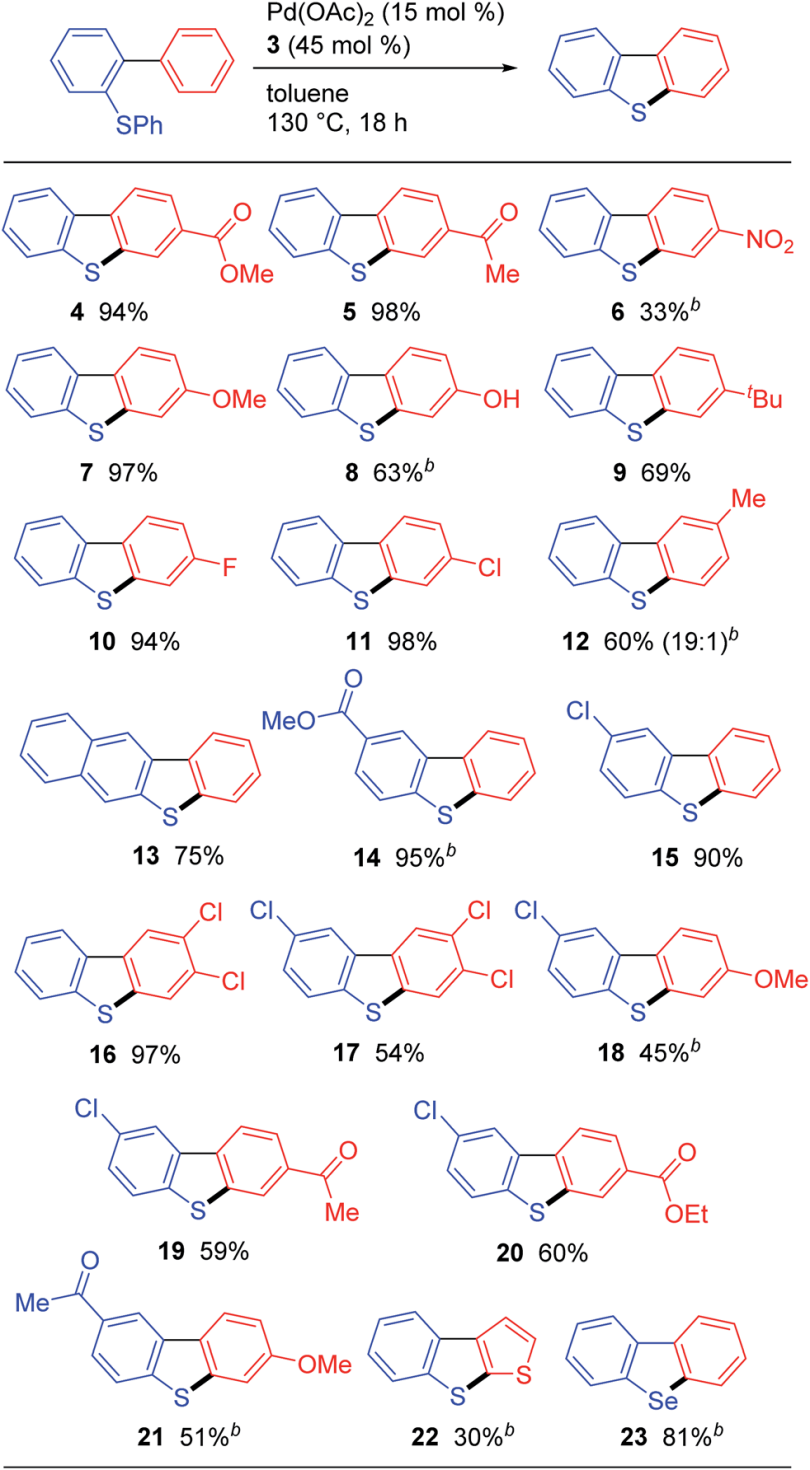
Pd-catalyzed synthesis of dibenzothiophenes *via* the cleavage of C–H and C–S bonds^*a*^. ^*a*^Reaction conditions: **1** (0.30 mmol), Pd(OAc)_2_ (0.045 mmol), and **3** (0.135 mmol) in toluene (1.0 mL) at 130 °C for 18 h. Isolated yields are shown. ^*b*^Pd(OAc)_2_ (0.090 mmol), and **3** (0.27 mmol) were used.

With the optimized conditions in hand, we proceeded to evaluate the scope of this palladium-catalyzed C–H/C–S coupling reaction (Fig. 1). Pleasingly, these conditions allowed for the successful activation of the C–H bonds in both electron-deficient (*i.e.*, **4**, **5** and **6**) and electron-rich (*i.e.*, **7**, **8** and **9**) aromatic rings to give the corresponding C–H/C–S coupling products. Notably, a phenolic OH, which would be incompatible with strong oxidants, was well-tolerated under these conditions, therefore highlighting one of the main advantages of our newly developed protocol over the existing oxidative methods (Scheme 1). Dibenzothiophenes bearing halogen atoms such as F and Cl can also be synthesized (compounds **10**, **11**, and **15**). When the hydrogen atoms at the 2′- and 6′-positions of the substrate were non-equivalent, the cyclization proceeded at the least hindered C–H bond, as evidenced by the regioselective formation of **12**. Unsymmetrical polysubstituted dibenzothiophenes are readily accessed by this method (compounds **16–20**). This method can also be used for the synthesis of the benzo[*b*]thieno[3,2-*d*]thiophene ring systems **22**, albeit in a lower yield than the corresponding dibenzothiophene derivatives likely because of the large angle strain associated with this target. Furthermore, this method provided facile access to dibenzoselenophene (**23**),^14^ thereby demonstrating that these palladium-catalyzed conditions can be used to activate a C–Se bond.^15^

**Scheme 2:**
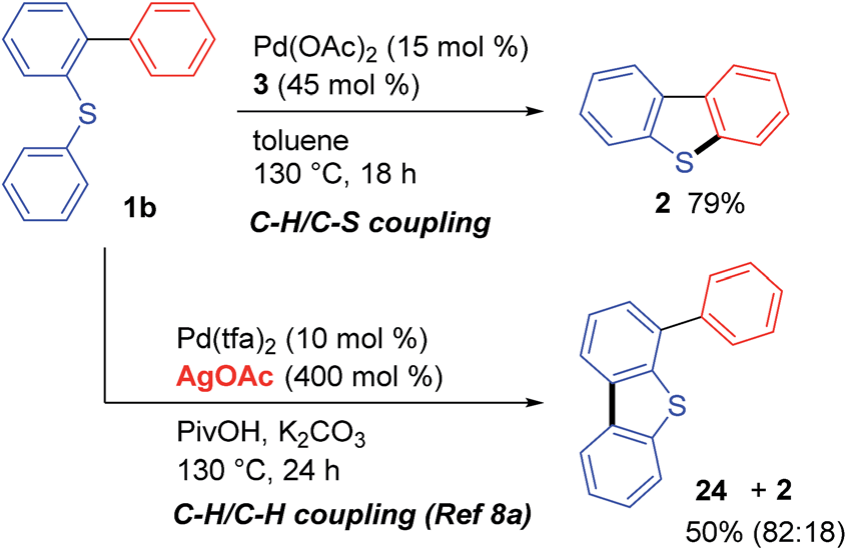
The effect of an added oxidant.

Interestingly, the course of the cyclization reaction of **1b** could be regulated by the addition of a silver oxidant. As detailed above, dibenzothiophene (**2**) was obtained as the sole product when the palladium-catalyzed reaction of **1b** was conducted in the absence of an external oxidant (Scheme 2, C–H/C–S coupling). In contrast, the addition of a silver salt (4.0 equiv. to **1b**) led to the formation of **24** as the major product *via* a C–H/C–H coupling reaction, which was consistent with the results reported by Zhou.8*a* Although both of these reactions are likely to be initiated by a Pd(ii)-mediated sulfur-directed C–H activation process,^11^,^12^ the position of the bond formation is clearly dependent on whether or not an oxidant is present in the reaction mixture.

**Scheme 3:**
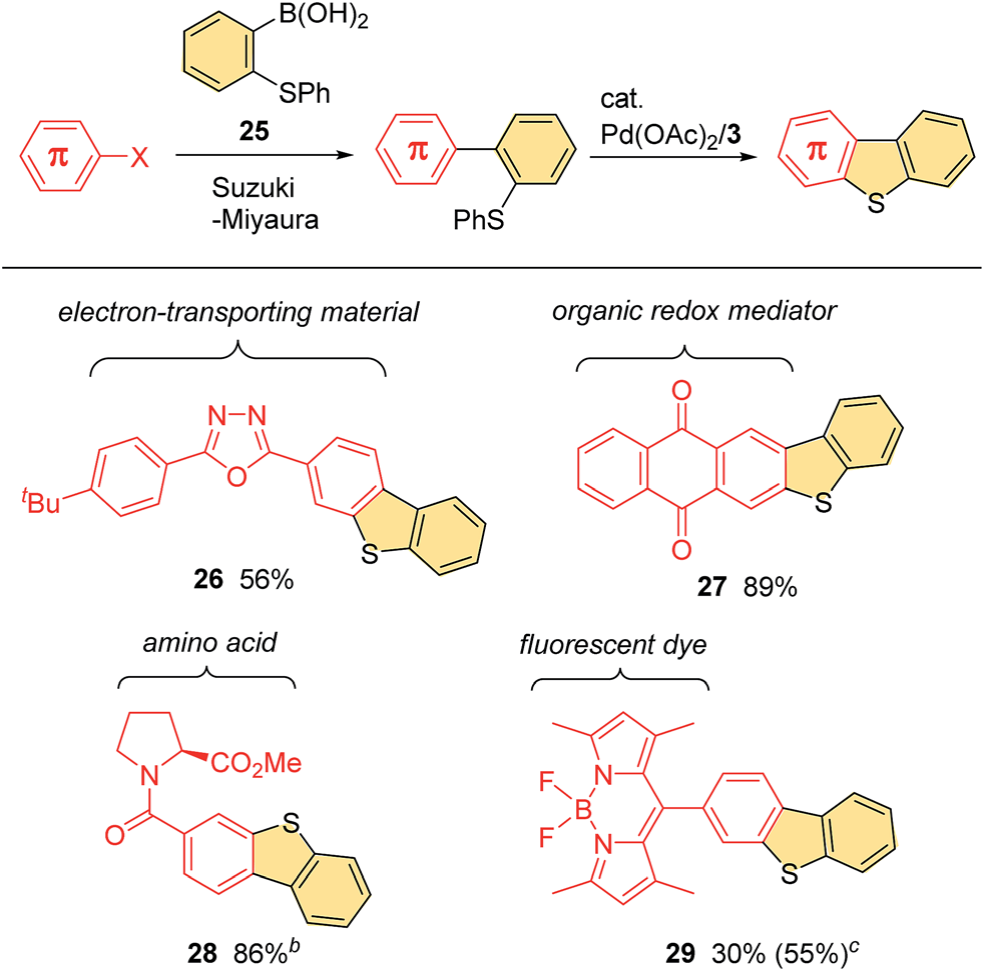
Boronic acid **25** as a versatile reagent for the incorporation of a fused benzothiophene ring to various π-systems^*a*^. ^*a*^Conditions for the Suzuki–Miyaura reaction: see ESI.† Conditions for the cyclization: see footnote *b* in Fig. 1. Isolated yields for the cyclization step are shown. ^*b*^See footnote *a* in Fig. 1 for the conditions used for the cyclization. ^*c*^NMR yield.

Given the numerous successful applications of fused thiophene scaffolds across a wide range of fields,^1^,^2^ it would be useful for the development of new functional molecules if our C–H/C–S coupling procedure could be used for the late-stage introduction of benzothiophene moieties to existing π-systems with characteristic properties. Pleasingly, we were able to accomplish this process using boronic acid **25** as an effective elaborating reagent (Scheme 3). Thus, it is possible to extend a wide variety of π-systems by fusing a benzothiophene ring through the Suzuki–Miyaura reaction with **25**, followed by ring closure *via* our C–H/C–S coupling. This whole process consists of two robust palladium-catalyzed reactions that do not require the use of any strong nucleophiles or oxidants, and therefore allows for the rapid modification of functionalized aromatic systems. For example, a bromophenyl group in 2,5-diaryloxadiazole motif can successfully participate in our two-step protocol to form benzothiophene-fused derivative **26**. Similarly, this protocol was found to be applicable to the π-extension of a range of useful compounds, such as anthraquinone **27**, amino acid **28** and BODIPY **29** derivatives.

**Scheme 4:**
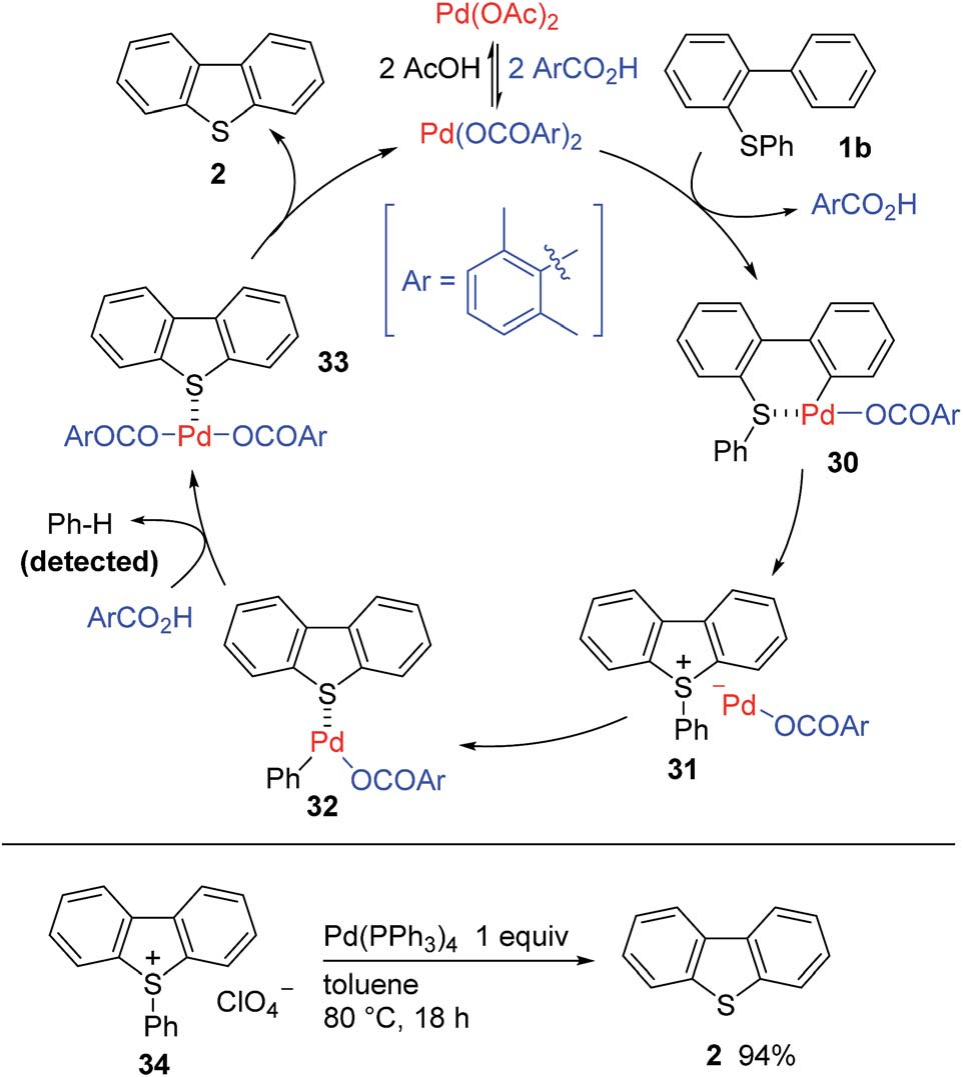
Possible mechanism.

Our current mechanistic proposal for the palladium-catalyzed C–H/C–S coupling is outlined in Scheme 4. Pd(OAc)_2_ would undergo ligand exchange with **3** to generate Pd(OCOAr)_2_,^16^ which would react with **1b** to form palladacycle **30***via* a sulfur-directed cyclometallation process.^10^ We hypothesized that the unusual C–S bond cleavage process would proceed through sulfonium intermediate **31**. Thus, a C–S bond-forming reductive elimination from **30**^17^ would provide ion pair **31** consisting of a dibenzosulfonium cation and an anionic Pd(0) fragment. The oxidative addition of the Ph–S bond in dibenzosulfonium^18^ to the Pd(0) center would lead to the cleavage of the C–S bond to give complex **32**. The cleaved phenyl group would be released as benzene following the protonolysis of **32** with **3**, which would be generated during the initial cyclometallation process, leading to the regeneration of Pd(OCOAr)_2_. Several experiments were conducted to support this mechanistic proposal. For example, the treatment of the independently synthesized sulfonium salt **34** with Pd(0) complex provided **2**, indicating the intermediacy of the sulfonium species in our catalytic reaction.^19^ Furthermore, we were able to confirm that benzene was generated (73% by GC) during the palladium-catalyzed reaction of **5**, which was consistent with our proposal. There were no significant differences between the initial reaction rates for the independent reactions of **1b** and deuterated **1b**, which indicated that the C–H bond cleavage (*i.e.*, **1b** → **30**) was not involved in the turnover-limiting step.^19^ Although no appreciable amounts of side product were observed in this C–H/C–S coupling, relatively high levels of catalyst loading (10–30 mol%) were currently required to obtain a high conversion. This requirement for a high catalyst loading could be attributed to the reluctance of the product **2** to dissociate from the Pd(ii) center (*i.e.*, **33** → **2**). In fact, the addition of **2** to the palladium-catalyzed C–H/C–S coupling reaction led to a decrease in the yield of the cyclized product by 30%.^19^ A characteristic feature of the mechanism of the C–H/C–S coupling is that the product is released by an oxidative addition step (**31** → **32**), which occurs *after* the reductive elimination step (**30** → **31**), and allows for the regeneration of the Pd(ii) species without the addition of an external oxidant. This mechanistic scenario is therefore unusual compared with the most common Pd(ii)-catalyzed processes, which typically end up with a product-forming reductive elimination step to generate a Pd(0) species and consequently require an external oxidant to regenerate Pd(ii).^20^

## Conclusions

In summary, we have developed a new C–H/C–S coupling strategy for the catalytic synthesis of dibenzothiophene derivatives. In contrast to previously reported methods for the synthesis of benzothiophene derivatives *via* C–H functionalization, our newly developed method does not require reactive functionalities such as Ar–X or S–H, or the addition of an external stoichiometric oxidant. This C–H/C–S coupling procedure is characterized by its unique mechanism, with the product being formed by an oxidative addition step, rather than a reductive elimination. Further studies towards the application of this mechanistic feature to the synthesis of other heterocycles are currently underway in our laboratories.

## Supplementary Material

Supplementary information
