# Supplementary material for: Palladium(ii)-catalyzed synthesis of dibenzothiophene derivatives via the cleavage of carbon–sulfur and carbon–hydrogen bonds
Source: Chem Sci. 2016 Jan 21;7(4):2587–91. doi: 10.1039/c5sc04890g (PMC5477049; doi:10.1039/c5sc04890g)
Supplement: Supplementary file 1 [file SC-007-C5SC04890G-s001.pdf]

Electronic Supplementary Material (ESI) for Chemical Science.
This journal is © The Royal Society of Chemistry 2016

Supporting Information
for
Palladium(II)-Catalyzed Synthesis of Dibenzothiophene Derivatives via the Cleavage of Carbon-Sulfur and Carbon-Hydrogen Bonds

Mamoru Tobisu*,† , Yoshihiro Masuya, Katsuaki Baba, and Naoto Chatani*

Department of Applied Chemistry, Faculty of Engineering, Osaka University, Suita, Osaka 565-0871, Japan

†*Center for Atomic and Molecular Technologies, Graduate School of Engineering, Osaka University, Suita, Osaka 565-0871, Japan*

tobisu@chem.eng.osaka-u.ac.jp; chatani@chem.eng.osaka-u.ac.jp

Contents

**I**	**General Information**	**S2**
**II**	**Materials**	**S2**
**III**	**Synthesis of Starting Materials**	**S2**
**IV**	**Typical Procedures for Palladium-Catalyzed Synthesis of Dibenzothiophenes via C-H/C-S Coupling**	**S12**
**V**	**Spectroscopic Data of Products Listed in Figure 1**	**S12**
**VI**	**Procedures for Experiments Shown in Scheme 2**	**S21**
**VII**	**Experimental Procedures for Scheme 3**	**S22**
**VIII**	**Mechanistic Studies**	**S29**
**IX**	**Copies of ^{1}H and ^{13}C NMR Spectra**	**S37**

I. General Information

^{1}H NMR and ^{13}C NMR spectra were recorded on a JEOL JMTC-400/54/ss spectrometer or VARIAN UNITY INOVA-600 spectrometer in $CDCl_3$ with tetramethylsilane as an internal reference standard. The NMR data have been reported as follows: chemical shift (δ) in ppm, multiplicity (s = singlet, d = doublet, t = triplet, quart = quartet, quint = quintet, m = multiplet and br = broad peak), coupling constant (Hz), and integration. Infrared spectra (IR) were obtained on a JASCO TF/IR-4000; absorptions have been reported in reciprocal centimeters with the following relative intensities: s (strong), m (medium), or w (weak). Mass spectra were recorded on a Shimadzu GCMS-QP 2010 instrument with an ionization voltage of 70 eV. High resolution mass spectra (HRMS) were obtained on a JEOL JMS-700 spectrometer. Analytical gas chromatography (GC) was carried out on Shimadzu GC-2014 gas chromatograph, equipped with a flame ionization detector. Melting points were determined using a Yamato melting point apparatus. Column chromatography was performed with SiO_2 [Merck SilicaGel 60 (230-400 mesh) or Silycycle Silica Flash F60 (230-400 mesh)]. Gel permeation chromatography (GPC) was performed on an LC-9210NEXT HPLC or LC9225NEXT HPLC system.

II. Materials

Unless otherwise noted, all of the reagents used in this study were obtained from commercial suppliers and used as received without further purification. $Pd(OAc)_2$ (CAS: 3375-31-3) was purchased from Wako Pure Chemical Industries, Ltd. 2,6-Dimethylbenzoic acid (**3**, CAS: 632-46-2) was purchased from Tokyo Chemical Industry Co., Ltd. Toluene was dried on a glass contour solvent-dispensing system (Nikko Hansen & Co., Ltd.). Compounds **1a**[1], **1b**,[2] **SM-7**,[3] and **SM-13**[4] were synthesized according to the reported procedures.

III. Synthesis of the Starting Materials

1 Guo, S.-R.; Yuan, Y.-Q. *Journal of Chemical Research*. **2009**, *12*, 745.
2 Park, N.; Park, K.; Jang, M.; Lee, S. *J. Org. Chem.* **2011**, *76*, 4371.
3 Liu, Y.; Wang, H.; Cao, X.; Fang, Z.; Wan, J.-P. *Synthesis* **2013**, *45*, 2977.
4 Asao, N.; Nogami, T.; Lee. S.; Yamamoto, Y. *J. Am. Chem. Soc.* **2003**, *125*, 10921.

General procedure for the Suzuki-Miyaura coupling of (2-bromophenyl)phenyl sulfide with a series of arylboronic acids.

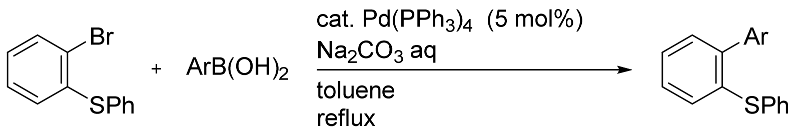

$Pd(PPh_3)_4$ (289 mg, 0.25 mmol), phenylboronic acid (914 mg, 7.5 mmol) and a saturated aqueous solution of Na_2CO_3 (12 mL) were added to a stirred solution of (2-bromophenyl)phenyl sulfideate[5] (1.32 g, 5.0 mmol) in toluene (24 mL), and the resulting mixture was refluxed overnight. The reaction mixture was then cooled to rt and partitioned between EtOAc and brine. The organic layer was collected, dried over Na_2SO_4, filtered and concentrated in vacuo to give a residue, which was purified by flash column chromatography to give [1,1'-biphenyl]-2-yl(phenyl)sulfane as a colorless oil (1.24 g, 95%).

Methyl 2'-(Phenylthio)-[1,1'-biphenyl]-4-carboxylate (SM-4).

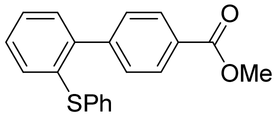

General procedure was followed on a 4 mmol scale except that 4-(methoxycarbonyl)phenylboronic acid was used instead of phenylboronic acid and 1,2-dimethoxyethane was used instead of toluene.

White solid (416 mg, 32%). Mp = 80 °C. Rf 0.40 (hexane/EtOAc = 10/1).

^{1}H NMR ($CDCl_3$, 399.78 MHz) δ: 3.93 (s, 3H), 7.23-7.31 (m, 9H), 7.47 (d, J = 8.7 Hz, 2H), 8.05 (d, J = 8.2 Hz, 2H).

^{13}C NMR ($CDCl_3$, 100.53 MHz) δ: 52.3, 127.2, 127.3, 128.7, 129.2, 129.3, 129.4, 129.6, 130.5, 131.7, 131.9, 134.8, 135.5, 142.3, 145.4, 167.1.

IR(ATR): 3056 w, 2949 w, 1718 s, 1462 w, 1435 m, 1275 s, 1180 w. 1102 m, 1071 w, 1005 w. 856 w, 827 w, 748 s, 705 m, 689 m.

MS, m/z (relative intensity, %): 320 (M^+, 100), 289 (M^+-31, 14), 152 (M^+-136, 17).

HRMS (EI): Calcd for $C_{20}H_{16}O_2S$ 320.0871, Found 320.0875.

2-Phenylthio-4'-acetylbiphenyl (SM-5).

[5] Wang, H.; Jiang, L.; Chen, T.; Li, Y. *Eur. J. Org. Chem.* **2010**, 2324.

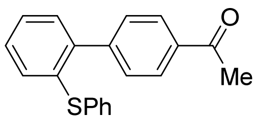

General procedure was followed on a 4 mmol scale except that 4-acetylphenylboronic acid was used instead of phenylboronic acid and DMAc used instead of toluene.

White solid (991 mg, 81%). Mp = 92 °C. Rf 0.30 (hexane/EtOAc = 20/1).

^{1}H NMR ($CDCl_3$, 399.78 MHz) δ: 2.63 (s, 3H), 7.22-7.31 (m, 9H), 7.50 (d, J = 8.0 Hz, 2H), 7.98 (d, J = 8.4 Hz, 2H).

^{13}C NMR ($CDCl_3$, 100.53 MHz) δ: 26.8, 127.2, 127.4, 128.2, 128.8, 129.3, 129.8, 130.5, 131.8, 131.9, 134.8, 135.4, 136.1, 142.2, 145.6, 198.0.

IR(ATR): 3055 w, 1681 s, 1605 m, 1581 w, 1462 m, 1436 m, 1400 m, 1357 m, 1263 s, 1038 w, 1023 w, 956 w, 837 m, 752 s, 691 s.

MS, m/z (relative intensity, %): 304 (M^+, 100), 289 (M^+-15, 52), 184 (M^+-120, 9).

HRMS (EI): Calcd for $C_{20}H_{16}OS$ 304.0922, Found 304.0923.

2-Phenylthio-4'-nitrobiphenyl (SM-6).

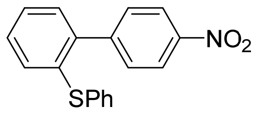

General procedure was followed on a 3 mmol scale except that 4-nitrophenylboronic acid, K_2CO_3 and 1,2-dimethoxyethane were used instead of phenylboronic acid, Na_2CO_3 and toluene, respectively.

Yellow solid (783 mg, 85%). Mp = 90 °C. Rf 0.29 (hexane/EtOAc = 20/1).

^{1}H NMR ($CDCl_3$, 399.78 MHz) δ: 7.16-7.36 (m, 9H), 7.53 (d, J = 8.7 Hz, 2H), 8.19-8.21 (m, 2H).

^{13}C NMR ($CDCl_3$, 150.9 MHz) δ: 123.1, 127.3, 127.5, 129.2, 129.3, 130.4 (two overlapping peaks), 131.3, 132.4, 134.5, 135.1, 141.1, 147.1, 147.3.

IR(ATR): 3058 w, 1598 m, 1514 s, 1476 w, 1461 m, 1437 w, 1400 w, 1345 s, 1178 w, 1161 w, 1107 w, 1024 w, 1005 w, 853 s, 744 s, 691 s.

MS, m/z (relative intensity, %): 307 (M^+, 100), 260 (M^+-47, 14), 184 (M^+-123, 11).

HRMS (EI): Calcd for $C_{18}H_{13}NO_2S$ 307.0667, Found 307.0668.

All of other substrates bearing an electron-withdrawing group reacted smoothly to give the desired products in high yields. It was therefore considered that the low yield obtained in the NO_2-containing substrate could be attributed to the poisoning of the catalyst by the NO_2 group, most likely through the coordination of this group to the

Pd(II) species. In this way, the NO_2 group would inhibit the binding of the substrate to the catalyst, thereby inhibiting the reaction, which would explain the low yield. To test this hypothesis, we investigated the impact of adding a single equivalent of nitrobenzene to the reaction of another substrate. The result of this reaction showed that there was a 3-fold decrease in the yield of the cyclized product (please see below). These results therefore clearly show that the presence of a nitro group in the reaction was detrimental to the catalytic process.

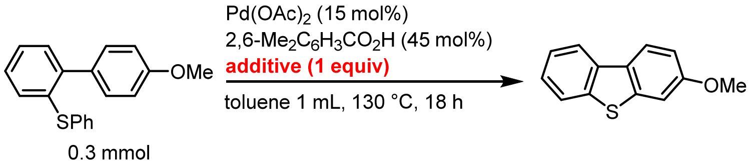

entry	additive	yield
1	none	97% (isolated)
2	*p*-nitrotoluene	33% (NMR)

2-Phenylthio-4'-hydroxybiphenyl (SM-8).

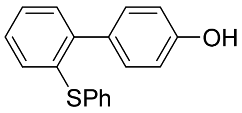

General procedure was followed on a 4 mmol scale except that 4-hydroxyphenylboronic acid was used instead of phenylboronic acid and 1,4-dioxane was used instead of toluene.

White solid (735 mg, 66%). Mp = 83 °C. Rf 0.23 (hexane/EtOAc = 5/2).

^{1}H NMR ($CDCl_3$, 399.78 MHz) δ: 4.83 (s, 1H), 6.84-6.87 (m, 2H), 7.19-7.31 (m, 11H).

^{13}C NMR ($CDCl_3$, 100.53 MHz) δ: 115.0, 126.9, 127.3, 127.9, 129.3, 130.7, 130.8, 131.2, 132.0, 133.3, 135.2, 135.6, 142.6, 155.1.

IR(ATR): 3379 w, 3053 w, 1612 m, 1590 w, 1517 m, 1442 m, 1258 m, 1205 m, 1173 m, 1125 w, 1099 w, 1038 w, 1021 w, 828 s, 752 s, 689 s.

MS, m/z (relative intensity, %): 278 (M^+, 100).

HRMS (EI): Calcd for $C_{18}H_{14}OS$ 278.0765, Found 278.0766.

2-Phenylthio-4'-*tert*-butylbiphenyl (SM-9).

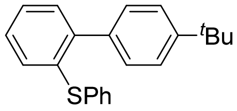

General procedure was followed on a 2 mmol scale except that 4-*tert*-butylphenylboronic acid was used instead of phenylboronic acid.

Colorless oil (548 mg, 86%). Rf 0.17 (hexane/EtOAc = 100/1).

^{1}H NMR ($CDCl_3$, 399.78 MHz) δ: 1.36 (s, 9H), 7.19-7.43 (m, 13H).

^{13}C NMR ($CDCl_3$, 100.53 MHz) δ: 31.5, 34.6, 125.0, 126.7, 127.2, 127.9, 129.1, 129.2, 130.7, 131.0, 132.1, 135.3, 135.7, 137.7, 142.8, 150.2.

IR(ATR): 3055 w, 2961 m, 2902 w, 2867 w, 1582 w, 1438 w, 1396 w, 1363 w, 1268 w, 1070 w, 1024 w, 834 w, 739 w, 690 w.

MS, m/z (relative intensity, %): 318 (M^+, 100), 303 (M^+-15, 43), 185 (M^+-133, 20).

HRMS (EI): Calcd for $C_{22}H_{22}S$ 318.1442, Found 318.1443.

2-Phenylthio-4'-fluorobiphenyl (SM-10).

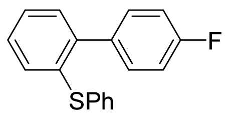

General procedure was followed on a 4 mmol scale except that 4-fluorophenylboronic acid was used instead of phenylboronic acid and K_3PO_4 was used instead of Na_2CO_3.

Colorless oil (821 mg, 75%). Rf 0.29 (hexane/EtOAc = 20/1).

^{1}H NMR ($CDCl_3$, 399.78 MHz) δ: 7.04-7.08 (m, 2H), 7.22-7.28 (m, 9H), 7.33-7.37 (m, 2H).

^{13}C NMR ($CDCl_3$, 100.53 MHz) δ: 115.3, 115.5, 127.6 (d, J = 20.1 Hz), 128.7, 129.7, 131.1, 131.5 (d, J = 7.6 Hz), 132.0, 132.2, 135.5, 136.0, 137.1 (d, J = 2.8 Hz), 142.6, 162.8 (d, J = 247.3 Hz).

IR(ATR): 3056 w, 1605 w, 1582 w, 1511 s, 1461 m, 1438 w, 1222 s, 1158 m, 1092 w, 1070 w, 1023 w, 834 s, 752 s, 736 s, 690 s.

MS, m/z (relative intensity, %): 280 (M^+, 100), 202 (M^+-78, 17), 170 (M^+-110, 14).

HRMS (EI): Calcd for $C_{18}H_{13}FS$ 280.0722, Found 280.0720.

2-Phenylthio-4'-chlorobiphenyl (SM-11).

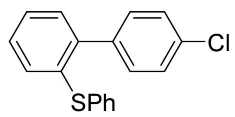

General procedure was followed on a 4 mmol scale except that 4-chlorophenylboronic acid, K_2CO_3 and 1,4-dioxane were used instead of phenylboronic acid, Na_2CO_3, and toluene, respectively.

White solid (553 mg, 47%). Mp = 63 °C. Rf 0.31 (hexane).

^{1}H NMR ($CDCl_3$, 399.78 MHz) δ: 7.20-7.36 (m, 13H).

^{13}C NMR ($CDCl_3$, 100.53 MHz) δ: 127.1, 127.3, 128.2, 128.4, 129.2, 130.5, 130.8, 131.6, 131.7, 133.5, 134.9, 135.4, 139.0, 141.9.

IR(ATR): 3048 w, 1581 w, 1460 m, 1437 w, 1394 w, 1090 m, 1021 w, 1003 m, 828 s, 751 s, 688 s.

MS, m/z (relative intensity, %): 296 (M^+, 100), 260 (M^+-36, 22), 184 (M^+-112, 22).

HRMS (EI): Calcd for $C_{18}H_{13}ClS$ 296.0426, Found 296.0425.

2-Phenylthio-3'-methylbiphenyl (SM-12).

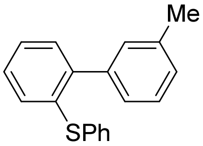

General procedure was followed on a 4 mmol scale except that 3-tolylboronic acid was used instead of phenylboronic acid.

Colorless oil (652 mg, 59%). Rf 0.20 (hexane).

^{1}H NMR ($CDCl_3$, 399.78 MHz) δ: 2.38 (s, 3H), 7.18-7.28 (m, 13H).

^{13}C NMR ($CDCl_3$, 100.53 MHz) δ: 21.6, 126.5, 126.7, 127.3, 127.9, 128.0, 128.3, 129.2, 130.2, 130.6, 131.0, 132.2, 135.2, 135.7, 137.6, 140.6, 143.1.

IR(ATR): 3054 w, 2919 w, 1605 w, 1581 w, 1461 m, 1438 m, 1073 w, 1039 w, 1024 w, 1000 w, 884 w, 788 m, 749 s, 702 s, 690 s.

MS, m/z (relative intensity, %): 276 (M^+, 100), 261 (M^+-15, 12), 184 (M^+-92, 20).

HRMS (EI): Calcd for $C_{19}H_{16}S$ 276.0973, Found 276.0971.

Methyl 6-(phenylthio)-[1,1'-biphenyl]3-carboxylate (SM-14).

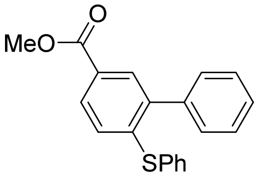

General procedure was followed on a 2.8 mmol scale except that methyl 3-bromo-4-(phenylthio)benzoate[6] was used instead of (2-bromophenyl)phenyl sulfide.

White solid (658 mg, 75%). Mp = 101 °C. Rf 0.29 (hexane/EtOAc = 10/1).

^{1}H NMR ($CDCl_3$, 399.78 MHz) δ: 3.89 (s, 3H), 6.98 (d, J = 8.7 Hz, 1H), 7.36-7.48 (m, 10H), 7.78-7.81 (m, 1H), 7.91-7.92 (m, 1H)

^{13}C NMR ($CDCl_3$, 100.53 MHz) δ: 52.3, 127.3, 127.6, 128.1, 128.4, 128.86, 128.89, 129.4, 129.8, 131.3, 132.6, 134.5, 139.6, 140.7, 143.7, 166.9.

IR(ATR): 2947 w, 1710 s, 1593 m, 1434 m, 1400 w, 1301 m, 1276 m, 1236 s, 1190 w, 1118 m, 1070 w, 1021 m, 973 w, 923 w, 850 w, 750 s, 704 s.

MS, m/z (relative intensity, %): 320 (M^+, 100), 289 (M^+-31, 22), 152 (M^+-168, 18).
HRMS (EI): Calcd for $C_{20}H_{16}O_2S$ 320.0871, Found 320.0869.

5-Chloro-2-phenylthiobiphenyl (SM-15).

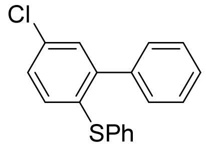

General procedure was followed on a 7.1 mmol scale except that 2-bromo-4-chloro-1-(phenylthio)benzene[6] was used instead of (2-bromophenyl)phenyl sulfide.
Colorless oil (938 mg, 44%). Rf 0.36 (hexane).
^{1}H NMR ($CDCl_3$, 399.78 MHz) δ: 7.10-7.13 (m, 1H), 7.18-7.21 (m, 1H), 7.24-7.31 (m, 6H), 7.37-7.42 (m, 5H).
^{13}C NMR ($CDCl_3$, 100.53 MHz) δ: 127.6, 128.0, 128.1, 128.2, 129.3, 129.4, 130.4, 132.1, 132.3, 132.6, 134.0, 135.0, 139.4, 144.3.
IR(ATR):3057 w, 1577 w, 1546 w, 1474 w, 1440 m, 1381 w, 1245 w, 1139 w, 1101 m, 1016 m, 883 w, 813 m, 764 m, 741 m, 695 s.
MS, m/z (relative intensity, %): 296 (M^+, 100), 260 (M^+-36, 21), 184 (M^+-112, 24).
HRMS (EI): Calcd for $C_{18}H_{13}ClS$ 296.0426, Found 296.0424.

(3',4'-dichloro-[1,1'-biphenyl]-2-yl)(phenyl)sulfane (SM-16).

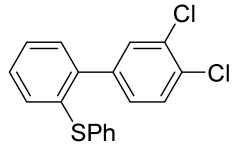

General procedure was followed on a 4 mmol scale except that 3,4-dichlorophenylboronic acid, and 1,2-dimethoxyethane were used instead of phenylboronic acid and toluene, respectively.
Colorless oil (595 mg, 45%). Rf 0.29(hexane).
^{1}H NMR ($CDCl_3$, 399.78 MHz) δ: 7.21-7.31 (m, 10H), 7.42-7.45 (m, 2H).
^{13}C NMR ($CDCl_3$, 100.53 MHz) δ: 127.3, 127.4, 128.8, 128.9, 129.3, 129.9, 130.5, 131.4, 131.7, 131.8, 131.9, 132.1, 135.0, 135.2, 140.6, 140.7.
IR(ATR):3058 w, 1581 w, 1475 m, 1454 s, 1373 m, 1325 m, 1131 m, 1074 w, 1027 m, 886 w, 822 m, 751 s, 690 s.

---

[6] Su, K.; Qiu, Y.; Yao, Y.; Zhang, D.; Jiang, S. *Synlett* **2012**, *23*, 2853.

MS, m/z (relative intensity, %): 330 (M^+, 100), 294 (M^+-36, 19), 260 (M^+-70, 14), 218 (M^+-112, 22).

HRMS (EI): Calcd for $C_{18}H_{12}Cl_2S$ 330.0037, Found 330.0038.

Phenyl(3',4',5-trichloro-[1,1'-biphenyl]-2-yl)sulfane (SM-17).

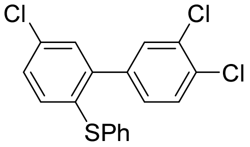

General procedure was followed on a 4 mmol scale except that 2-bromo-4-chloro-1-(phenylthio)benzene[6] , 3,4-dichlorophenylboronic acid, and 1,2-dimethoxyethane were used instead of (2-bromophenyl)phenyl sulfide, phenylboronic acid and toluene, respectively.

Colorless oil (1.04 g, 71%). Rf 0.34(hexane).

^{1}H NMR ($CDCl_3$, 399.78 MHz) δ: 7.16-7.31 (m, 9H), 7.43-7.45 (m, 2H).

^{13}C NMR ($CDCl_3$, 100.53 MHz) δ: 127.8, 128.7, 128.8, 129.4, 130.1, 130.3, 131.2, 132.0, 132.2, 132.3, 132.9, 133.0, 133.9, 134.5, 139.2, 141.9.

IR(ATR):3060 w, 2926 w, 1579 w, 1544 w, 1475 m, 1450 s, 1368 m, 1252 m, 1134 m, 1102 m, 1030 s, 878 m, 856 m, 819 s, 744 s, 690 s.

MS, m/z (relative intensity, %): 366 (M^++2, 100), 364 (M^+, 97), 330 (M^+-34, 13), 328 (M^+-36, 13), 298 (M^+-66, 17), 296 (M^+-68, 16), 294 (M^+-70, 18).

HRMS (EI): Calcd for $C_{18}H_{11}Cl_3S$ 363.9647, Found 363.9647.

(5-chloro-4'-methoxy-[1,1'-biphenyl]-2-yl)(phenyl)sulfane (SM-18).

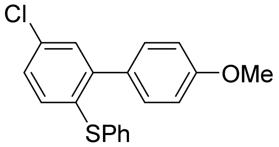

General procedure was followed on a 4 mmol scale except that 2-bromo-4-chloro-1-(phenylthio)benzene[6] and 4-methoxyphenylboronic acid were used instead of (2-bromophenyl)phenyl sulfide, phenylboronic acid, respectively.

Colorless oil (836 mg, 64%). Rf 0.46(hexane/EtOAc = 10/1).

^{1}H NMR ($CDCl_3$, 399.78 MHz) δ: 3.83 (s, 3H), 6.94 (dd, J = 6.4 Hz, 2.3 Hz, 2H), 7.11 (d, J = 8.7 Hz, 1H), 7.15-7.18 (m, 1H), 7.23-7.35 (m, 8H).

^{13}C NMR ($CDCl_3$, 100.53 MHz) δ: 55.3, 113.6, 114.2, 127.6, 127.8, 129.4, 130.5, 131.8, 132.1, 132.2, 132.5, 134.0, 135.1, 144.0, 159.4.

IR(ATR):3059 w, 2931 w, 2835 w, 1608 m, 1577 w, 1512 m, 1452 m, 1381 w, 1294 m, 1246 s, 1177 m, 1103 m, 1037 m, 1023 m, 885 w, 826 m, 773 m, 747 m, 691 m.

MS, m/z (relative intensity, %): 328 (M^{+}+2, 38), 326 (M^{+}, 100), 298 (M^{+}-28, 19), 214 (M^{+}-112, 29).

HRMS (EI): Calcd for $C_{19}H_{15}ClOS$ 326.0532, Found 326.0531.

1-(5'-chloro-2'-(phenylthio)-[1,1'-biphenyl])-4yl)ethane-1-one (SM-19).

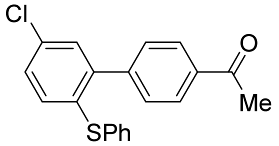

General procedure was followed on a 4 mmol scale except that 2-bromo-4-chloro-1-(phenylthio)benzene[6], 4-acetylphenylboronic acid, and DMA were used instead of (2-bromophenyl)phenyl sulfide, phenylboronic acid, and toluene, respectively.

Colorless oil (620 mg, 46%). Rf 0.29(hexane/EtOAc = 10/1).

^{1}H NMR ($CDCl_3$, 399.78 MHz) δ: 2.64 (s, 3H), 7.17-7.30 (m, 8H), 7.47-7.49 (m, 2H), 7.97-7.99 (m, 2H).

^{13}C NMR ($CDCl_3$, 100.53 MHz) δ: 26.7, 127.6, 128.2, 128.7, 129.4, 129.6, 130.2, 131.8, 132.9, 132.9, 133.6, 134.7, 136.4, 143.3, 144.1, 197.6.

IR(ATR):3057 w, 1683 s, 1605 w, 1579 w, 1453 w, 1405 w, 1358 w, 1264 s, 1103 w, 1024 w, 957 w, 885 w, 821 m, 739 m, 691 m.

MS, m/z (relative intensity, %): 338 (M^{+}, 100), 323 (M^{+}-15, 49), 260 (M^{+}-78, 26).

HRMS (EI): Calcd for $C_{20}H_{15}ClOS$ 338.0532, Found 338.0529.

Ethyl 5'-chloro-2'-(phenylthio)-[1,1'-biphenyl]-4-carboxylate (SM-20).

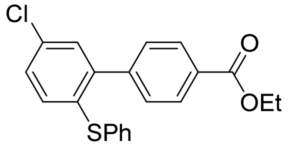

General procedure was followed on a 4 mmol scale except that 2-bromo-4-chloro-1-(phenylthio)benzene[6], 4-(ethoxycarbonyl)phenylboronic acid, and 1,2-dimethoxyethane were used instead of (2-bromophenyl)phenyl sulfide, phenylboronic acid, and toluene, respectively.

Colorless oil (1.01 g, 87%). Rf 0.36(hexane/EtOAc = 10/1).

^{1}H NMR ($CDCl_3$, 399.78 MHz) δ: 1.41 (s, J = 7.3 Hz, 3H), 4.40 (dd, J = 14.2 Hz, 6.9 Hz, 2H), 7.17-7.30 (m, 8H), 7.43-7.46 (m, 2H), 8.05-8.08 (m, 2H).

^{13}C NMR ($CDCl_3$, 100.53 MHz) δ: 14.4, 61.0, 127.5, 128.6, 129.3, 129.3, 129.9, 130.2, 131.7, 132.8, 132.9, 133.6, 134.8, 143.5, 143.8, 166.2.
IR(ATR):3059 w, 2980 w, 1714 s, 1609 w, 1579 w, 1453 w, 1367 w, 1271 s, 1178 w, 1101 s, 1026 w, 1015 w, 856 w, 814 m, 773 m, 745 m, 705 m, 690 m.
MS, m/z (relative intensity, %): 368 (M^+, 100), 323 (M^+-45, 19), 260 (M^+-108, 21).
HRMS (EI): Calcd for $C_{21}H_{17}ClO_2S$ 368.0638, Found 338.0635.

1-(4'-Methoxy-6-(phenylthio)-[1,1'-biphenyl]-3yl)ethane-1-one (SM-21).

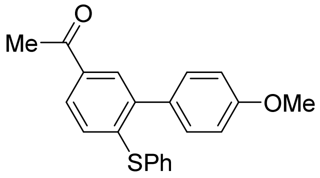

General procedure was followed on a 1.7 mmol scale except that 4-methoxyphenylboronic acid was used instead of phenylboronic acid and 1-(3-bromo-4-(phenylthio)phenyl)ethane-1-one was used instead of (2-bromophenyl)phenyl sulfideate.
Pale yellow oil (426 mg, 75%). Rf 0.21 (hexane/EtOAc = 10/1).
^1H NMR ($CDCl_3$, 399.78 MHz) δ: 2.56 (s, 3H), 3.87 (s, 3H), 6.95-7.01 (m, 3H), 7.37-7.46 (m, 7H), 7.69-7.71 (m, 1H), 7.80 (d, J = 1.8 Hz, 1H)
^{13}C NMR ($CDCl_3$, 100.53 MHz) δ: 26.6, 55.4, 113.8, 127.3 (two overlapping peaks), 129.0, 129.8, 130.1, 130.6, 131.9, 132.4, 134.3, 134.6, 140.3, 144.4, 159.5, 197.4.
IR(ATR): 3002 w, 2836 w, 1679 m, 1586 m, 1512 m, 1462 m, 1299 m, 1245 s, 1232 s, 1177 m, 1035 m, 1024 m, 961 m, 831 m, 751 m.
MS, m/z (relative intensity, %): 334 (M^+, 100), 319 (M^+-15, 41).
HRMS (EI): Calcd for $C_{21}H_{18}O_2S$ 334.1028, Found 334.1031.

3-(2-(Phenylthio)phenyl)thiophene (SM-22).

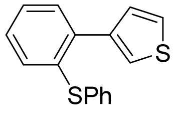

General procedure was followed on a 3.8 mmol scale except that 3-thiopheneboronic acid, K_2CO_3 and 1,4-dioxane were used instead of phenylboronic acid, Na_2CO_3, and toluene, respectively.
Colorless oil (612 mg, 60%). Rf 0.24 (hexane).
^1H NMR ($CDCl_3$, 399.78 MHz) δ: 7.21-7.29 (m, 9H), 7.32-7.34 (m, 2H), 7.37-7.39 (m, 1H).

^{13}C NMR ($CDCl_3$, 100.53 MHz) δ: 123.9, 124.9, 127.0, 127.2, 128.1, 129.0, 129.3, 130.6, 131.6, 131.7, 135.0, 135.6, 137.8, 140.8.

IR(ATR):3102 w, 3055 w, 3019 w, 1581 w, 1472 w, 1437 w, 1262 w, 1192 w, 1071 w, 1038 w, 1024 w, 856 w, 787 m, 744 s, 688 s.

MS, m/z (relative intensity, %): 268 (M^+, 100), 235 (M^+-33, 60), 190 (M^+-78, 18).

HRMS (EI): Calcd for $C_{16}H_{12}S_2$ 268.0380, Found 268.0379.

2-Phenylselenobiphenyl (SM-23) [CAS: 126146-85-8].[7]

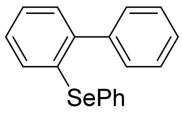

A two-necked flask was charged with diphenyl diselenide (1.1 g, 3.5 mmol), 2-iodobiphenyl (1.4g , 5 mmol), CuI (47.6 mg, 0.25 mmol), Cs_2CO_3 (4.89 g, 15 mmol) and acetonitrile (25 mL), and the resulting solution was heated at 82 °C for 28 h. The reaction mixture was then cooled to rt and partitioned between EtOAc and brine. The organic layer was collected, dried over $MgSO_4$, filtered, and concentrated in vacuo to give a residue, which was purified by flash column chromatography (hexane, Rf = 0.27) to give as an yellow oil (572 mg, 37%).

^1H NMR ($CDCl_3$, 399.78 MHz) δ: 7.21-7.29 (m, 9H), 7.32-7.34 (m, 2H), 7.37-7.39 (m, 1H).

^{13}C NMR ($CDCl_3$, 100.53 MHz) δ: 123.9, 124.9, 127.0, 127.2, 128.1, 129.0, 129.3, 130.6, 131.6, 131.7, 135.0, 135.6, 137.8, 140.8.

IR(ATR):3102 w, 3055 w, 3019 w, 1581 w, 1472 w, 1437 w, 1262 w, 1192 w, 1071 w, 1038 w, 1024 w, 856 w, 787 m, 744 s, 688 s.

MS, m/z (relative intensity, %): 268 (M^+, 100), 235 (M^+-33, 60), 190 (M^+-78, 18).

HRMS (EI): Calcd for $C_{16}H_{12}S_2$ 268.0380, Found 268.0379.

IV. Typical Procedure for the Palladium-Catalyzed Synthesis of Dibenzothiophenes via C-H/C-S Coupling (Table 1, Entry 6)

An oven-dried 5 mL screw-capped vial was charged with $Pd(OAc)_2$ (10 mg, 0.045 mmol), [1,1'-biphenyl]-2-yl(phenyl)sulfane (79 mg, 0.30 mmol), 2,6-dimethylbenzoic acid (**3**, 20 mg, 0.14 mmol) and toluene (1.0 mL) under a gentle stream of nitrogen. The vessel was then sealed

---

[7] Dandapat, A.; Korupalli, C. Prasad, D. J. C.; Singh, R.; Sekar, G. *Synthesis* **2011**, *14*, 2297.

and heated at 130 °C for 18 h. The mixture was cooled to rt and filtered through a short pad of silica gel, eluting with EtOAc. The eluent was evaporated, and the residue was purified by flash chromatography (hexane) to give dibenzothiophene as a white solid (44 mg, 79%).

V. Spectroscopic Data of Products Listed in Figure 1

Dibenzothiophene (2) [CAS: 132-65-0].

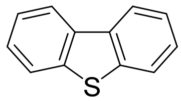

White solid (44 mg, 79%). Rf 0.43 (hexane).

^{1}H NMR ($CDCl_3$, 399.78 MHz): δ 7.44-7.48 (m, 4H), 7.85-7.87 (m, 2H), 8.16-8.18 (m, 2H).

^{13}C NMR ($CDCl_3$, 100.53 MHz): δ 121.7, 122.9, 124.5, 126.8, 135.6, 139.5.

HRMS (EI): Calcd for $C_{12}H_8S$ 184.0347, Found 184.0346.

Methyldibenzo[b,d]thiophene-3-carboxylate (4) [CAS: 60718-96-9].

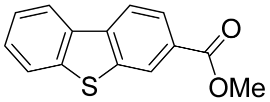

General procedure was followed except that Methyl 2’-(phenylthio)-[1,1’-biphenyl]-4-carboxylate was used instead of [1,1'-biphenyl]-2-yl(phenyl)sulfane.

White solid (68 mg, 94%). Rf 0.57 (hexane/EtOAc = 10/1).

^{1}H NMR ($CDCl_3$, 399.78 MHz): δ 3.99 (s, 3H), 7.50-7.54 (m, 2H), 7.89-7.91 (m, 1H), 8.12-8.14 (m, 1H), 8.20-8.23 (m, 2H), 8.58 (d, J = 0.9 Hz, 1H).

^{13}C NMR ($CDCl_3$, 100.53 MHz): δ 52.5, 121.4, 122.6, 123.1, 124.82, 124.84, 125.6, 127.9, 128.4, 134.8, 139.3, 139.4, 141.1, 167.0.

HRMS (EI): Calcd for $C_{14}H_{10}O_2S$ 242.0402, Found 242.0401.

3-Acetyldibenzothiophene (5) [CAS: 5337-07-2]

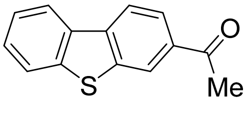

General procedure was followed except that 2-Phenylthio-4'-acetylbiphenyl was used instead of [1,1'-biphenyl]-2-yl(phenyl)sulfane.

White solid (67 mg, 98%). Rf 0.31 (hexane/EtOAc = 10/1).

^{1}H NMR ($CDCl_3$, 399.78 MHz): δ 2.70 (s, 3H), 7.49-7.53 (m, 2H), 7.87-7.89 (m, 1H), 8.02-8.05 (m, 1H), 8.17-8.20 (m, 2H), 8.46 (t, J = 0.8 Hz, 1H).

^{13}C NMR ($CDCl_3$, 100.53 MHz): δ 26.8, 121.4, 122.4, 122.9, 123.4, 124.3, 124.7, 127.8, 134.5, 135.2, 139.1, 139.4, 141.1, 197.4.

HRMS (EI): Calcd for $C_{14}H_{10}OS$ 226.0452, Found 226.0452.

3-Nitrodibenzothiophene (6) [CAS: 94764-55-3].

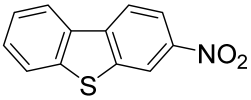

General procedure was followed except that 2-Phenylthio-4'-nitrobiphenyl was used instead of [1,1'-biphenyl]-2-yl(phenyl)sulfane. $Pd(OAc)_2$ (20 mg, 0.09 mmol) and 2,6-dimethylbenzoic acid (40 mg, 0.27 mmol) were used respectively.

Yellow solid (23 mg, 33%). Rf 0.25 (hexane/EtOAc = 20/1).

^{1}H NMR ($CDCl_3$, 399.78 MHz): δ 7.53-7.61 (m, 2H), 7.93 (d, J = 7.8 Hz, 1H), 8.23-8.34 (m, 3H), 8.77 (d, J = 1.8 Hz, 1H).

^{13}C NMR ($CDCl_3$, 100.53 MHz): δ 119.0, 119.8, 121.8, 123.0, 123.3, 125.4, 128.8, 133.9, 139.8, 140.5, 141.9, 146.4.

HRMS (EI): Calcd for $C_{14}H_{10}OS$ 226.0452, Found 226.0452.

All of other substrates bearing an electron-withdrawing group reacted smoothly to give the desired products in high yields. It was therefore considered that the low yield obtained in the NO_2-containing substrate could be attributed to the poisoning of the catalyst by the NO_2 group, most likely through the coordination of this group to the Pd(II) species. In this way, the NO_2 group would inhibit the binding of the substrate to the catalyst, thereby inhibiting the reaction, which would explain the low yield. To test this hypothesis, we investigated the impact of adding a single equivalent of nitrobenzene to the reaction of another substrate. The result of this reaction showed that there was a 3-fold decrease in the yield of the cyclized product (see below). These results therefore clearly show that the presence of a nitro group in the reaction was detrimental to the catalytic process.

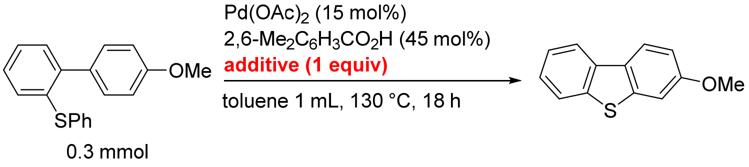

entry	additive	yield
1	none	97% (isolated)
2	*p*-nitrotoluene	33% (NMR)

3-Methoxybenzothiophene (7) [CAS: 54815-67-7].

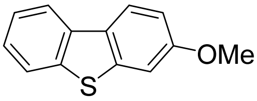

General procedure was followed except that 2-Phenylthio-4’-methoxybiphenyl was used instead of [1,1'-biphenyl]-2-yl(phenyl)sulfane.

White solid (63 mg, 97%). Rf 0.36 (hexane).

^{1}H NMR ($CDCl_3$, 399.78 MHz): δ 3.89 (s, 3H), 7.02-7.05 (m, 1H), 7.31-7.43 (m, 3H), 7.78-7.80 (m, 1H), 7.99-8.03 (m, 2H).

^{13}C NMR ($CDCl_3$, 100.53 MHz): δ 55.7, 105.9, 113.6, 120.9, 122.4, 122.8, 124.5, 125.6, 129.2, 135.6, 138.7, 141.1, 159.1.

HRMS (EI): Calcd for $C_{13}H_{10}OS$ 214.0452, Found 214.0453.

Dibenzothiophene-3-ol (8) [CAS: 69747-77-9].

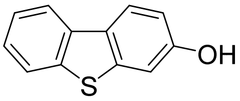

General procedure was followed except that 2-Phenylthio-4’-hydroxybiphenyl was used instead of [1,1'-biphenyl]-2-yl(phenyl)sulfane. $Pd(OAc)_2$ (20 mg, 0.09 mmol) and 2,6-dimethylbenzoic acid (40 mg, 0.27 mmol) were used respectively.

White solid (38 mg, 63%). Rf 0.51 (hexane/EtOAc = 5/2).

^{1}H NMR ($CDCl_3$, 399.78 MHz): δ 5.04 (s, 1H), 6.96-6.99 (m, 1H), 7.29 (d, J = 2.4 Hz, 1H), 7.38-7.43 (m, 2H), 7.80 (d, J = 7.2 Hz, 1H), 7.99-8.04 (m, 2H).

^{13}C NMR ($CDCl_3$, 100.53 MHz): δ 108.6, 113.7, 120.9, 122.6, 122.8, 124.6, 125.8, 129.5, 135.5, 138.7, 141.2, 155.0.

HRMS (EI): Calcd for $C_{12}H_8OS$ 200.0296, Found 200.0294.

3-*tert*-Butyldibenzothiophene (9) [CAS: 147792-07-2].

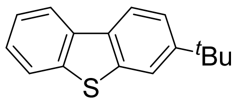

General procedure was followed except that 2-Phenylthio-4'-*tert*-butylbiphenyl was used instead of [1,1'-biphenyl]-2-yl(phenyl)sulfane.

White solid (50 mg, 69%). Rf 0.43 (hexane).

^{1}H NMR ($CDCl_3$, 399.78 MHz): δ 1.41 (s, 9H), 7.41-7.43 (m, 2H), 7.49-7.52 (m, 1H), 7.82-7.85 (m, 2H), 8.06-8.12 (m, 2H).

^{13}C NMR ($CDCl_3$, 100.53 MHz): δ 31.7, 35.2, 119.2, 121.2, 121.4, 122.5, 122.9, 124.4, 126.3, 133.2, 135.6, 139.5, 139.7, 150.3.

HRMS (EI): Calcd for $C_{16}H_{16}S$ 240.0973, Found 240.0971.

3-Fluorodibenzothiophene (10) [CAS: 169690-06-6].

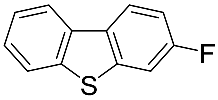

General procedure was followed except that 2-Phenylthio-4'-fluorobiphenyl was used instead of [1,1'-biphenyl]-2-yl(phenyl)sulfane.

White solid (57 mg, 94%). Rf 0.51 (hexane).

^{1}H NMR ($CDCl_3$, 399.78 MHz): δ 7.19 (td, J = 8.7, 2.3 Hz, 1H), 7.43-7.48 (m, 2H), 7.54 (dd, J = 4.3, 2.3 Hz, 1H), 7.82-7.85 (m, 1H), 8.07-8.11 (m, 2H).

^{13}C NMR ($CDCl_3$, 100.53 MHz): δ 109.3 (d, J = 24.9 Hz), 113.0 (d, J = 24.0 Hz), 121.4, 122.7 (d, J = 9.7 Hz), 122.9, 124.7, 126.5, 132.0, 134.9, 139.3, 140.8 (d, J = 10.6 Hz), 161.9 (d, J = 246.3 Hz).

HRMS (EI): Calcd for $C_{12}H_7FS$ 202.0252, Found 202.0251.

3-Chlorodibenzothiophene (11) [CAS: 109014-35-9].

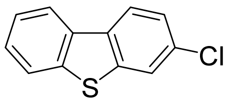

General procedure was followed except that 2-Phenylthio-4'-chlorobiphenyl was used instead of [1,1'-biphenyl]-2-yl(phenyl)sulfane.

White solid (64 mg, 98%). Rf 0.56 (hexane).

^{1}H NMR ($CDCl_3$, 399.78 MHz): δ 7.41-7.48 (m, 3H), 7.83-7.86 (m, 2H), 8.06 (d, J = 8.4 Hz, 1H), 8.10-8.13 (m, 1H).

^{13}C NMR ($CDCl_3$, 100.53 MHz): δ 121.7, 122.4, 122.6, 122.9, 124.8, 125.2, 127.1, 132.6, 134.1, 134.8, 139.5, 140.7.

HRMS (EI): Calcd for $C_{12}H_7ClS$ 217.9957, Found 217.9958.

2-Methyldibenzothiophene (12) [CAS: 20928-02-3].

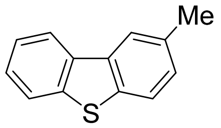

General procedure was followed except that 2-Phenylthio-3'-methylbiphenyl was used instead of [1,1'-biphenyl]-2-yl(phenyl)sulfane. $Pd(OAc)_2$ (20 mg, 0.09 mmol) and 2,6-dimethylbenzoic acid (40 mg, 0.27 mmol) were used respectively.

White solid (36 mg, 60%). Rf 0.51 (hexane).

^{1}H NMR ($CDCl_3$, 399.78 MHz): δ 2.54 (s, 3H), 7.29 (d, J = 8.0 Hz, 1H), 7.43-7.45 (m, 2H), 7.73 (d, J = 8.0 Hz, 1H), 7.82-7.84 (m, 1H), 7.97 (s, 1H), 8.12-8.14 (m, 1H).

^{13}C NMR ($CDCl_3$, 100.53 MHz): δ 21.7, 121.6, 121.9, 122.6, 123.0, 124.3, 126.7, 128.4, 134.3, 135.6, 135.8, 136.5, 139.9.

HRMS (EI): Calcd for $C_{13}H_{10}S$ 198.0503, Found 198.0502.

Benzo[b]naphtha[2,3-d]thiophene (13) [CAS: 243-46-9].

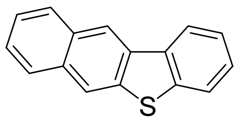

General procedure was followed except that **SM-13** was used instead of [1,1'-biphenyl]-2-yl(phenyl)sulfane.

White solid (53 mg, 75%). Rf 0.29 (hexane/EtOAc = 50/1).

^{1}H NMR ($CDCl_3$, 399.78 MHz): δ 7.48-7.54 (m, 4H), 7.82-7.85 (m, 1 H), 7.91-7.94 (m, 1H), 8.04-8.06 (m, 1H), 8.27-8.30 (m, 2H), 8.64 (s, 1H).

^{13}C NMR ($CDCl_3$, 100.53 MHz): δ 120.1, 120.8, 122.1, 123.0, 124.7, 125.3, 126.1, 127.2, 127.8, 128.5, 131.0, 132.7, 135.2, 137.8, 140.2.

HRMS (EI): Calcd for $C_{16}H_{10}S$ 234.0503, Found 234.0507.

Methyldibenzo[b,d]thiophene-2-carboxylate (14) [CAS: 22099-28-1].

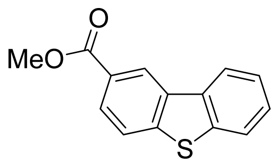

General procedure was followed except that Methyl 6-(phenylthio)-[1,1'-biphenyl]3-carboxylate was used instead of [1,1'-biphenyl]-2-yl(phenyl)sulfane. Pd(OAc)$_2$ (20 mg, 0.09 mmol) and 2,6-dimethylbenzoic acid (40 mg, 0.27 mmol) were used respectively.

White solid (72 mg, 98%). Rf 0.42 (hexane/EtOAc = 10/1).

^{1}H NMR (CDCl$_3$, 399.78 MHz): δ 4.00 (s, 3H), 7.49-7.53 (m, 2H), 7.86-7.91 (m, 2H), 8.11-8.14 (m, 1H), 8.24-8.26 (m, 1H), 8.85 (d, J = 1.4 Hz, 1H).

^{13}C NMR (CDCl$_3$, 100.53 MHz): δ 52.4, 122.1, 122.7, 123.0, 123.3, 125.0, 126.6, 127.4, 127.5, 135.3, 135.7, 139.8, 144.5, 167.4.

HRMS (EI): Calcd for $C_{14}H_{10}O_2S$ 242.0402, Found 242.0402.

2-Chlorodibenzothiophene (15) [CAS: 68820-91-7].

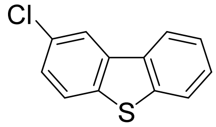

General procedure was followed except that 5-Chloro-2-phenylthiobiphenyl was used instead of [1,1'-biphenyl]-2-yl(phenyl)sulfane.

White solid (60 mg, 90%). Rf 0.50 (hexane).

^{1}H NMR (CDCl$_3$, 399.78 MHz): δ 7.42-7.50 (m, 3H), 7.77 (d, J = 8.8 Hz, 1H), 7.85-7.87 (m, 1H), 8.10-8.13 (m, 2H).

^{13}C NMR (CDCl$_3$, 100.53 MHz): δ 121.6, 121.9, 123.0, 123.9, 124.7, 127.0, 127.5, 130.8, 134.6, 137.0, 137.6, 140.2.

HRMS (EI): Calcd for $C_{12}H_7ClS$ 217.9957, Found 217.9959.

2,3-Dichlorodibenzothiophene (16) [CAS: 230308-30-2].

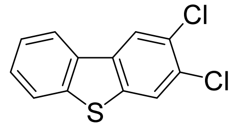

General procedure was followed except that (3',4'-dichloro-[1,1'-biphenyl]-2-yl)(phenyl)sulfane was used instead of [1,1'-biphenyl]-2-yl(phenyl)sulfane.

White solid (78 mg, 97%). Rf 0.49 (hexane).

^{1}H NMR ($CDCl_3$, 399.78 MHz): δ 7.46-7.52 (m, 2H), 7.83-7.85 (m, 1H), 7.92 (s, 1H), 8.06-8.08 (m, 1H), 8.19 (s, 1H).

^{13}C NMR ($CDCl_3$, 100.53 MHz): δ 121.9, 122.9, 123.1, 124.1, 125.1, 127.7, 129.2, 130.8, 134.1, 135.5, 138.7, 140.1.

HRMS (EI): Calcd for $C_{12}H_6Cl_2S$ 251.9567, Found 251.9568.

2,3,8-Trichlorodibenzothiophene (17) [CAS: 153524-15-3].

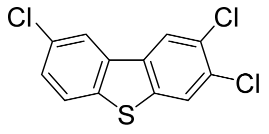

General procedure was followed except that Phenyl(3',4',5-trichloro-[1,1'-biphenyl]-2-yl)sulfane was used instead of [1,1'-biphenyl]-2-yl(phenyl)sulfane.

White solid (46 mg, 54%). Rf 0.57 (hexane).

^{1}H NMR ($CDCl_3$, 399.78 MHz): δ 7.44-7.47 (m, 1H), 7.74 (d, J = 8.2 Hz, 1H), 7.91 (s, 1H), 8.02 (d, J = 1.8 Hz, 1H), 8.14 (s, 1H).

^{13}C NMR ($CDCl_3$, 100.53 MHz): δ 121.8, 123.1, 124.0, 124.2, 128.0, 129.6, 131.5, 131.6, 134.4, 135.4, 138.2, 139.3.

HRMS (EI): Calcd for $C_{12}H_5Cl_3S$ 285.9178, Found 285.9178.

2-Chloro-7-methoxydibenzothiophene (18).

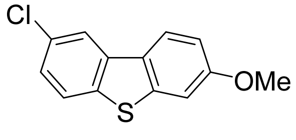

General procedure was followed except that (5-chloro-4'-methoxy-[1,1'-biphenyl]-2-yl)(phenyl)sulfane was used instead of [1,1'-biphenyl]-2-yl(phenyl)sulfane. $Pd(OAc)_2$ (20 mg, 0.090 mmol) and 2,6-dimethylbenzoic acid (40 mg, 0.27 mmol) were used, respectively.

White solid (33 mg, 45%). Rf 0.53 (hexane/EtOAc = 10/1). Mp = 88 °C.

^{1}H NMR ($CDCl_3$, 399.78 MHz): δ 3.91 (s, 3H), 7.05 (dd, J = 8.7 Hz, 2.3 Hz, 1H), 7.30-7.35 (m, 2H), 7.70 (d, J = 8.2 Hz, 1H), 7.95-7.98 (m, 2H).

^{13}C NMR ($CDCl_3$, 100.53 MHz): δ 55.8, 106.0, 113.9, 120.8, 122.6, 123.7, 125.8, 128.2, 130.8, 136.8, 137.0, 142.0, 159.6.

IR(ATR): 2936 w, 2833 w, 1601 s, 1564 w, 1485 s, 1451 s, 1412 m, 1264 s, 1251 s, 1215 s, 1079 m, 1059 m, 1036 m, 1008 w, 880 m, 829 m, 801 s, 772 m.

MS, m/z (relative intensity, %): 248 (M^+, 100), 233 (M^+-15, 28), 205 (M^+-43, 38).

HRMS (EI): Calcd for $C_{13}H_9ClOS$ 248.0063, Found 248.0059.

1-(8-Chlorodibenzo[*b*,*d*]thiophene-3-yl)ethane-1-one (19).

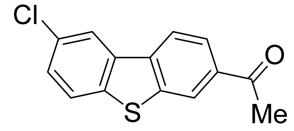

General procedure was followed except that 1-(5’-chloro-2’-(phenylthio)-[1,1’-biphenyl])-4yl)ethane-1-one was used instead of [1,1'-biphenyl]-2-yl(phenyl)sulfane.

White solid (46 mg, 59%). Rf 0.24 (hexane/EtOAc = 10/1). Mp = 155 °C.

^{1}H NMR ($CDCl_3$, 399.78 MHz): δ 2.71 (s, 3H), 7.48 (dd, *J* = 8.5 Hz, 1.8 Hz, 1H), 7.79 (d, *J* = 8.7 Hz, 1H), 8.03-8.06 (m, 1H), 8.13-8.15 (m, 2H), 8.45 (d, *J* = 1.4 Hz, 1H).

^{13}C NMR ($CDCl_3$, 100.53 MHz): δ 27.0, 121.8, 122.4, 123.7, 124.1, 124.7, 128.3, 131.3, 136.0, 136.0, 138.2, 139.3, 140.4, 197.4.

IR(ATR): 1674 s, 1597 w, 1442 w, 1394 m, 1358 m, 1324 w, 1268 s, 1252 s, 1225 w, 1082 m, 1014 w, 974 w, 905 w, 878 w, 822 m, 801 m, 772 m, 670 w.

MS, m/z (relative intensity, %): 260 (M^+, 63), 245 (M^+-15, 100), 217 (M^+-43, 44).

HRMS (EI): Calcd for $C_{14}H_9ClOS$ 260.0063, Found 260.0059.

Ethyl 8-chlorodibenzo[*b,d*]thiophene-3-carboxylate (20).

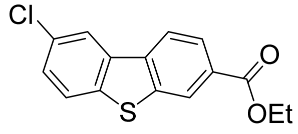

General procedure was followed except that Ethyl 5’-chloro-2’-(phenylthio)-[1,1’-biphenyl]-4-carboxylate was used instead of [1,1'-biphenyl]-2-yl(phenyl)sulfane.

Pale yellow solid (52 mg, 60%). Rf 0.31 (hexane/EtOAc = 20/1). Mp = 138 °C.

^{1}H NMR ($CDCl_3$, 399.78 MHz): δ 1.45 (t, *J* = 7.2 Hz, 3H), 4.44 (q, *J* = 7.2 Hz, 2H), 7.46 (dd, *J* = 8.7 Hz, 1.8 Hz, 1H), 7.78 (d, *J* = 5.4 Hz, 1H), 8.12-8.14 (m, 3H), 8.55 (d, *J* = 0.9 Hz, 1H).

^{13}C NMR ($CDCl_3$, 100.53 MHz): δ 14.5, 61.5, 121.6, 122.4, 124.1, 124.8, 125.8, 128.1, 129.4, 131.2, 136.1, 138.1, 139.1, 140.1, 166.3.

IR(ATR): 2976 w, 1705 s, 1587 w, 1445 w, 1396 w, 1365 w, 1271 m, 1251 m, 1223 w, 1116 w, 1082 m, 1016 w, 869 w, 836 w, 802 w, 770 m, 734 w.
MS, m/z (relative intensity, %): 290 (M^+, 100), 245 (M^+-55, 82), 217 (M^+-73, 39).
HRMS (EI): Calcd for $C_{15}H_{11}ClO_2S$ 290.0168, Found 290.0168.

1-(7-Methoxydibenzo[*b*,*d*]thiophene-2-yl)ethane-1-one (21).

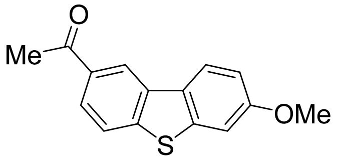

General procedure was followed except that 2-Phenylthio-4’-chlorobiphenyl was used instead of [1,1'-biphenyl]-2-yl(phenyl)sulfane. $Pd(OAc)_2$ (20 mg, 0.090 mmol) and 2,6-dimethylbenzoic acid (40 mg, 0.27 mmol) were used respectively.
Pale yellow solid (39 mg, 51%). Mp = 140 °C. Rf 0.27 (hexane).
^{1}H NMR ($CDCl_3$, 399.78 MHz): δ 2.73 (s, 3H), 3.92 (s, 3H), 7.09-7.10 (m, 1H), 7.34 (d, J = 2.3 Hz, 1H), 7.86 (d, J = 8.2 Hz, 1H), 7.96-7.99 (m, 1H), 8.12 (d, J = 8.7 Hz, 1H), 8.63 (d, J = 1.8 Hz, 1H).
^{13}C NMR ($CDCl_3$, 100.53 MHz): δ 26.9, 55.8, 106.1, 114.0, 120.9, 122.7, 122.7, 125.3, 128.8, 133.9, 135.8, 141.5, 144.0, 159.7, 197.9.
IR(ATR): 3002 w, 2939 w, 2834 w, 1675 s, 1590 s, 1357 m, 1304 m, 1271 s, 1254 s, 1233 s, 1214 s, 1033 s, 961 m, 881 m, 811 m, 753 m.
MS, m/z (relative intensity, %): 256 (M^+, 100), 241 (M^+-15, 95), 213 (M^+-43, 44).
HRMS (EI): Calcd for $C_{15}H_{12}O_2S$ 256.0558, Found 256.0559.

Thieno[2,3-b]benzothiophene (22) [CAS: 247-16-5].

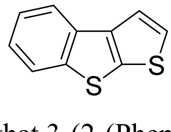

General procedure was followed except that 3-(2-(Phenylthio)phenyl)thiophene was used instead of [1,1'-biphenyl]-2-yl(phenyl)sulfane. $Pd(OAc)_2$ (20 mg, 0.09 mmol) and 2,6-dimethylbenzoic acid (40 mg, 0.27 mmol) were used respectively.
White solid (17 mg, 30%). Rf 0.54 (hexane).
^{1}H NMR ($CDCl_3$, 399.78 MHz): δ 7.32-7.44 (m, 3H), 7.54-7.55 (m, 1H), 7.83 (d, J = 8.0 Hz, 1H), 7.94 (d, J = 7.2 Hz, 1H).
^{13}C NMR ($CDCl_3$, 100.53 MHz): δ 119.4, 121.6, 123.4, 124.4, 124.7, 127.8, 132.9, 137.7, 142.2, 144.2.

HRMS (EI): Calcd for $C_{10}H_6S_2$ 189.9911, Found 189.9912.

Dibenzoselenophene (23) [CAS: 244-95-1].

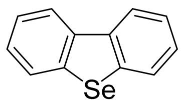

General procedure was followed except that 2-Phenylselenobiphenyl was used instead of [1,1'-biphenyl]-2-yl(phenyl)sulfane. $Pd(OAc)_2$ (20 mg, 0.09 mmol) and 2,6-dimethylbenzoic acid (40 mg, 0.27 mmol) were used respectively.

Pale yellow solid (56 mg, 81%). Rf 0.37 (hexane/EtOAc = 50/1).

^{1}H NMR ($CDCl_3$, 399.78 MHz): δ 7.38-7.49 (m, 4H), 7.90 (d, J = 7.6 Hz, 2H), 8.14 (d, J = 7.6 Hz, 2H).

^{13}C NMR ($CDCl_3$, 100.53 MHz): δ 123.0, 125.0, 126.2, 127.0, 138.4, 139.4.

HRMS (EI): Calcd for $C_{12}H_8Se$ 231.9791, Found 231.9795.

VI. Procedures for Experiments Shown in Scheme 2

The reaction of 1b in the presence of AgOAc.[8] An oven-dried 5 mL screw-capped vial was charged with **1b** (79 mg, 0.30 mmol), $Pd(tfa)_2$ (10.0 mg, 0.03 mmol), AgOAc (200 mg, 1.2 mmol), K_2CO_3 (42 mg, 0.3 mmol) and PivOH (1.0 mL), and the resulting mixture was stirred at rt for 10 min until the generation of CO_2 ceased. The cap was then closed, and the mixture was stirred at 130 °C for 24 h. The mixture was cooled to rt and filtered through a short pad of silica gel, eluting with CH_2Cl_2. The eluent was then evaporated to give a residue, which was purified by flash chromatography (hexane) to give a inseparable mixture of 4-phenyldibenzothiophene **19** and dibenzothiophene **2** (37 mg, 51% in total). The ratio of 19/2 was determined to be 82:18 by GC analysis. Further purification by GPC gave an analytically pure **19**.

4-Phenyldibenzothiophene (24) [CAS: 98251-31-1].

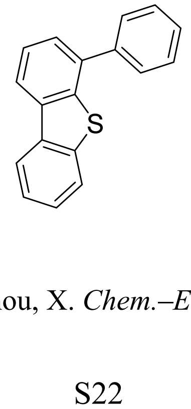

[8] Che, R.; Wu, Z.; Li, Z.; Xiang, H.; Zhou, X. *Chem.–Eur. J.* **2014**, *20*, 7258.

White solid. Mp = 69 °C. Rf 0.34 (hexane).

^{1}H NMR ($CDCl_3$, 399.78 MHz): δ 7.45-7.59 (m, 7H), 7.74-7.76 (m, 2H), 7.83-7.85 (m, 1H), 8.16-8.20 (m, 2H).

^{13}C NMR ($CDCl_3$, 100.53 MHz): δ 120.6, 121.9, 122.8, 124.5, 125.2, 126.9, 127.0, 128.2, 128.4, 128.9, 135.9, 136.3, 137.2, 138.7, 139.7, 140.7.

HRMS (EI): Calcd for $C_{12}H_{12}S$ 260.0660, Found 260.0659.

VII. Experimental Procedures for Scheme 3

(2-Phenylthio)phenylboronic acid (25) [CAS: 515158-87-9].

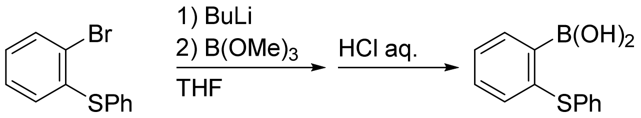

BuLi (1.6 M in hexane, 16.3 mL, 26 mmol) was added in a dropwise manner to a stirred solution of (2-bromophenyl)phenyl sulfide (5.3 g, 20 mmol) in THF (40 mL) at -78 °C, and the resulting mixture was stirred at -78°C for 1 h. $B(OMe)_3$ (2.8 mL, 26 mmol) was then added to the mixture in a dropwise manner at -78°C, and the resulting mixture was warmed to rt and stirred for 2 h. The mixture was acidified with an aqueous solution of HCl (1.0 M), and evaporated to give a residue, which was extracted twice with CH_2Cl_2. The combined organic extracts were dried over $MgSO_4$ and concentrated in vacuo to give a residue, which was triturated with hexane to give (2-phenylthio)phenylboronic acid (**25**) as a white solid (2.2 g, 48%).

Mp = 154 °C.

^{1}H NMR ($CDCl_3$, 399.78 MHz) δ: 6.02 (s, 2H), 7.16-7.21 (m, 3H), 7.24-7.28 (m, 2H), 7.41-7.46 (m, 2H), 7.51-7.54 (m, 1H), 8.09-8.11 (m, 1H).

^{13}C NMR ($CDCl_3$, 100.53 MHz) δ: 126.9, 128.8, 128.9, 129.4, 130.1, 132.1, 136.4, 136.6, 137.4, 138.0.

IR(ATR): 3376 w, 3058 w, 1582 w, 1557 w, 1476 w, 1432 m, 1332 s, 1250 w, 1128 w, 1070 w, 1053 w, 1022 w, 913 w, 738 s, 688 m.

HRMS was measured after converting to the corresponding neopentyl glycol ester.

HRMS (EI): Calcd for $C_{17}H_{19}BO_2S$ 298.1199, Found 298.1205.

2-(4-(tert-Butyl)phenyl)-5-(2'-(phenylthio)-[1,1'-biphenyl]-4-yl)1,3,4-oxadiazole (SM-26).

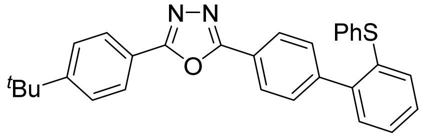

A saturated aqueous solution of Na_2CO_3 (38 mL), $Pd(PPh_3)_4$ (347 mg, 0.3 mmol), and 2-(4-bromophenyl)-5-[4-(1,1-dimethylethyl)phenyl]-1,3,4-oxadiazole[9] (1.8 g, 5.0 mmol) were added to a stirred solution of **25** (690 mg, 3.0 mmol) in toluene (75 mL), and the resulting mixture was refluxed under a nitrogen atmosphere overnight. The reaction mixture was then cooled to rt and partitioned between EtOAc and brine. The separated organic extract was then dried over Na_2SO_4, filtered, and concentrated in vacuo to give a residue, which was purified by flash column chromatography to give **SM-26** as a pale yellow solid (863 mg, 62%).

Mp = 113 °C. Rf = 0.37 (hexane/EtOAc = 5/1).

^{1}H NMR ($CDCl_3$, 399.78 MHz) δ: 1.39 (s, 9H), 7.23-7.35 (m, 9H), 7.57 (dd, *J* = 8.3 Hz, 2.3 Hz, 4H), 8.12 (dd, *J* = 28.8 Hz, 8.2 Hz, 4H).

^{13}C NMR ($CDCl_3$, 150.9 MHz) δ: 31.3, 35.3, 121.4, 123.2, 126.2, 126.7, 127.0, 127.3, 127.4, 128.8, 129.3, 130.3, 130.6, 131.6, 132.3, 134.9, 135.7, 142.4, 144.3, 155.5, 164.5, 164.9.

IR(ATR): 2962 w, 1613 w, 1581 w, 1494 s, 1462 m, 1269 w, 1067 w, 1020 w, 844 m, 749 s, 713 w.

MS, m/z (relative intensity, %): 463 (M^++1, 100).

HRMS (CI): Calcd for $C_{30}H_{26}N_2OS+H^+$ 463.1839, Found 463.1854.

2-(4-(*tert*-Butyl)phenyl)-5-(dibenzothiophene-3-yl)-1,3,4-oxadiazole (26).

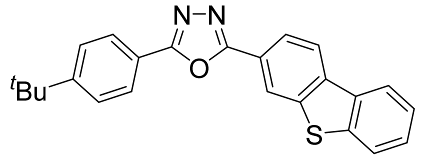

A typical procedure was followed except that **SM-26** was used as the substrate and 0.09 mmol of $Pd(OAc)_2$ and 0.27 mmol of **3** were used.

Pale yellow solid (65 mg, 56%). Mp = 197 °C. Rf 0.43 (hexane/EtOAc = 5/1).

^{1}H NMR ($CDCl_3$, 399.78 MHz): δ 1.39 (s, 9H), 7.51-7.58 (m, 4H), 7.89-7.91 (m, 1H), 8.10 (d, *J* = 8.2 Hz, 2H), 8.21-8.30 (m, 3H), 8.62 (s, 1H).

---

[9] Xu, Q.-L.; Li, H.-Y.; Wang, C.-C.; Zhang, S.; Li, T.-Y.; Jing, Y.-M.; Zheng, Y.-X.; Huang, W.; Zuo, J.-L.; You, X.-Z. *Inorganica Chimica Acta* **2012**, *391*, 50.

^{13}C NMR ($CDCl_3$, 100.53 MHz): δ 31.3, 35.3, 121.2, 121.5, 122.2, 122.2, 122.3, 123.0, 123.1, 124.9, 126.2, 126.9, 127.9, 134.8, 138.2, 140.1, 140.6, 155.6, 164.4, 164.9.

IR(ATR): 2960 m, 1494 w, 1450 w, 1401 w, 1320 w, 839 w, 766 w, 734 m.

MS, m/z (relative intensity, %): 385 (M^++1, 100).

HRMS (CI): Calcd for $C_{24}H_{20}N_2OS+H^+$ 385.1369, Found $C_{24}H_{21}N_2OS$ 385.1370.

2-(2-(Phenylthio)phenyl)anthraquinone (SM-27).

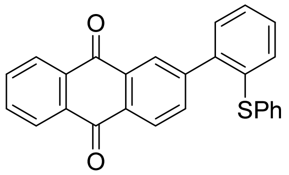

A saturated aqueous solution of Na_2CO_3 (38 mL), $Pd(PPh_3)_4$ (347 mg, 0.3 mmol), 2-bromoanthraquinone (2.15 g, 7.5 mmol) were added to a stirred solution of **25** (690 mg, 3.0 mmol) in toluene (75 mL), and the resulting mixture was refluxed under a nitrogen atmosphere overnight. The reaction mixture was then cooled to rt and partitioned between EtOAc and brine. The separated organic extract was then dried over Na_2SO_4, filtered, and concentrated in vacuo to give a residue, which was purified by flash column chromatography to give **SM-27** as a yellow solid (998 mg, 85%).

Mp = 131 °C. Rf = 0.27 (hexane/EtOAc = 10/1).

^1H NMR ($CDCl_3$, 399.78 MHz) δ: 7.18-7.23 (m, 5H), 7.34-7.38 (m, 4H), 7.80-7.86 (m, 3H), 8.30-8.35 (m, 4H).

^{13}C NMR ($CDCl_3$, 150.9 MHz) δ: 127.1, 127.3, 127.4 (two overlapping peaks), 127.6, 128.3, 129.3, 129.3, 130.5, 131.5, 132.4, 132.5, 133.3, 133.7, 133.7, 134.2, 134.2, 134.8, 135.3, 135.3, 141.6, 146.8, 183.0, 183.1.

IR(ATR): 3057 w, 1739 m, 1674 w, 1577 w, 1440 m, 1379 m, 1323 w, 1280 w, 1231 w, 1101 m, 1016 m, 883 w, 813 m, 740 s, 695 s.

MS, m/z (relative intensity, %): 393 (M^++1, 100).

HRMS (CI): Calcd for $C_{26}H_{16}O_2S$ 392.0871, Found 392.0864.

Anthra[2,3-*b*]benzo[*d*]thiophene-7,12-dione (27) [CAS: 13781-50-5].

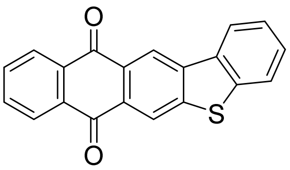

A typical procedure was followed except that **SM-27** was used as the substrate and 0.09 mmol of $Pd(OAc)_2$ and 0.27 mmol of **3** were used.

Yellow solid (84 mg, 89%). Rf 0.27 (hexane/EtOAc = 10/1)
^{1}H NMR ($CDCl_3$, 399.78 MHz): δ 7.55-7.60 (m, 2H), 7.81-7.84 (m, 2H), 7.90-7.92 (m, 1H), 8.34-8.39 (m, 3H), 8.78 (s, 1H), 9.05 (s, 1H).
^{13}C NMR ($CDCl_3$, 150.9 MHz): δ 121.0, 122.5, 123.2, 123.3, 125.6, 127.5 (two overlapping peaks), 128.9, 130.1, 131.3, 134.0, 134.1, 134.3, 134.3, 134.9, 140.0, 141.5, 145.4, 182.9, 183.2.
HRMS (CI): Calcd for $C_{20}H_{10}O_2S+H^+$ 315.0474, Found 315.0484.

Methyl (2'-(Phenylthio)-[1,1'-biphenyl]-4-carbonyl)prolinate (SM-28).

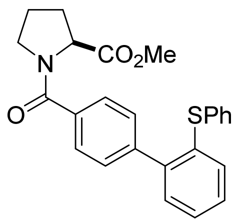

A saturated aqueous solution of Na_2CO_3 (38 mL), $Pd(PPh_3)_4$ (347 mg, 0.3 mmol), methyl (4-bromobenzoyl)-*L*-prolinate (1.87 g, 6.0 mmol) were added to a stirred solution of **25** (690 mg, 3.0 mmol) in toluene (75 mL), and the resulting mixture was refluxed under a nitrogen atmosphere overnight. The reaction mixture was then cooled to rt and partitioned between EtOAc and brine. The separated organic extract was then dried over Na_2SO_4, filtered, and concentrated in vacuo to give a residue, which was purified by flash column chromatography (hexane/EtOAc = 1/1) to give **SM-28** as a yellow oil (1.06 g, 90%).
Rf 0.26 (hexane/EtOAc = 1/1).
The spectroscopic date indicated that **SM-28** existed as a mixture of two rotational isomers.
^{1}H NMR ($CDCl_3$, 399.78 MHz) δ: 1.87-2.06 (m, 4H), 2.29-2.34 (m, 1H), 3.47-3.81 (m, 6H), 4.68 (dd, *J* = 8.4, 5.6 Hz, 1H), 7.18-7.29 (m, 8H), 7.39-7.44 (m, 3H), 7.59 (d, *J* = 7.6 Hz, 2H).
^{13}C NMR ($CDCl_3$, 100.53 MHz) δ: [22.4, 25.0], [29.0, 31.1], [46.2, 49.6], [51.8, 51.9], [58.8, 60.9], [125.8, 126.7], 12.8, 126.8, 128.1, 128.8, [128.8, 129.0], 130.1, 131.1, 131.3, 134.1, 134.6, 135.0, 141.8, 142.1, 168.8, 172.3.
IR(ATR): 3054 w, 2952 w, 2877 w, 1743 s, 1631 s, 1414 s, 1200 m, 1174 m, 1004 w, 848 w, 751 s, 691 m.
MS, m/z (relative intensity, %): 417 (M^+, 25), 289 (M^+-128, 100).
HRMS (CI): Calcd for $C_{25}H_{23}NO_3S$ 417.1399, Found 417.1394.

Methyl (Dibenzothiophene-3-carbonyl)prolinate (28).

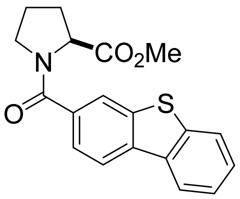

A typical procedure was followed except that **SM-28** was used as the substrate and 0.045 mmol of $Pd(OAc)_2$ and 0.135 mmol of **3** were used.

The spectroscopic date indicated that **28** existed as a mixture of two rotational isomers.
^{1}H NMR ($CDCl_3$, 399.78 MHz) δ: 1.90-2.39 (m, 4H), 3.54-3.85 (m, 5H), 4.73 (dd, *J* = 8.0, 5.2 Hz, 1H), 7.46-7.52 (m, 2H), 7.68 (d, *J* = 8.0 Hz, 1H), 7.86-7.88 (m, 1H), 8.09 (s, 1H), 8.18 (d, *J* = 7.6 Hz, 1H).
^{13}C NMR ($CDCl_3$, 100.53 MHz) δ: [22.8, 25.5], [29.4, 31.5], [46.8, 50.2], 52.4, [59.3, 61.6], [121.4, 121.6], 122.1, 122.1, [122.8, 122.9], 123.7, 124.7, 127.4, 134.4, [134.9, 135.2], [136.6, 137.0], [139.3, 139.4], [140.1, 140.3], [169.3, 170.2], 172.8.
IR(ATR): 3381 w, 2952 w, 2878 w, 1739 m, 1623 m, 1548 w, 1486 w, 1442 m, 1409 m, 1316 w, 1281 w, 1198 m, 1172 m, 1003 w, 834 w, 747 s.
MS, m/z (relative intensity, %): 339 (M^+, 32), 280 (M^+-59, 34), 211 (M^+-128, 100), 183 (M^+-156, 52), 139 (M^+-200, 30).
HRMS (EI): Calcd for $C_{19}H_{17}NO_3S$ 339.0929, Found 339.0923.
The stability of the stereocenter of the proline moiety of **28** under the reaction conditions used for Suzuki-Miyaura and C-H/C-S couplings was confirmed by HPLC (conditions: DAICEL Chiralpak AD, 1.0 mL/min, *n*-hexane/isopropanol = 85/15, at 40 °C).
Racemate:

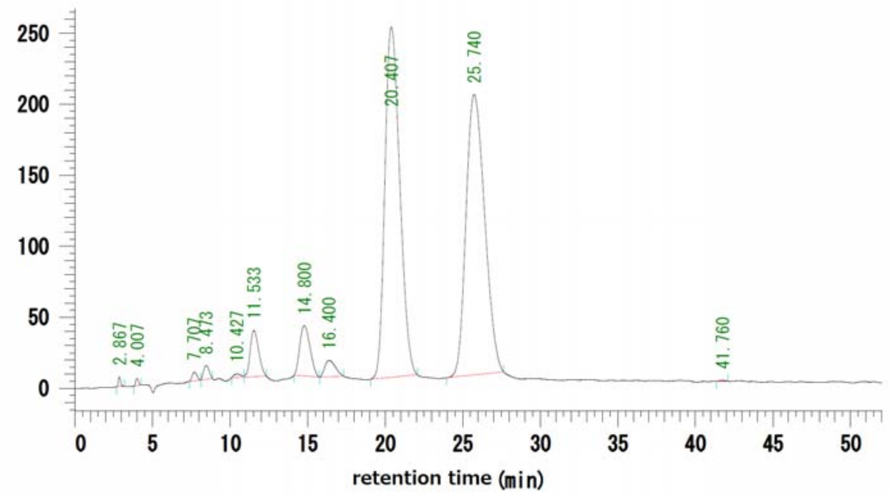

NO	RT	area	conc.	BC
1	2.867	65551	0.179	BB
2	4.007	59094	0.162	BB
3	7.707	127119	0.348	BB
4	8.473	230894	0.632	BB
5	10.427	80794	0.221	BV
6	11.533	1216035	3.327	VB
7	14.800	1604565	4.391	BB
8	16.400	536434	1.468	BB
9	20.407	16279156	44.544	BB
10	25.740	16335077	44.697	BB
11	41.760	11567	0.032	BB
		36546286	100.000	

L-Isomer after being exposed to the conditions of C-H/C-S coupling:

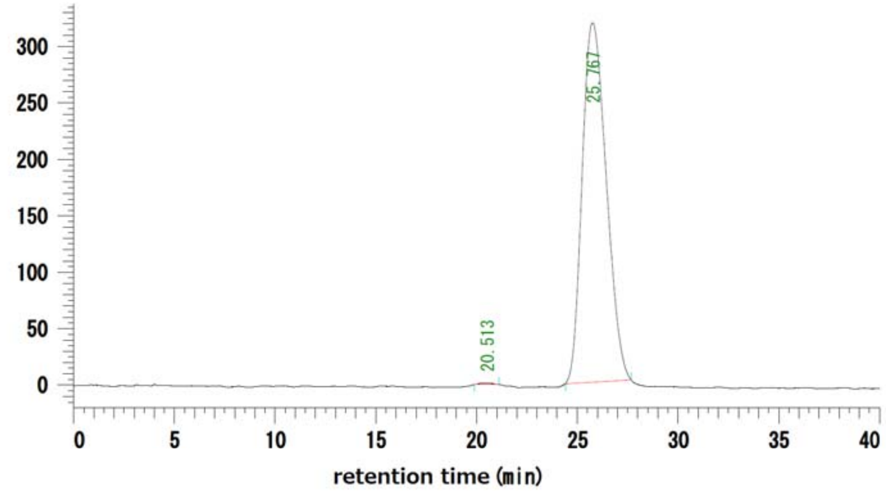

NO	RT	area	conc.	BC
1	20.513	59869	0.227	BB
2	25.767	26308482	99.773	BB
		26368351	100.000	

5,5-Difluoro-1,3,7,9-tetramethyl-10-(2'-phenylthio)-[1,1'-biphenyl]-4-yl) -5*H*-4λ^{4}, 5λ^{4}-dipyrrolo[1,2-*c*:2',1'-*f*][1,3,2]diazaborinine (SM-29).

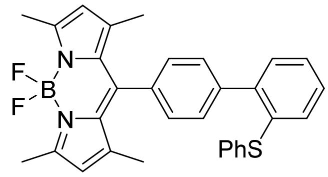

A saturated aqueous solution of Na_2CO_3 (15 mL), $Pd(PPh_3)_4$ (231 mg, 0.2 mmol), 10-(4-bromophenyl)-5,5-difluoro-1,3,7,9-tetramethyl-5*H*-4λ^{4}, 5λ^{4}-dipyrrolo[1,2-*c*:2',1'-*f*][1,3,2]diazaborinine[10] (806 mg, 2.0 mmol) were added to a stirred solution of **25** (920 mg, 4.0 mmol) in toluene (30 mL), and the resulting mixture was refluxed under a nitrogen atmosphere overnight. The reaction mixture was then cooled to rt and partitioned between CH_2Cl_2 and brine. The separated organic extract was then dried over Na_2SO_4, filtered, and concentrated in vacuo to give a residue, which was purified by flash column chromatography (hexane/CH_2Cl_2 = 3/2) to give **SM-29** as a red solid (912 mg, 89%). Rf 0.29 (hexane/CH_2Cl_2 = 3/2).

^{1}H NMR ($CDCl_3$, 399.78 MHz) δ: 1.42 (s, 6H), 2.56 (s, 6H), 598 (s, 2H), 7.19-7.39 (m, 11H), 7.54 (d, *J* = 8.2 Hz, 2H).

^{13}C NMR ($CDCl_3$, 100.53 MHz) δ: 14.7, 14.7, 121.4, 127.0, 127.5, 127.7, 128.7, 129.3, 130.3, 130.7, 130.9, 131.6, 132.3, 134.1, 134.2, 135.7, 141.6, 141.7, 142.8, 143.4, 155.5.

IR(ATR): 2925 w, 1544 s, 1510 m, 1464 m, 1410 w, 1370 w, 1307 m, 1193 s, 1156 s, 1081 m, 1053 m, 979 s, 835 w, 751 m, 714 w.

MS, m/z (relative intensity, %): 508 (M^+, 8), 154 (M^+-354, 100), 136 (M^+-372, 65).

HRMS (FAB): Calcd for $C_{31}H_{27}BF_2N_2S$ 508.1956, Found 508.1977.

10-(Dibenzothiophene-3-yl)-5,5-difluoro-1,3,7,9-tetramethyl-5*H*-4λ^{4}, 5λ^{4}-dipyrrolo[1,2-*c*:2',1'-*f*][1,3,2]diazaborinine (29)

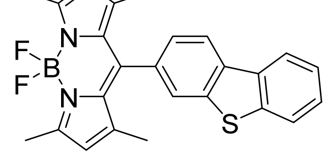

A typical procedure was followed on a 0.1 mmol scale except that **SM-29** was used as the substrate and 0.03 mmol of $Pd(OAc)_2$ and 0.09 mmol of **3** were used. HFIP (0.34 mL) was used instead of toluene.

[10] Zhang, X.; Xiao, Y.; Qian, X. *Org. Lett.*, **2008**, *10*, 29.

Red solid (12.8 mg, 30%). Rf 0.26 (hexane/CH_2Cl_2 = 3/2)

^{1}H NMR ($CDCl_3$, 399.78 MHz): δ 1.36 (s, 6H), 2.58 (s, 6H), 5.99 (s, 2H), 7.37-7.40 (m, 1H), 7.52-7.54 (m, 2H), 7.79 (d, J = 0.92 Hz, 1H), 7.90-7.92 (m, 1H), 8.22-8.24 (m, 1H), 8.29 (d, J = 7.60 Hz, 1H).

^{13}C NMR ($CDCl_3$, 100.53 MHz): δ 14.8 (two overlapping peaks), 121.5, 121.8, 122.0, 122.4, 122.5, 123.1, 124.5, 124.9, 127.5, 131.7, 133.5, 135.1, 136.1, 140.1 (d, J = 26.8 Hz), 141.3, 143.3, 155.8.

IR(ATR): 2925 w, 2856 w, 1545 s, 1510 s, 1469 m, 1409 m, 1369 w, 1308 m, 1193 s, 1157 s, 1082 m, 977 s, 826 w, 751 s, 702 w.

MS, m/z (relative intensity, %): 430 (M^+, 4), 307 (M^+-123, 22), 154 (M^+-276, 100), 136 (M^+-294, 63).

HRMS (FAB): Calcd for $C_{25}H_{21}BF_2N_2S$ 430.1487, Found 430.1495.

VIII. Mechanistic Studies

VIII-1. Intermediacy of a sulfonium intermediate.

Synthesis of 5-phenyl-dibenzothiophenium perchlorate (34) [CAS: 42065-20-3].[11]

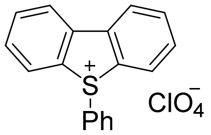

A two-necked flask was charged with dibenzpthiophene 5-oxide (2.0 g, 10.0 mmol) and benzene (20 mL), and the resulting solution was cooled to 0 °C. Conc. H_2SO_4 (2.7 mL) was then added to the mixture, and the resulting mixture was stirred at rt for 24 h. The reaction mixture was added to ice water (50 mL) and extracted with benzene. The aqueous layer was collected, and added 70% $HClO_4$ (5.0 mL). Sparated out solid was collected, which was triturated with MeOH to give 5-phenyl-perchloratedibenzothiophenium (**34**) as a white solid (2.1g, 58%).

^{1}H NMR (DMSO-d_6, 399.78 MHz) δ: 7.59-7.78 (m, 7H), 7.94-7.98 (m, 3H), 8.38 (d, J = 7.80 Hz, 2H), 8.53 (d, J = 7.80 Hz, 2H).

^{13}C NMR (DMSO-d_6, 100.53 MHz) δ: 124.5, 128.3, 129.2, 129.6, 131.2, 131.3, 133.2, 133.9, 134.0, 139.2.

[11] Hori, M.; Kataoka, T.; Shimizu, H.; Miyagaki, M. *Yakugaku Zasshi.* **1973**, *93*, 476.

HRMS (FAB): Calcd for $C_{18}H_{13}S$ $[M-ClO_4]^+$ 261.0732, Found 261.0739.

A procedure for the reaction of 34 with stoichiometric Pd(0) complexes.

An oven-dried 5 mL screw-capped vial was charged with a palladium complex (0.15 mmol), 5-phenyl-perchloratedibenzothiophenium (**34**) (54.1 mg, 0.15 mmol) and toluene (0.5 mL) under a gentle stream of nitrogen, and the resulting mixture was heated at the indicated temperature for 18 h. The mixture was cooled to rt and filtered through a short pad of silica gel, eluting with EtOAc. The eluent was evaporated to give a residue, which was purified by flash column chromatography over silica gel eluting with hexane. In some cases, the yield was too low to allow for the isolation of the product, and the yield was consequently determined by NMR using 1,1,2,2-tetrachloroethane as an internal standard. When $Pd(PPh_3)_4$ was used as the palladium(0) source, dibenzothiophene (**2**) was obtained in good yield, even at 80 °C (entries 1). A similar result was also obtained with a phosphine-free palladium(0) source $[CpPd(\eta^3\text{-}1\text{-}PhC_3H_4)]$, although a higher temperature (130 °C) was required for completion (entries 2 and 3). These results indicated that the sulfonium salt **31** in Scheme 4 was involved as a potential intermediate in the palladium-catalyzed C-H/C-S coupling reaction. Sulfonium salt **34** did not give **2** in the absence of a palladium complex (entry 4), which indicates that the C-S bond cleavage is mediated by a palladium complex.

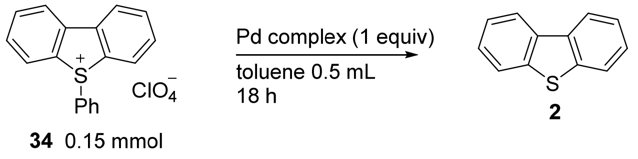

entry	Pd complex	temp. (°C)	NMR yield of **2**
1	$Pd(PPh_3)_4$	80	94%
2	$[CpPd(\eta^3\text{-}1\text{-}PhC_3H_4)]$	80	12%
3	$[CpPd(\eta^3\text{-}1\text{-}PhC_3H_4)]$	130	87%
4	none	130	0%

VIII-2. The fate of the cleaved phenyl group.

An oven-dried 5 mL screw-capped vial was charged with $Pd(OAc)_2$ (10.1 mg, 0.045 mmol), 2-phenylthio-4'-acetylbiphenyl (**SM-5**, 91.3 mg, 0.30 mmol), 2,6-dimethylbenzoic acid (**3**, 20.3 mg, 0.135 mmol) and toluene (1 mL) under a gentle stream of nitrogen, and the resulting mixture was heated at 130 °C for 18 h. The mixture was cooled to rt and filtered through a short pad of silica gel, eluting with EtOAc. The eluent was analyzed by GC using 4-benzylbiphenyl

as an internal standard. The results of this analysis revealed that benzene was formed in 73% yield, along with the cyclized product (94% by NMR).

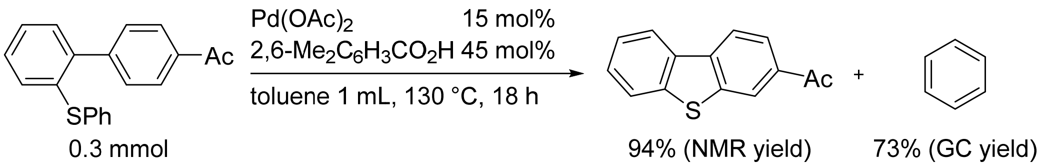

VIII-3. Labeling studies.

An oven-dried 10 mL two necked flask was charged with $Pd(OAc)_2$ (6.7 mg, 0.03 mmol), **1b** (78.7 mg, 0.30 mmol), 2,6-dimethylbenzoic acid (13.5 mg, 0.09 mmol), 4-benzylbiphenyl (25 mg as an internal standard) and toluene (2 mL) under a gentle stream of nitrogen, and the resulting mixture was refluxed under a nitrogen atmosphere. The reaction was sampled after 30, 60, 90, 120, 240, and 480 min. Each sample was diluted with EtOAc and analyzed by GC. The same reaction was also conducted in parallel using **1b-*d*$_5$** (80.2 mg, 0.3 mmol) instead of **1b**.

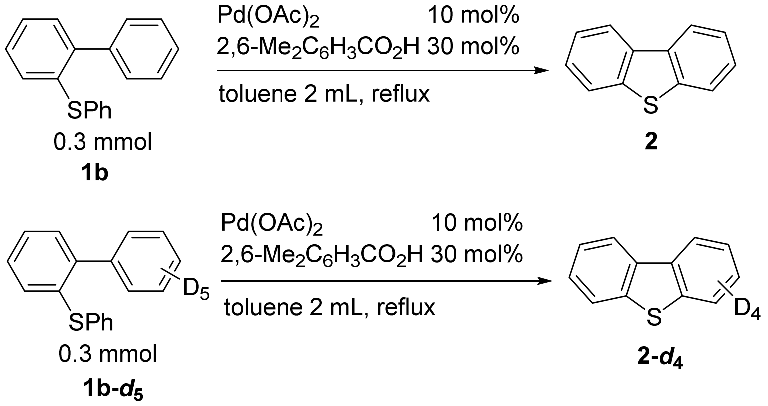

The amount of **2** or **2-*d*$_4$** in each reaction mixture was determined over time, and the results are shown below. The profiles for the reactions of **1b** and **1b-*d*$_4$** were similar, indicating that C-H bond cleavage was not involved in the turnover-limiting step of the catalytic cycle.

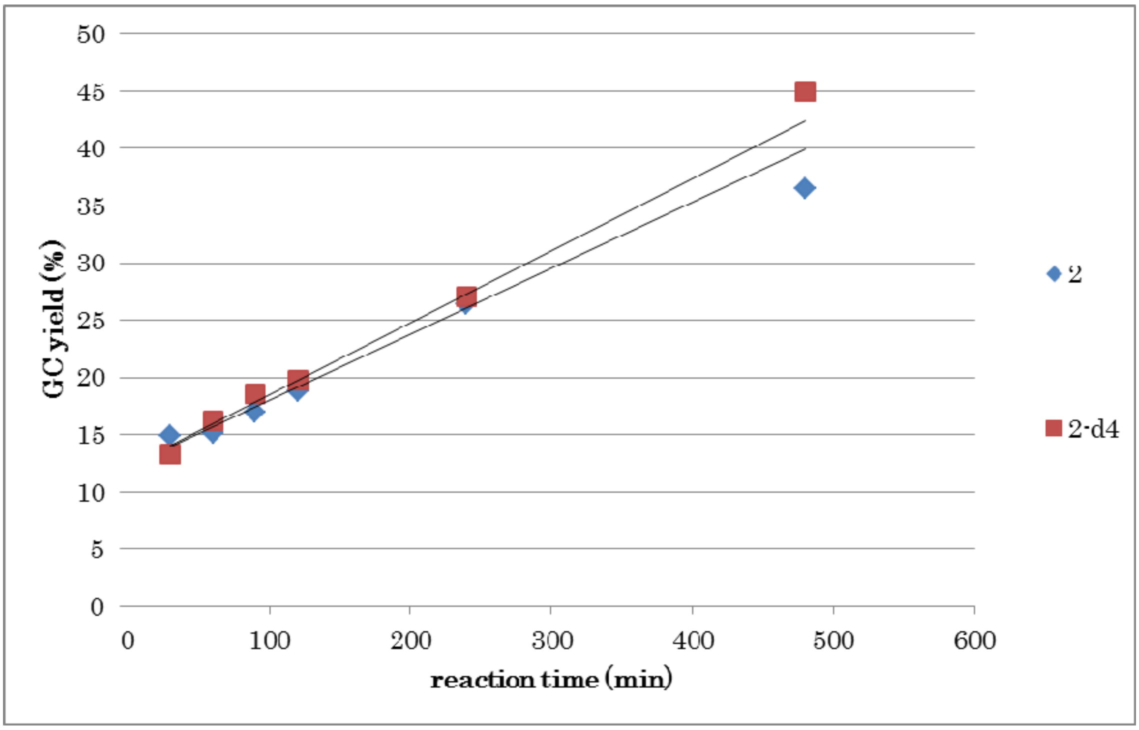

([1,1'-Biphenyl]-2-yl-2',3',4',5',6',-d_5)(phenyl)sulfane (1b-d_5).

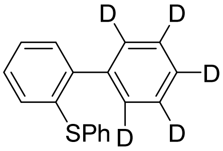

A saturated aqueous solution of Na_2CO_3 (38 mL), $Pd(PPh_3)_4$ (578 mg, 0.50 mmol), and bromobenzene-d_5 (2.43 g, 15.0 mmol) were added to a stirred solution of (2-phenylthio)phenyl boronic acid (1.15 g, 5.0 mmol) in toluene (75 mL), and the resulting mixture was then refluxed under nitrogen atmosphere overnight. The reaction mixture was then cooled to rt and partitioned between EtOAc and brine. The organic layer was then dried over Na_2SO_4, filtered, and concentrated in vacuo to give a residue, which was purified by flash column chromatography (hexane, Rf 0.24) to give **1b-d_5** (1.18 g, 88%) as a colorless oil.

^{1}H NMR ($CDCl_3$, 399.78 MHz) δ: 7.19-7.32 (m, 9H).

^{13}C NMR ($CDCl_3$, 100.53 MHz) δ: 126.8, 127.1, 127.4, 128.0, 128.1, 129.2, 129.4, 130.6, 131.2, 131.9, 135.0, 135.6, 140.4, 143.0.

IR(ATR): 3056 w, 1581 w, 1472 m, 1437 w, 1383 w, 1321 w, 1067 w, 1024 w, 834 w, 745 s, 690 s.

MS, m/z (relative intensity, %): 267 (M^+, 100), 190 (M^+-77, 14), 157 (M^+-110, 8).

HRMS (EI): Calcd for $C_{18}H_9D_5S$ 267.1130, Found 267.1129.

Dibenzothiophene-1,2,3,4-*d*$_4$ (2-*d*$_4$).

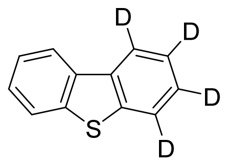

General procedure was followed except that **1b-*d*$_5$** was used instead of [1,1'-biphenyl]-2-yl(phenyl)sulfane.

White solid (28 mg, 50%). Mp = 94 °C. Rf 0.46 (hexane).

^{1}H NMR ($CDCl_3$, 399.78 MHz) δ: 7.45-7.47 (m, 2H), 7.85-7.87 (m, 1H), 8.15-8.18 (m, 1H).

^{13}C NMR ($CDCl_3$, 100.53 MHz) δ: 121.7, 122.9, 124.5, 126.8, 135.7, 139.6.

IR(ATR): 3056 w, 2924 w, 1739 w, 1563 w, 1442 w, 1375 w, 1334 w, 1309 w, 1261 w, 1224 w, 1130 w, 1069 w, 1049 w, 1024 w, 733 s.

MS, m/z (relative intensity, %): 188 (M^+, 100).

HRMS (CI): Calcd for $C_{12}H_4D_4S$ 188.0598, Found 188.0600.

VIII-4. Product inhibition.

An oven-dried 5 mL screw-capped vial was charged with $Pd(OAc)_2$ (10.1 mg, 0.045 mmol), **1b** (78.7 mg, 0.30 mmol) or 2-phenylthio-4'-acetylbiphenyl **SM-5** (91.3 mg, 0.30 mmol), 2,6-dimethylbenzoic acid (20.3 mg, 0.135 mmol), DBT derivative (0.30 mmol) and toluene (1 mL) under a gentle stream of nitrogen, and the resulting mixture was heated at 130 °C for 18 h. The mixture was cooled to rt and filtered through a short pad of silica gel, eluting with EtOAc. The filtrate was evaporated. The yield of **5** were determined by NMR using 1,1,2,2-tetrachloroethane as an internal standard. As shown below, the yields of the product decreased by 28-48 % following the addition of DBT derivatives. These results indicated that the magnitude of the inhibitory effect of the dibenzothiophene derivative was dependent on its structure, with the electron-rich derivative showing higher inhibition than the electron-neutral derivative.

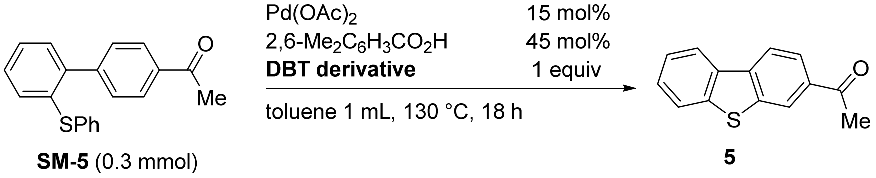

entry	**DBT derivative**	NMR yield
1	none	98%
2	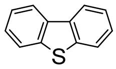	68%
3	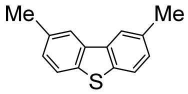	50%

VIII-5. Effect of the leaving group.

Several other SAr groups were also investigated in this reaction to determine the electronic effects on the outcome of the catalytic cyclization process. The results revealed that the use of an electron-rich phenyl ring (i.e., Ar = *p*-MeO-C_6H_4) resulted in a similar yield of the cyclized product to that obtained using a naked phenyl ring (i.e., Ar = Ph). However, the use of an electron-deficient phenyl ring (i.e., Ar = *p*-F_3C-C_6H_4) led to a 6-fold decrease in the yield (see the table below). These electronic effects can be attributed to a delicate balance between the demands of the different steps involved in the ring-forming process. For example, the use of an electron-rich SAr group would be preferred during the reductive elimination step from **30'**, whereas an electron-deficient SAr group would better facilitate the subsequent oxidative addition step of **31'**. Based on these considerations, it is clear to see why the use of an electron-neutral SPh group gave the highest yield of the product.

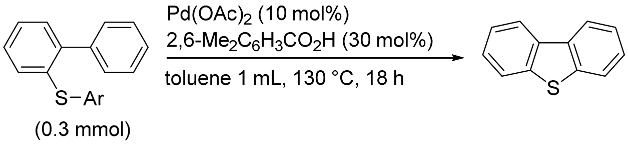

entry	Ar	NMR yield
1	Ph	66%
2	*p*-OMe-C_6H_4	54%
3	*p*-CF_3-C_6H_4	10%

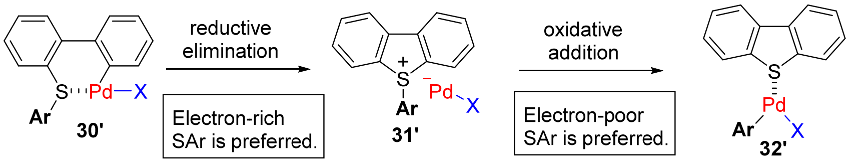

General procedure for the synthesis of diaryl sulfide with aryl thiols.[12]

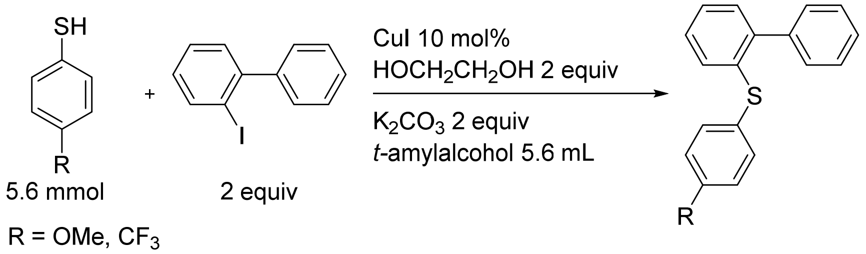

Cu(I) iodide (213 mg, 1.12 mmol), K_2CO_3 (1.5 g, 11.2 mmol), ethylene glycol (624 µL, 11.2 mmol), 2-iodobiphenyl (3.14 g, 11.2 mmol), 4-methoxybenzenethiol (785 mg, 5.6 mmol) and *t*-amylalcohol (5.6 mL) were added to a 50 mL Schlenk flask under a nitrogen atmosphere. The flask was heated at 100 °C and stirred for 24 h. The mixture was cooled to rt and filtered through a short pad of silica gel, eluting with EtOAc. The eluent was evaporated, and the residue was purified by flash chromatography (hexane/EtOAc = 10/1, Rf 0.50) to give [1,1'-biphenyl]-2-yl(4-methoxyphenyl)sulfane as a white solid (442 mg, 27%).

[1,1'-Biphenyl]-2-yl(4-methoxyphenyl)sulfane .

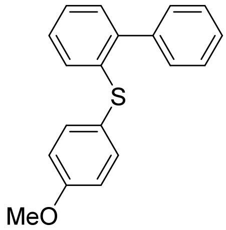

Mp = 93 °C.

^{1}H NMR ($CDCl_3$, 399.78 MHz) δ: 3.81 (s, 3H), 6.87 (dd, *J* = 6.3 Hz, 2.9 Hz, 2H), 6.93-6.96 (m, 1H), 7.16-7.26 (m, 3H), 7.32-7.46 (m, 7H).

^{13}C NMR ($CDCl_3$, 100.53 MHz) δ: 55.5, 115.1, 124.3, 125.6, 127.6, 128.0, 128.2, 128.2, 129.5, 130.3, 136.1, 137.8, 140.7, 141.0, 160.0.

IR(ATR): 3055 w, 2937 w, 2835 w, 1590 w, 1491 m, 1460 m, 1286 w, 1245 s, 1173 w, 1031 w, 1008 w, 913 w, 827 w, 746 s, 700 s.

MS, m/z (relative intensity, %): 292 (M^+, 100), 244 (M^+-48, 24), 216 (M^+-76, 11).

HRMS (CI): Calcd for $C_{19}H_{16}OS$ 292.0922, Found 292.0922.

[1,1'-Biphenyl]-2-yl(4-(trifluoromethyl)phenyl)sulfane .

---

[12] Kwong, F. Y.; Buchwald, S. L. *Org. Lett.* **2002**, *4*, 3517.

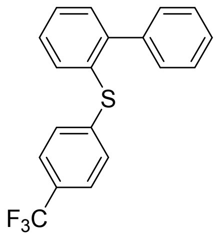

General procedure was followed except that 4-trifluoromethylbenzenethiol was used instead of 4-methoxybenzenethiol

Colorless oil. (668 mg, 36%). Rf 0.34 (hexane).

^{1}H NMR ($CDCl_3$, 399.78 MHz) δ: 7.16 (d, *J* = 8.0 Hz, 2H), 7.30-7.36 (m, 6H), 7.39-7.42 (m, 4H), 7.47-7.49 (m, 1H).

^{13}C NMR ($CDCl_3$, 100.53 MHz) δ: 124.2 (d, *J* = 271.2 Hz), 125.8 (dd, *J* = 6.7 Hz, 3.8 Hz), 127.6, 128.0, 128.1 (d, *J* = 32.6 Hz), 128.6, 128.8, 129.0, 129.4, 131.2, 131.7, 134.5, 140.6, 142.9, 145.5.

IR(ATR):3059 w, 1604 w, 1463 w, 1401 w, 1324 s, 1164 m, 1120 s, 1092 m, 1063 m, 1038 w, 1012 m, 827 w, 772 w, 750 s, 699 m.

MS, m/z (relative intensity, %): 330 (M^+, 100), 184 (M^+-146, 25), 152 (M^+-178, 18).

HRMS (CI): Calcd for $C_{19}H_{16}OS$ 330.0690, Found 330.0687.

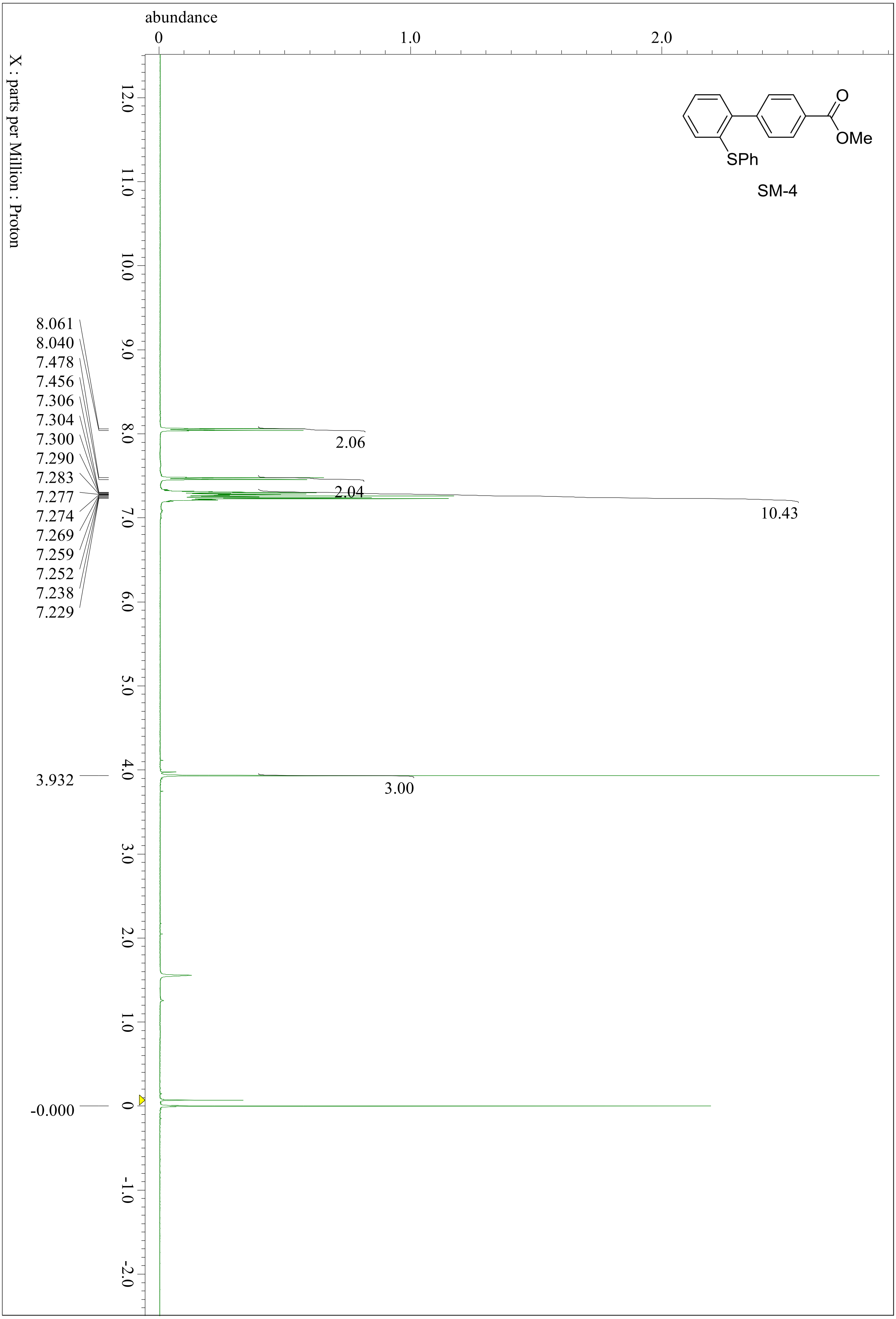

abundance
0
1.0
2.0
X : parts per Million : Proton
12.0
11.0
10.0
9.0
8.0
7.0
6.0
5.0
4.0
3.0
2.0
1.0
0
-1.0
-2.0
8.061
8.040
7.478
7.456
7.306
7.304
7.300
7.290
7.283
7.277
7.274
7.269
7.259
7.252
7.238
7.229
3.932
-0.000
2.06
2.04
10.43
3.00
O
OMe
SPh
SM-4

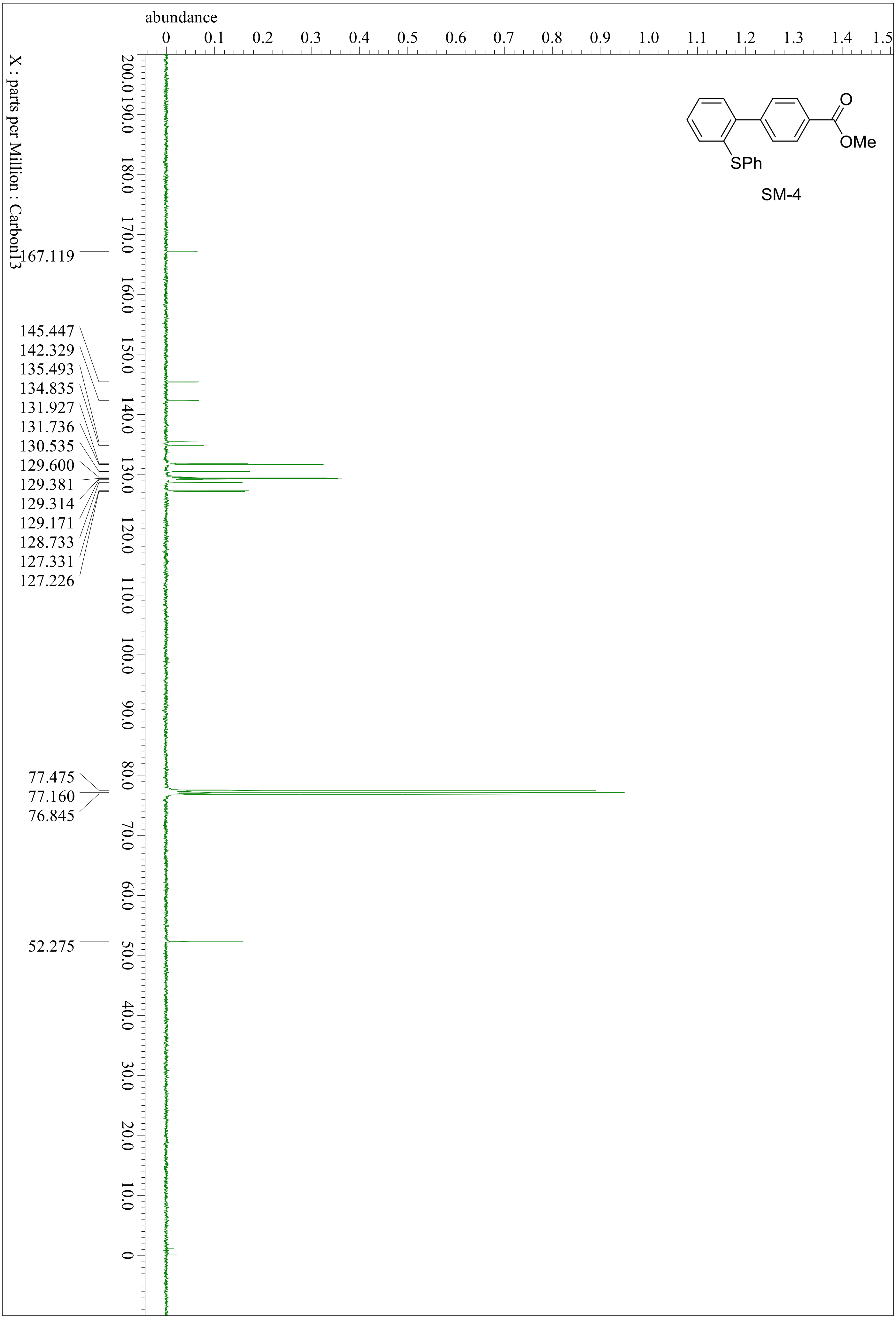

abundance
0 0.1 0.2 0.3 0.4 0.5 0.6 0.7 0.8 0.9 1.0 1.1 1.2 1.3 1.4 1.5
X : parts per Million : Carbon13
200.0 190.0 180.0 170.0 160.0 150.0 140.0 130.0 120.0 110.0 100.0 90.0 80.0 70.0 60.0 50.0 40.0 30.0 20.0 10.0 0
167.119
145.447
142.329
135.493
134.835
131.927
131.736
130.535
129.600
129.381
129.314
129.171
128.733
127.331
127.226
77.475
77.160
76.845
52.275
O
OMe
SPh
SM-4

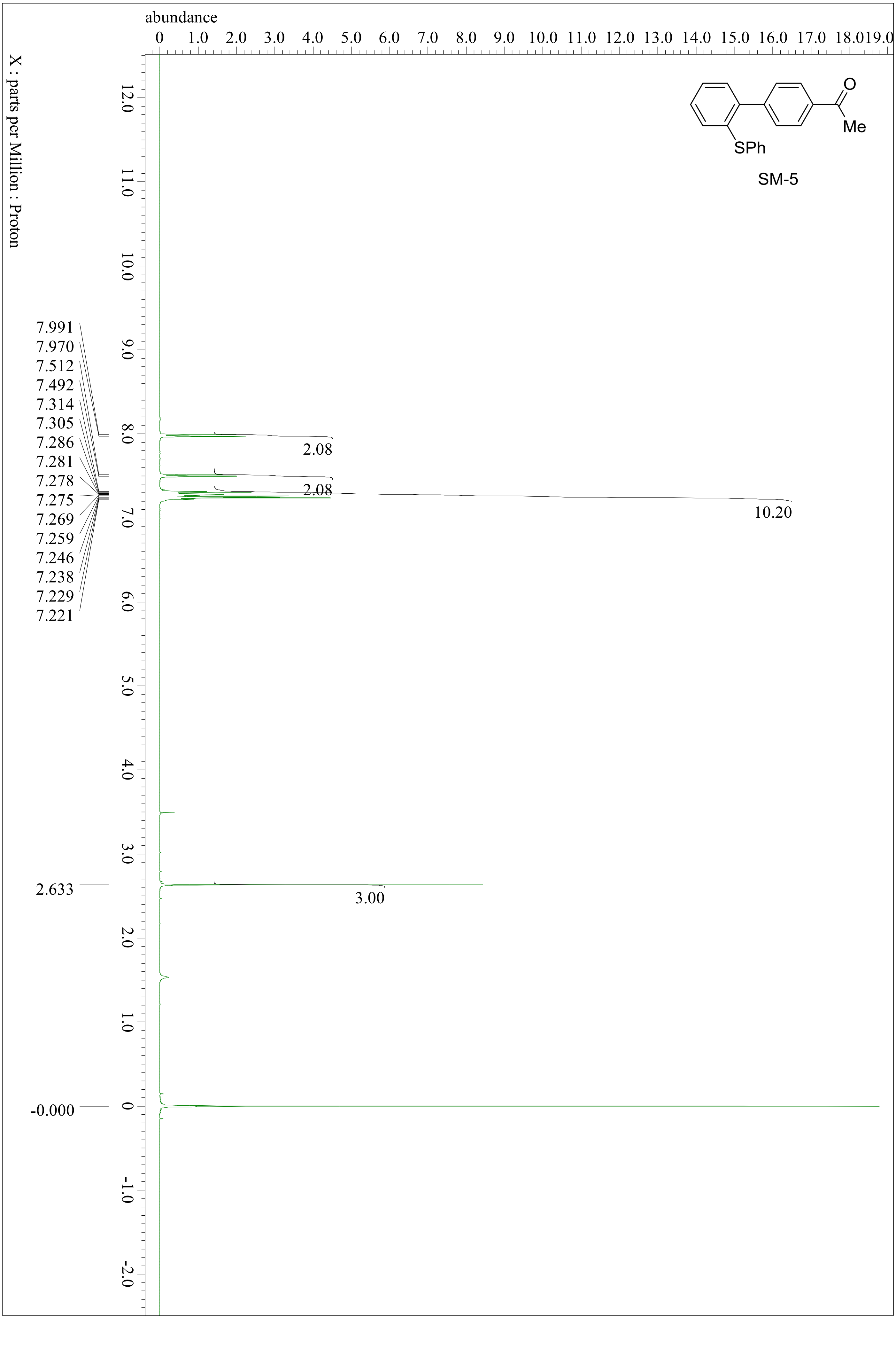
SM-5
SPh
Me
O
X : parts per Million : Proton
abundance
7.991
7.970
7.512
7.492
7.314
7.305
7.286
7.281
7.278
7.275
7.269
7.259
7.246
7.238
7.229
7.221
2.633
-0.000
2.08
2.08
10.20
3.00

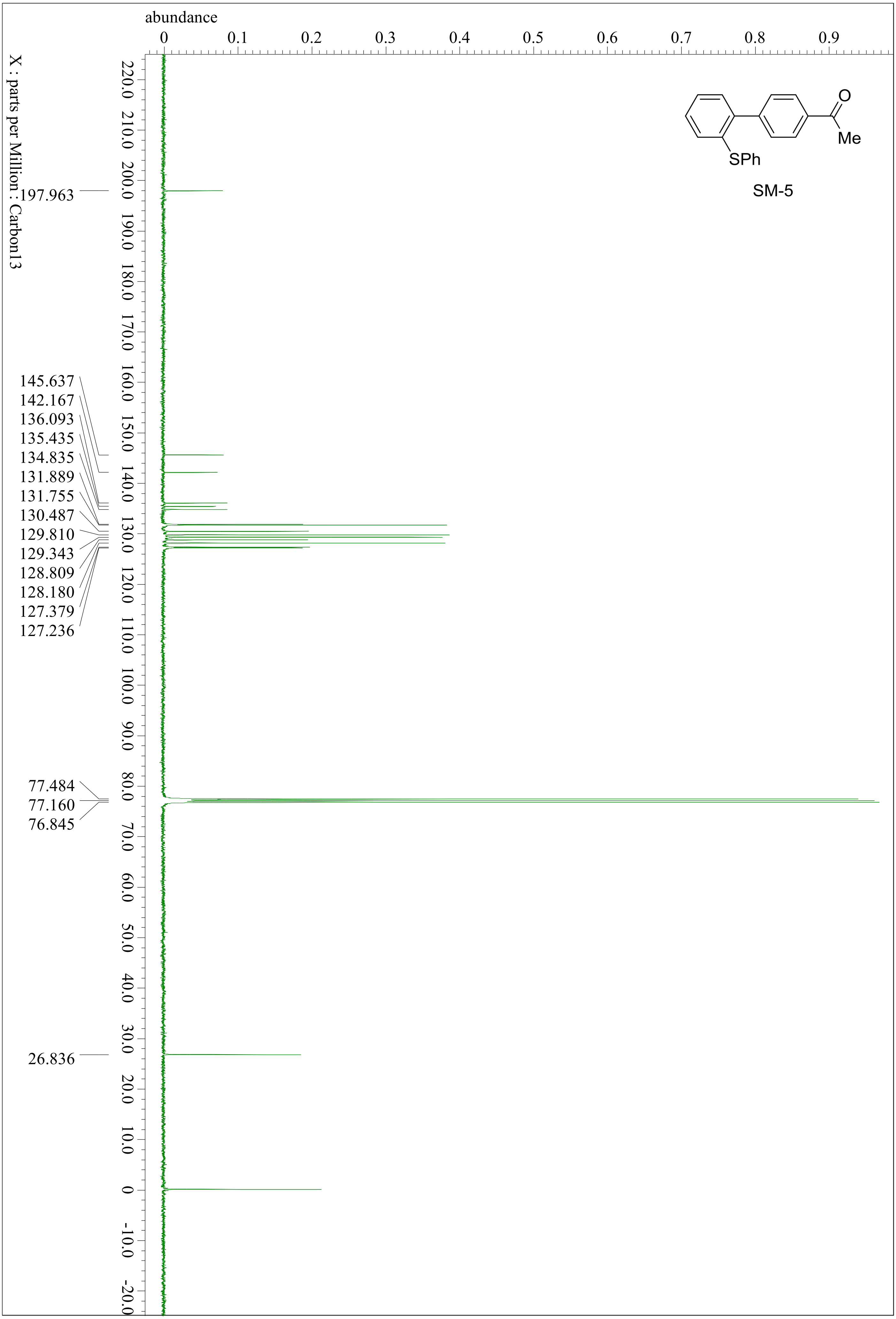
abundance
0 0.1 0.2 0.3 0.4 0.5 0.6 0.7 0.8 0.9
X : parts per Million : Carbon13
220.0 210.0 200.0 190.0 180.0 170.0 160.0 150.0 140.0 130.0 120.0 110.0 100.0 90.0 80.0 70.0 60.0 50.0 40.0 30.0 20.0 10.0 0 -10.0 -20.0
197.963
145.637
142.167
136.093
135.435
134.835
131.889
131.755
130.487
129.810
129.343
128.809
128.180
127.379
127.236
77.484
77.160
76.845
26.836
O
Me
SPh
SM-5

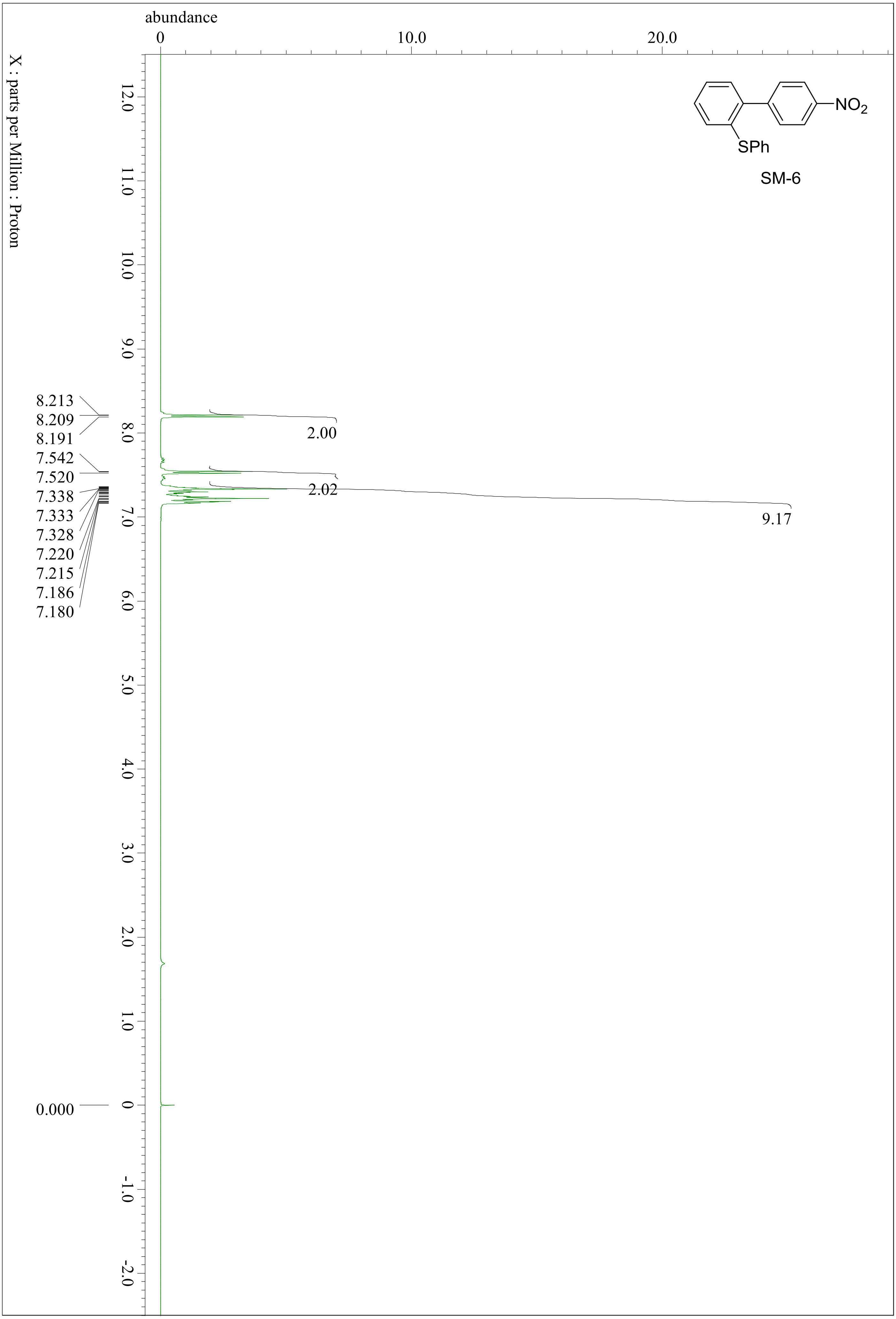

abundance
0
10.0
20.0
X : parts per Million : Proton
12.0
11.0
10.0
9.0
8.0
7.0
6.0
5.0
4.0
3.0
2.0
1.0
0
-1.0
-2.0
8.213
8.209
8.191
7.542
7.520
7.338
7.333
7.328
7.220
7.215
7.186
7.180
0.000
2.00
2.02
9.17
NO2
SPh
SM-6

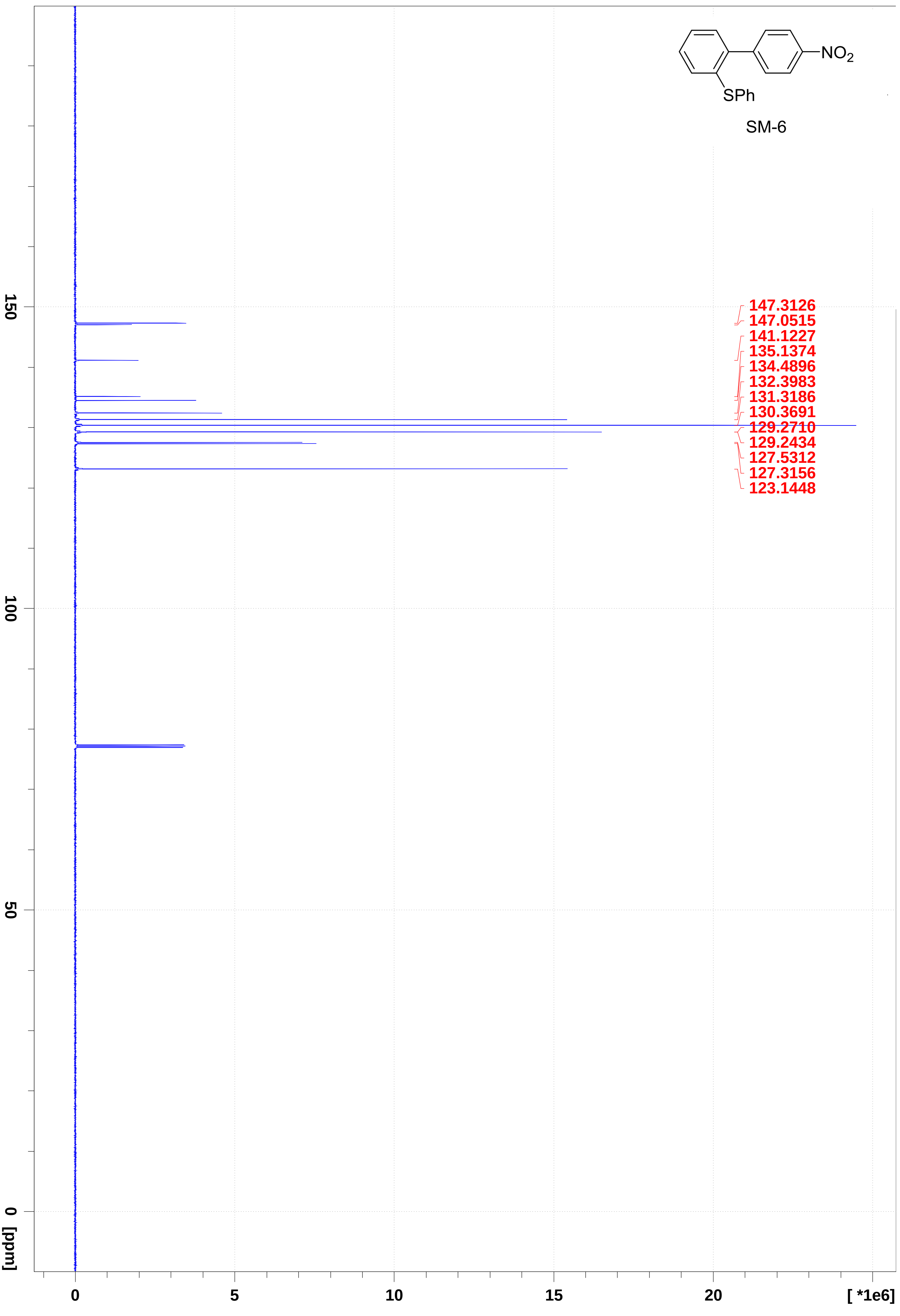
NO2
SPh
SM-6
147.3126
147.0515
141.1227
135.1374
134.4896
132.3983
131.3186
130.3691
129.2710
129.2434
127.5312
127.3156
123.1448
150
100
50
0
[ppm]
0
5
10
15
20
[*1e6]

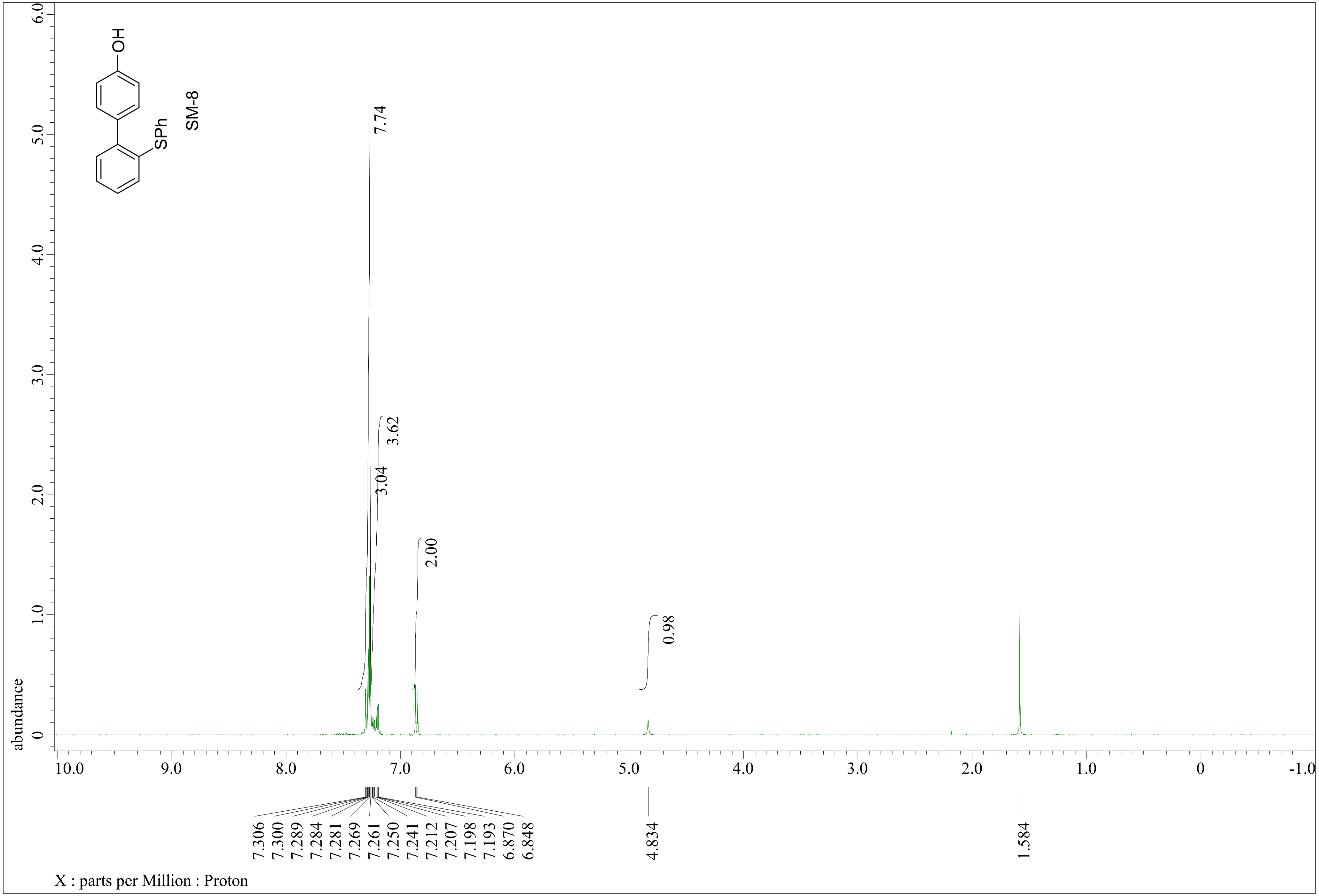

OH
SPh
SM-8
abundance
6.0
5.0
4.0
3.0
2.0
1.0
0
7.74
3.62
3.04
2.00
0.98
10.0
9.0
8.0
7.0
6.0
5.0
4.0
3.0
2.0
1.0
0
-1.0
7.306
7.300
7.289
7.284
7.281
7.269
7.261
7.250
7.241
7.212
7.207
7.198
7.193
6.870
6.848
4.834
1.584
X : parts per Million : Proton

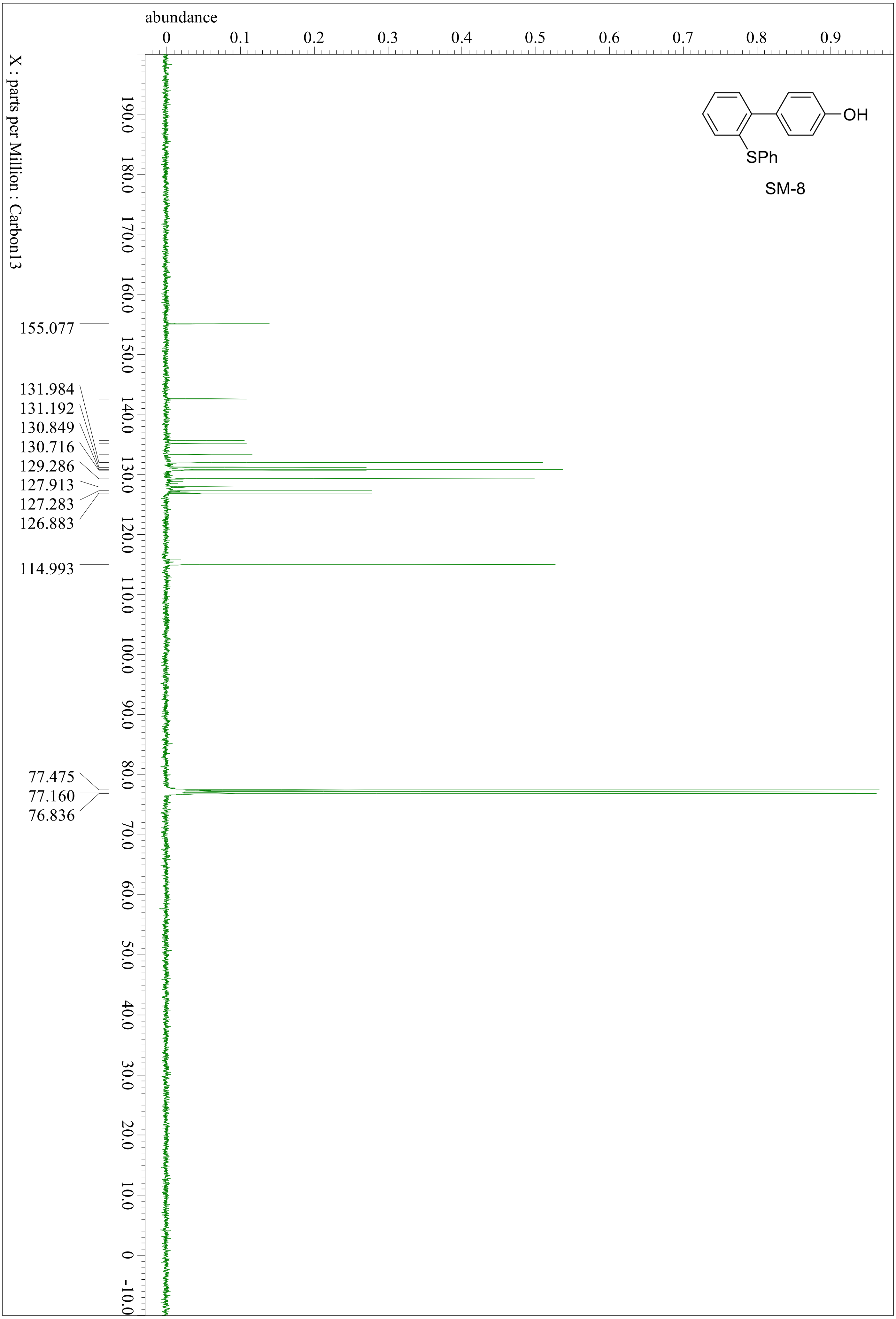

abundance
0
0.1
0.2
0.3
0.4
0.5
0.6
0.7
0.8
0.9
X : parts per Million : Carbon13
190.0
180.0
170.0
160.0
150.0
140.0
130.0
120.0
110.0
100.0
90.0
80.0
70.0
60.0
50.0
40.0
30.0
20.0
10.0
0
-10.0
155.077
131.984
131.192
130.849
130.716
129.286
127.913
127.283
126.883
114.993
77.475
77.160
76.836
OH
SPh
SM-8

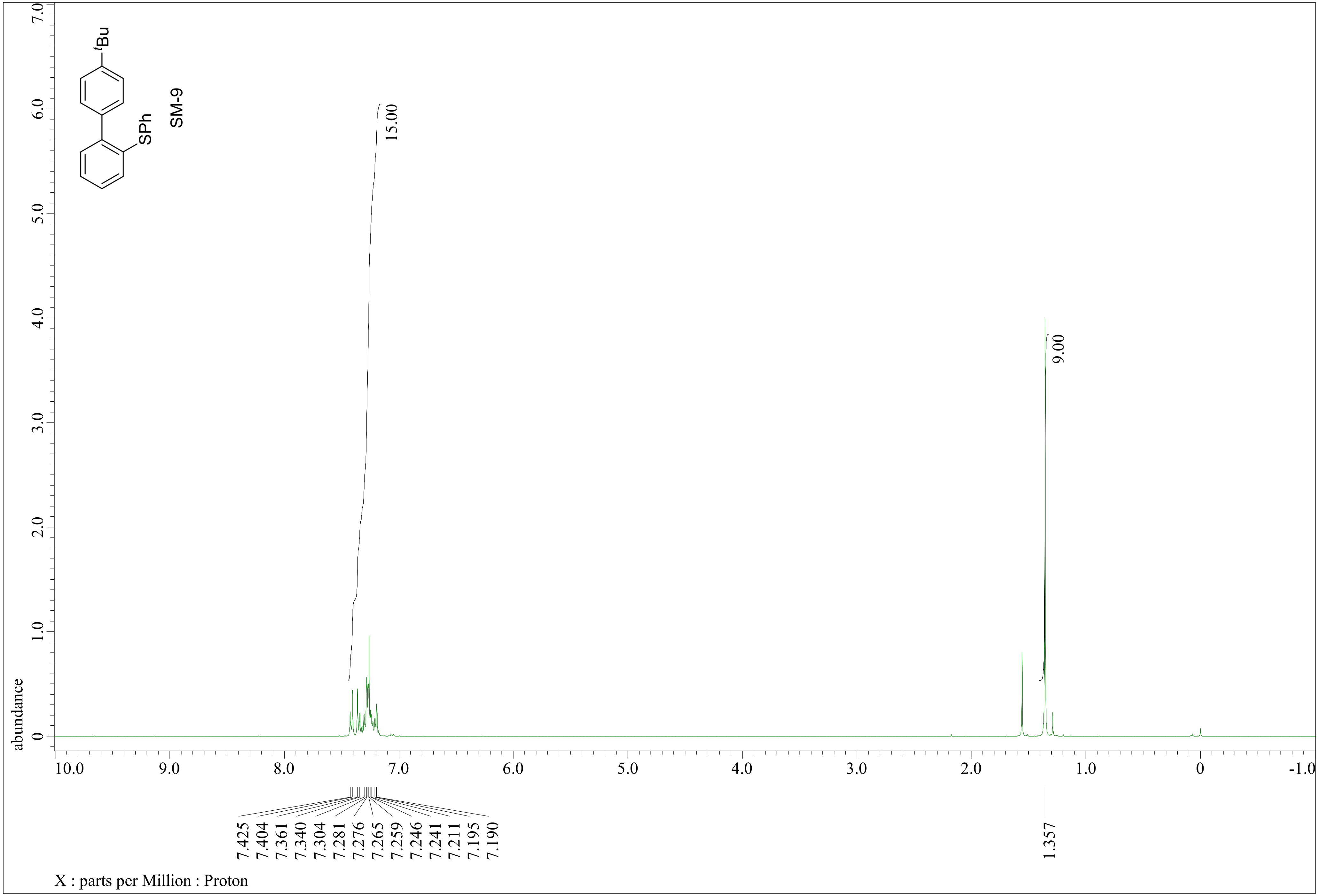

SPh
tBu
SM-9
abundance
0
1.0
2.0
3.0
4.0
5.0
6.0
7.0
15.00
9.00
10.0
9.0
8.0
7.0
6.0
5.0
4.0
3.0
2.0
1.0
0
-1.0
7.425
7.404
7.361
7.340
7.304
7.281
7.276
7.265
7.259
7.246
7.241
7.211
7.195
7.190
1.357
X : parts per Million : Proton

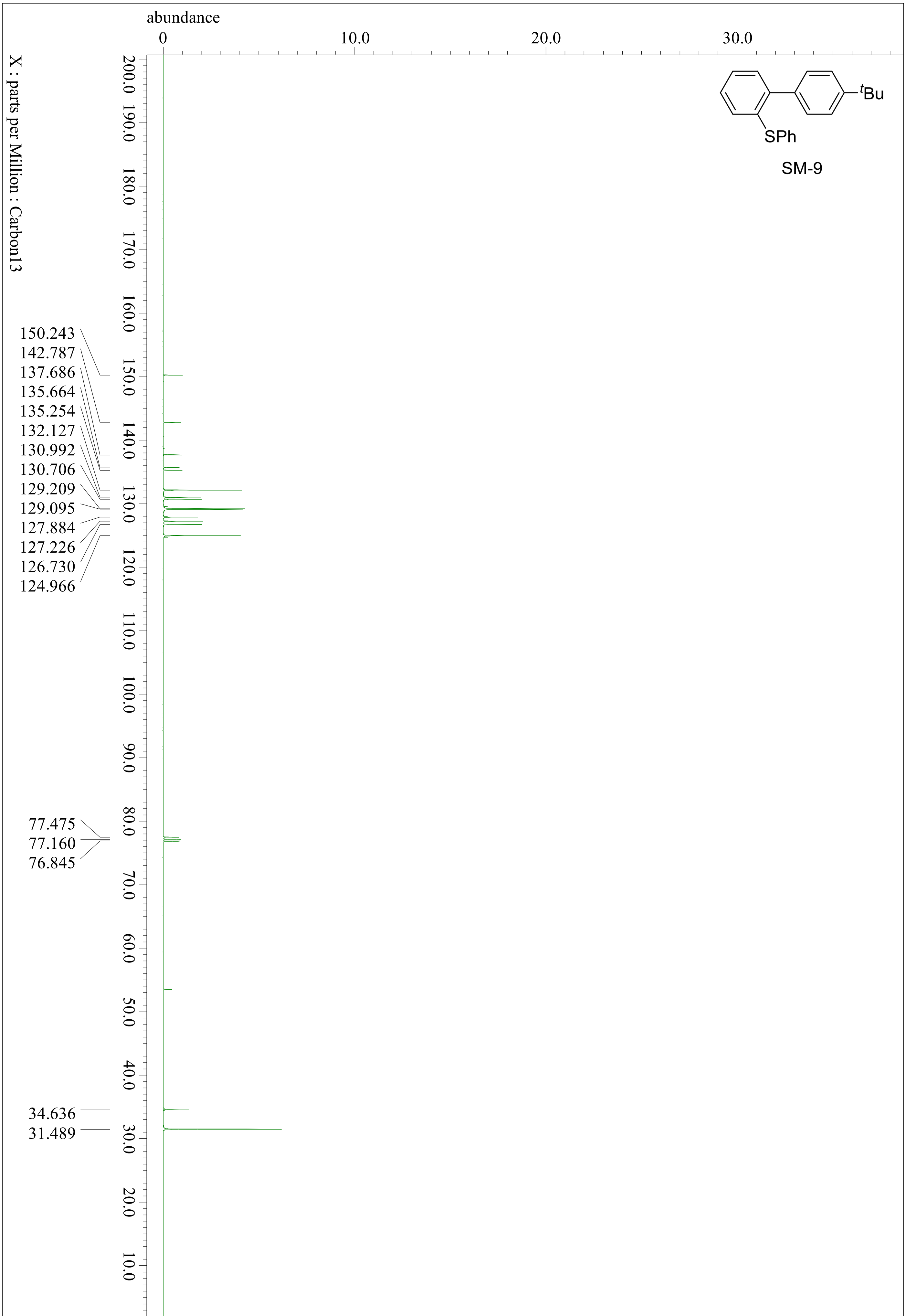
abundance
0
10.0
20.0
30.0
X : parts per Million : Carbon13
200.0
190.0
180.0
170.0
160.0
150.0
140.0
130.0
120.0
110.0
100.0
90.0
80.0
70.0
60.0
50.0
40.0
30.0
20.0
10.0
0
150.243
142.787
137.686
135.664
135.254
132.127
130.992
130.706
129.209
129.095
127.884
127.226
126.730
124.966
77.475
77.160
76.845
34.636
31.489
SPh
^{t}Bu
SM-9

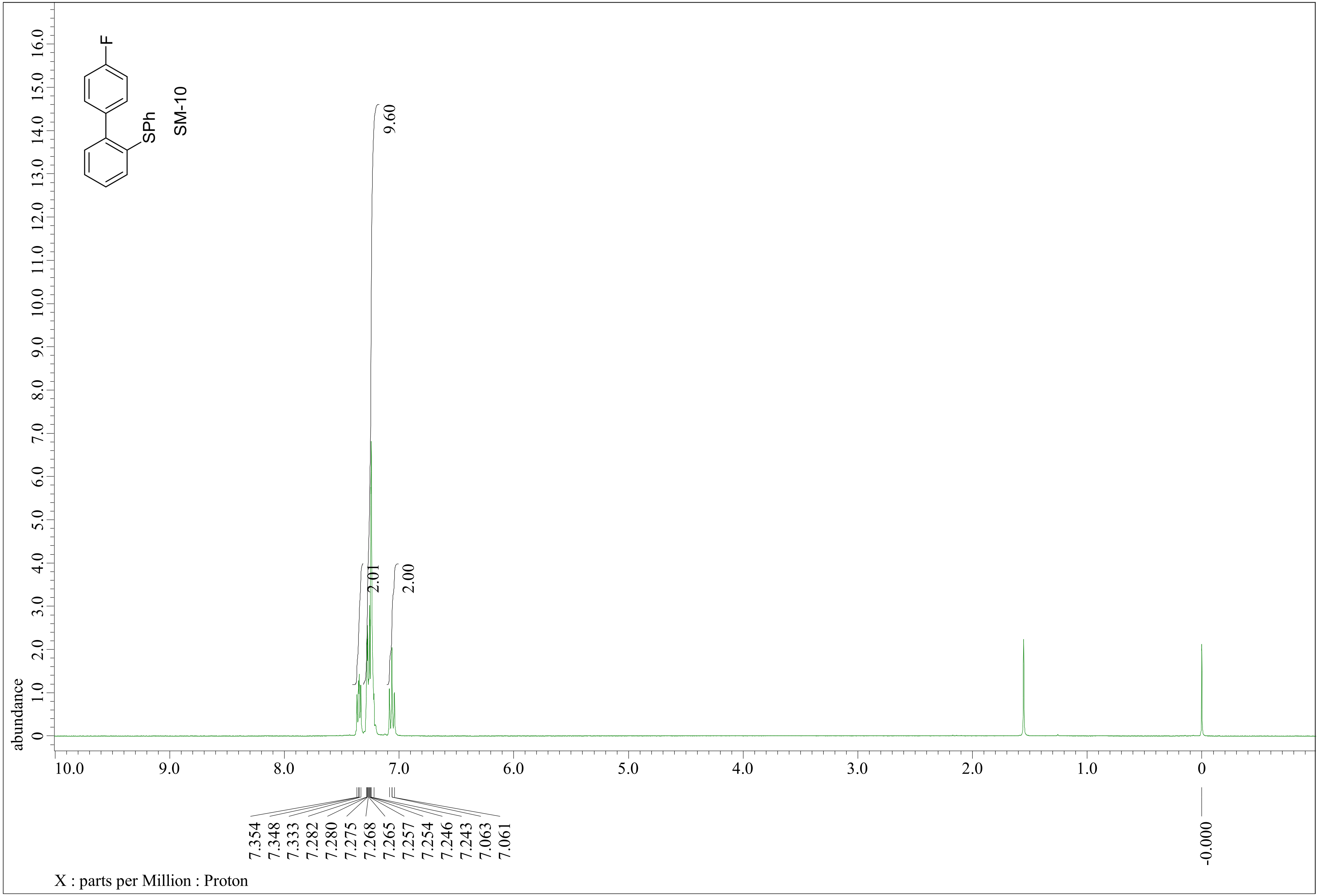
SPh
F
SM-10
9.60
2.01
2.00
abundance
X : parts per Million : Proton
7.354
7.348
7.333
7.282
7.280
7.275
7.268
7.265
7.257
7.254
7.246
7.243
7.063
7.061
-0.000

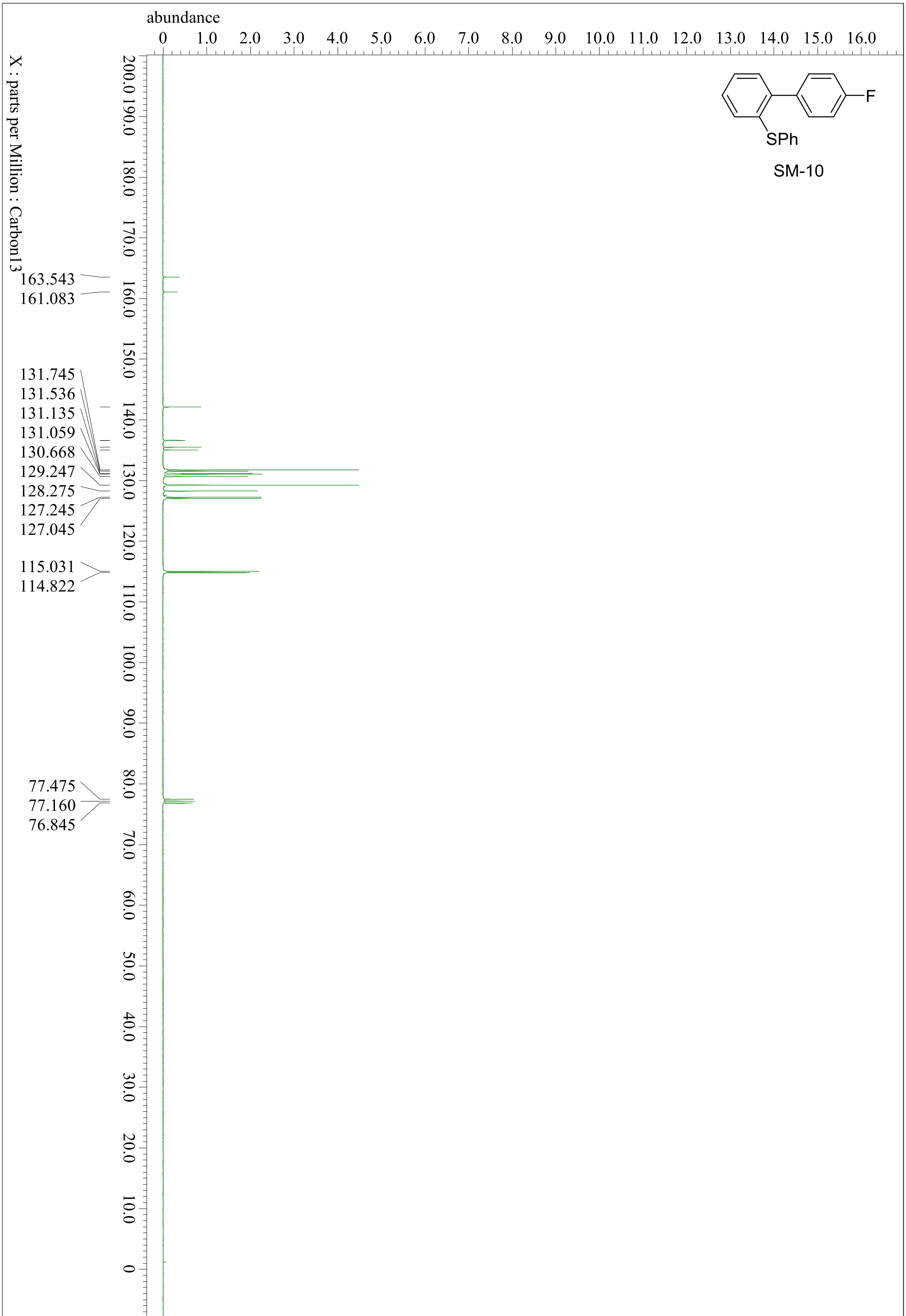

abundance
0 1.0 2.0 3.0 4.0 5.0 6.0 7.0 8.0 9.0 10.0 11.0 12.0 13.0 14.0 15.0 16.0
X : parts per Million : Carbon13
200.0 190.0 180.0 170.0 160.0 150.0 140.0 130.0 120.0 110.0 100.0 90.0 80.0 70.0 60.0 50.0 40.0 30.0 20.0 10.0 0
163.543
161.083
131.745
131.536
131.135
131.059
130.668
129.247
128.275
127.245
127.045
115.031
114.822
77.475
77.160
76.845
F
SPh
SM-10

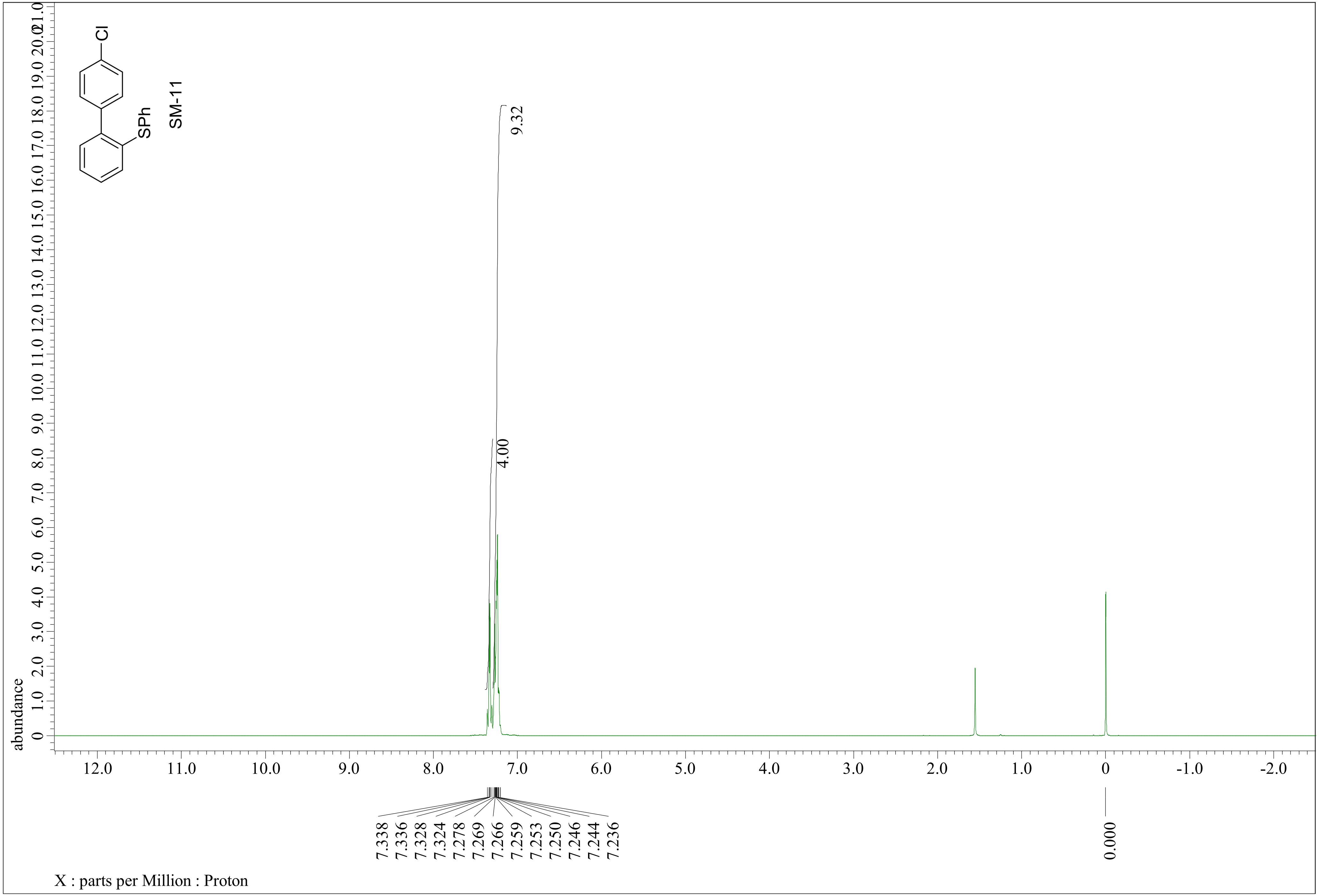

SPh
Cl
SM-11
abundance
X : parts per Million : Proton
4.00
9.32
7.338
7.336
7.328
7.324
7.278
7.269
7.266
7.259
7.253
7.250
7.246
7.244
7.236
0.000

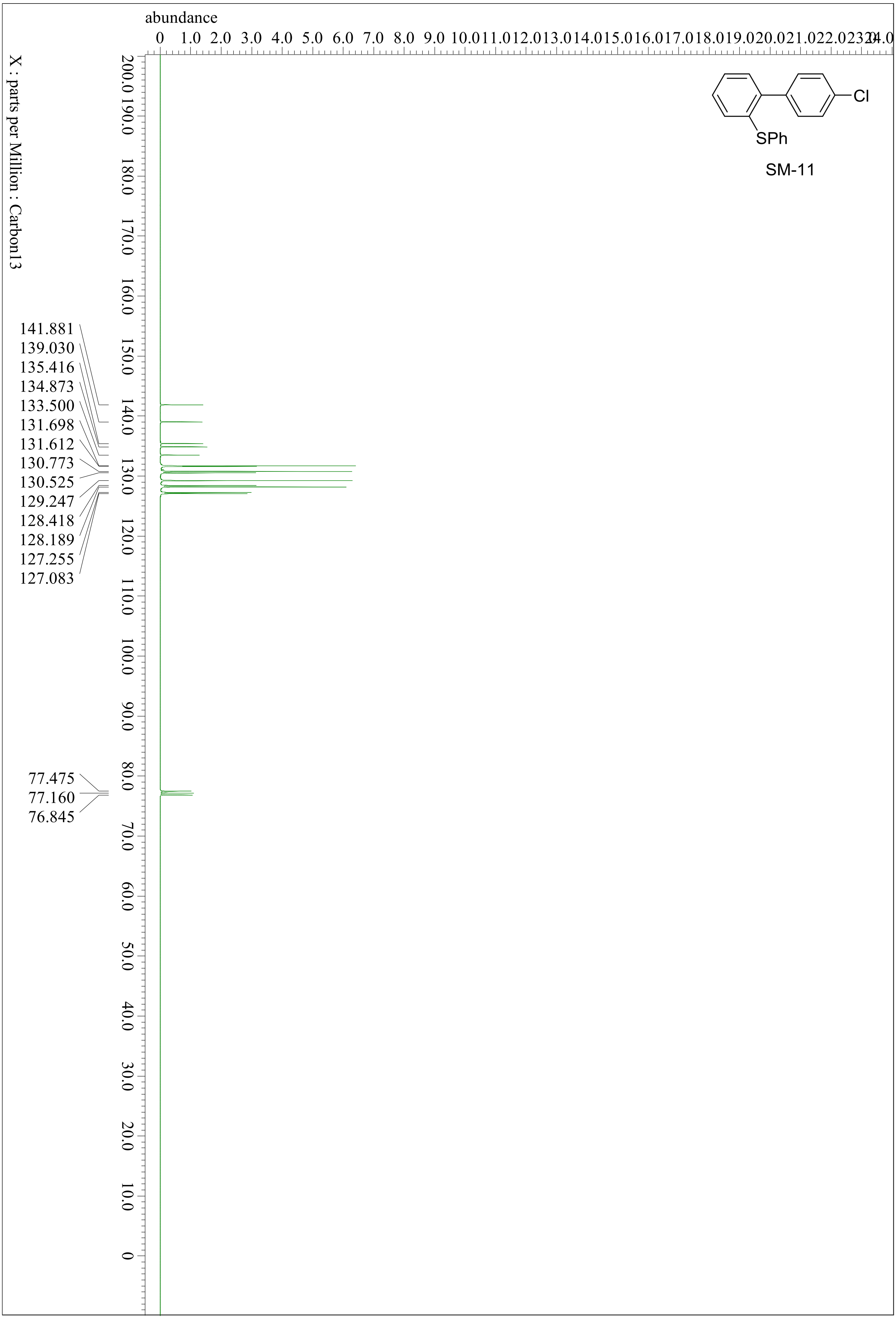

abundance
0 1.0 2.0 3.0 4.0 5.0 6.0 7.0 8.0 9.0 10.0 11.0 12.0 13.0 14.0 15.0 16.0 17.0 18.0 19.0 20.0 21.0 22.0 23.0 24.0
X : parts per Million : Carbon13
200.0 190.0 180.0 170.0 160.0 150.0 140.0 130.0 120.0 110.0 100.0 90.0 80.0 70.0 60.0 50.0 40.0 30.0 20.0 10.0 0
141.881
139.030
135.416
134.873
133.500
131.698
131.612
130.773
130.525
129.247
128.418
128.189
127.255
127.083
77.475
77.160
76.845
Cl
SPh
SM-11

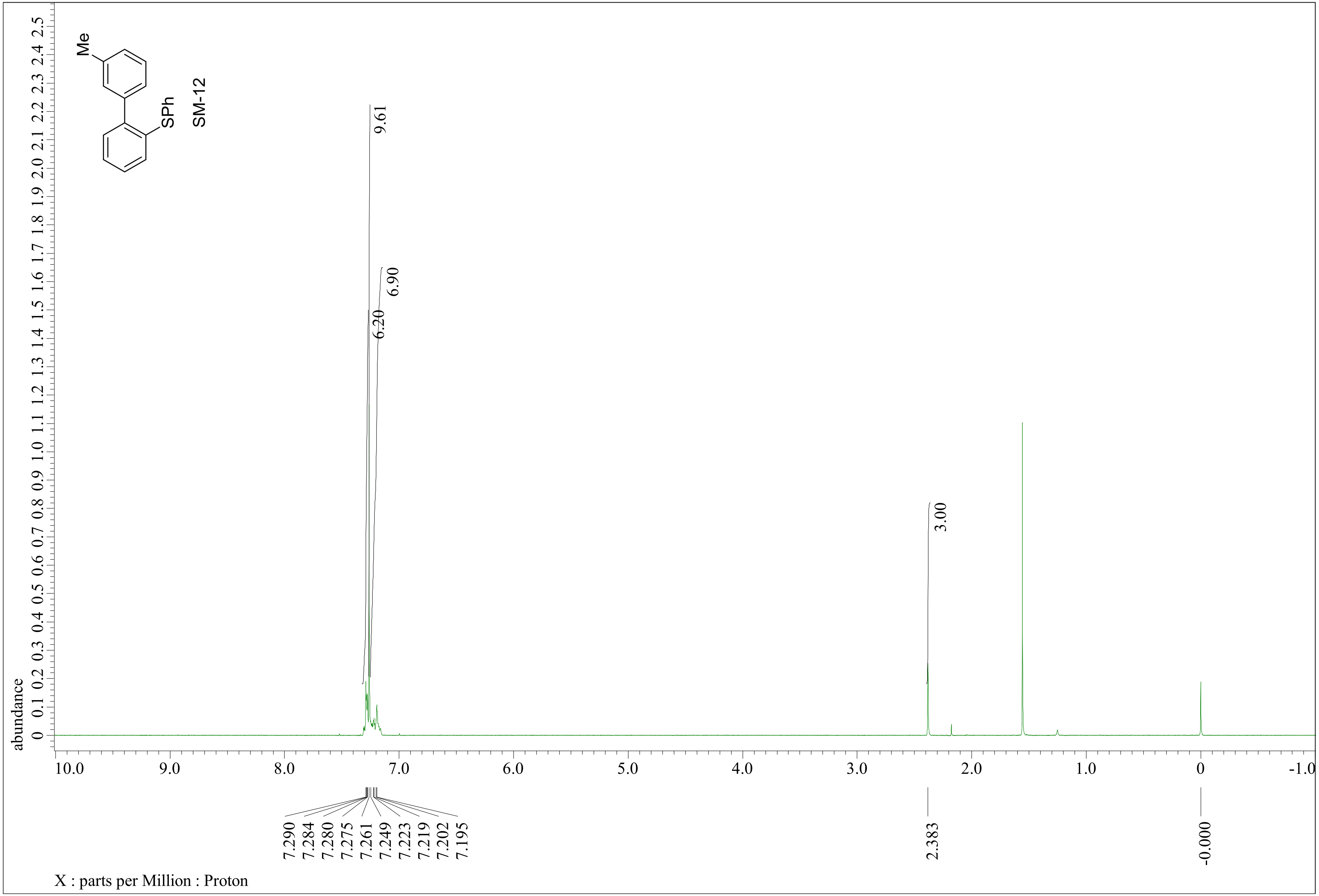
abundance
0 0.1 0.2 0.3 0.4 0.5 0.6 0.7 0.8 0.9 1.0 1.1 1.2 1.3 1.4 1.5 1.6 1.7 1.8 1.9 2.0 2.1 2.2 2.3 2.4 2.5
Me
SPh
SM-12
9.61
6.20
6.90
3.00
10.0 9.0 8.0 7.0 6.0 5.0 4.0 3.0 2.0 1.0 0 -1.0
7.290
7.284
7.280
7.275
7.261
7.249
7.223
7.219
7.202
7.195
2.383
-0.000
X : parts per Million : Proton

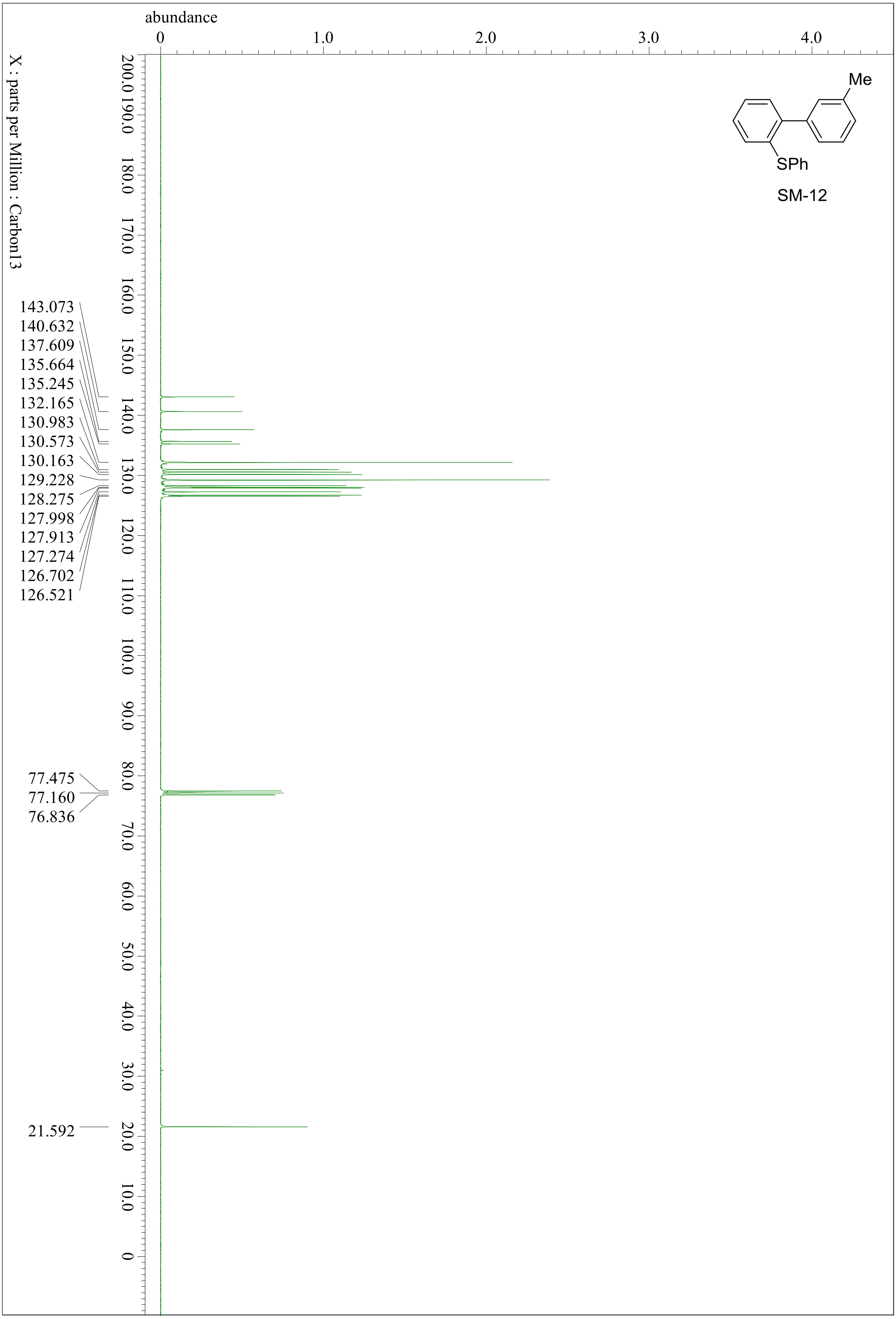
abundance
0
1.0
2.0
3.0
4.0
X : parts per Million : Carbon13
200.0
190.0
180.0
170.0
160.0
150.0
140.0
130.0
120.0
110.0
100.0
90.0
80.0
70.0
60.0
50.0
40.0
30.0
20.0
10.0
0
143.073
140.632
137.609
135.664
135.245
132.165
130.983
130.573
130.163
129.228
128.275
127.998
127.913
127.274
126.702
126.521
77.475
77.160
76.836
21.592
Me
SPh
SM-12

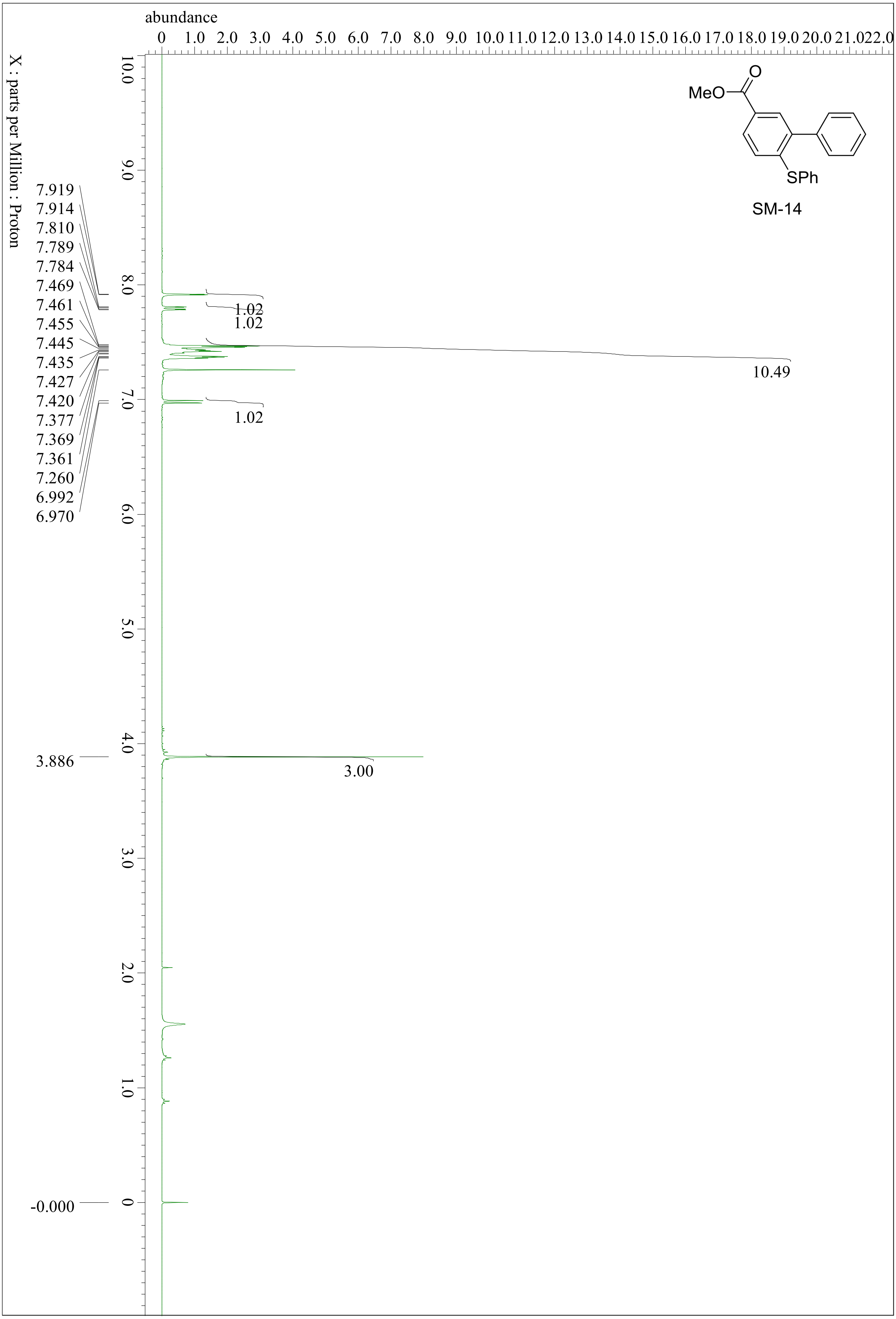
abundance
X : parts per Million : Proton
7.919
7.914
7.810
7.789
7.784
7.469
7.461
7.455
7.445
7.435
7.427
7.420
7.377
7.369
7.361
7.260
6.992
6.970
3.886
-0.000
1.02
1.02
10.49
1.02
3.00
MeO
O
SPh
SM-14

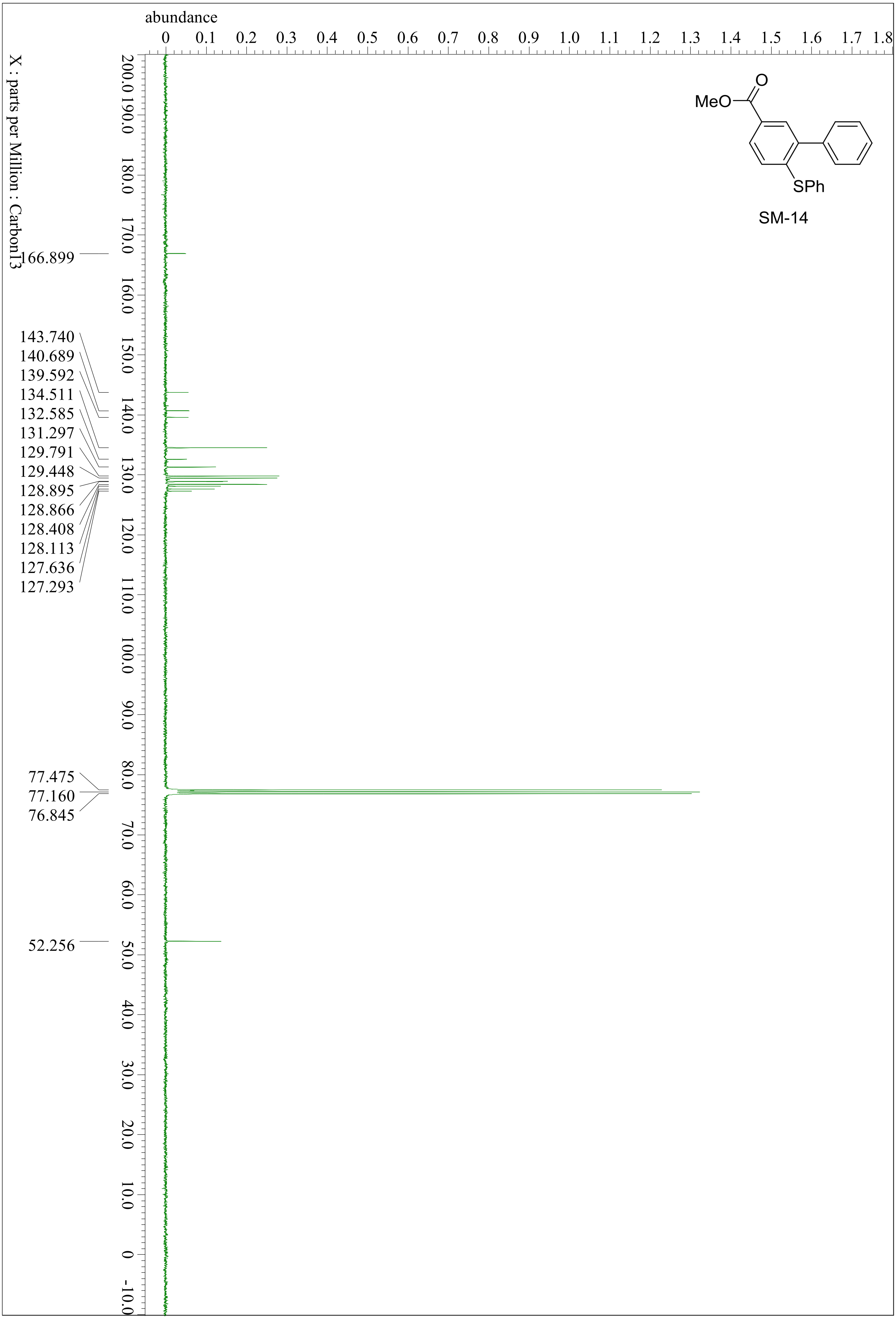
abundance
0 0.1 0.2 0.3 0.4 0.5 0.6 0.7 0.8 0.9 1.0 1.1 1.2 1.3 1.4 1.5 1.6 1.7 1.8
X : parts per Million : Carbon13
200.0 190.0 180.0 170.0 160.0 150.0 140.0 130.0 120.0 110.0 100.0 90.0 80.0 70.0 60.0 50.0 40.0 30.0 20.0 10.0 0 -10.0
166.899
143.740
140.689
139.592
134.511
132.585
131.297
129.791
129.448
128.895
128.866
128.408
128.113
127.636
127.293
77.475
77.160
76.845
52.256
MeO
O
SPh
SM-14

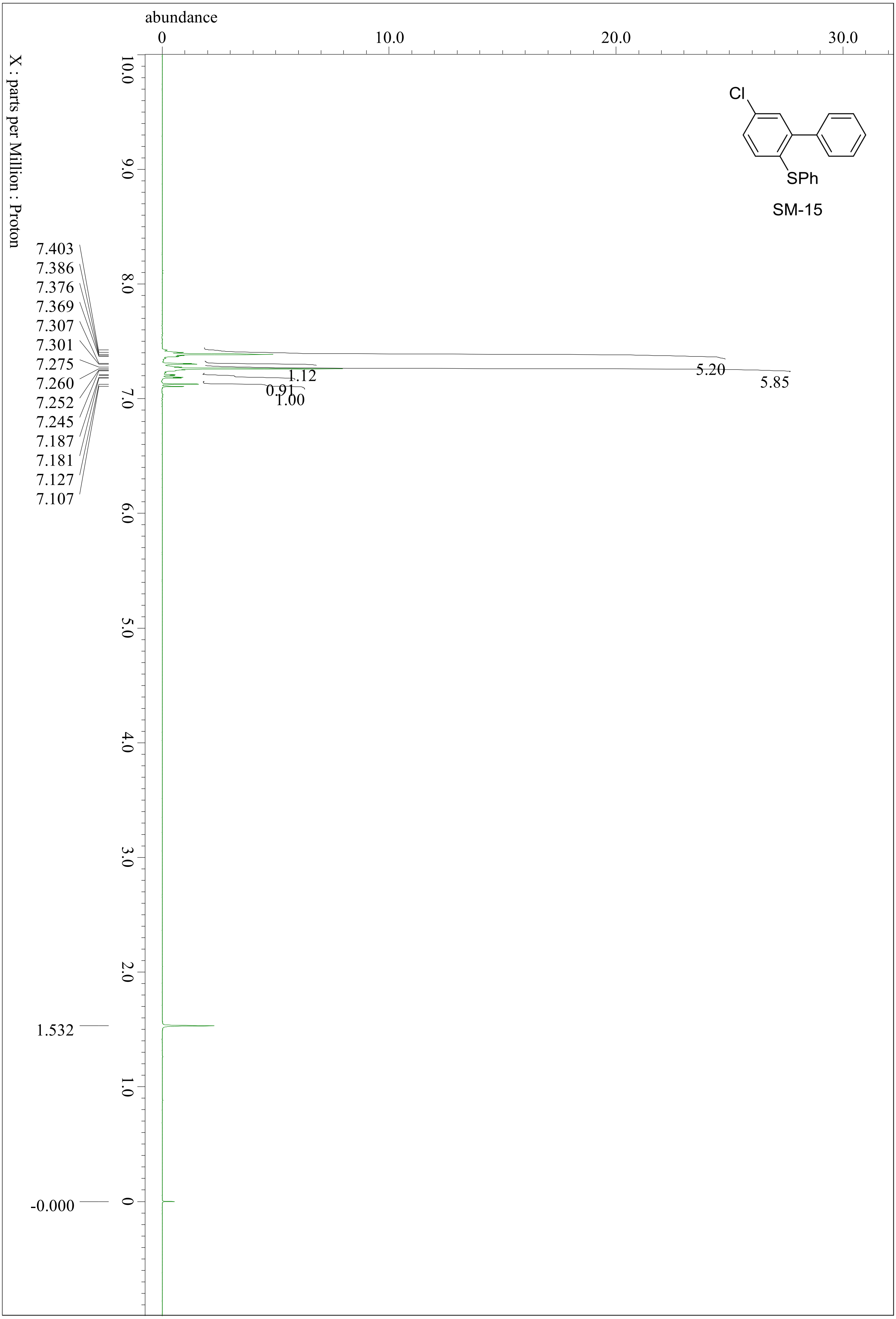

abundance
0
10.0
20.0
30.0
X : parts per Million : Proton
Cl
SPh
SM-15
7.403
7.386
7.376
7.369
7.307
7.301
7.275
7.260
7.252
7.245
7.187
7.181
7.127
7.107
1.532
-0.000
5.20
5.85
1.12
0.91
1.00
10.0
9.0
8.0
7.0
6.0
5.0
4.0
3.0
2.0
1.0
0

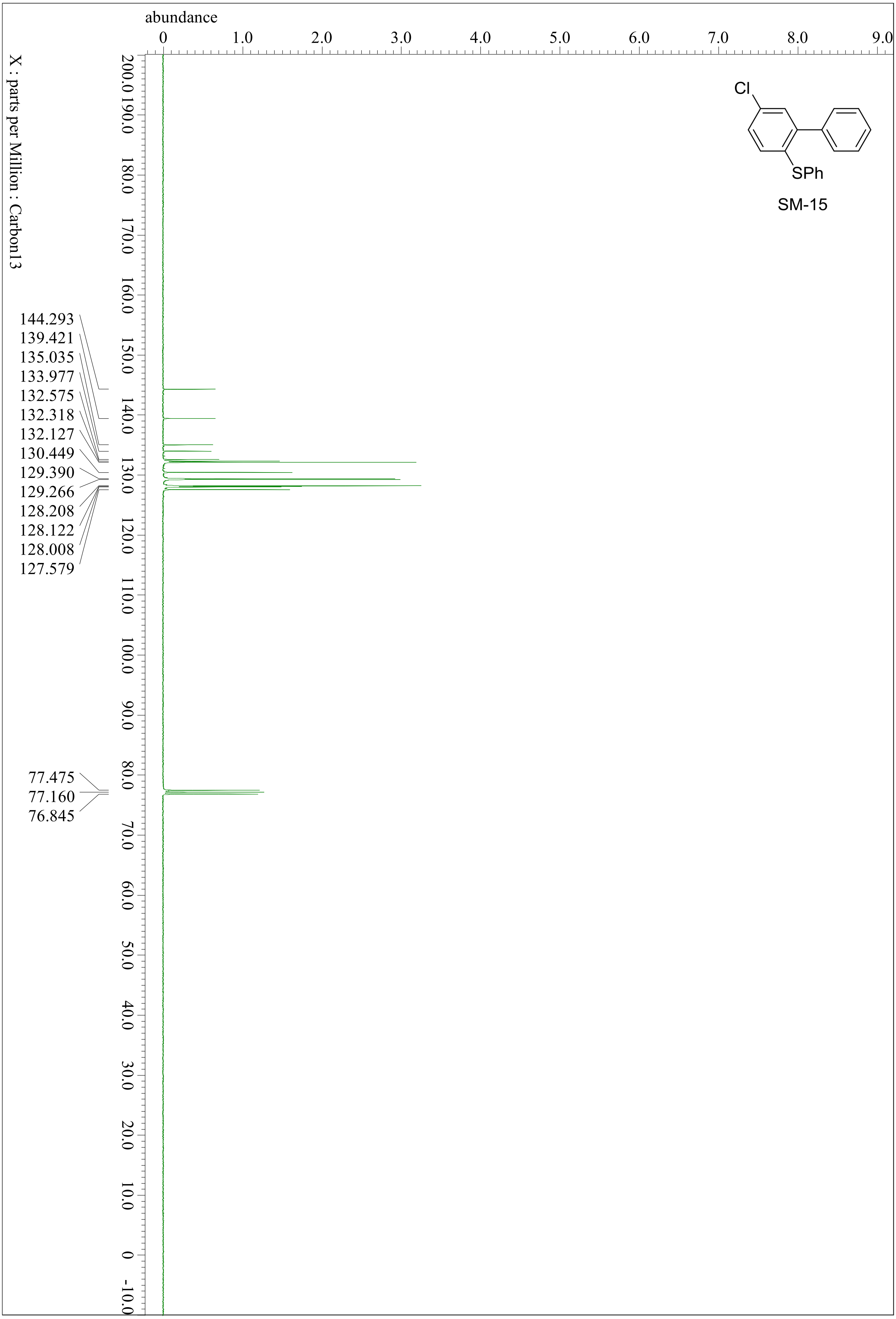

abundance
0
1.0
2.0
3.0
4.0
5.0
6.0
7.0
8.0
9.0
X : parts per Million : Carbon13
200.0
190.0
180.0
170.0
160.0
150.0
140.0
130.0
120.0
110.0
100.0
90.0
80.0
70.0
60.0
50.0
40.0
30.0
20.0
10.0
0
-10.0
144.293
139.421
135.035
133.977
132.575
132.318
132.127
130.449
129.390
129.266
128.208
128.122
128.008
127.579
77.475
77.160
76.845
Cl
SPh
SM-15

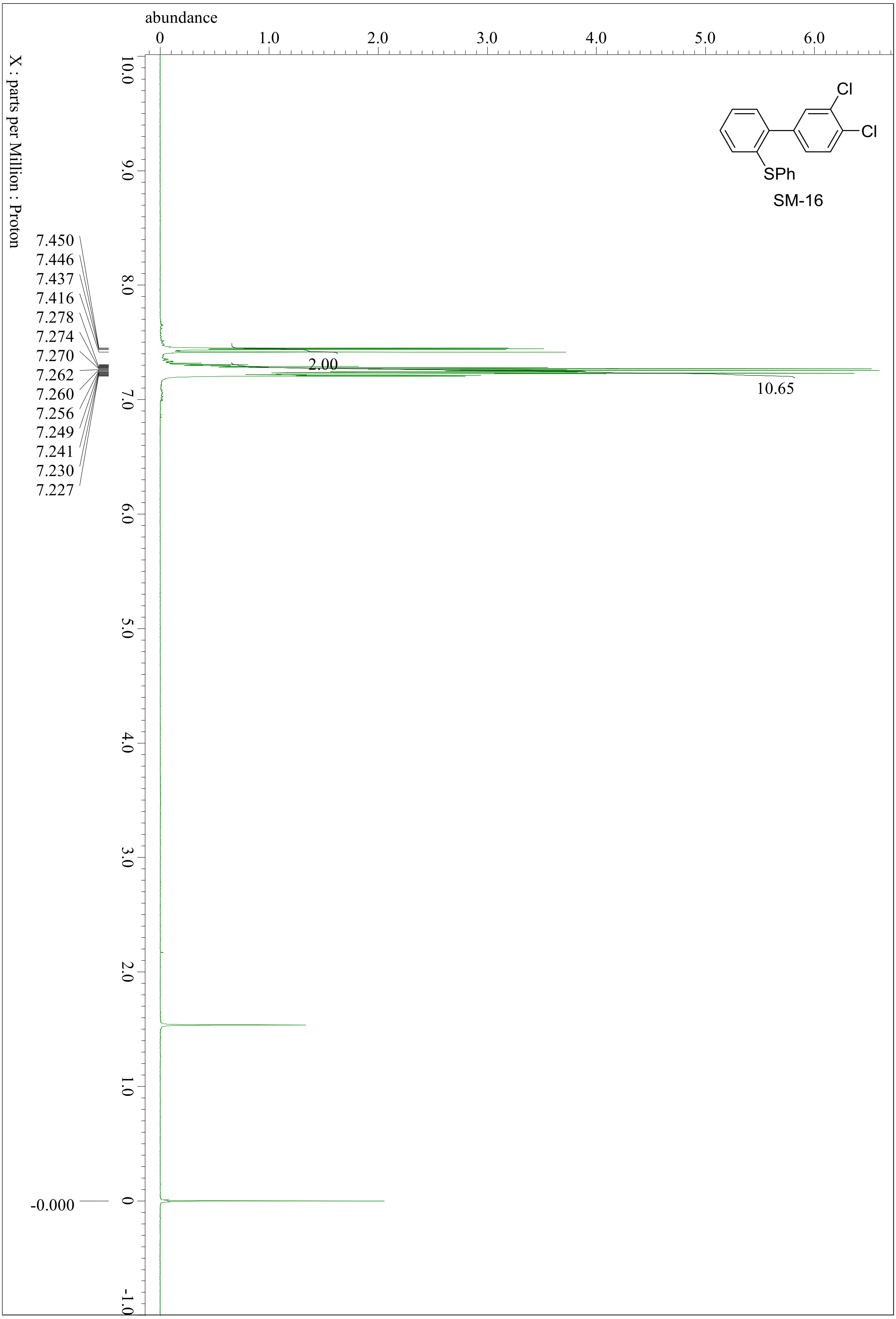

abundance
0
1.0
2.0
3.0
4.0
5.0
6.0
X : parts per Million : Proton
10.0
9.0
8.0
7.0
6.0
5.0
4.0
3.0
2.0
1.0
0
-1.0
7.450
7.446
7.437
7.416
7.278
7.274
7.270
7.262
7.260
7.256
7.249
7.241
7.230
7.227
-0.000
2.00
10.65
Cl
Cl
SPh
SM-16

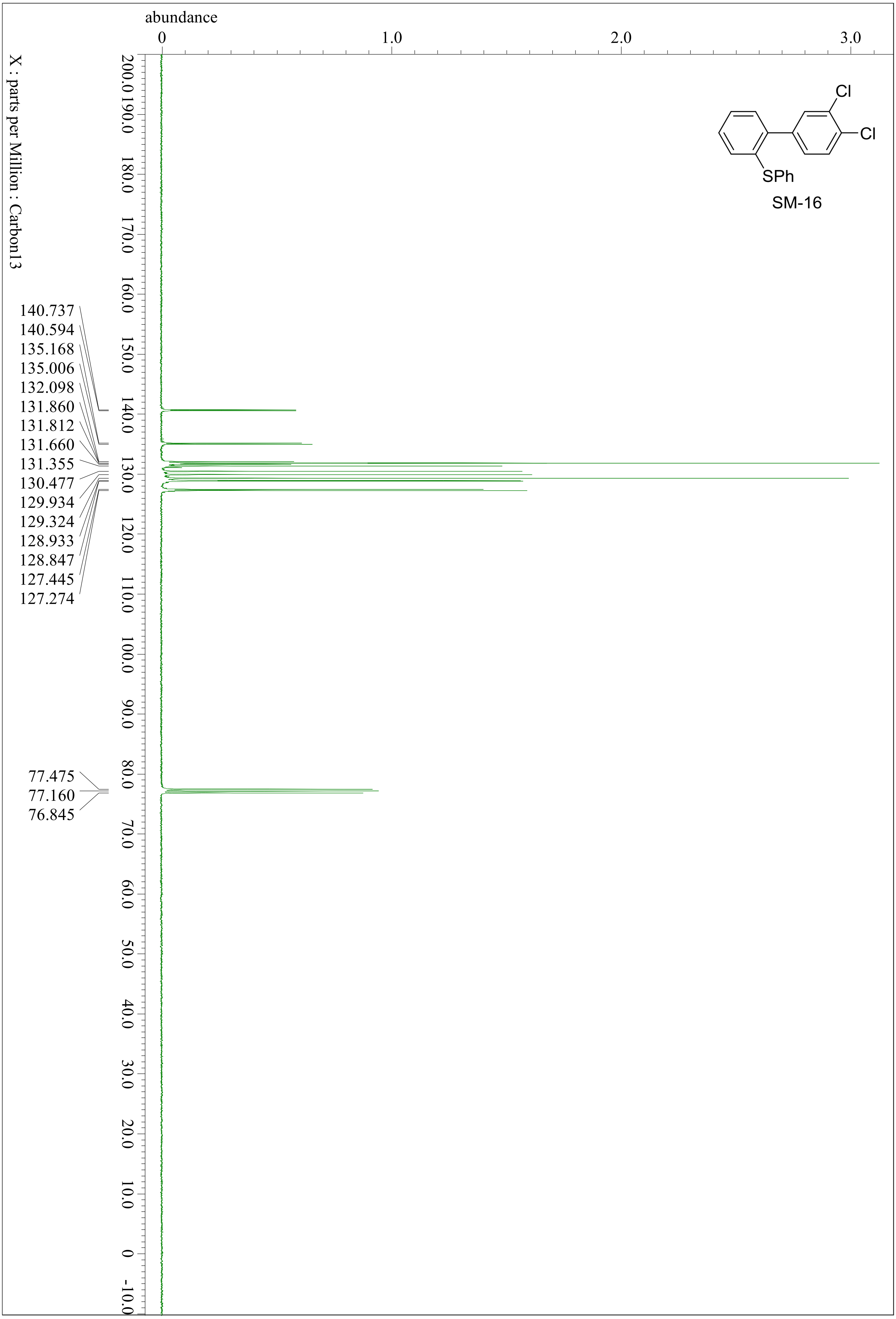

abundance
0
1.0
2.0
3.0
X : parts per Million : Carbon13
200.0
190.0
180.0
170.0
160.0
150.0
140.0
130.0
120.0
110.0
100.0
90.0
80.0
70.0
60.0
50.0
40.0
30.0
20.0
10.0
0
-10.0
140.737
140.594
135.168
135.006
132.098
131.860
131.812
131.660
131.355
130.477
129.934
129.324
128.933
128.847
127.445
127.274
77.475
77.160
76.845
Cl
Cl
SPh
SM-16

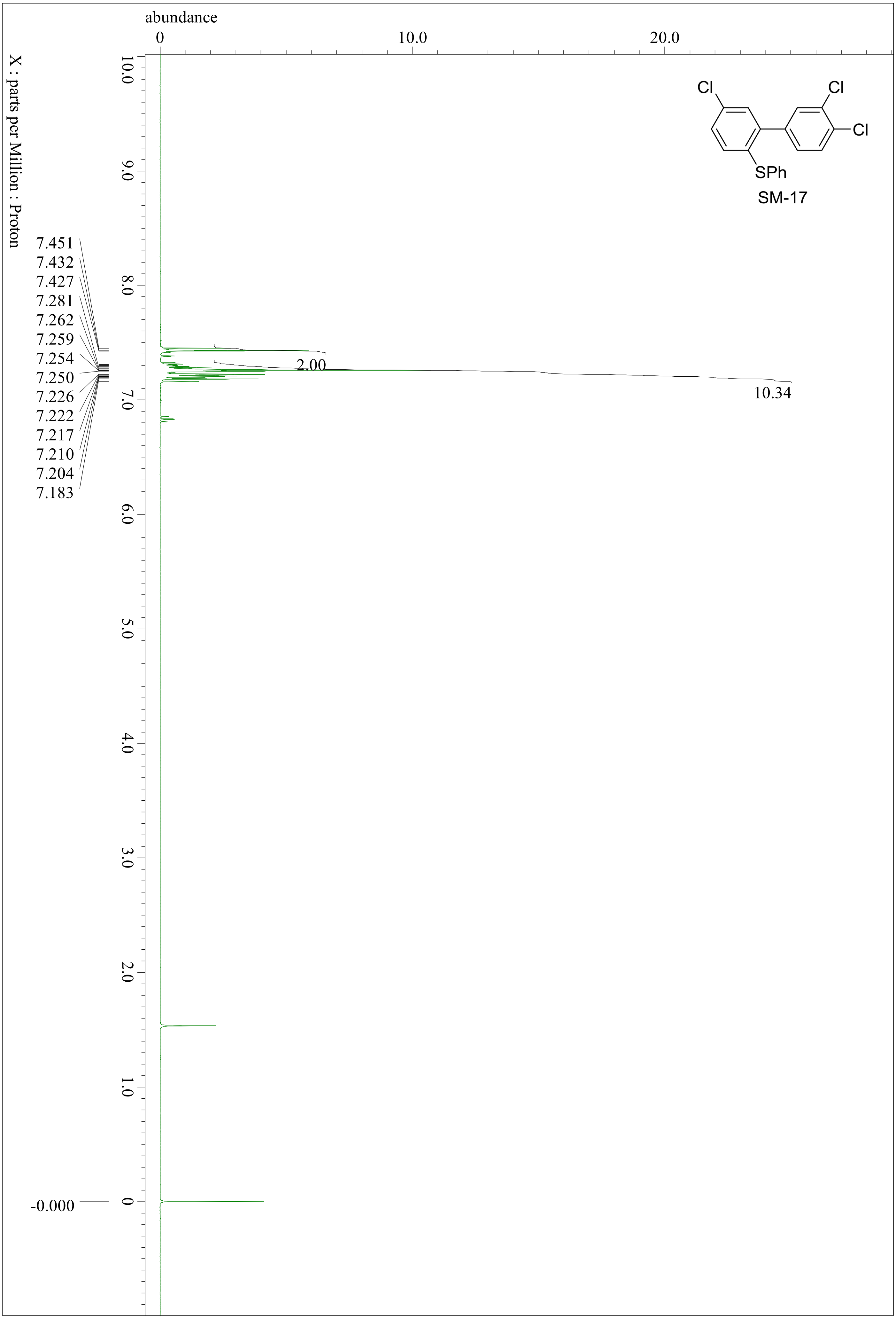
abundance
0
10.0
20.0
X : parts per Million : Proton
10.0
9.0
8.0
7.0
6.0
5.0
4.0
3.0
2.0
1.0
0
7.451
7.432
7.427
7.281
7.262
7.259
7.254
7.250
7.226
7.222
7.217
7.210
7.204
7.183
-0.000
2.00
10.34
Cl
Cl
Cl
SPh
SM-17

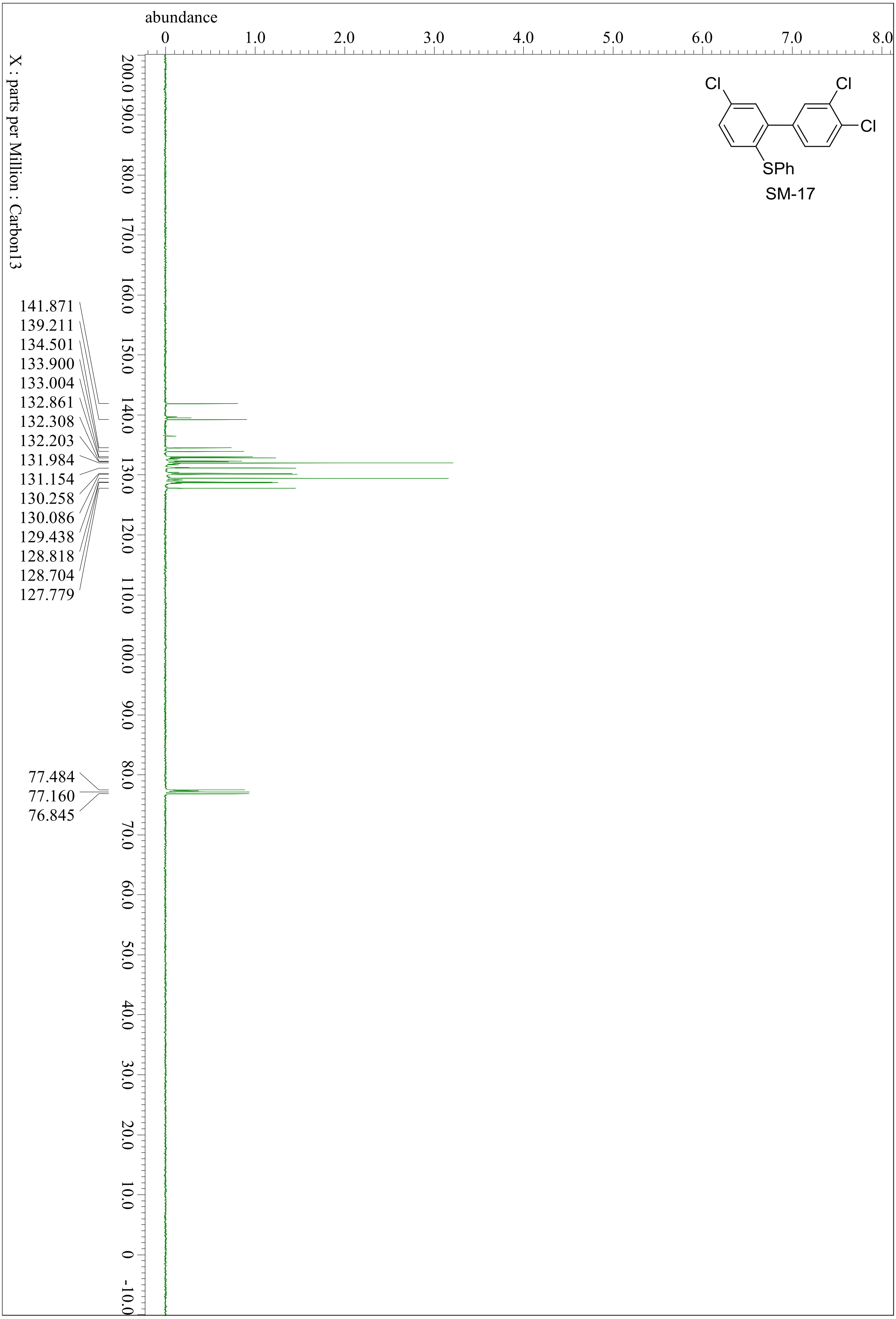
abundance
0
1.0
2.0
3.0
4.0
5.0
6.0
7.0
8.0
X : parts per Million : Carbon13
200.0
190.0
180.0
170.0
160.0
150.0
140.0
130.0
120.0
110.0
100.0
90.0
80.0
70.0
60.0
50.0
40.0
30.0
20.0
10.0
0
-10.0
141.871
139.211
134.501
133.900
133.004
132.861
132.308
132.203
131.984
131.154
130.258
130.086
129.438
128.818
128.704
127.779
77.484
77.160
76.845
Cl
Cl
Cl
SPh
SM-17

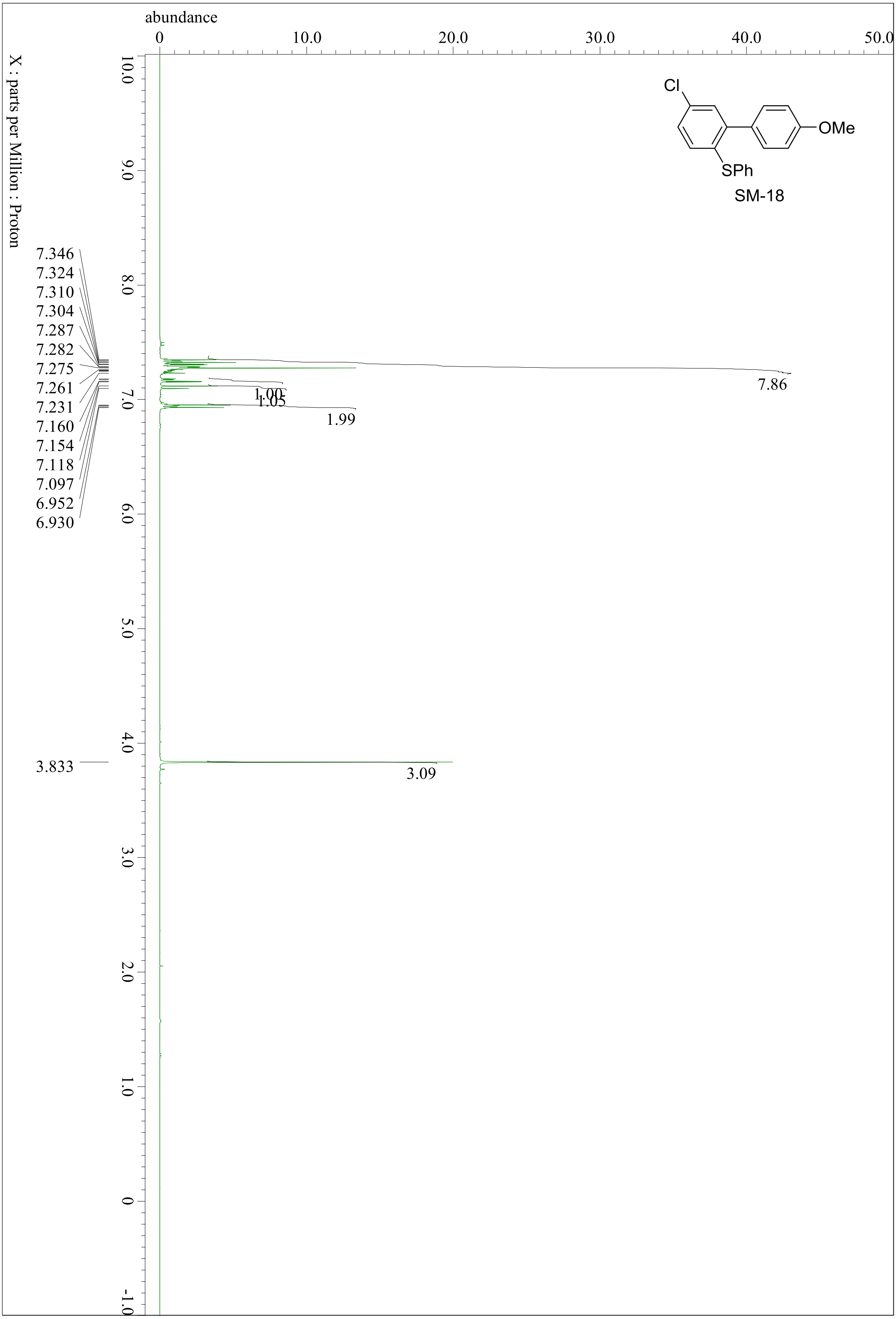
abundance
0
10.0
20.0
30.0
40.0
50.0
X : parts per Million : Proton
10.0
9.0
8.0
7.0
6.0
5.0
4.0
3.0
2.0
1.0
0
-1.0
7.346
7.324
7.310
7.304
7.287
7.282
7.275
7.261
7.231
7.160
7.154
7.118
7.097
6.952
6.930
3.833
7.86
1.00
1.05
1.99
3.09
Cl
OMe
SPh
SM-18

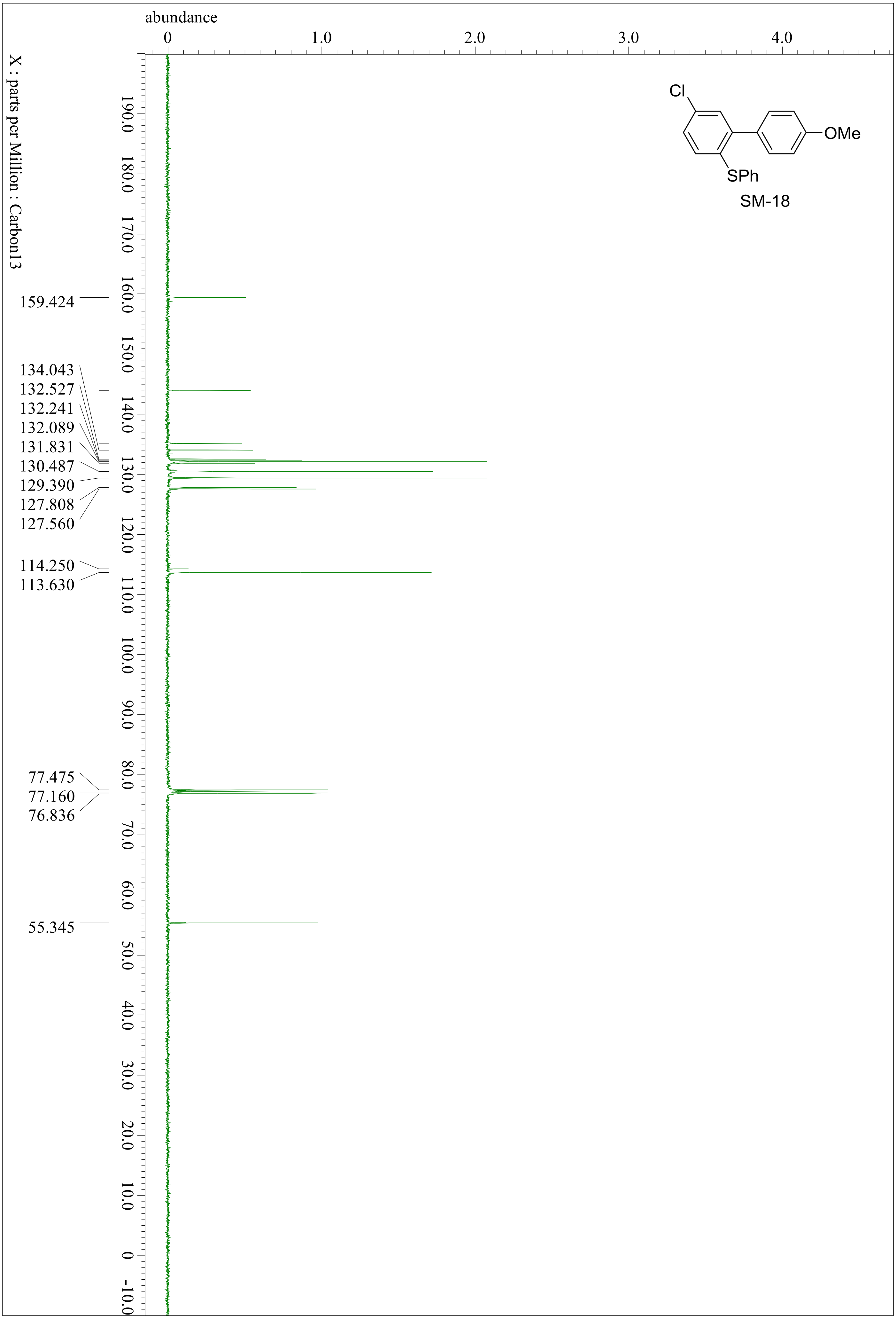

abundance
0
1.0
2.0
3.0
4.0
X : parts per Million : Carbon13
190.0
180.0
170.0
160.0
150.0
140.0
130.0
120.0
110.0
100.0
90.0
80.0
70.0
60.0
50.0
40.0
30.0
20.0
10.0
0
-10.0
159.424
134.043
132.527
132.241
132.089
131.831
130.487
129.390
127.808
127.560
114.250
113.630
77.475
77.160
76.836
55.345
Cl
OMe
SPh
SM-18

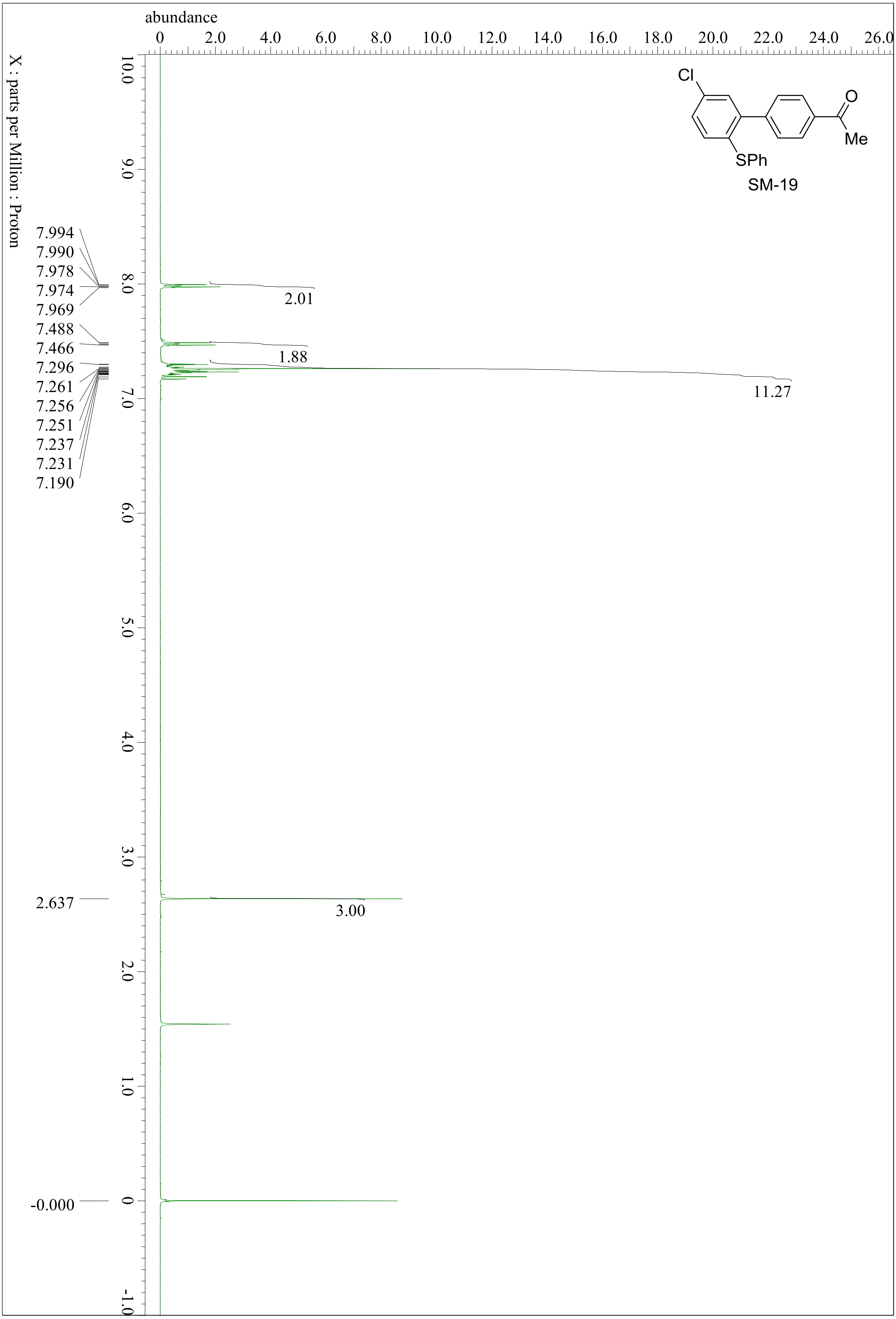

abundance
0
2.0
4.0
6.0
8.0
10.0
12.0
14.0
16.0
18.0
20.0
22.0
24.0
26.0
X : parts per Million : Proton
10.0
9.0
8.0
7.0
6.0
5.0
4.0
3.0
2.0
1.0
0
-1.0
7.994
7.990
7.978
7.974
7.969
7.488
7.466
7.296
7.261
7.256
7.251
7.237
7.231
7.190
2.637
-0.000
2.01
1.88
11.27
3.00
Cl
O
Me
SPh
SM-19

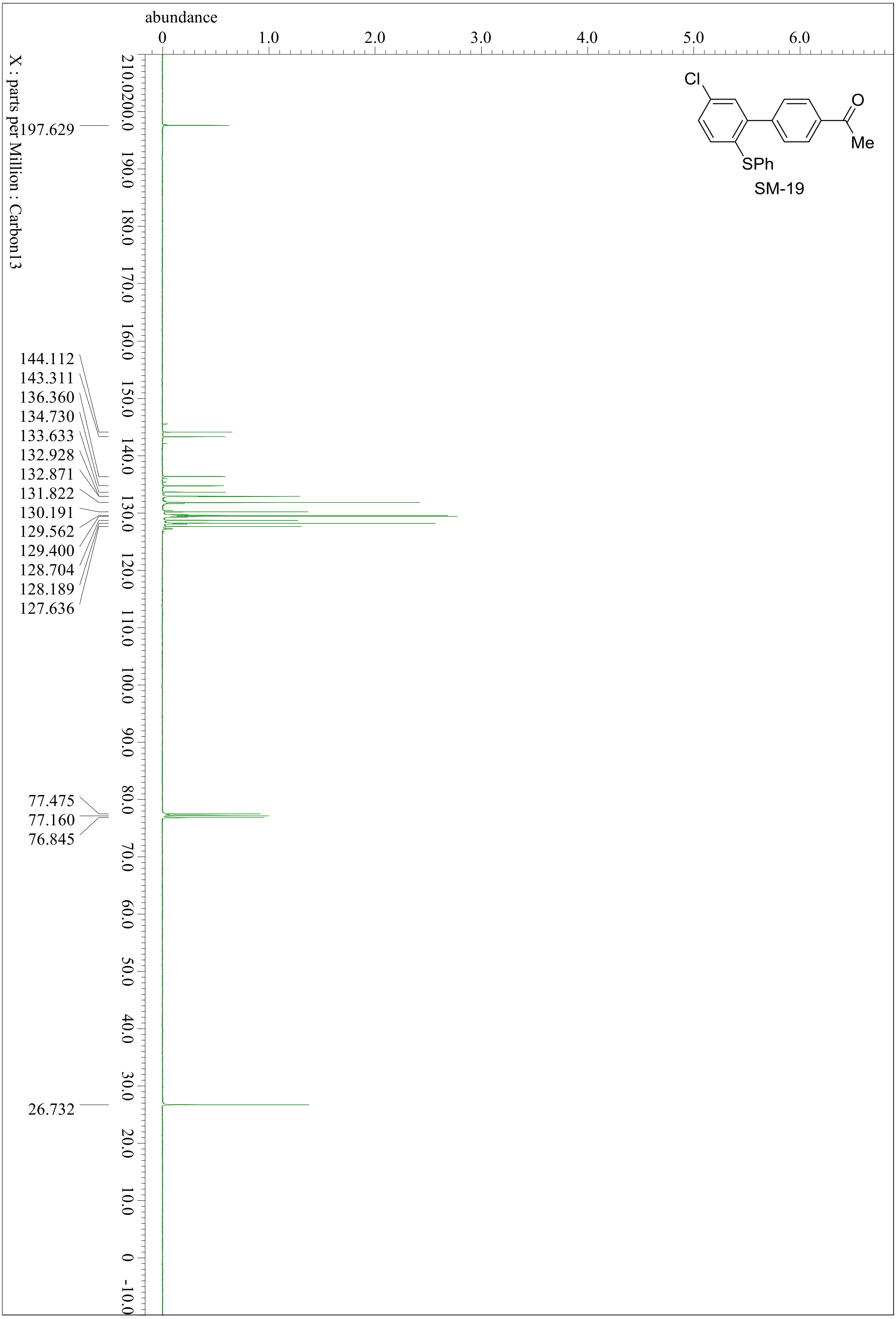
abundance
0
1.0
2.0
3.0
4.0
5.0
6.0
X : parts per Million : Carbon13
197.629
144.112
143.311
136.360
134.730
133.633
132.928
132.871
131.822
130.191
129.562
129.400
128.704
128.189
127.636
77.475
77.160
76.845
26.732
210.0
200.0
190.0
180.0
170.0
160.0
150.0
140.0
130.0
120.0
110.0
100.0
90.0
80.0
70.0
60.0
50.0
40.0
30.0
20.0
10.0
0
-10.0
Cl
O
Me
SPh
SM-19

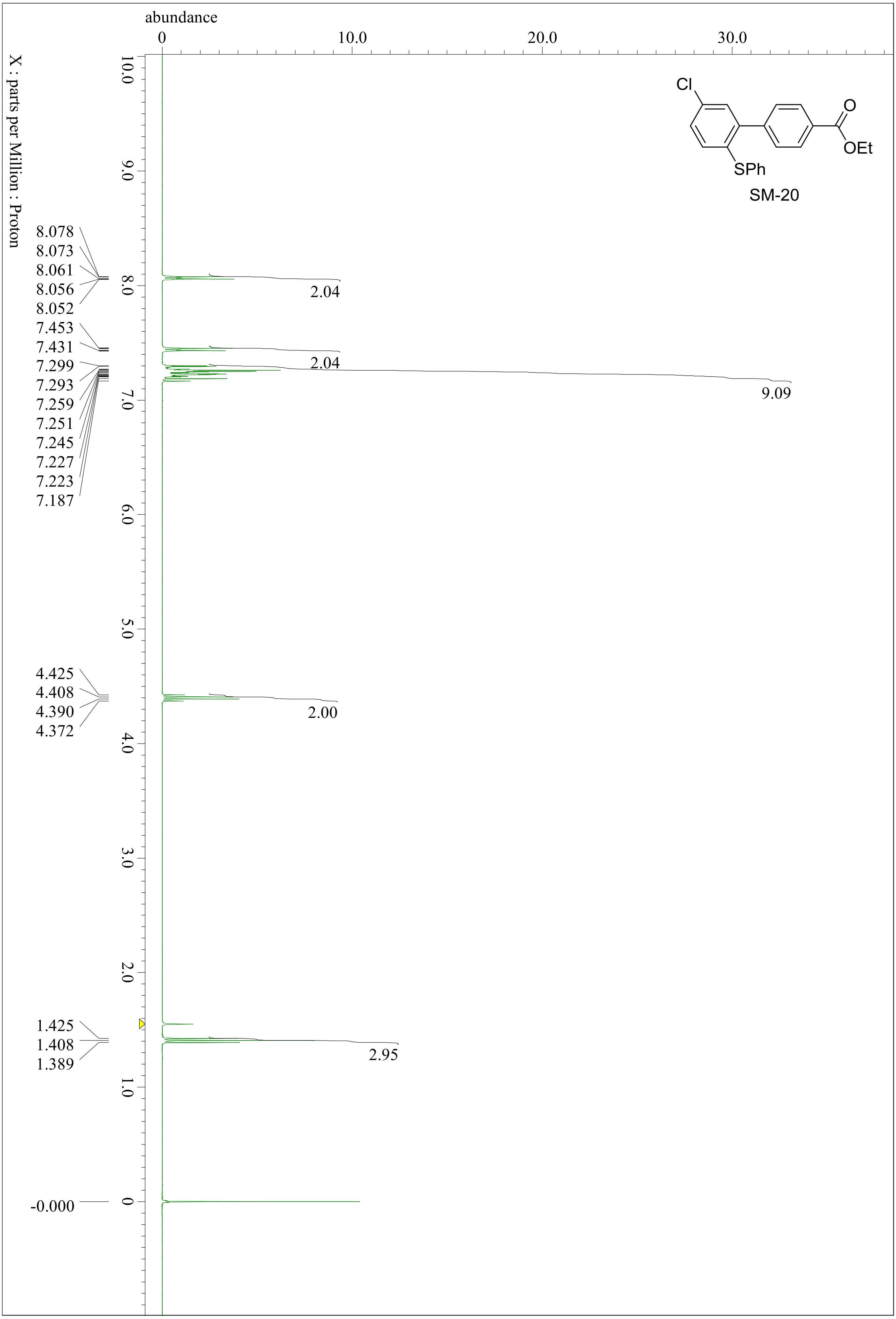
abundance
0
10.0
20.0
30.0
X : parts per Million : Proton
10.0
9.0
8.0
7.0
6.0
5.0
4.0
3.0
2.0
1.0
0
8.078
8.073
8.061
8.056
8.052
7.453
7.431
7.299
7.293
7.259
7.251
7.245
7.227
7.223
7.187
4.425
4.408
4.390
4.372
1.425
1.408
1.389
-0.000
2.04
2.04
9.09
2.00
2.95
Cl
O
OEt
SPh
SM-20

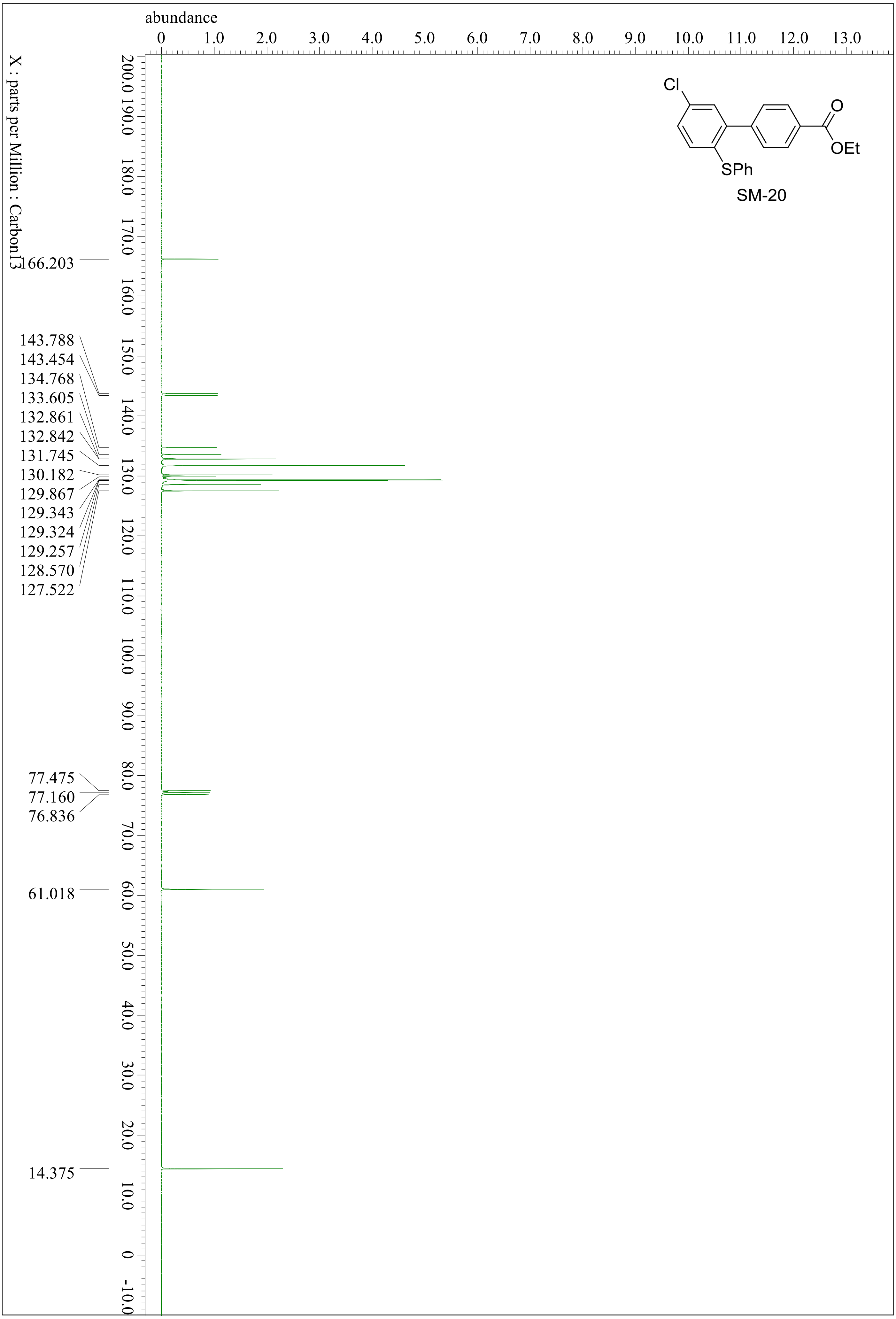
abundance
0 1.0 2.0 3.0 4.0 5.0 6.0 7.0 8.0 9.0 10.0 11.0 12.0 13.0
X : parts per Million : Carbon13
200.0 190.0 180.0 170.0 160.0 150.0 140.0 130.0 120.0 110.0 100.0 90.0 80.0 70.0 60.0 50.0 40.0 30.0 20.0 10.0 0 -10.0
166.203
143.788
143.454
134.768
133.605
132.861
132.842
131.745
130.182
129.867
129.343
129.324
129.257
128.570
127.522
77.475
77.160
76.836
61.018
14.375
Cl
O
OEt
SPh
SM-20

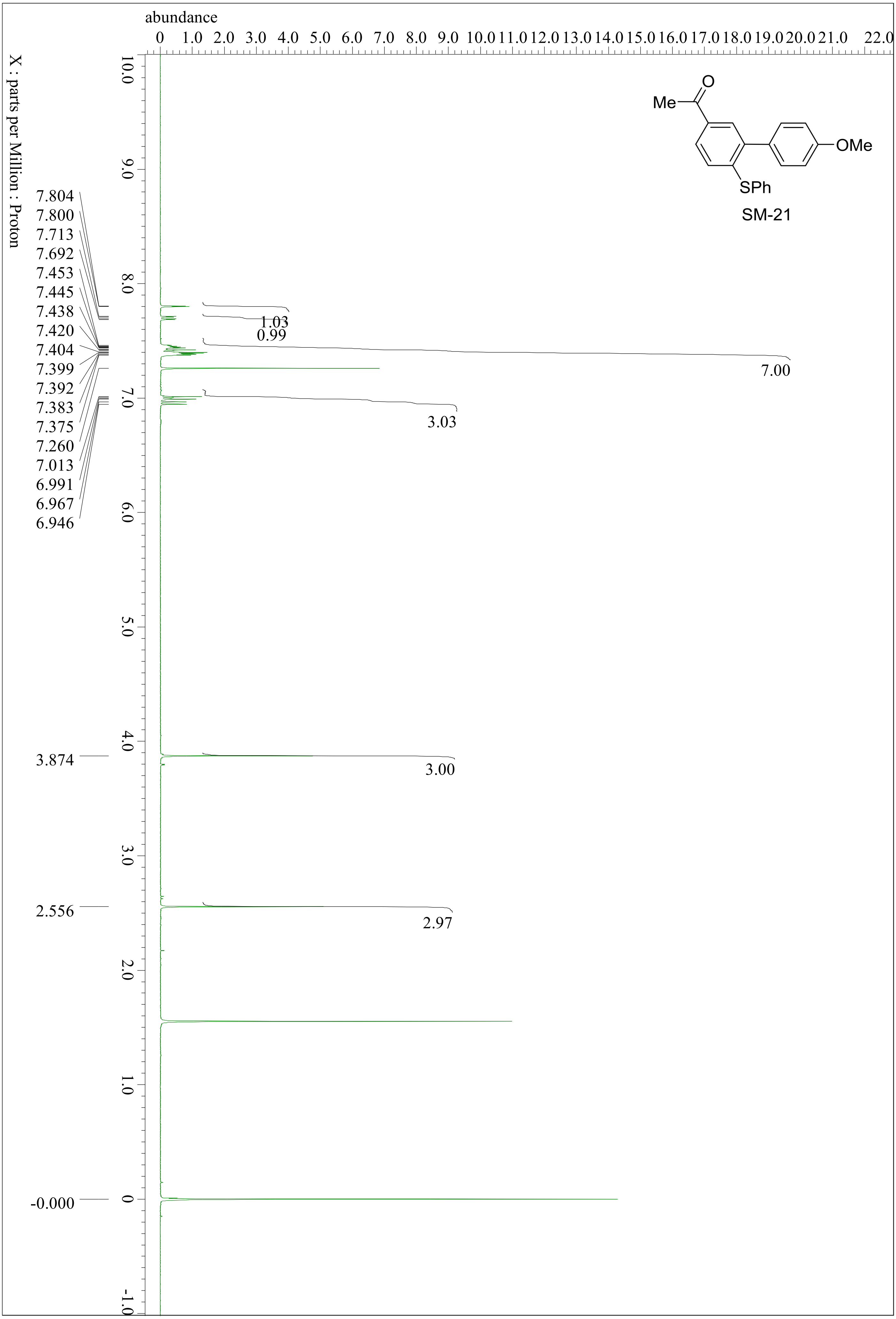
abundance
0 1.0 2.0 3.0 4.0 5.0 6.0 7.0 8.0 9.0 10.0 11.0 12.0 13.0 14.0 15.0 16.0 17.0 18.0 19.0 20.0 21.0 22.0
X : parts per Million : Proton
10.0 9.0 8.0 7.0 6.0 5.0 4.0 3.0 2.0 1.0 0 -1.0
7.804
7.800
7.713
7.692
7.453
7.445
7.438
7.420
7.404
7.399
7.392
7.383
7.375
7.260
7.013
6.991
6.967
6.946
3.874
2.556
-0.000
1.03
0.99
7.00
3.03
3.00
2.97
Me
O
OMe
SPh
SM-21

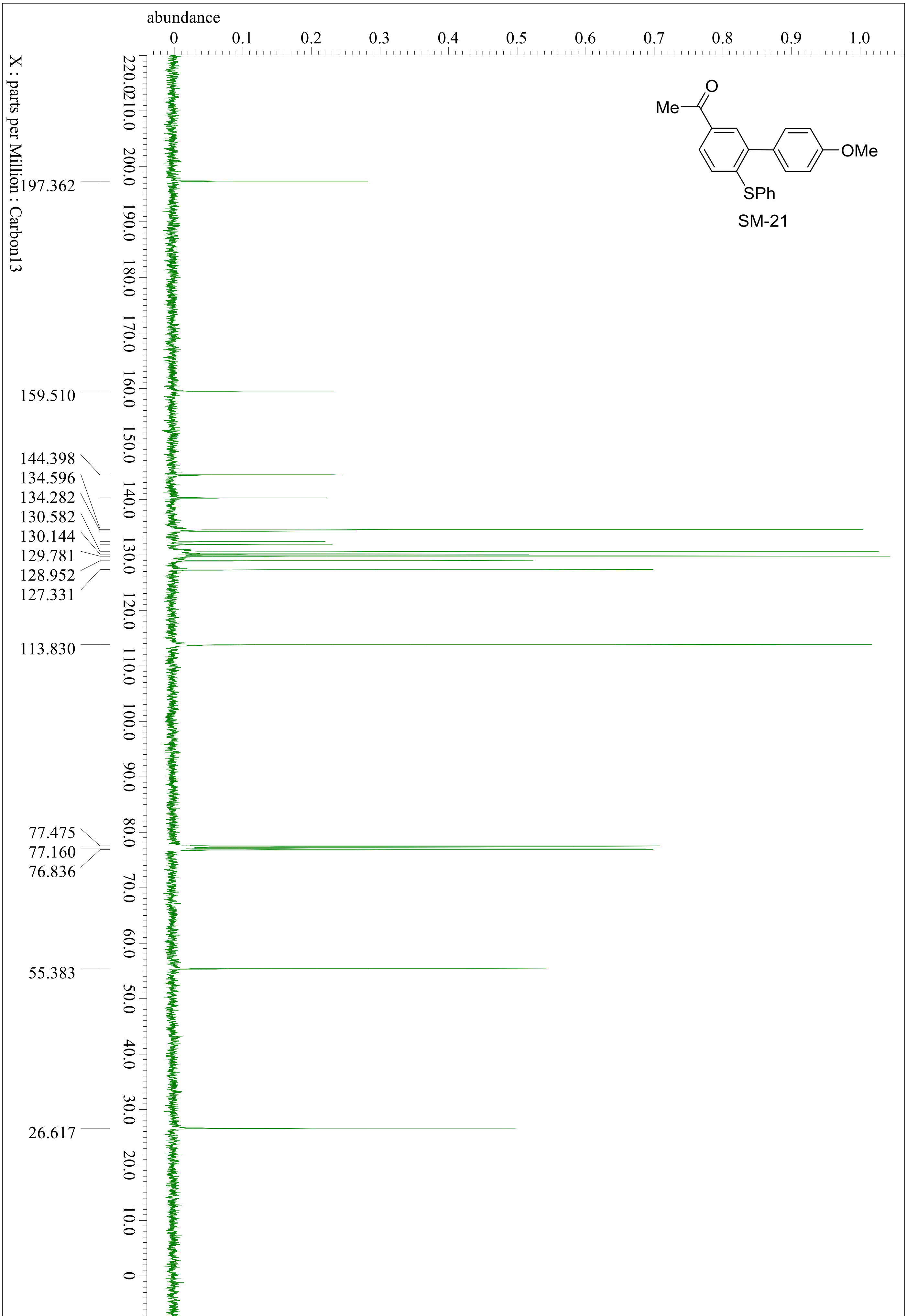
abundance
0
0.1
0.2
0.3
0.4
0.5
0.6
0.7
0.8
0.9
1.0
X : parts per Million : Carbon13
220.0
210.0
200.0
190.0
180.0
170.0
160.0
150.0
140.0
130.0
120.0
110.0
100.0
90.0
80.0
70.0
60.0
50.0
40.0
30.0
20.0
10.0
0
197.362
159.510
144.398
134.596
134.282
130.582
130.144
129.781
128.952
127.331
113.830
77.475
77.160
76.836
55.383
26.617
O
Me
OMe
SPh
SM-21

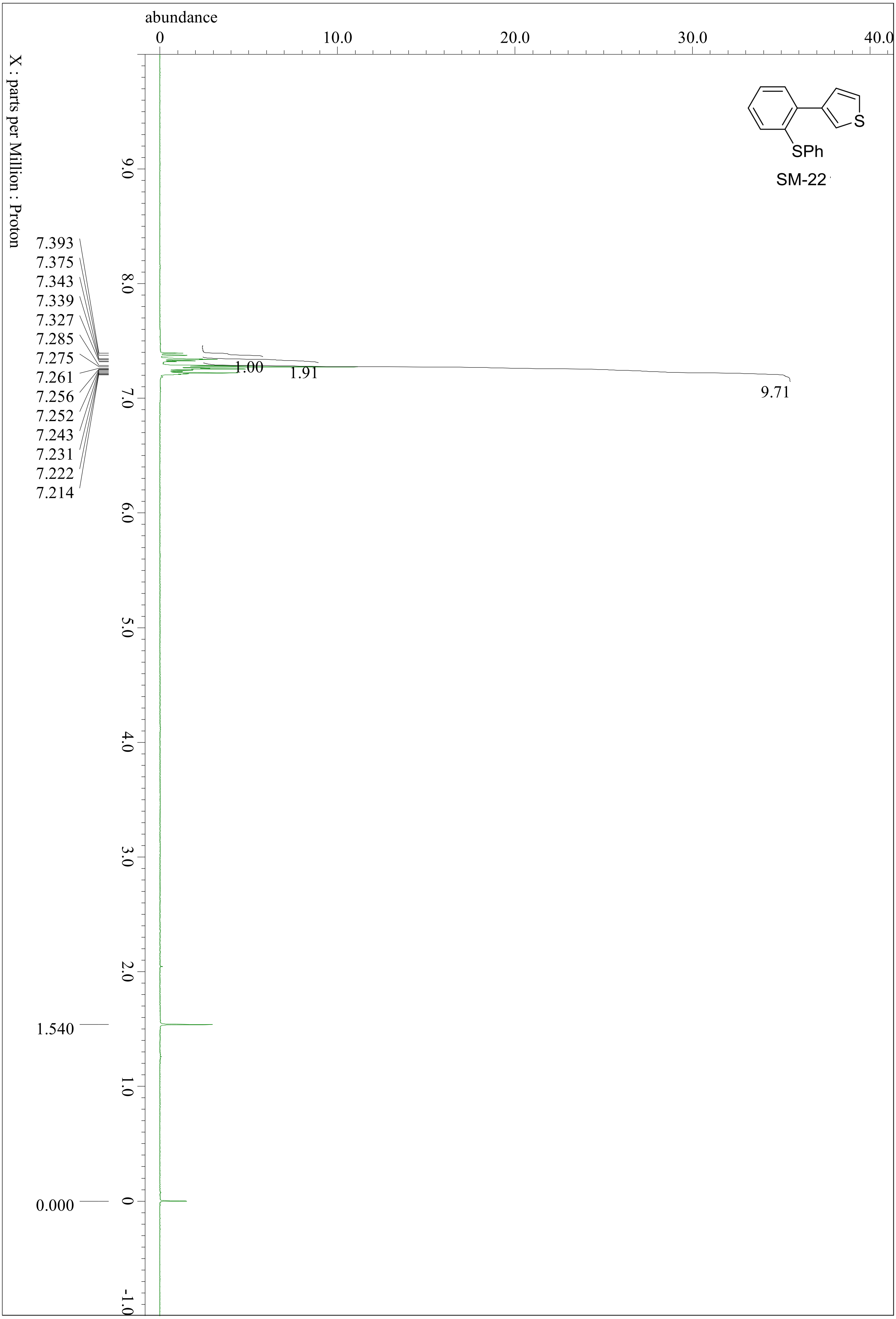
abundance
0
10.0
20.0
30.0
40.0
X : parts per Million : Proton
9.0
8.0
7.0
6.0
5.0
4.0
3.0
2.0
1.0
0
-1.0
7.393
7.375
7.343
7.339
7.327
7.285
7.275
7.261
7.256
7.252
7.243
7.231
7.222
7.214
1.540
0.000
1.00
1.91
9.71
S
SPh
SM-22

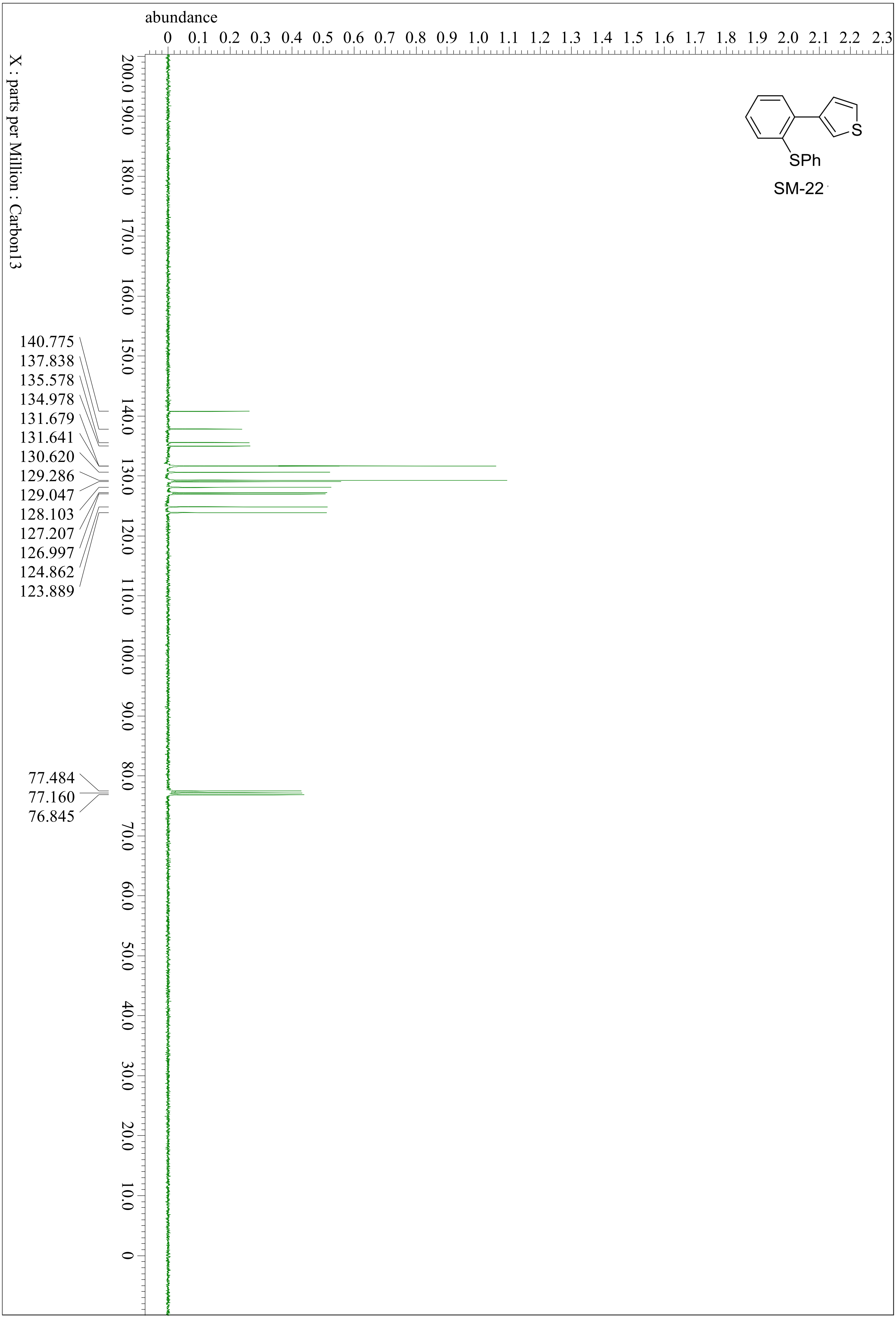

abundance
0 0.1 0.2 0.3 0.4 0.5 0.6 0.7 0.8 0.9 1.0 1.1 1.2 1.3 1.4 1.5 1.6 1.7 1.8 1.9 2.0 2.1 2.2 2.3
X : parts per Million : Carbon13
200.0 190.0 180.0 170.0 160.0 150.0 140.0 130.0 120.0 110.0 100.0 90.0 80.0 70.0 60.0 50.0 40.0 30.0 20.0 10.0 0
140.775
137.838
135.578
134.978
131.679
131.641
130.620
129.286
129.047
128.103
127.207
126.997
124.862
123.889
77.484
77.160
76.845
SPh
S
SM-22

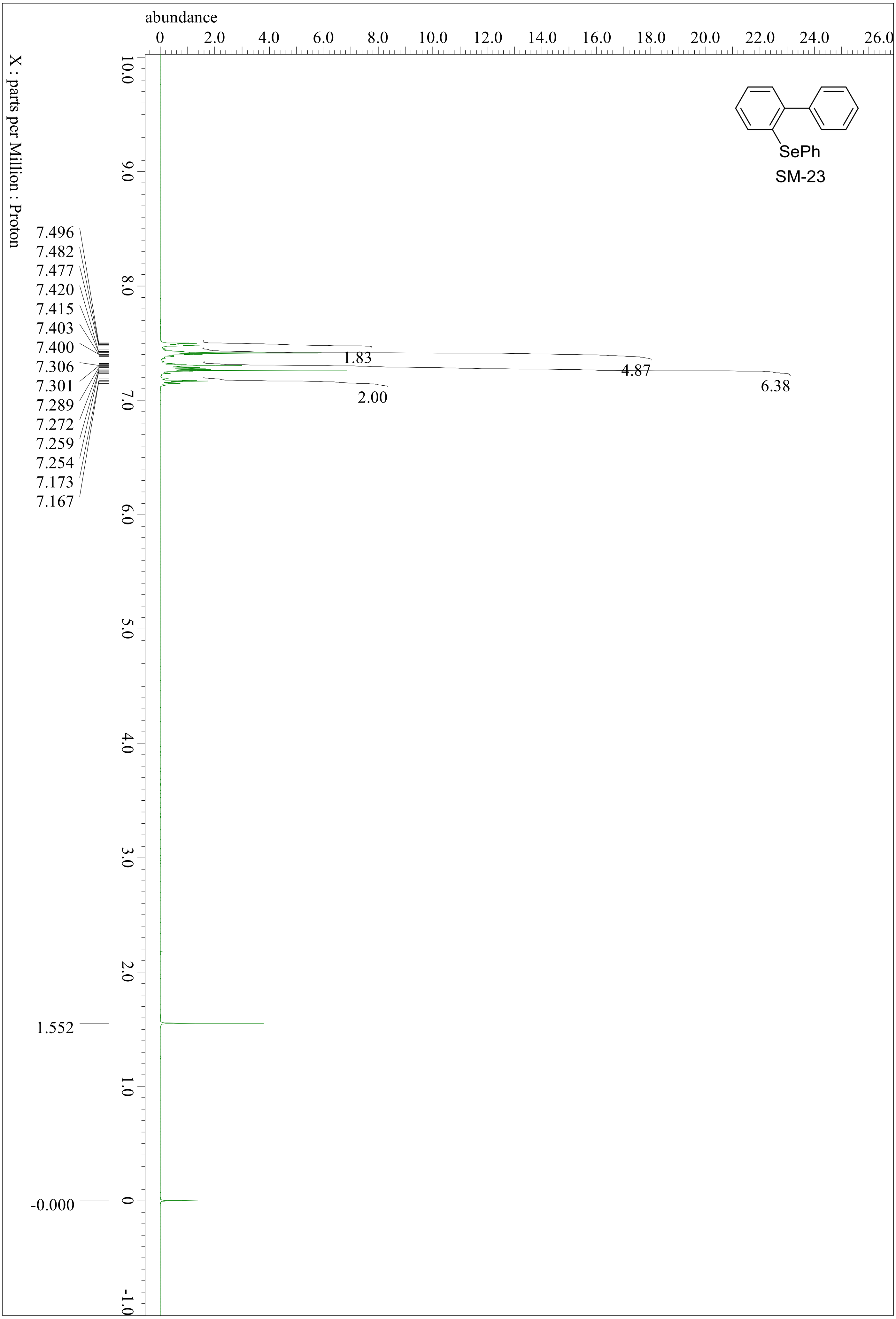
abundance
0
2.0
4.0
6.0
8.0
10.0
12.0
14.0
16.0
18.0
20.0
22.0
24.0
26.0
X : parts per Million : Proton
10.0
9.0
8.0
7.0
6.0
5.0
4.0
3.0
2.0
1.0
0
-1.0
7.496
7.482
7.477
7.420
7.415
7.403
7.400
7.306
7.301
7.289
7.272
7.259
7.254
7.173
7.167
1.552
-0.000
1.83
4.87
6.38
2.00
SePh
SM-23

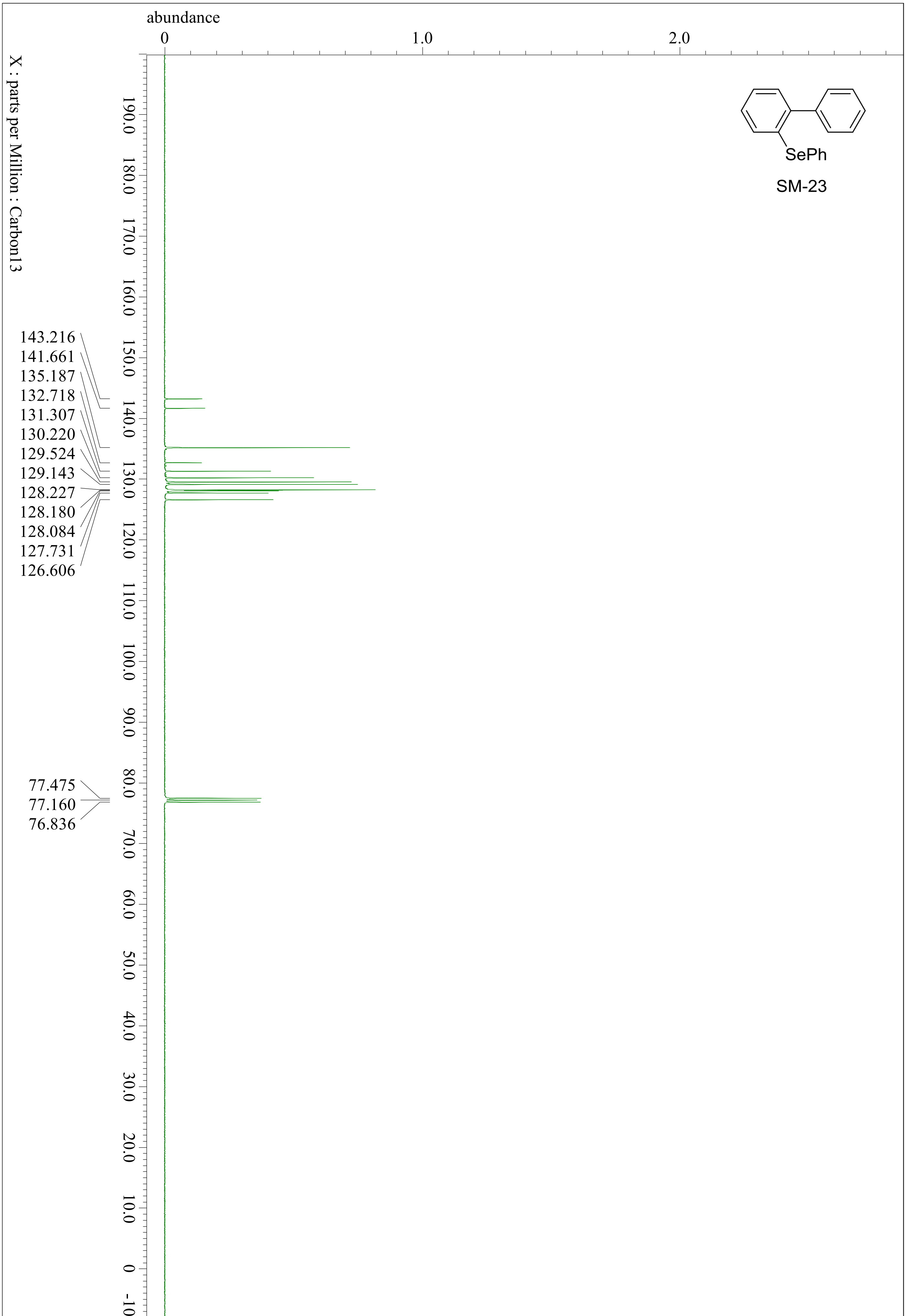

abundance
0
1.0
2.0
X : parts per Million : Carbon13
190.0
180.0
170.0
160.0
150.0
140.0
130.0
120.0
110.0
100.0
90.0
80.0
70.0
60.0
50.0
40.0
30.0
20.0
10.0
0
-10.0
143.216
141.661
135.187
132.718
131.307
130.220
129.524
129.143
128.227
128.180
128.084
127.731
126.606
77.475
77.160
76.836
SePh
SM-23

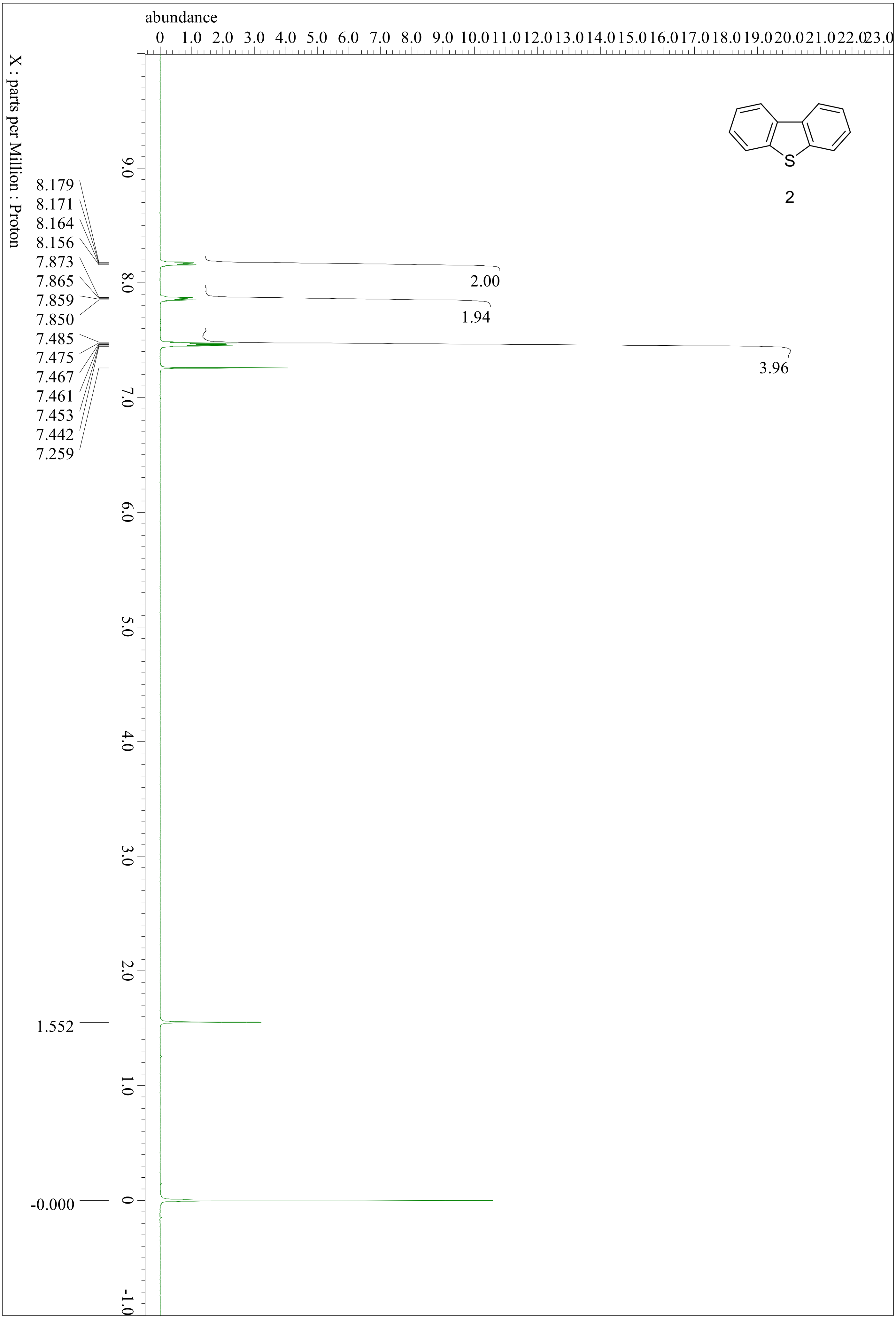

abundance
0 1.0 2.0 3.0 4.0 5.0 6.0 7.0 8.0 9.0 10.0 11.0 12.0 13.0 14.0 15.0 16.0 17.0 18.0 19.0 20.0 21.0 22.0 23.0
X : parts per Million : Proton
8.179
8.171
8.164
8.156
7.873
7.865
7.859
7.850
7.485
7.475
7.467
7.461
7.453
7.442
7.259
1.552
-0.000
9.0
8.0
7.0
6.0
5.0
4.0
3.0
2.0
1.0
0
-1.0
2.00
1.94
3.96
S
2

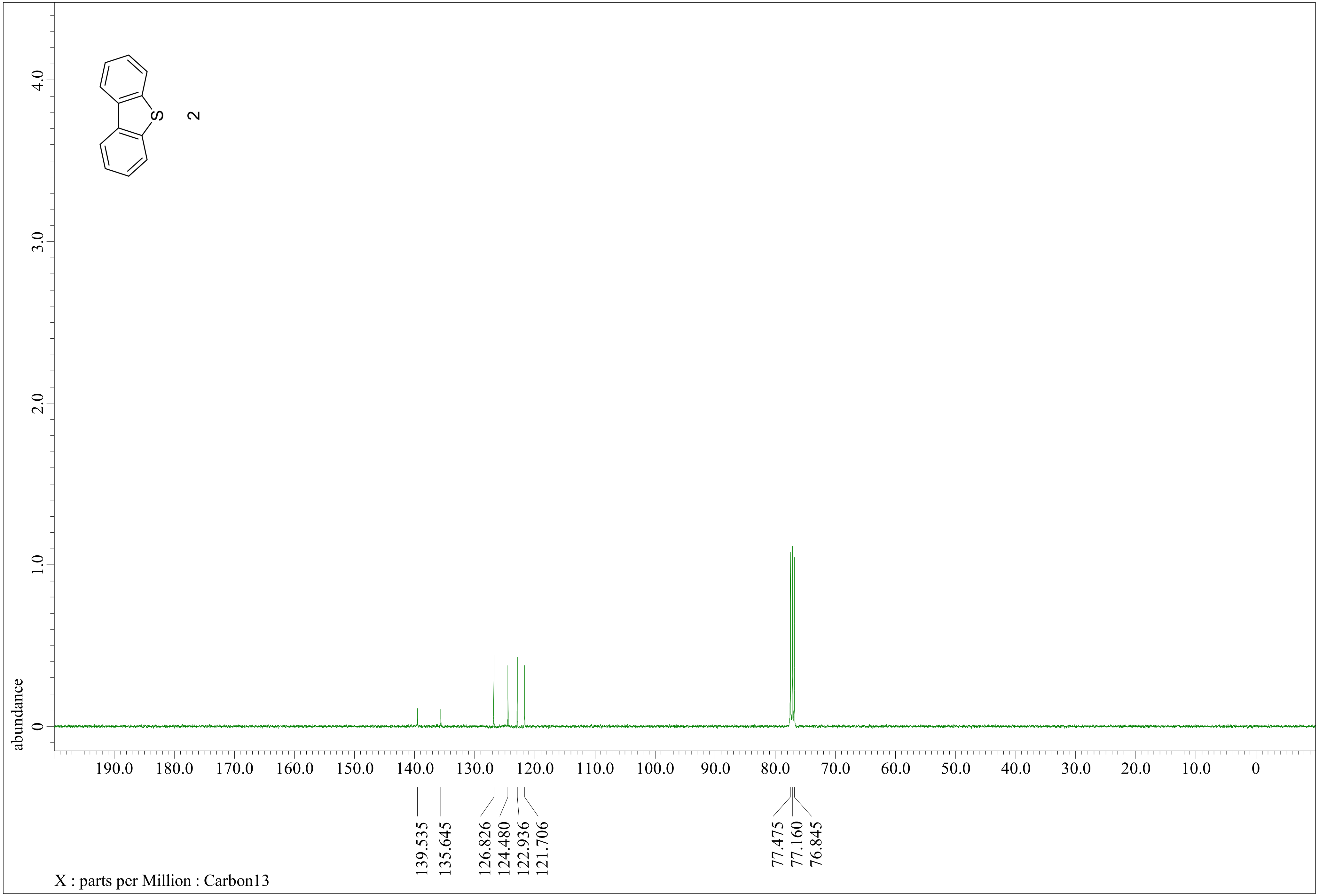

S
2
abundance
0
1.0
2.0
3.0
4.0
190.0
180.0
170.0
160.0
150.0
140.0
130.0
120.0
110.0
100.0
90.0
80.0
70.0
60.0
50.0
40.0
30.0
20.0
10.0
0
139.535
135.645
126.826
124.480
122.936
121.706
77.475
77.160
76.845
X : parts per Million : Carbon13

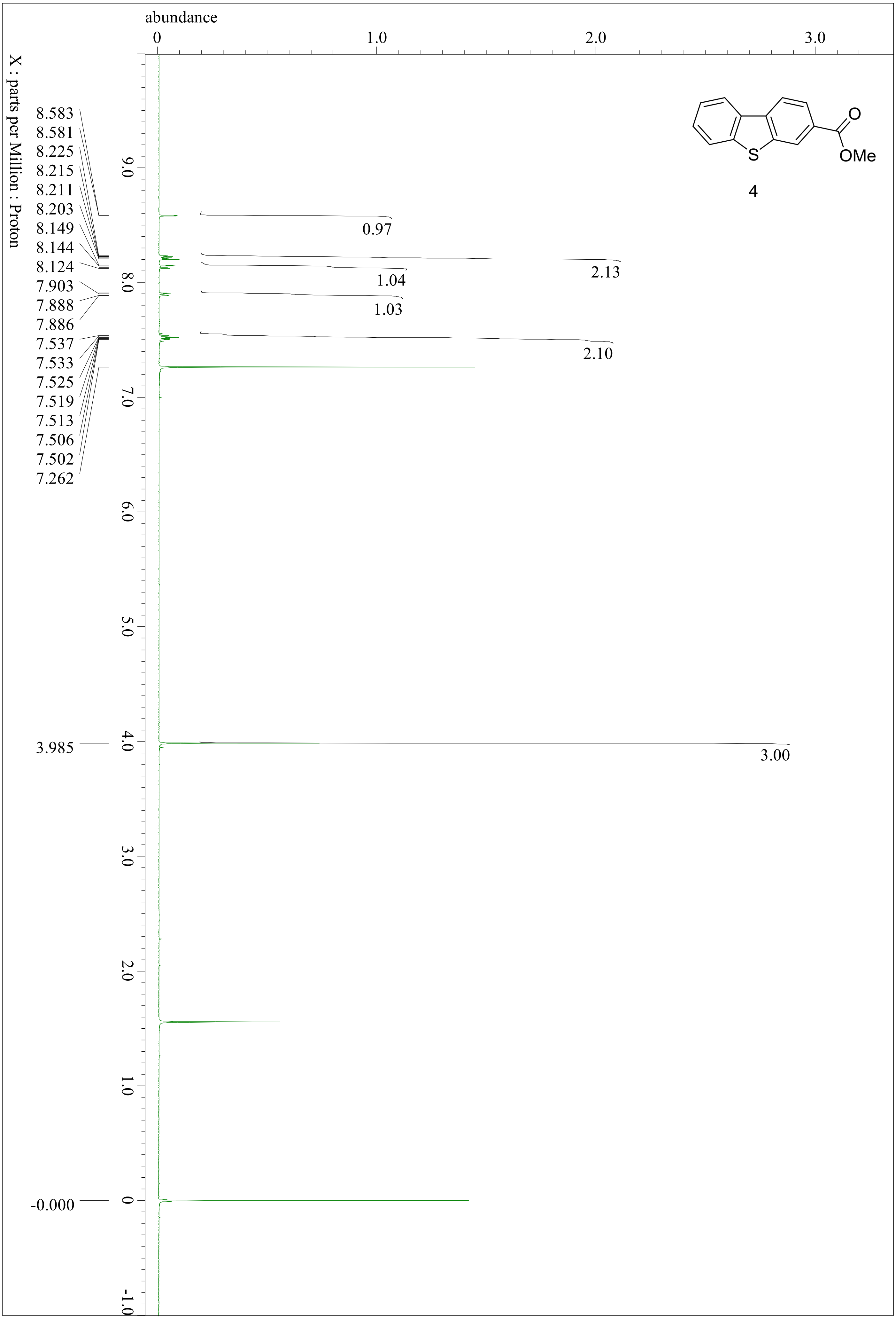
abundance
0
1.0
2.0
3.0
X : parts per Million : Proton
8.583
8.581
8.225
8.215
8.211
8.203
8.149
8.144
8.124
7.903
7.888
7.886
7.537
7.533
7.525
7.519
7.513
7.506
7.502
7.262
3.985
-0.000
9.0
8.0
7.0
6.0
5.0
4.0
3.0
2.0
1.0
0
-1.0
0.97
2.13
1.04
1.03
2.10
3.00
S
O
OMe
4

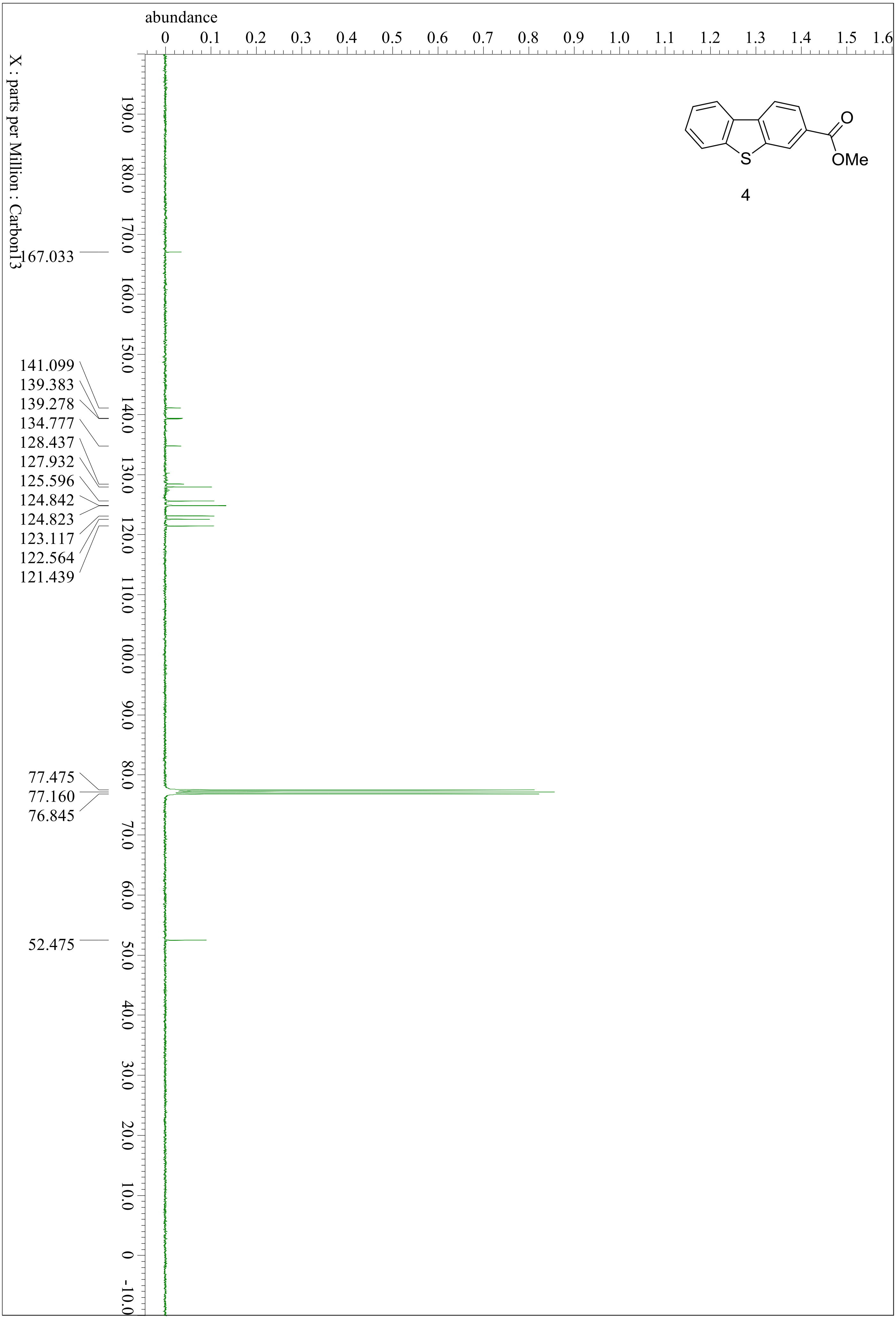

abundance
0 0.1 0.2 0.3 0.4 0.5 0.6 0.7 0.8 0.9 1.0 1.1 1.2 1.3 1.4 1.5 1.6
X : parts per Million : Carbon13
190.0 180.0 170.0 160.0 150.0 140.0 130.0 120.0 110.0 100.0 90.0 80.0 70.0 60.0 50.0 40.0 30.0 20.0 10.0 0 -10.0
167.033
141.099
139.383
139.278
134.777
128.437
127.932
125.596
124.842
124.823
123.117
122.564
121.439
77.475
77.160
76.845
52.475
O
S
OMe
4

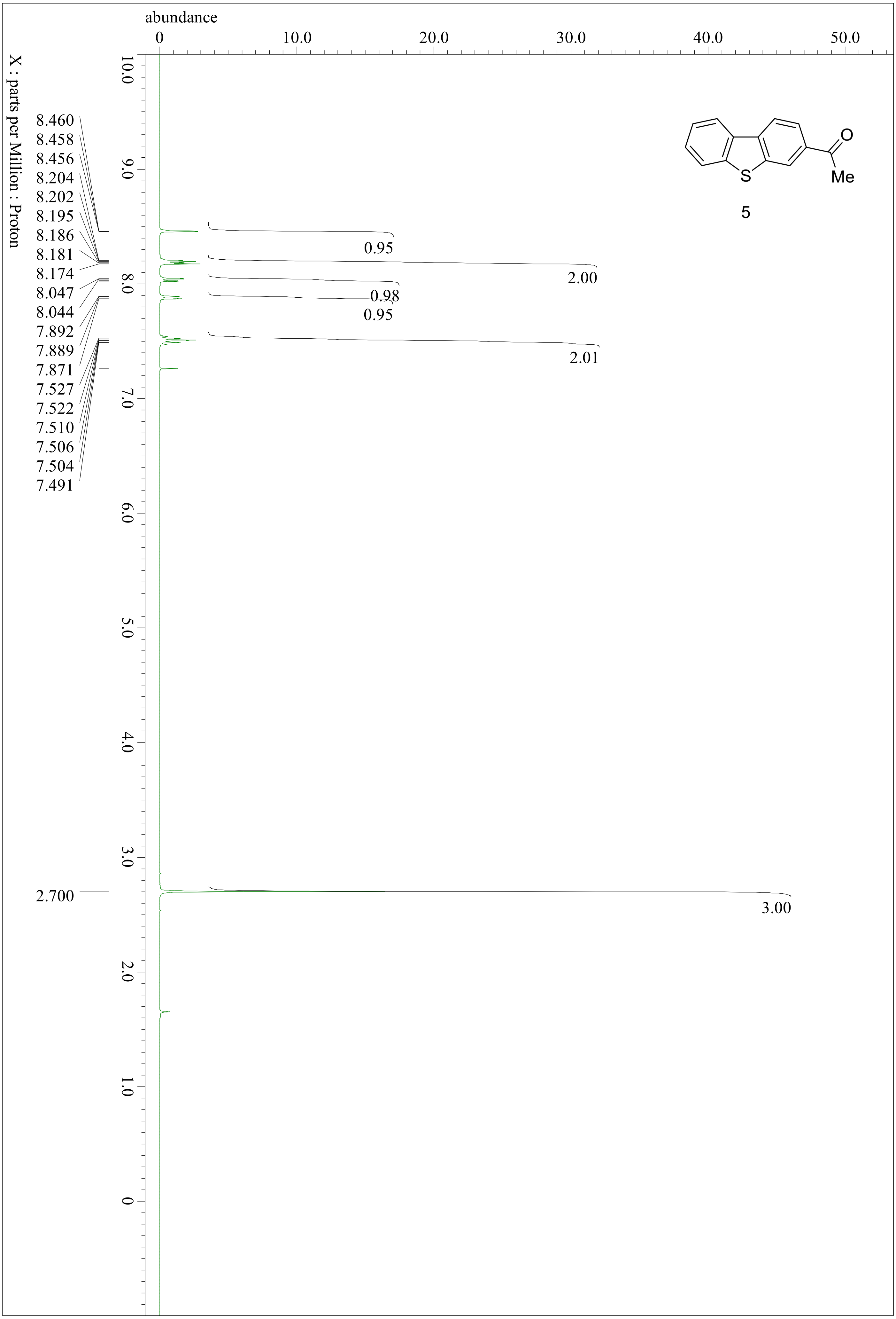
abundance
0
10.0
20.0
30.0
40.0
50.0
X : parts per Million : Proton
10.0
9.0
8.0
7.0
6.0
5.0
4.0
3.0
2.0
1.0
0
8.460
8.458
8.456
8.204
8.202
8.195
8.186
8.181
8.174
8.047
8.044
7.892
7.889
7.871
7.527
7.522
7.510
7.506
7.504
7.491
2.700
0.95
2.00
0.98
0.95
2.01
3.00
O
S
Me
5

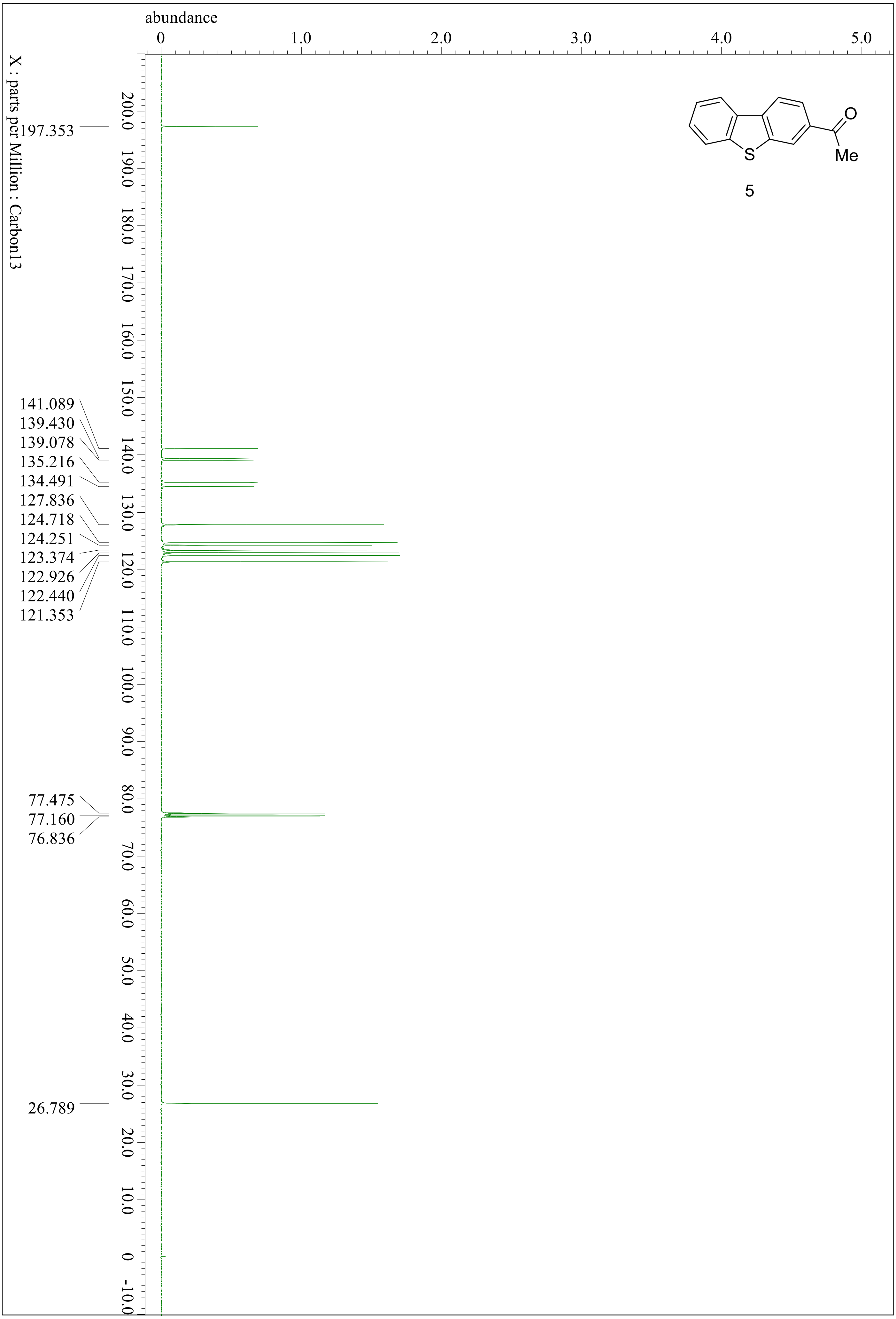
abundance
0
1.0
2.0
3.0
4.0
5.0
X : parts per Million : Carbon13
197.353
141.089
139.430
139.078
135.216
134.491
127.836
124.718
124.251
123.374
122.926
122.440
121.353
77.475
77.160
76.836
26.789
200.0
190.0
180.0
170.0
160.0
150.0
140.0
130.0
120.0
110.0
100.0
90.0
80.0
70.0
60.0
50.0
40.0
30.0
20.0
10.0
0
-10.0
O
S
Me
5

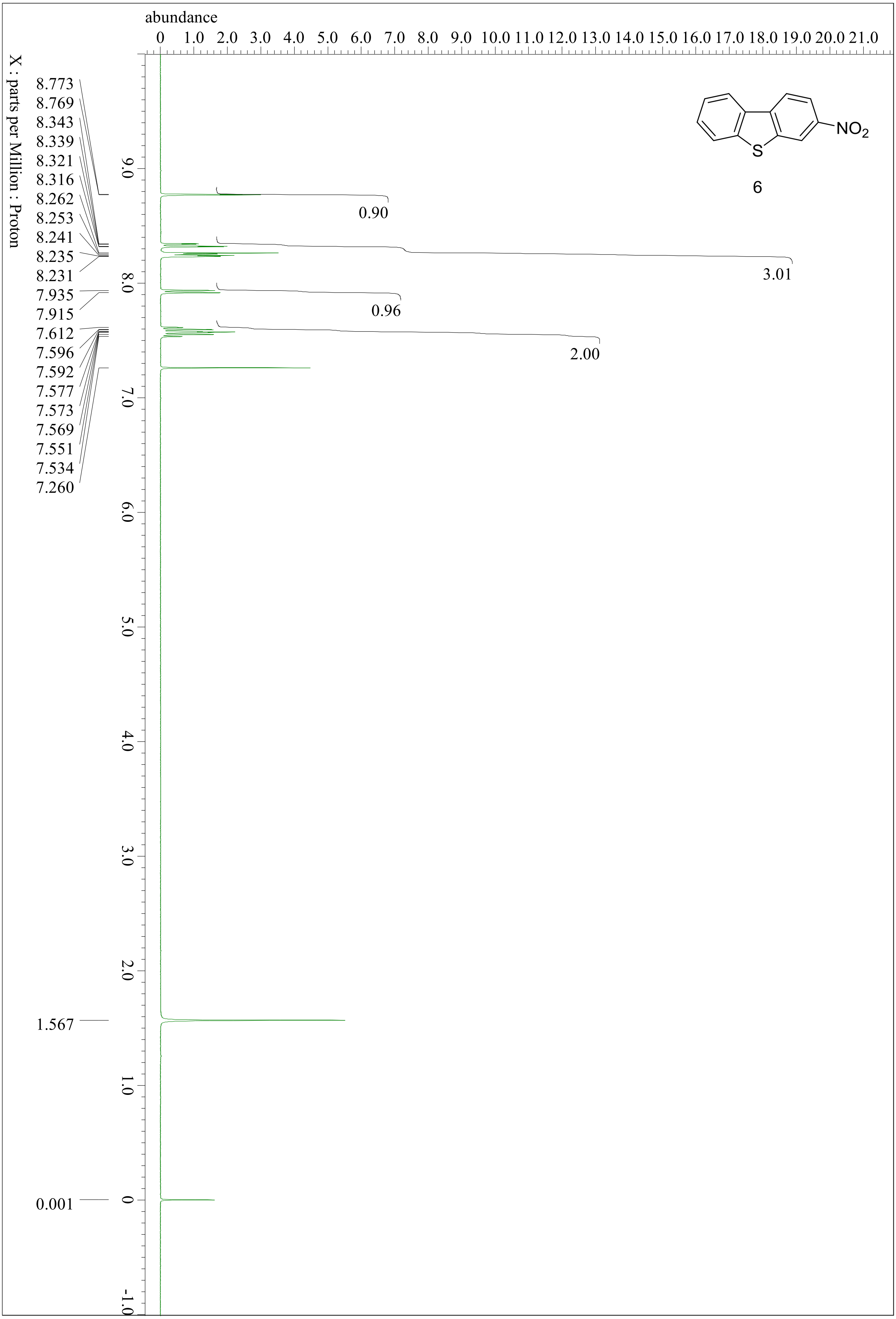
abundance
0 1.0 2.0 3.0 4.0 5.0 6.0 7.0 8.0 9.0 10.0 11.0 12.0 13.0 14.0 15.0 16.0 17.0 18.0 19.0 20.0 21.0
X : parts per Million : Proton
8.773
8.769
8.343
8.339
8.321
8.316
8.262
8.253
8.241
8.235
8.231
7.935
7.915
7.612
7.596
7.592
7.577
7.573
7.569
7.551
7.534
7.260
1.567
0.001
9.0
8.0
7.0
6.0
5.0
4.0
3.0
2.0
1.0
0
-1.0
0.90
3.01
0.96
2.00
NO2
S
6

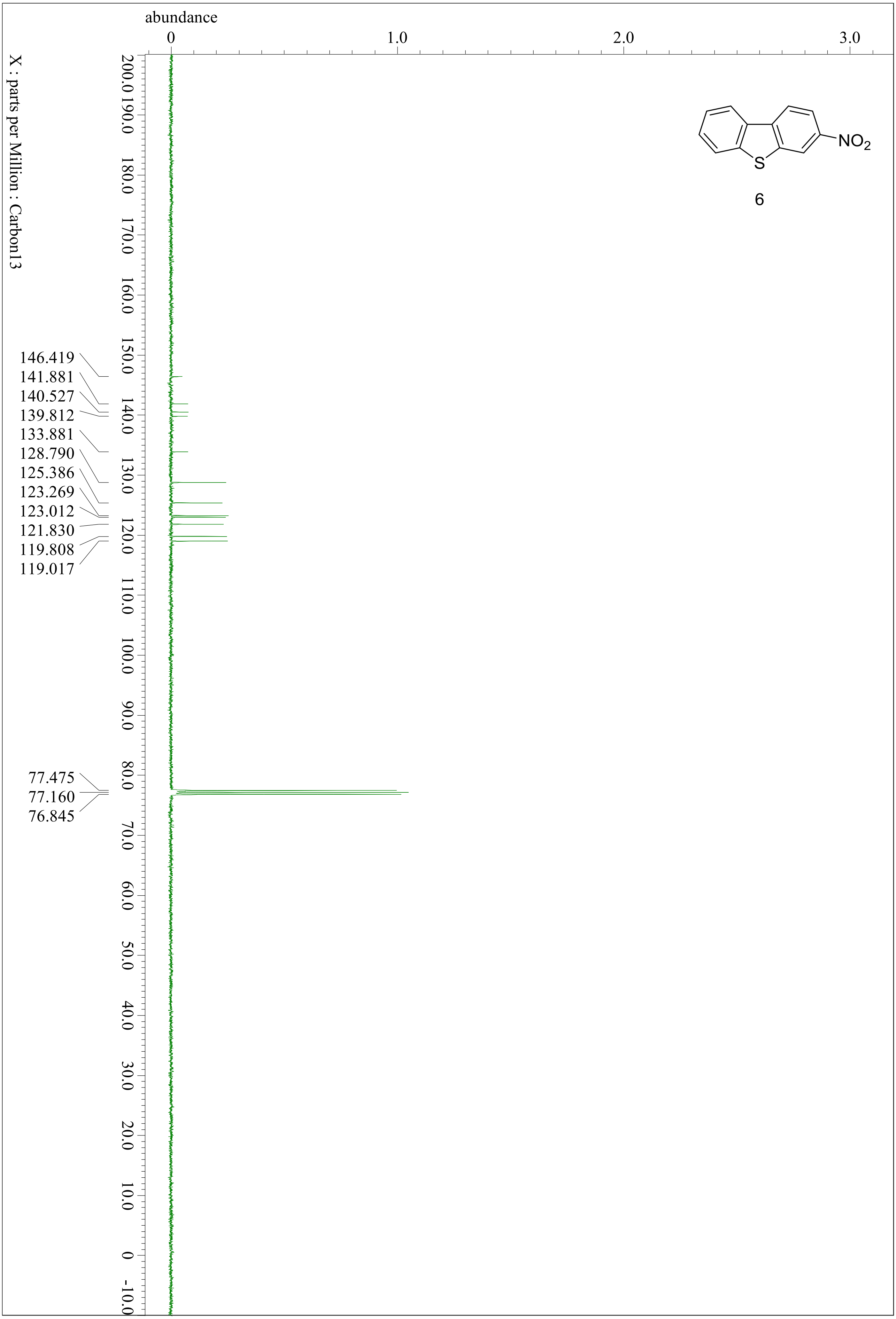
abundance
0
1.0
2.0
3.0
X : parts per Million : Carbon13
200.0
190.0
180.0
170.0
160.0
150.0
140.0
130.0
120.0
110.0
100.0
90.0
80.0
70.0
60.0
50.0
40.0
30.0
20.0
10.0
0
-10.0
146.419
141.881
140.527
139.812
133.881
128.790
125.386
123.269
123.012
121.830
119.808
119.017
77.475
77.160
76.845
S
NO2
6

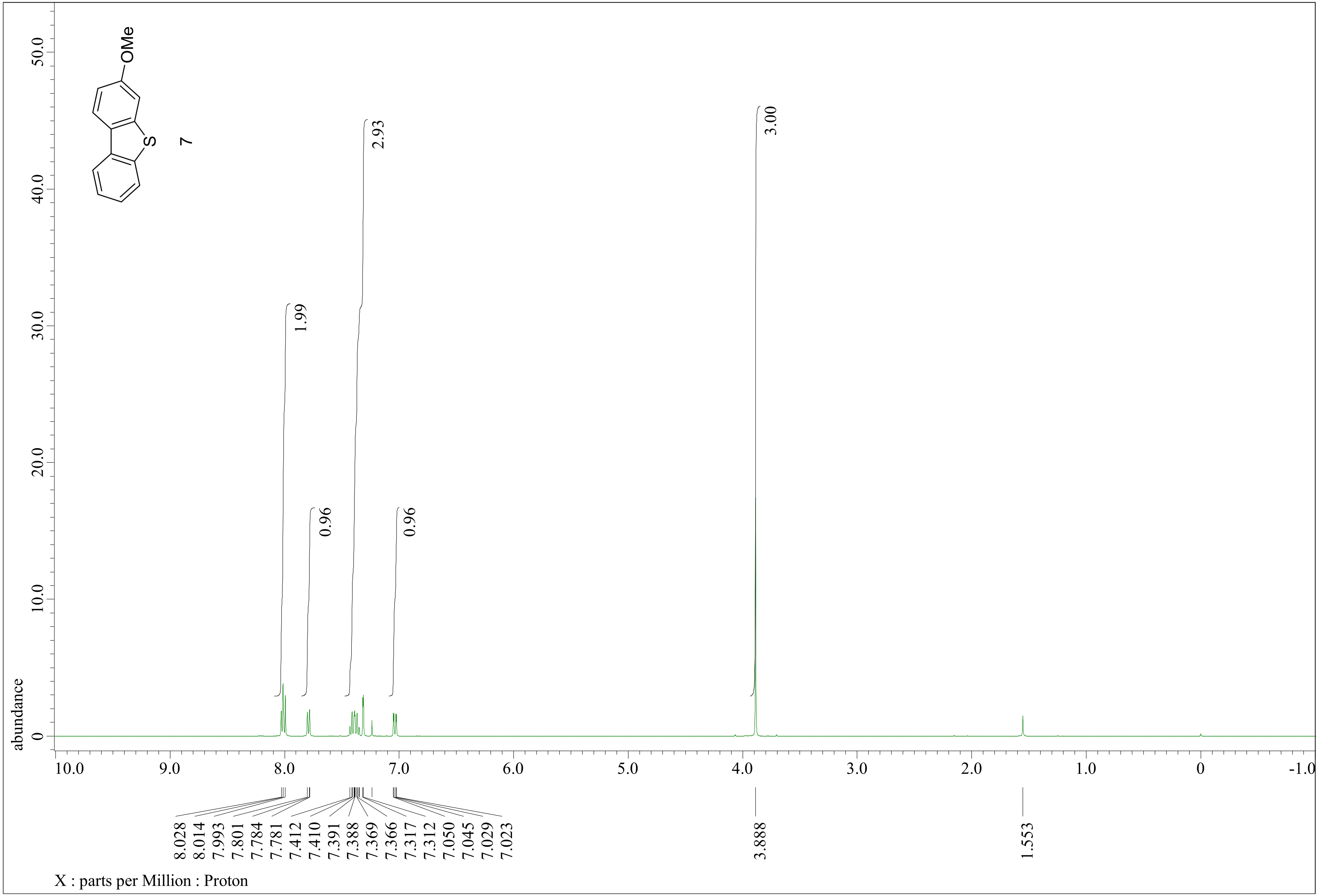
abundance
50.0
40.0
30.0
20.0
10.0
0
OMe
S
7
1.99
0.96
2.93
0.96
3.00
10.0
9.0
8.0
7.0
6.0
5.0
4.0
3.0
2.0
1.0
0
-1.0
8.028
8.014
7.993
7.801
7.784
7.781
7.412
7.410
7.391
7.388
7.369
7.366
7.317
7.312
7.050
7.045
7.029
7.023
3.888
1.553
X : parts per Million : Proton

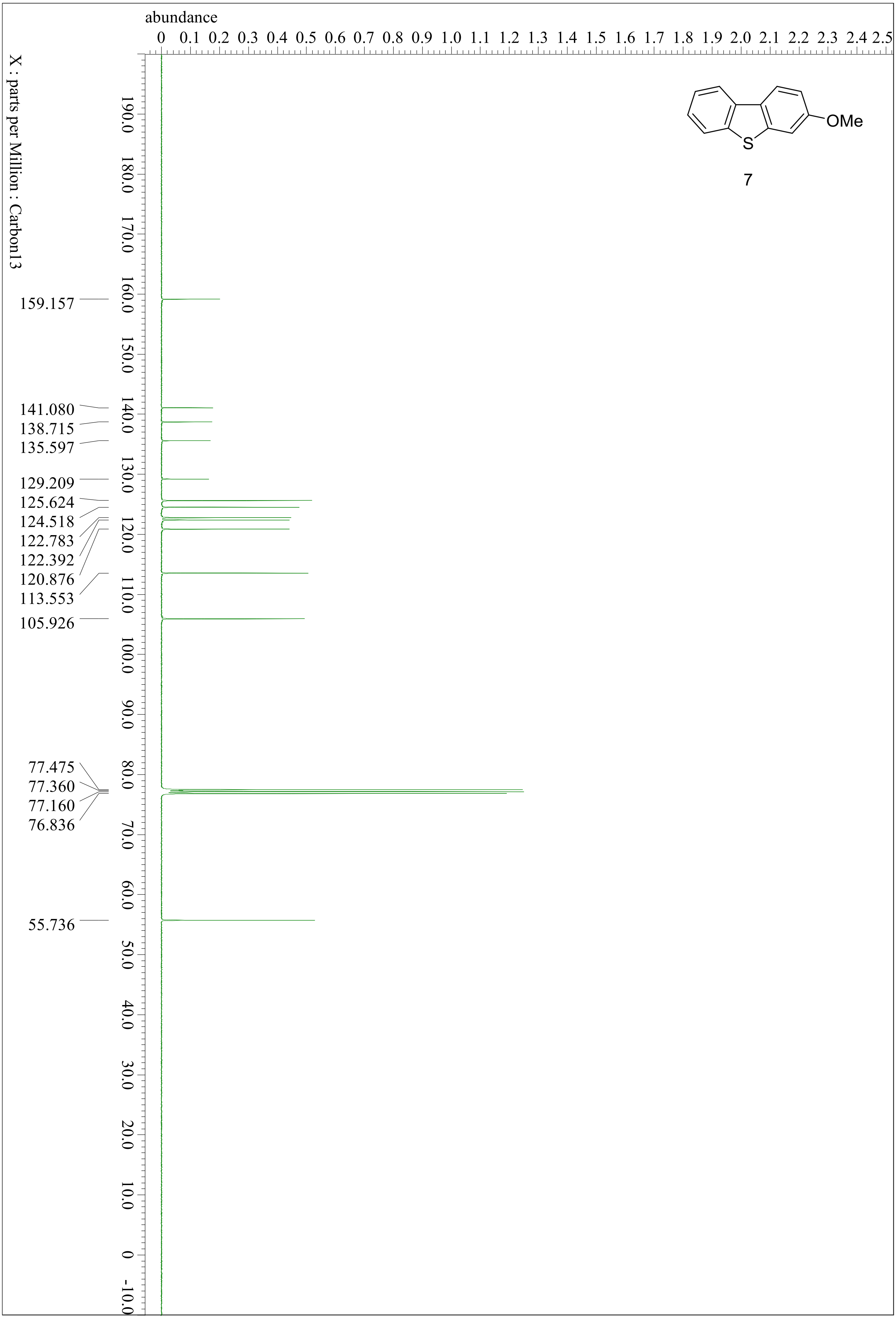
abundance
0 0.1 0.2 0.3 0.4 0.5 0.6 0.7 0.8 0.9 1.0 1.1 1.2 1.3 1.4 1.5 1.6 1.7 1.8 1.9 2.0 2.1 2.2 2.3 2.4 2.5
X : parts per Million : Carbon13
190.0 180.0 170.0 160.0 150.0 140.0 130.0 120.0 110.0 100.0 90.0 80.0 70.0 60.0 50.0 40.0 30.0 20.0 10.0 0 -10.0
159.157
141.080
138.715
135.597
129.209
125.624
124.518
122.783
122.392
120.876
113.553
105.926
77.475
77.360
77.160
76.836
55.736
OMe
S
7

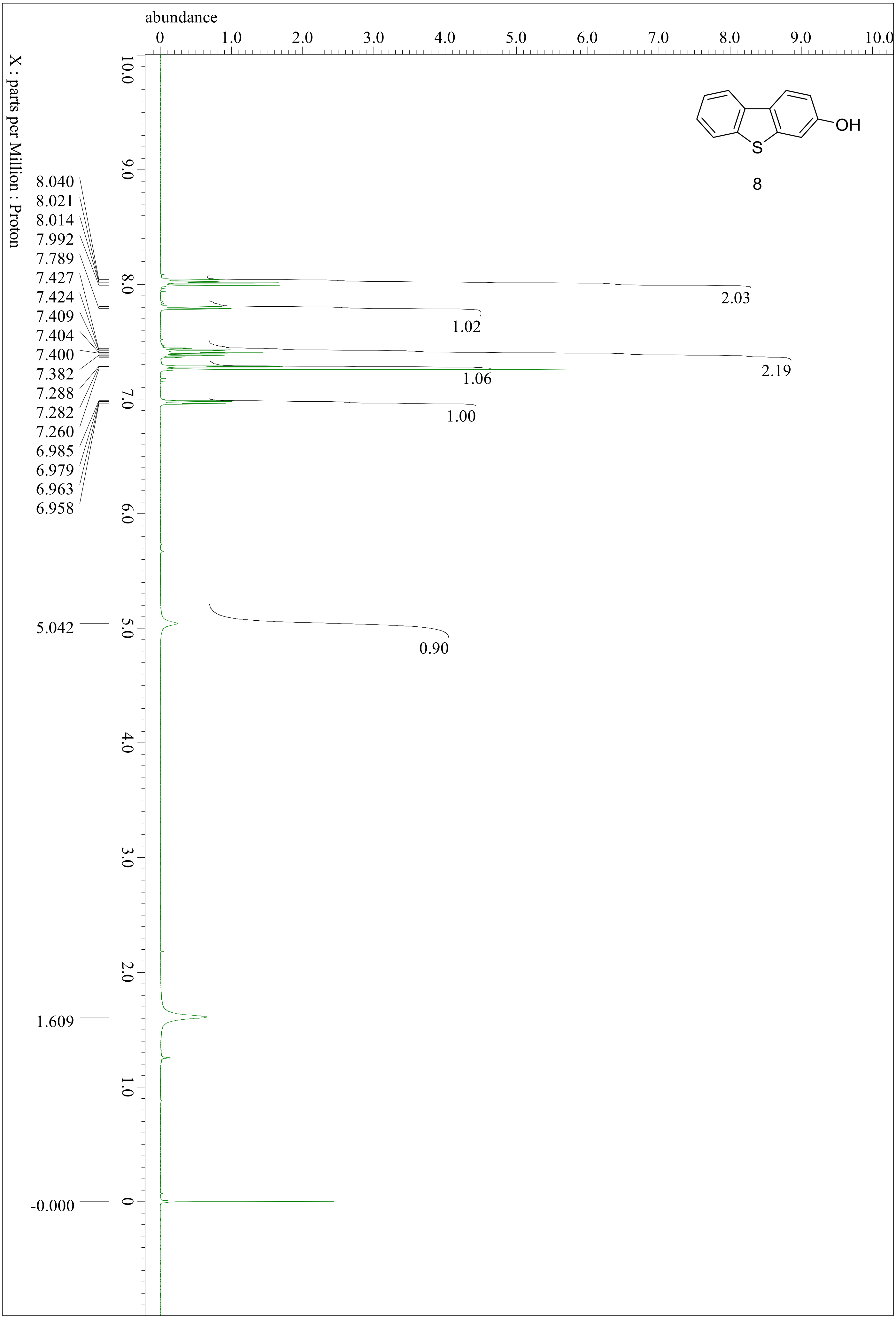
abundance
0 1.0 2.0 3.0 4.0 5.0 6.0 7.0 8.0 9.0 10.0
X : parts per Million : Proton
10.0 9.0 8.0 7.0 6.0 5.0 4.0 3.0 2.0 1.0 0
8.040
8.021
8.014
7.992
7.789
7.427
7.424
7.409
7.404
7.400
7.382
7.288
7.282
7.260
6.985
6.979
6.963
6.958
5.042
1.609
-0.000
2.03
1.02
2.19
1.06
1.00
0.90
OH
S
8

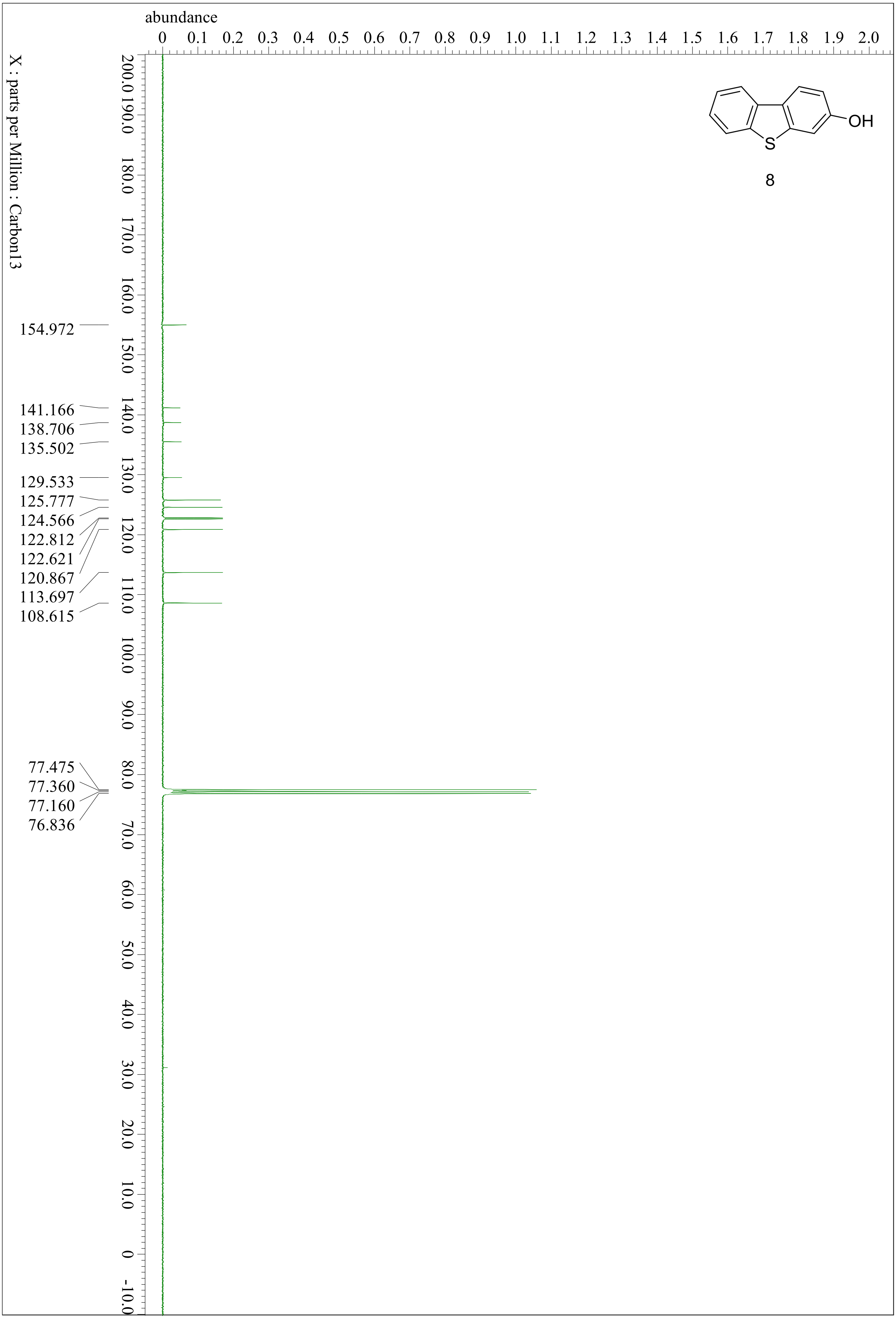
abundance
X : parts per Million : Carbon13
154.972
141.166
138.706
135.502
129.533
125.777
124.566
122.812
122.621
120.867
113.697
108.615
77.475
77.360
77.160
76.836
OH
S
8

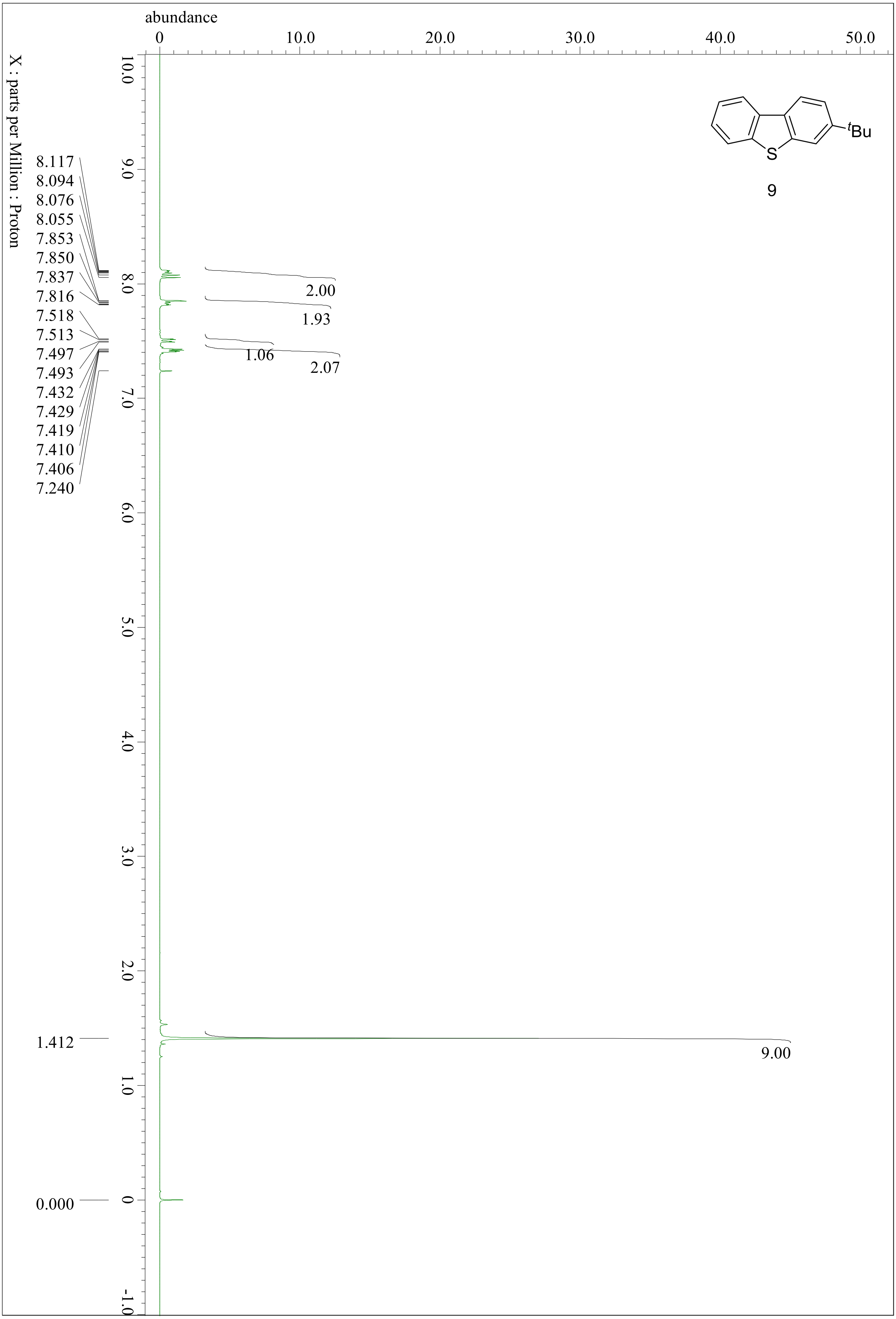

abundance
0
10.0
20.0
30.0
40.0
50.0
X : parts per Million : Proton
8.117
8.094
8.076
8.055
7.853
7.850
7.837
7.816
7.518
7.513
7.497
7.493
7.432
7.429
7.419
7.410
7.406
7.240
1.412
0.000
2.00
1.93
1.06
2.07
9.00
S
tBu
9

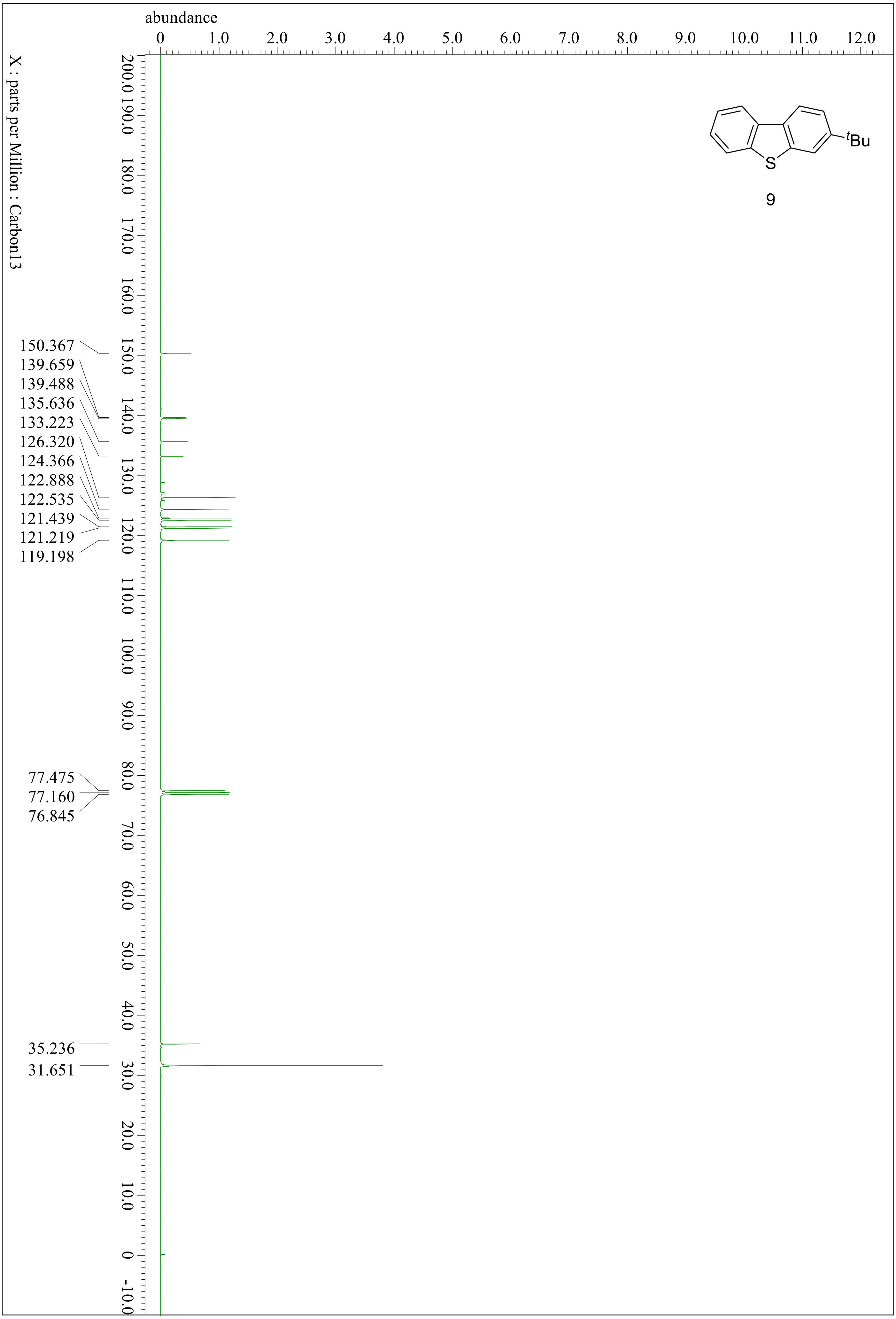
abundance
0 1.0 2.0 3.0 4.0 5.0 6.0 7.0 8.0 9.0 10.0 11.0 12.0
X : parts per Million : Carbon13
200.0 190.0 180.0 170.0 160.0 150.0 140.0 130.0 120.0 110.0 100.0 90.0 80.0 70.0 60.0 50.0 40.0 30.0 20.0 10.0 0 -10.0
150.367
139.659
139.488
135.636
133.223
126.320
124.366
122.888
122.535
121.439
121.219
119.198
77.475
77.160
76.845
35.236
31.651
S
tBu
9

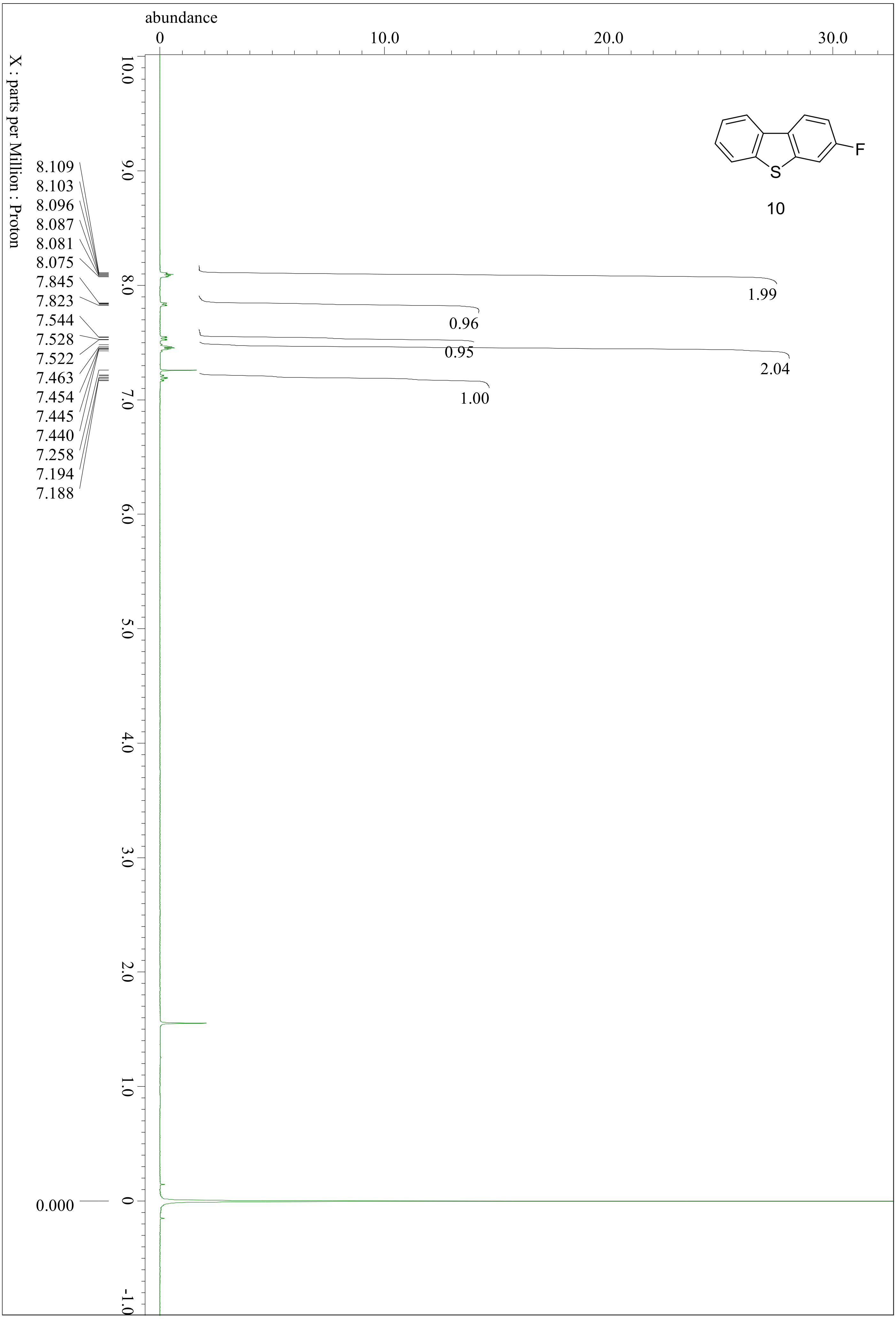

abundance
0
10.0
20.0
30.0
X : parts per Million : Proton
10.0
9.0
8.0
7.0
6.0
5.0
4.0
3.0
2.0
1.0
0
-1.0
8.109
8.103
8.096
8.087
8.081
8.075
7.845
7.823
7.544
7.528
7.522
7.463
7.454
7.445
7.440
7.258
7.194
7.188
0.000
1.99
0.96
0.95
2.04
1.00
F
S
10

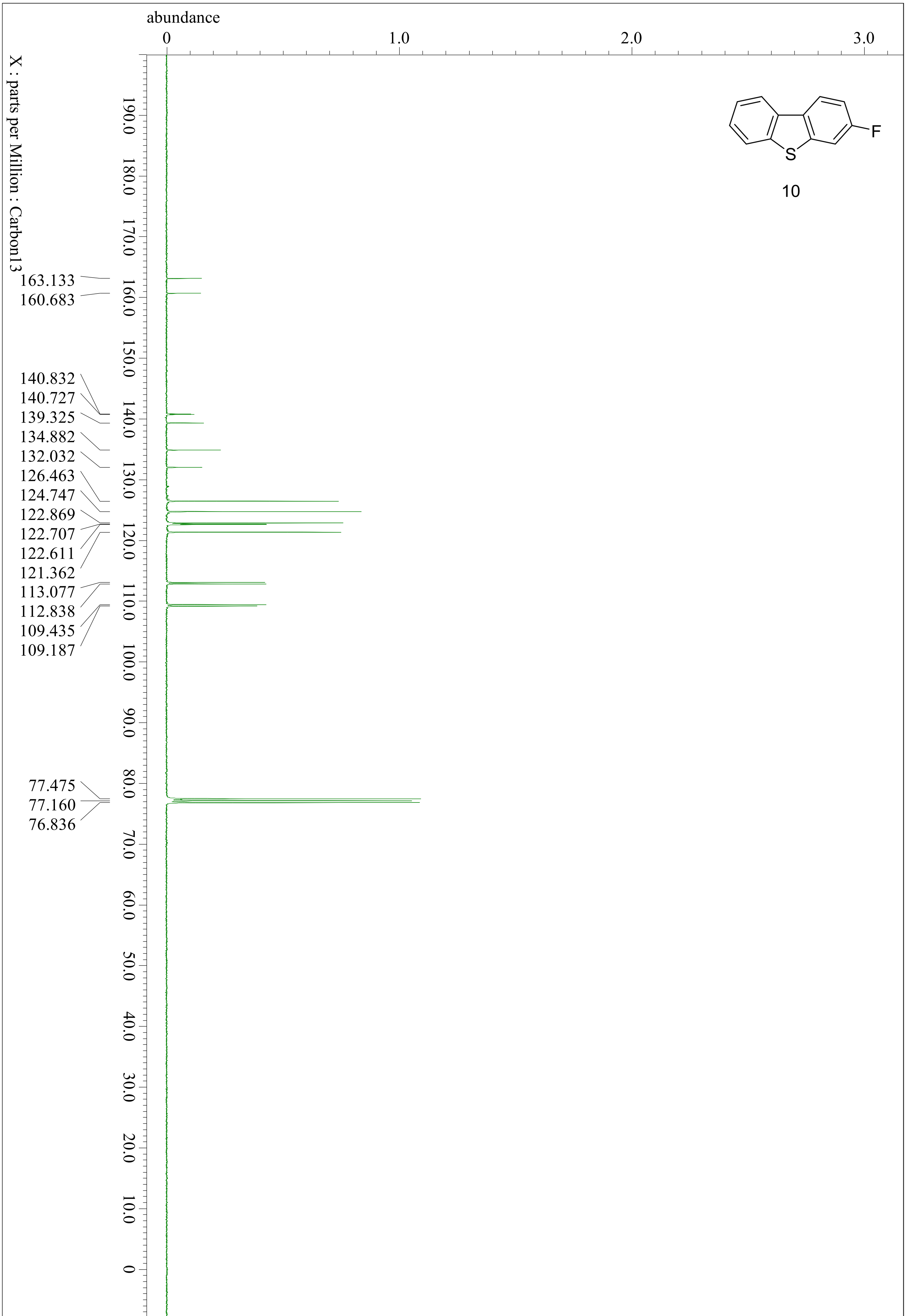

abundance
0
1.0
2.0
3.0
X : parts per Million : Carbon13
190.0
180.0
170.0
160.0
150.0
140.0
130.0
120.0
110.0
100.0
90.0
80.0
70.0
60.0
50.0
40.0
30.0
20.0
10.0
0
163.133
160.683
140.832
140.727
139.325
134.882
132.032
126.463
124.747
122.869
122.707
122.611
121.362
113.077
112.838
109.435
109.187
77.475
77.160
76.836
S
F
10

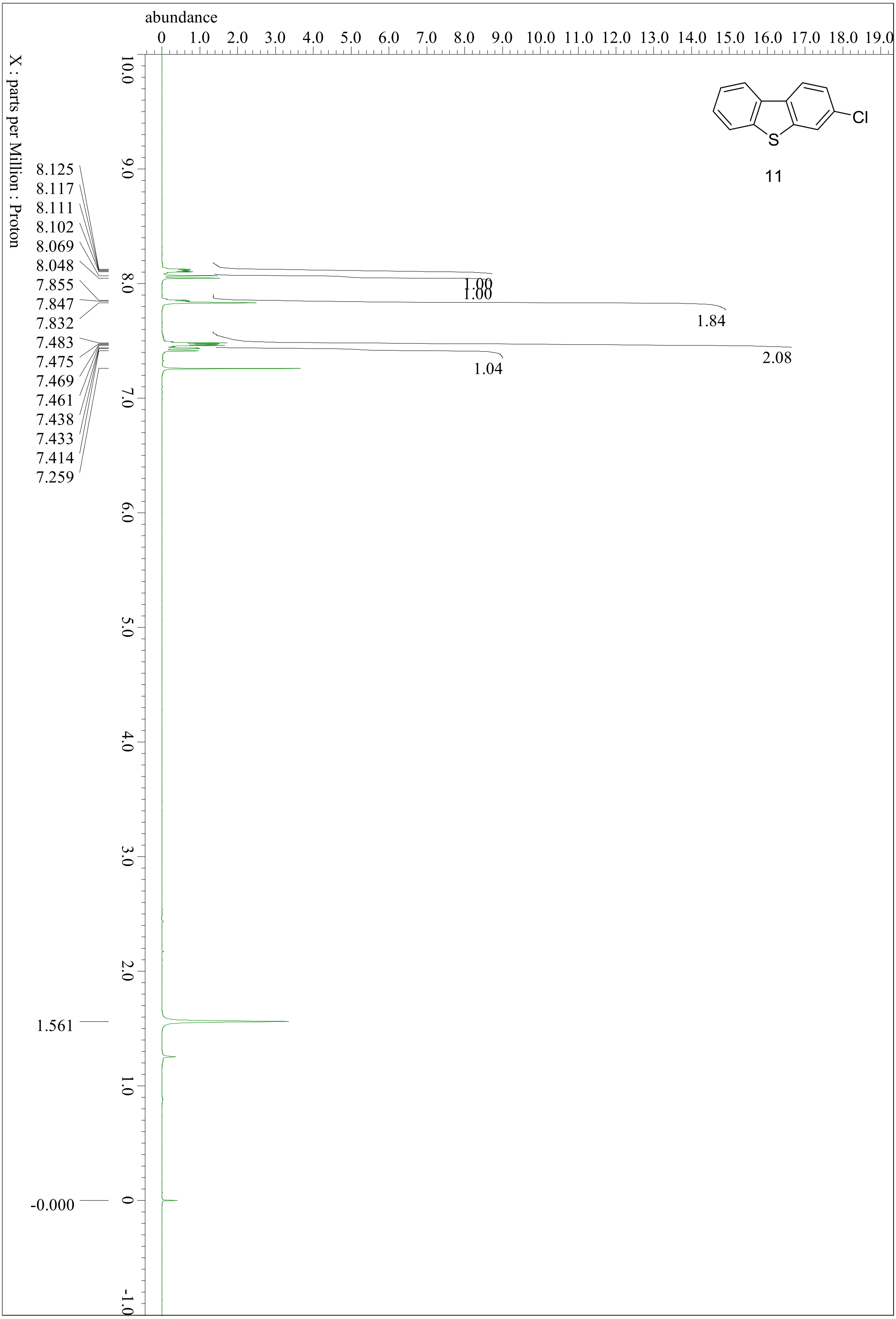
abundance
0 1.0 2.0 3.0 4.0 5.0 6.0 7.0 8.0 9.0 10.0 11.0 12.0 13.0 14.0 15.0 16.0 17.0 18.0 19.0
X : parts per Million : Proton
10.0 9.0 8.0 7.0 6.0 5.0 4.0 3.0 2.0 1.0 0 -1.0
8.125
8.117
8.111
8.102
8.069
8.048
7.855
7.847
7.832
7.483
7.475
7.469
7.461
7.438
7.433
7.414
7.259
1.561
-0.000
1.00
1.00
1.84
2.08
1.04
Cl
S
11

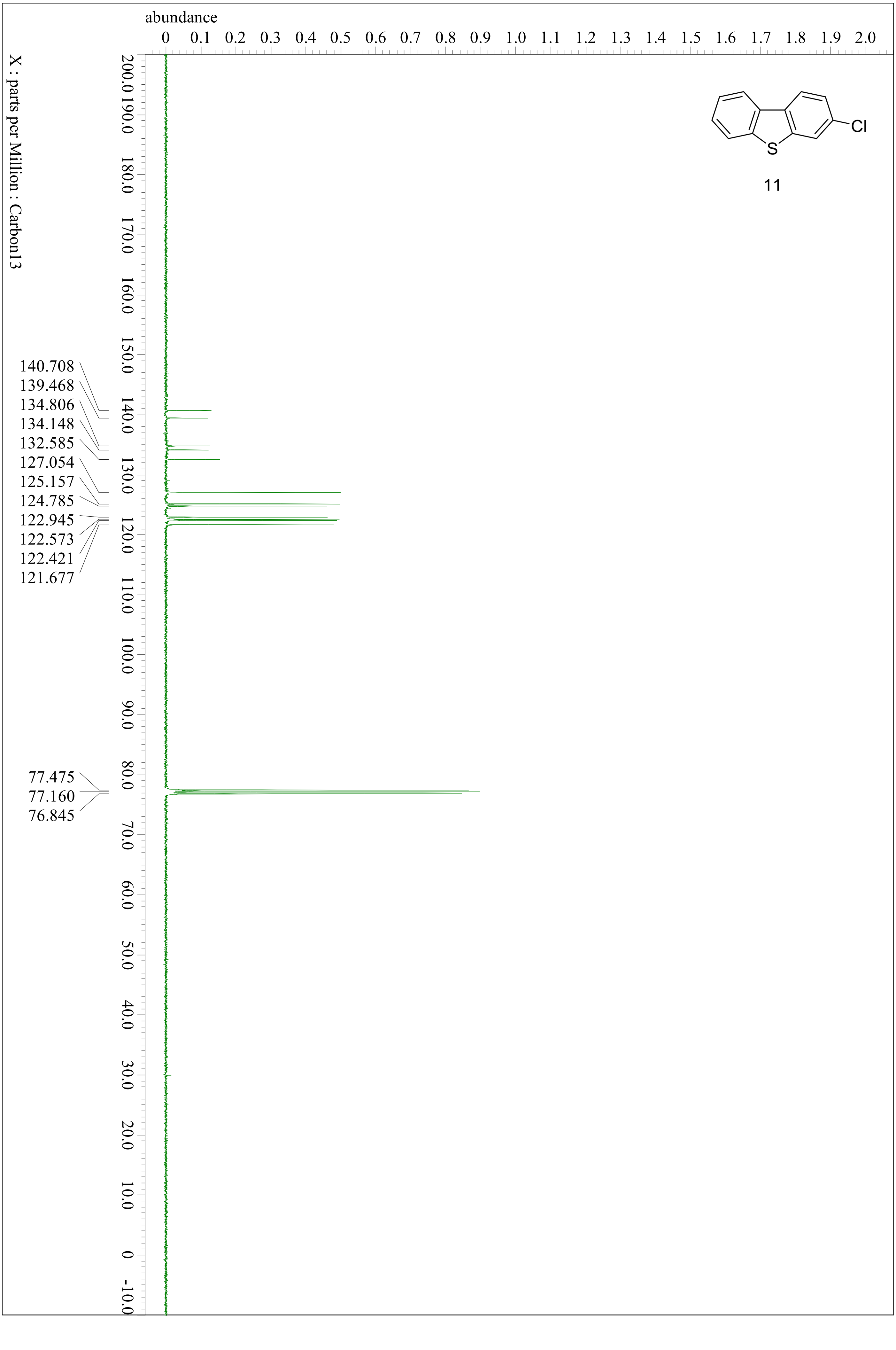

abundance
X : parts per Million : Carbon13
140.708
139.468
134.806
134.148
132.585
127.054
125.157
124.785
122.945
122.573
122.421
121.677
77.475
77.160
76.845
Cl
S
11

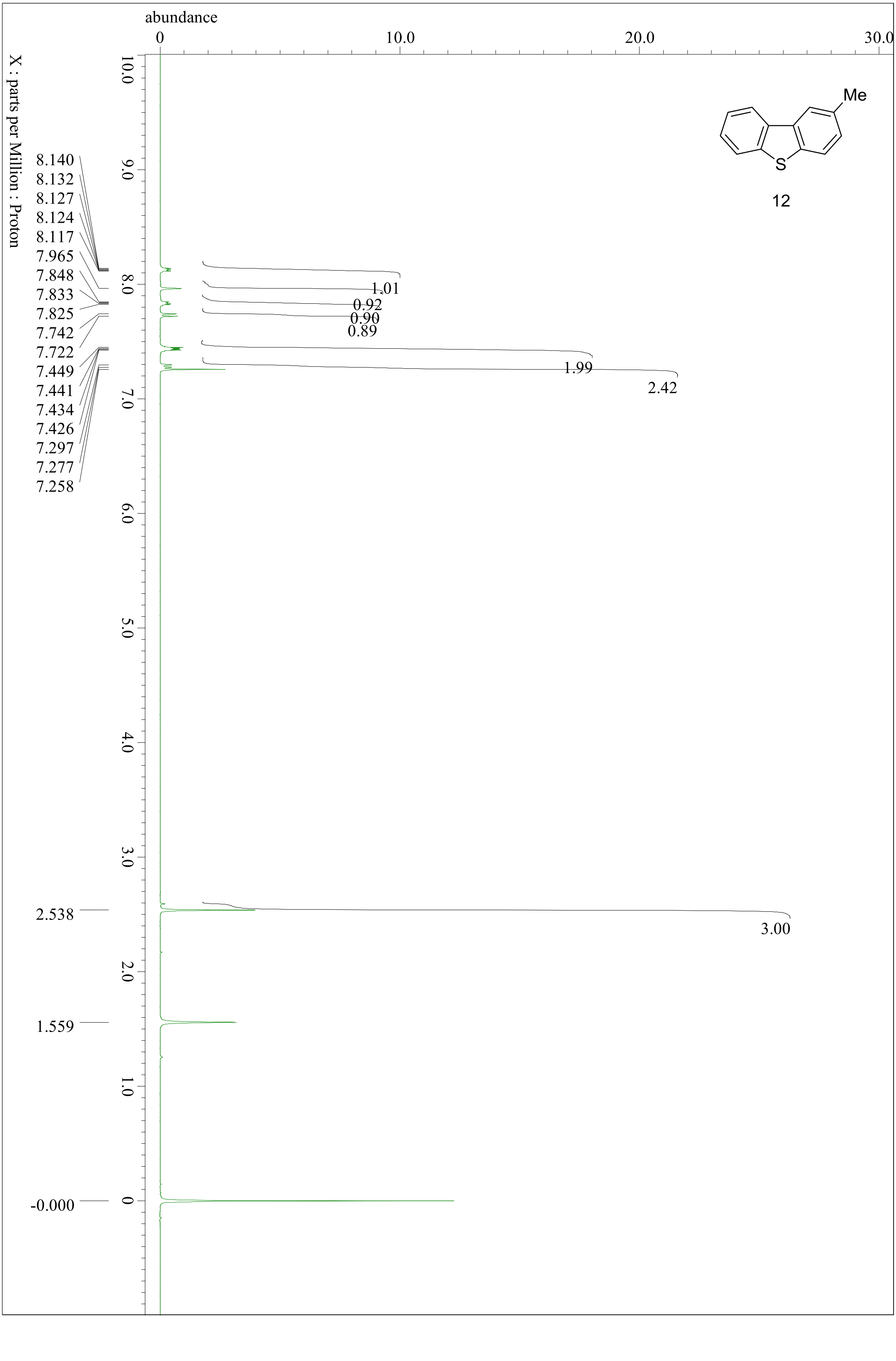

abundance
0
10.0
20.0
30.0
X : parts per Million : Proton
10.0
9.0
8.0
7.0
6.0
5.0
4.0
3.0
2.0
1.0
0
8.140
8.132
8.127
8.124
8.117
7.965
7.848
7.833
7.825
7.742
7.722
7.449
7.441
7.434
7.426
7.297
7.277
7.258
2.538
1.559
-0.000
1.01
0.92
0.90
0.89
1.99
2.42
3.00
Me
S
12

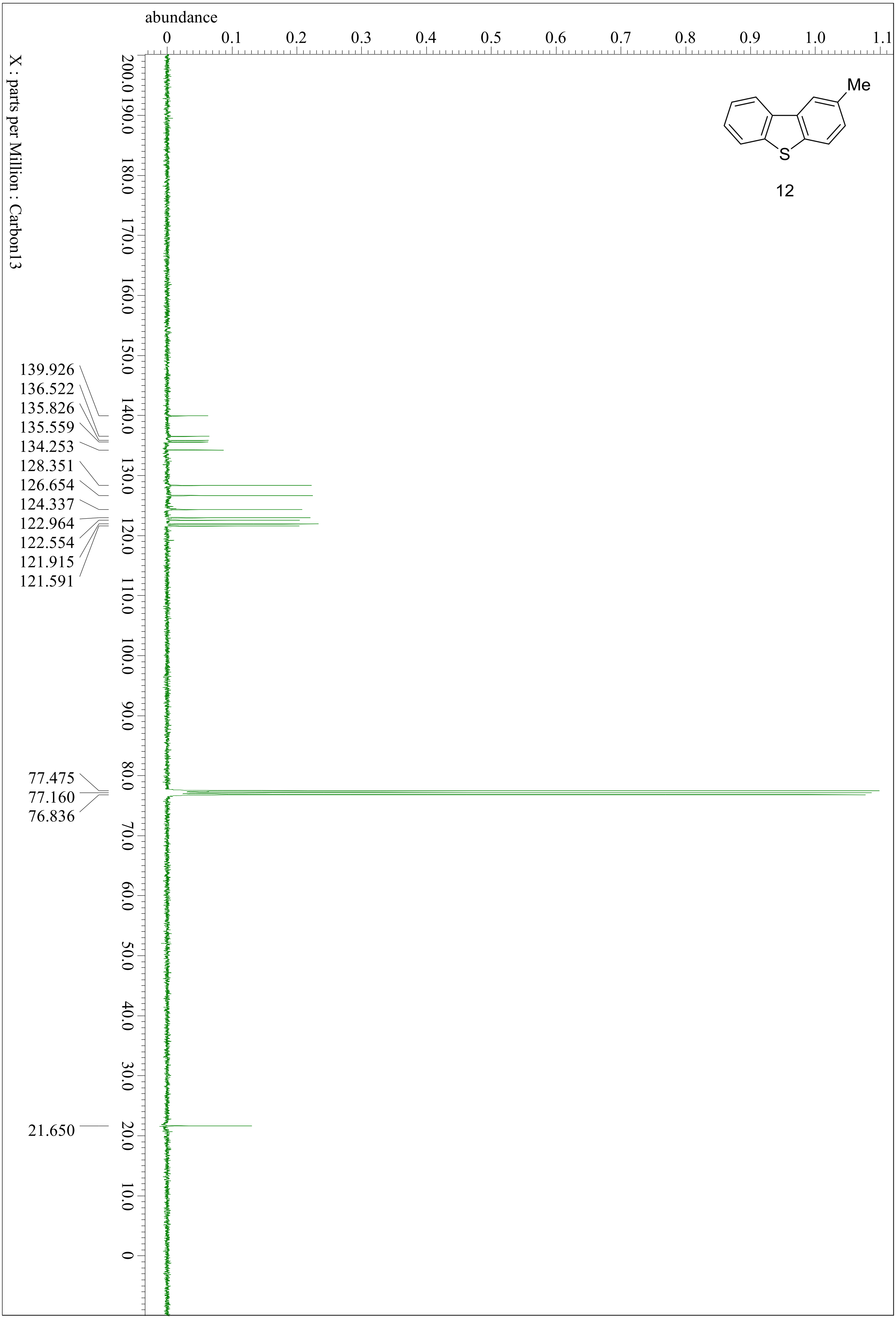

abundance
0 0.1 0.2 0.3 0.4 0.5 0.6 0.7 0.8 0.9 1.0 1.1
X : parts per Million : Carbon13
200.0 190.0 180.0 170.0 160.0 150.0 140.0 130.0 120.0 110.0 100.0 90.0 80.0 70.0 60.0 50.0 40.0 30.0 20.0 10.0 0
139.926
136.522
135.826
135.559
134.253
128.351
126.654
124.337
122.964
122.554
121.915
121.591
77.475
77.160
76.836
21.650
Me
S
12

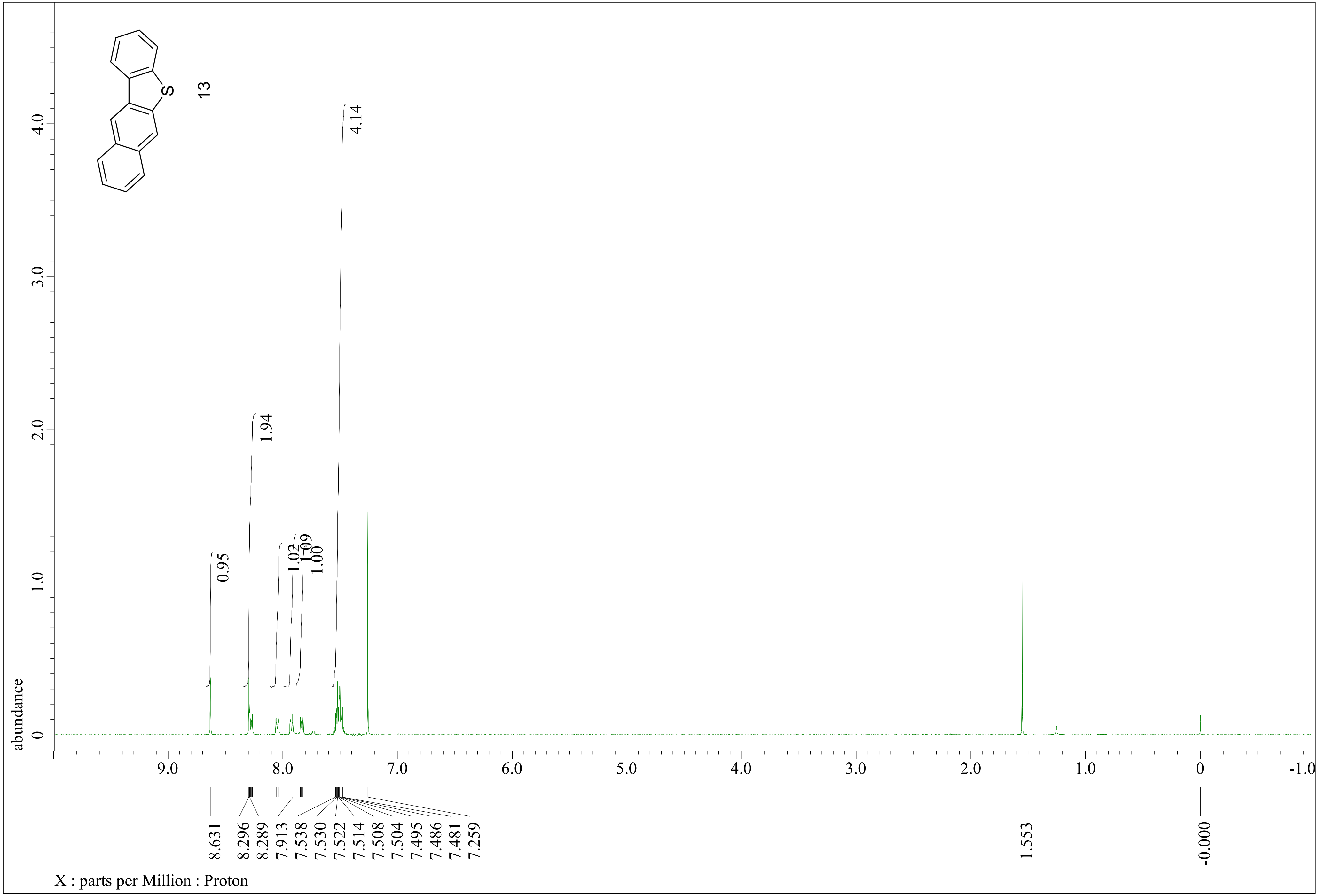
abundance
0
1.0
2.0
3.0
4.0
S
13
0.95
1.94
1.02
1.09
1.00
4.14
9.0
8.0
7.0
6.0
5.0
4.0
3.0
2.0
1.0
0
-1.0
8.631
8.296
8.289
7.913
7.538
7.530
7.522
7.514
7.508
7.504
7.495
7.486
7.481
7.259
1.553
-0.000
X : parts per Million : Proton

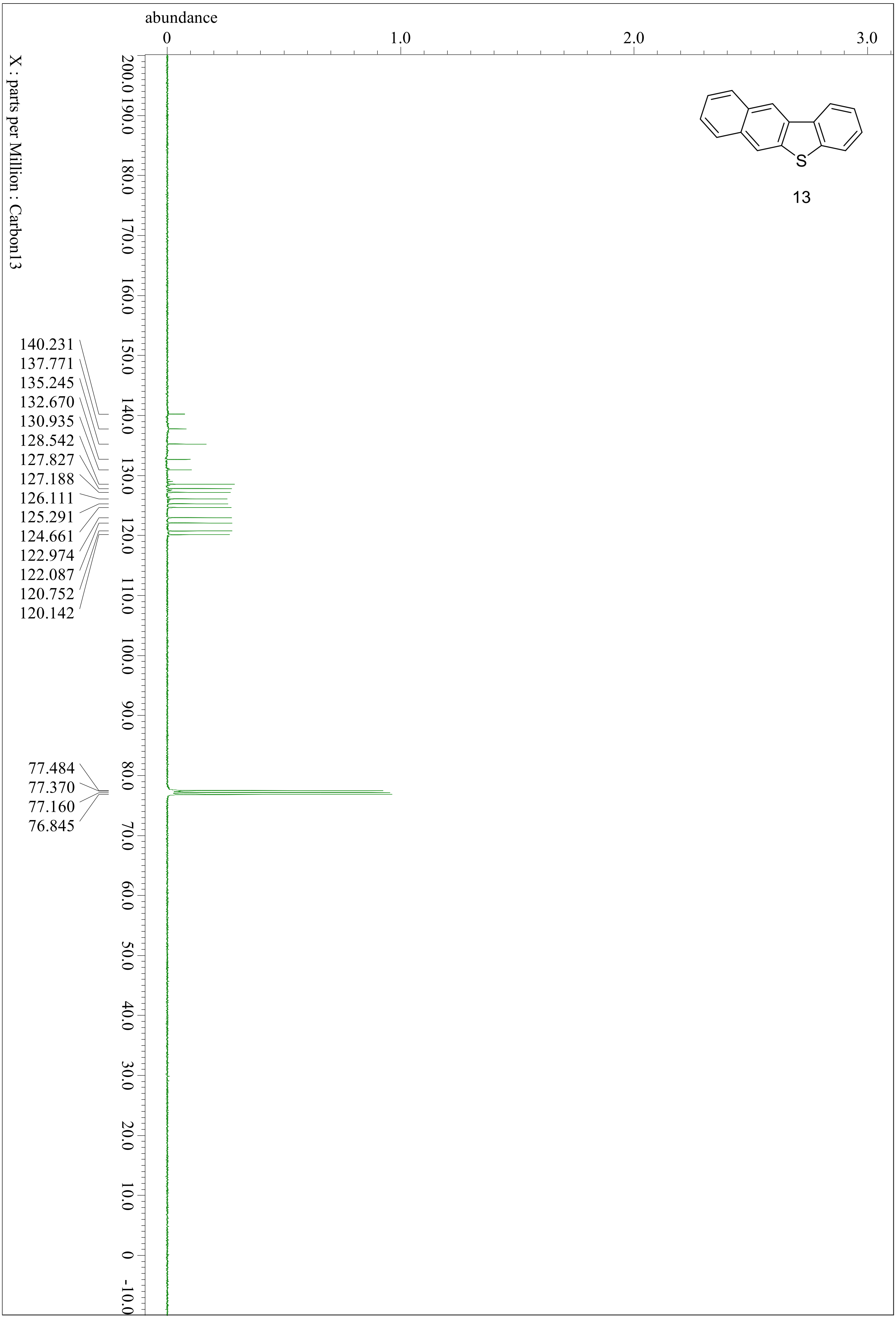

abundance
0
1.0
2.0
3.0
X : parts per Million : Carbon13
140.231
137.771
135.245
132.670
130.935
128.542
127.827
127.188
126.111
125.291
124.661
122.974
122.087
120.752
120.142
77.484
77.370
77.160
76.845
200.0
190.0
180.0
170.0
160.0
150.0
140.0
130.0
120.0
110.0
100.0
90.0
80.0
70.0
60.0
50.0
40.0
30.0
20.0
10.0
0
-10.0
S
13

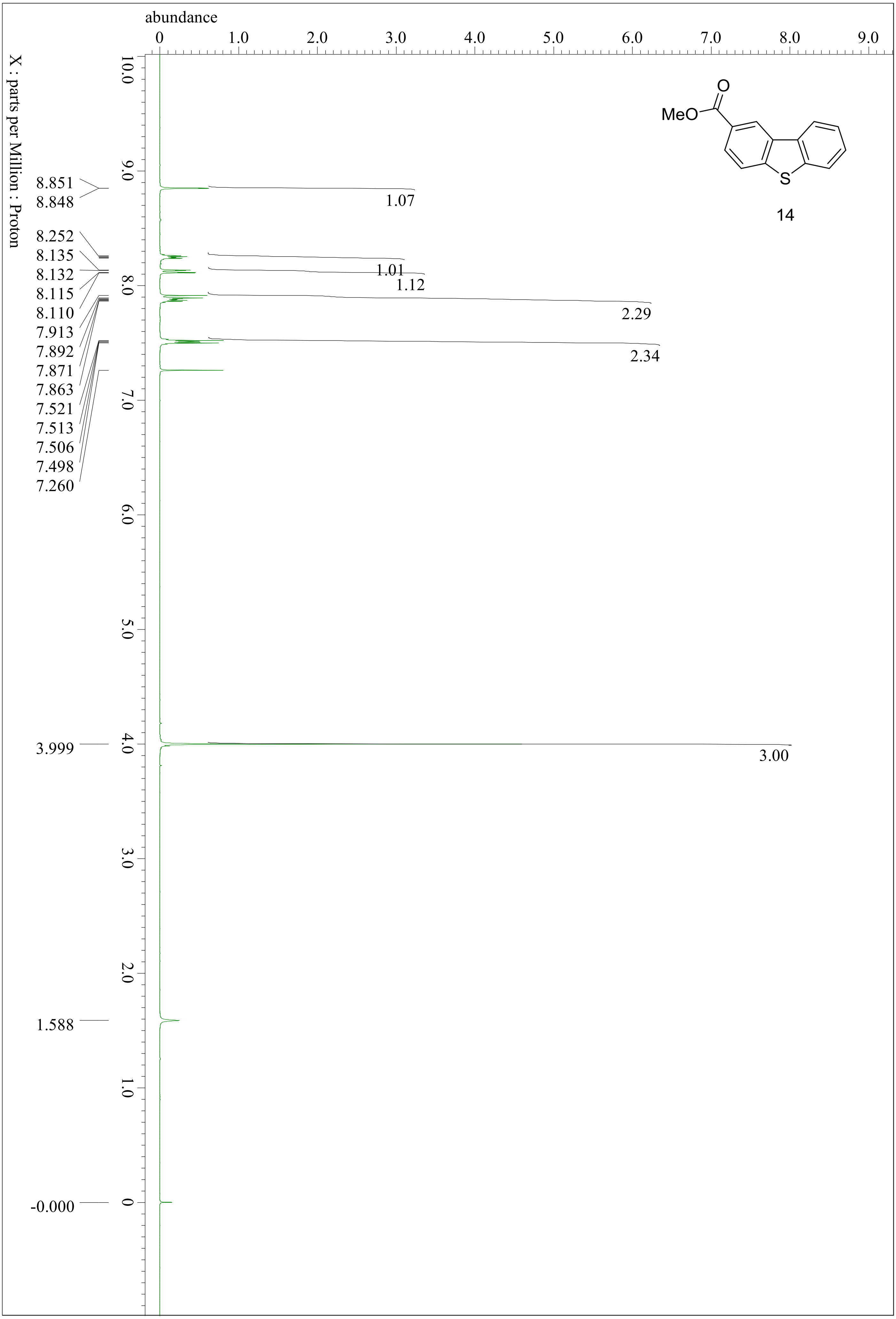

abundance
0
1.0
2.0
3.0
4.0
5.0
6.0
7.0
8.0
9.0
X : parts per Million : Proton
10.0
9.0
8.0
7.0
6.0
5.0
4.0
3.0
2.0
1.0
0
8.851
8.848
8.252
8.135
8.132
8.115
8.110
7.913
7.892
7.871
7.863
7.521
7.513
7.506
7.498
7.260
3.999
1.588
-0.000
1.07
1.01
1.12
2.29
2.34
3.00
MeO
O
S
14

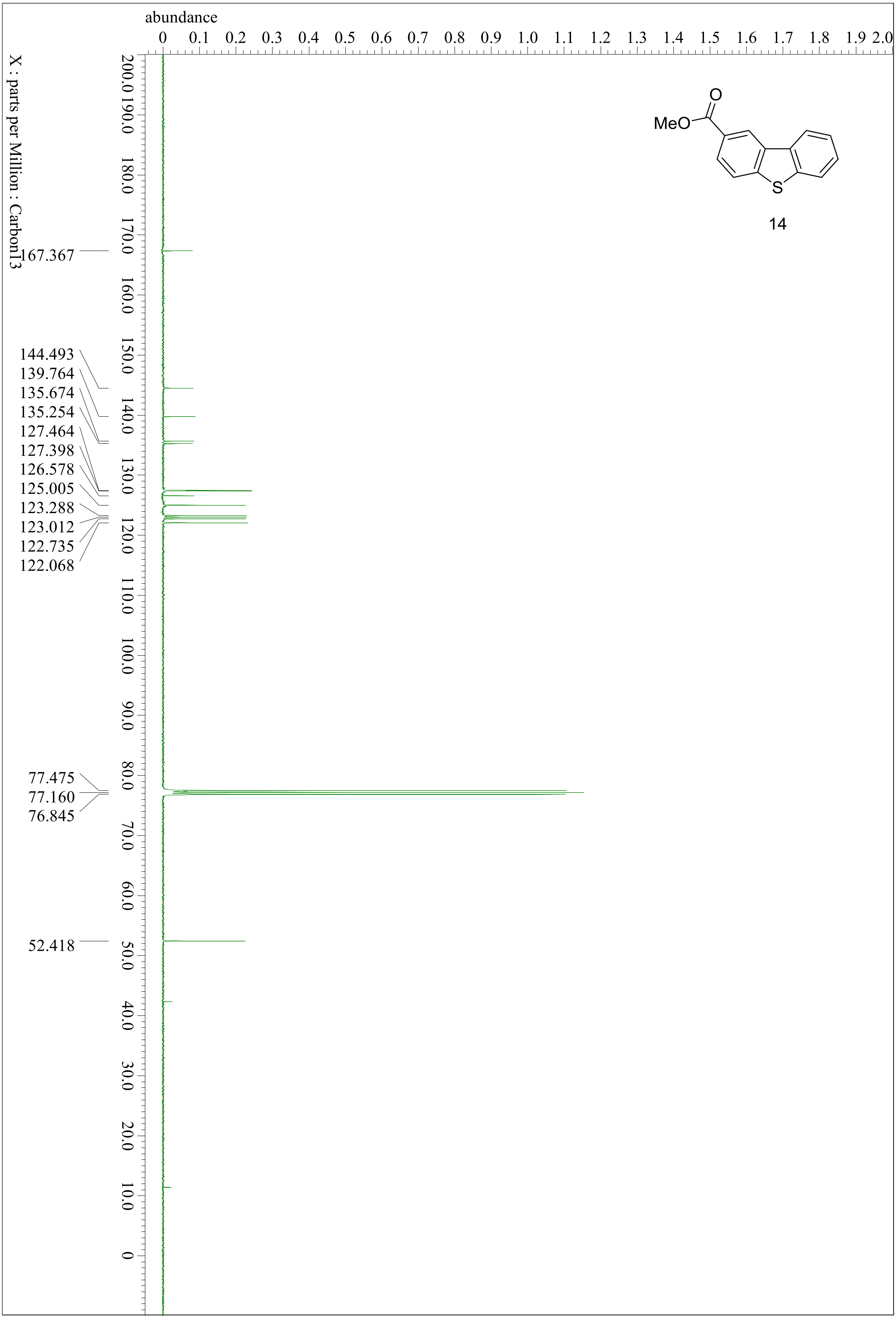
abundance
0 0.1 0.2 0.3 0.4 0.5 0.6 0.7 0.8 0.9 1.0 1.1 1.2 1.3 1.4 1.5 1.6 1.7 1.8 1.9 2.0
X : parts per Million : Carbon13
200.0 190.0 180.0 170.0 160.0 150.0 140.0 130.0 120.0 110.0 100.0 90.0 80.0 70.0 60.0 50.0 40.0 30.0 20.0 10.0 0
167.367
144.493
139.764
135.674
135.254
127.464
127.398
126.578
125.005
123.288
123.012
122.735
122.068
77.475
77.160
76.845
52.418
MeO
O
S
14

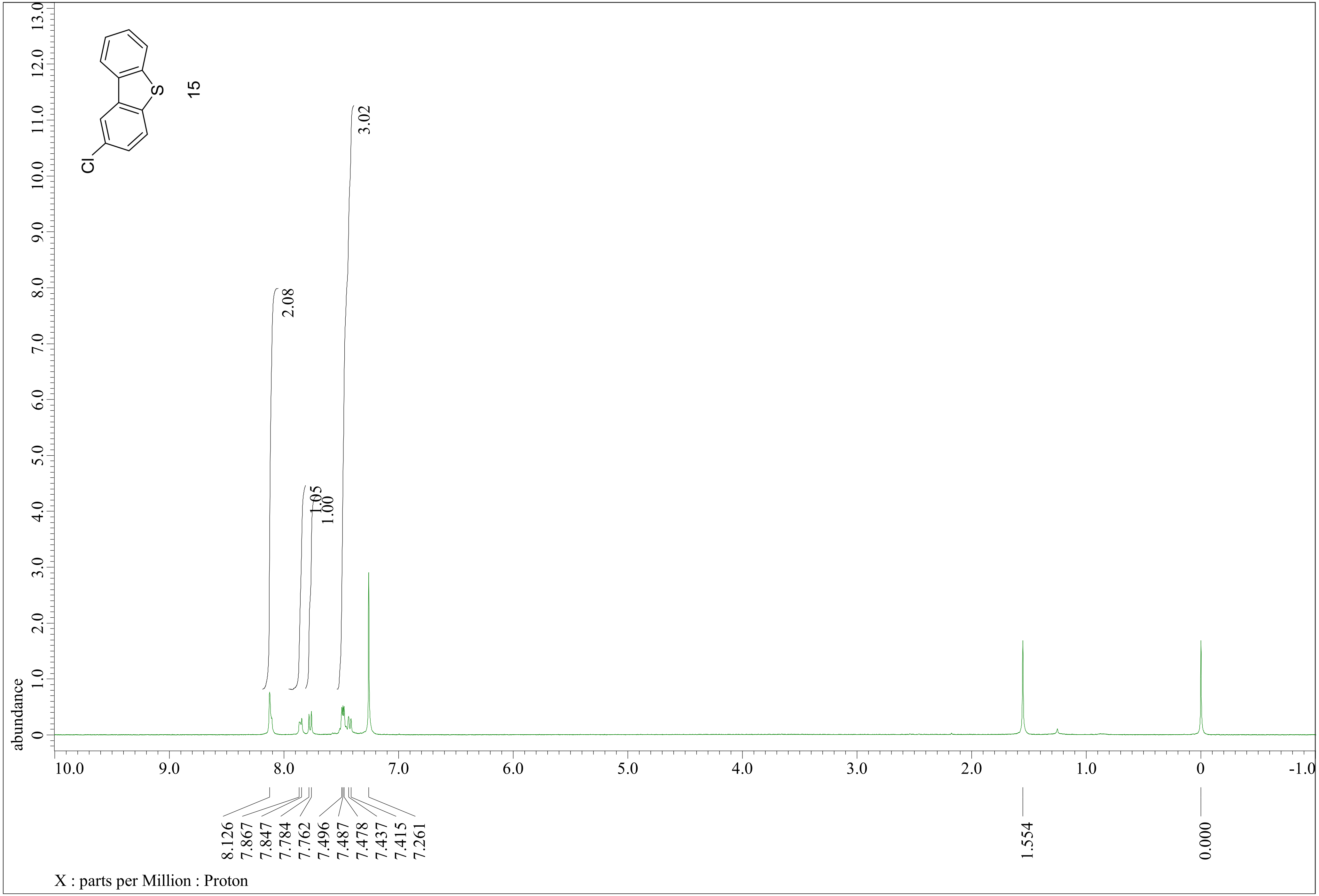

15
abundance
3.02
2.08
1.05
1.00
8.126
7.867
7.847
7.784
7.762
7.496
7.487
7.478
7.437
7.415
7.261
1.554
0.000
X : parts per Million : Proton

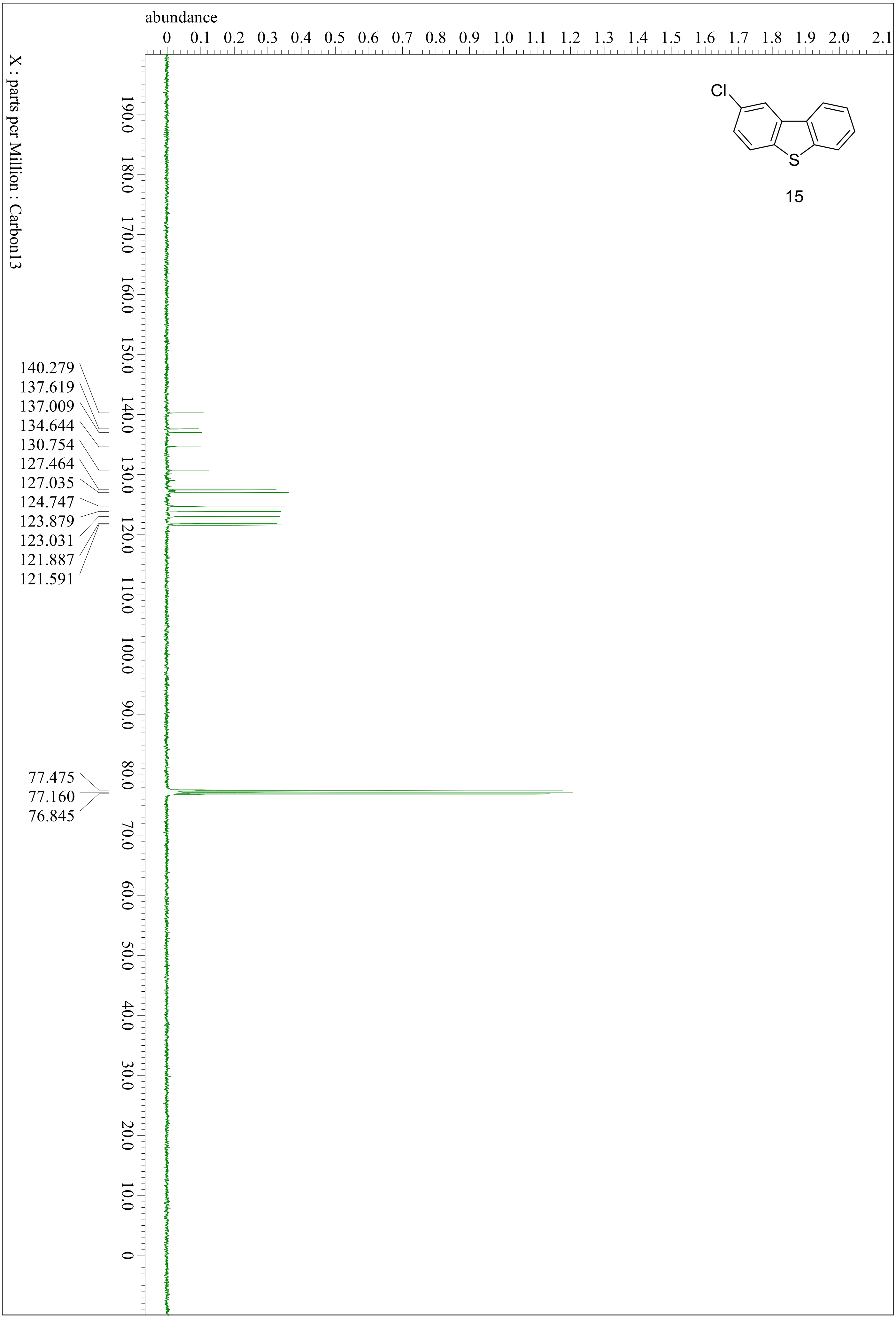
abundance
0 0.1 0.2 0.3 0.4 0.5 0.6 0.7 0.8 0.9 1.0 1.1 1.2 1.3 1.4 1.5 1.6 1.7 1.8 1.9 2.0 2.1
X : parts per Million : Carbon13
190.0 180.0 170.0 160.0 150.0 140.0 130.0 120.0 110.0 100.0 90.0 80.0 70.0 60.0 50.0 40.0 30.0 20.0 10.0 0
140.279
137.619
137.009
134.644
130.754
127.464
127.035
124.747
123.879
123.031
121.887
121.591
77.475
77.160
76.845
Cl
S
15

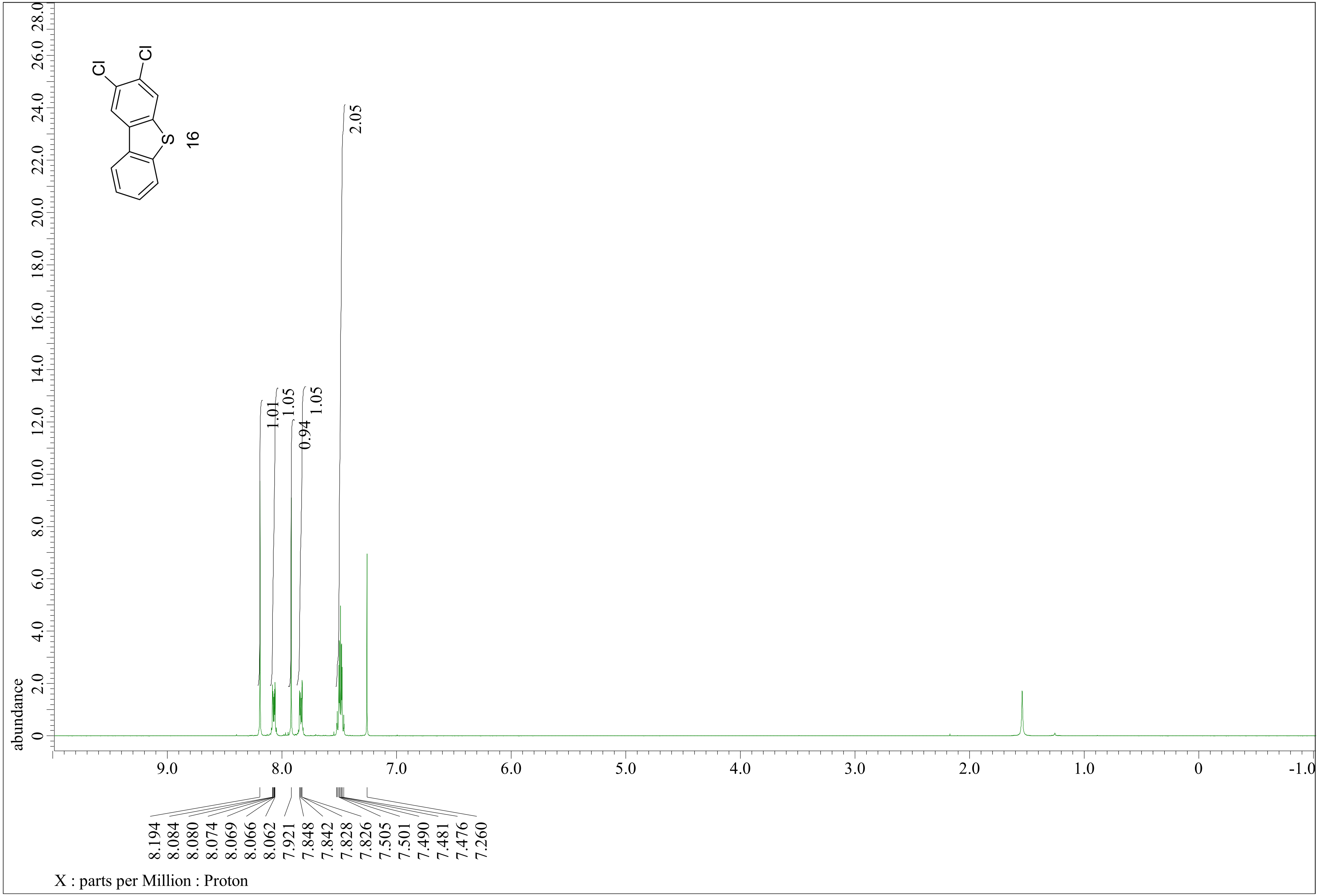

abundance
0
2.0
4.0
6.0
8.0
10.0
12.0
14.0
16.0
18.0
20.0
22.0
24.0
26.0
28.0
Cl
Cl
S
16
1.01
1.05
0.94
1.05
2.05
9.0
8.0
7.0
6.0
5.0
4.0
3.0
2.0
1.0
0
-1.0
8.194
8.084
8.080
8.074
8.069
8.066
8.062
7.921
7.848
7.842
7.828
7.826
7.505
7.501
7.490
7.481
7.476
7.260
X : parts per Million : Proton

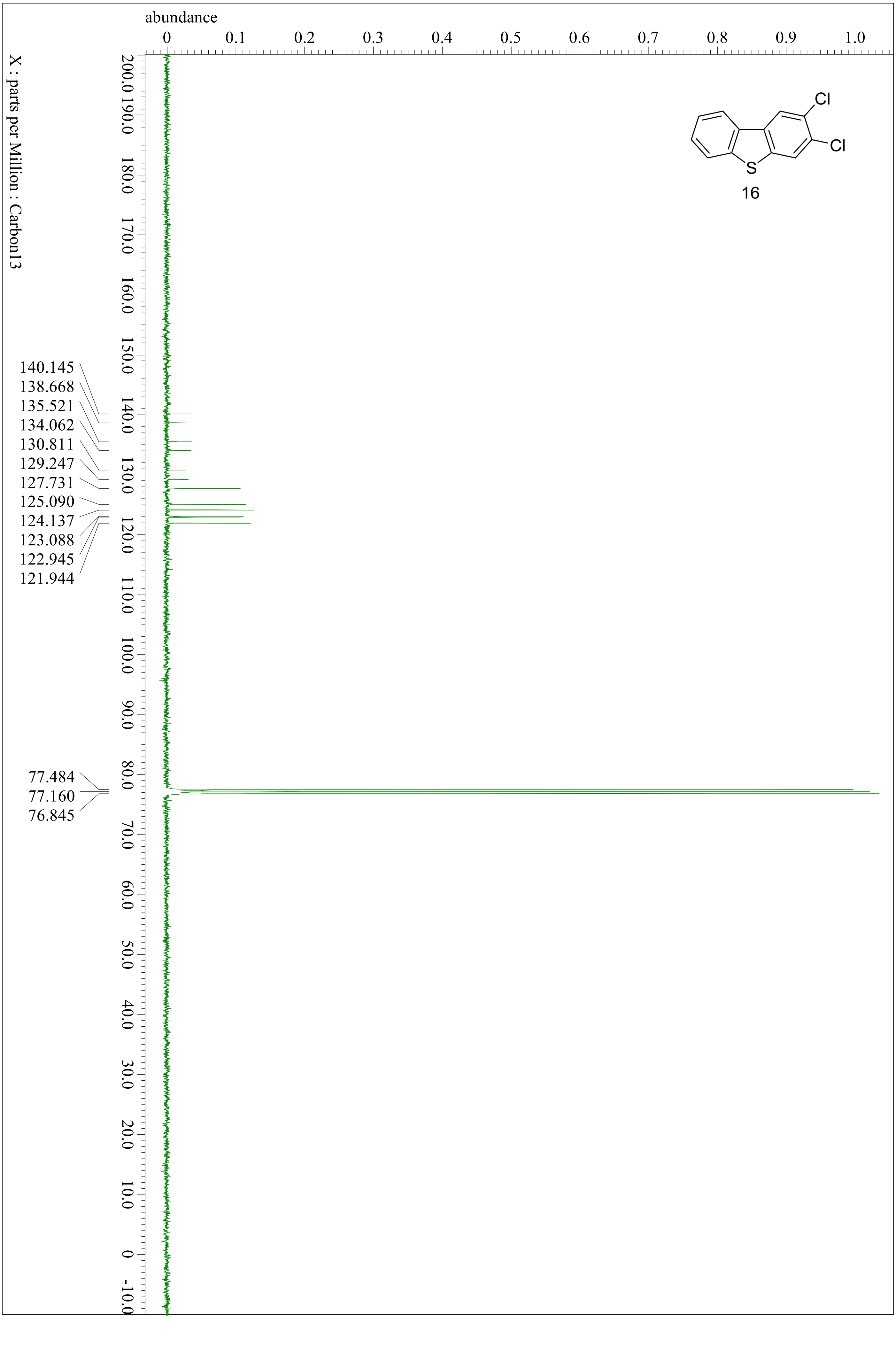

abundance
0 0.1 0.2 0.3 0.4 0.5 0.6 0.7 0.8 0.9 1.0
X : parts per Million : Carbon13
200.0 190.0 180.0 170.0 160.0 150.0 140.0 130.0 120.0 110.0 100.0 90.0 80.0 70.0 60.0 50.0 40.0 30.0 20.0 10.0 0 -10.0
140.145
138.668
135.521
134.062
130.811
129.247
127.731
125.090
124.137
123.088
122.945
121.944
77.484
77.160
76.845
Cl
Cl
S
16

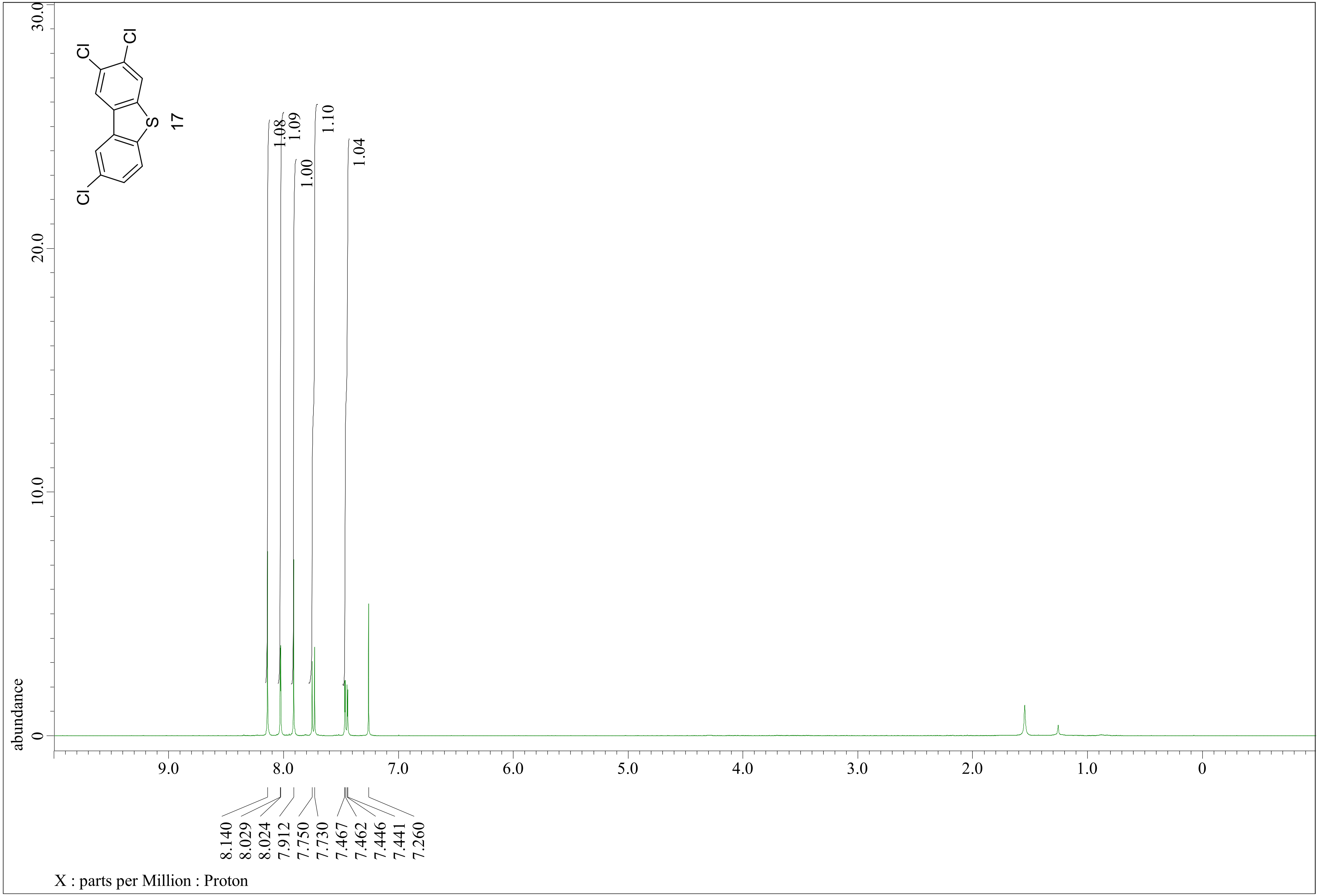
Cl
Cl
Cl
S
17
abundance
30.0
20.0
10.0
0
1.08
1.09
1.00
1.10
1.04
9.0
8.0
7.0
6.0
5.0
4.0
3.0
2.0
1.0
0
8.140
8.029
8.024
7.912
7.750
7.730
7.467
7.462
7.446
7.441
7.260
X : parts per Million : Proton

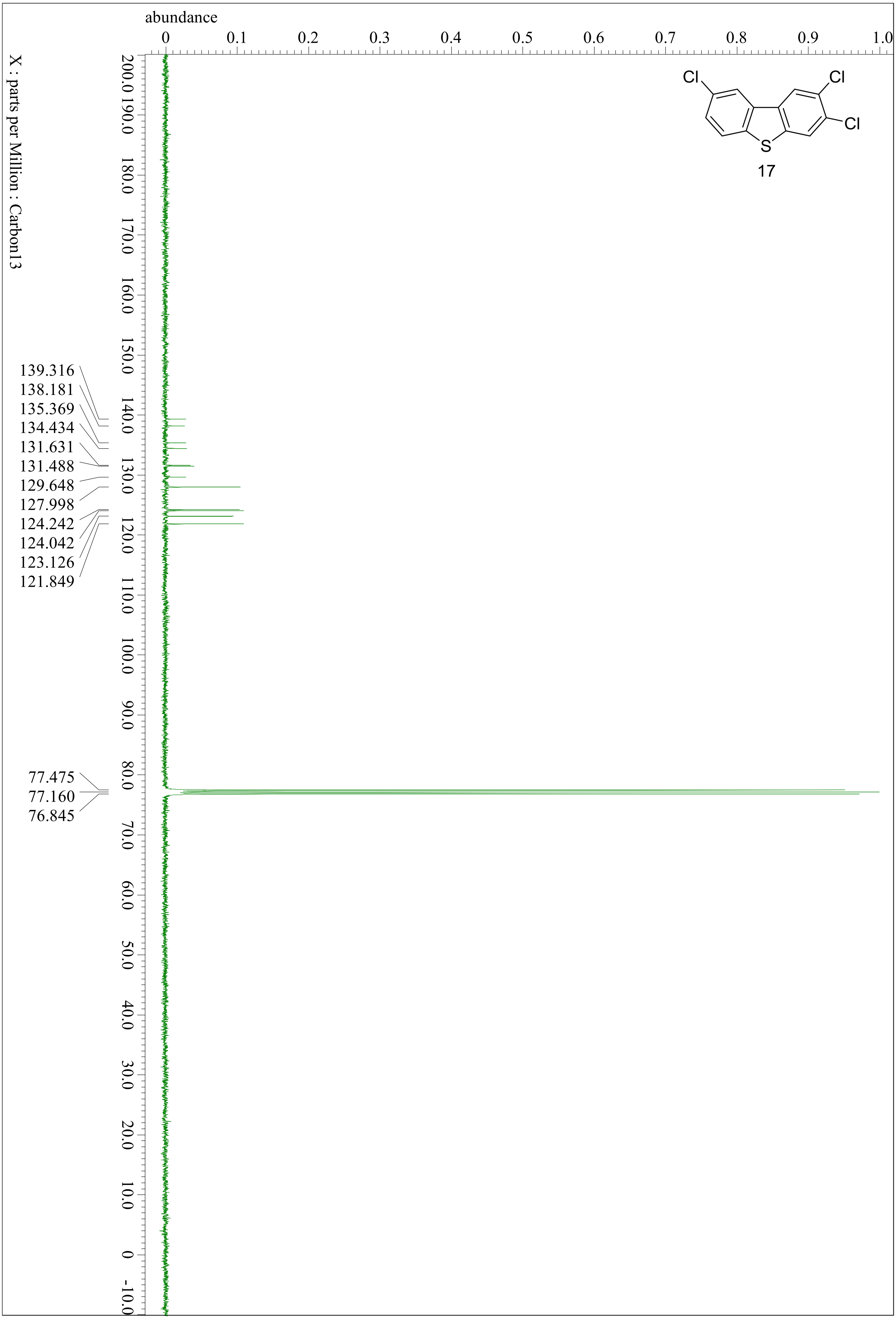

abundance
0
0.1
0.2
0.3
0.4
0.5
0.6
0.7
0.8
0.9
1.0
X : parts per Million : Carbon13
200.0
190.0
180.0
170.0
160.0
150.0
140.0
130.0
120.0
110.0
100.0
90.0
80.0
70.0
60.0
50.0
40.0
30.0
20.0
10.0
0
-10.0
139.316
138.181
135.369
134.434
131.631
131.488
129.648
127.998
124.242
124.042
123.126
121.849
77.475
77.160
76.845
Cl
Cl
Cl
S
17

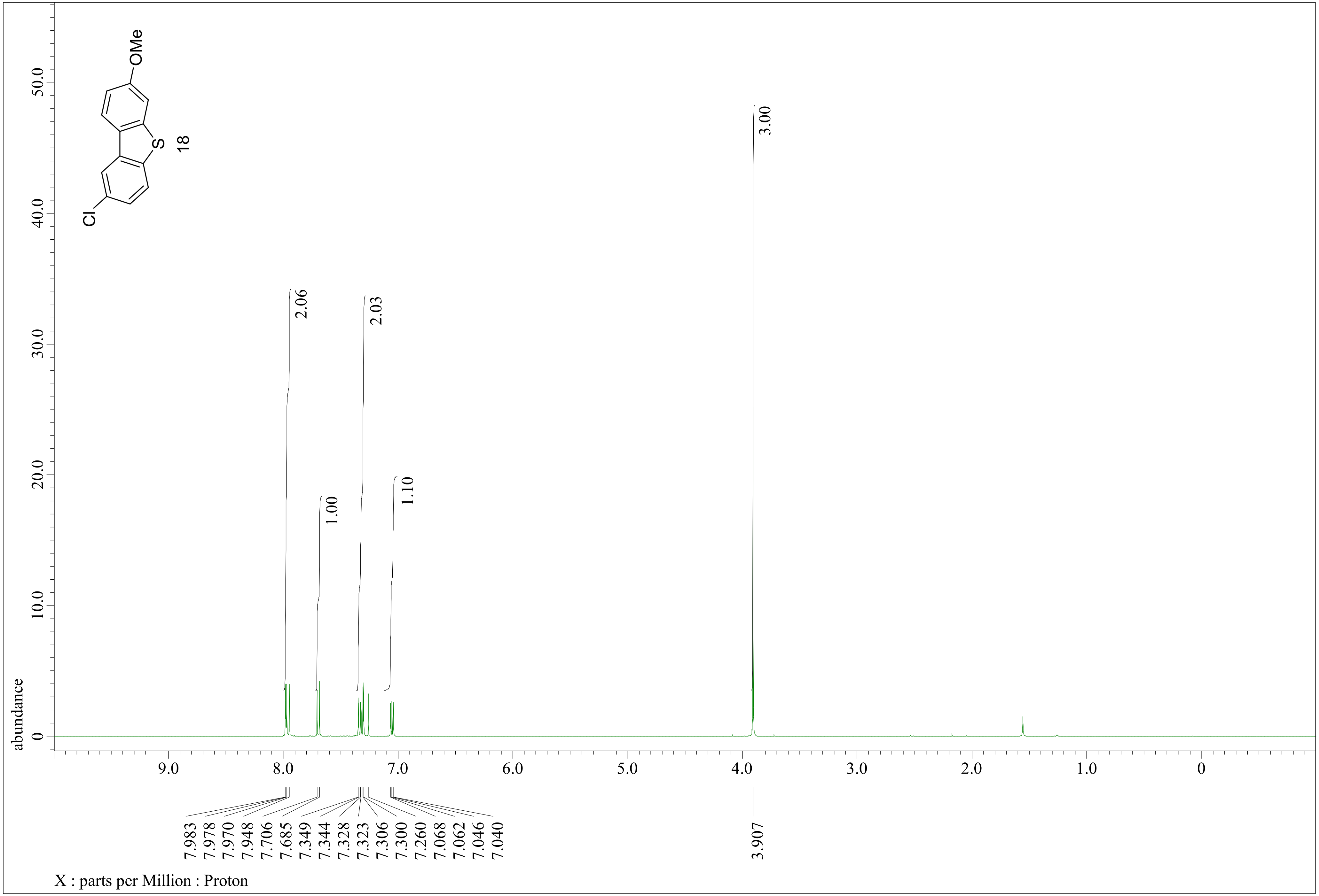

abundance
0
10.0
20.0
30.0
40.0
50.0
9.0
8.0
7.0
6.0
5.0
4.0
3.0
2.0
1.0
0
7.983
7.978
7.970
7.948
7.706
7.685
7.349
7.344
7.328
7.323
7.306
7.300
7.260
7.068
7.062
7.046
7.040
3.907
2.06
1.00
2.03
1.10
3.00
X : parts per Million : Proton
Cl
OMe
S
18

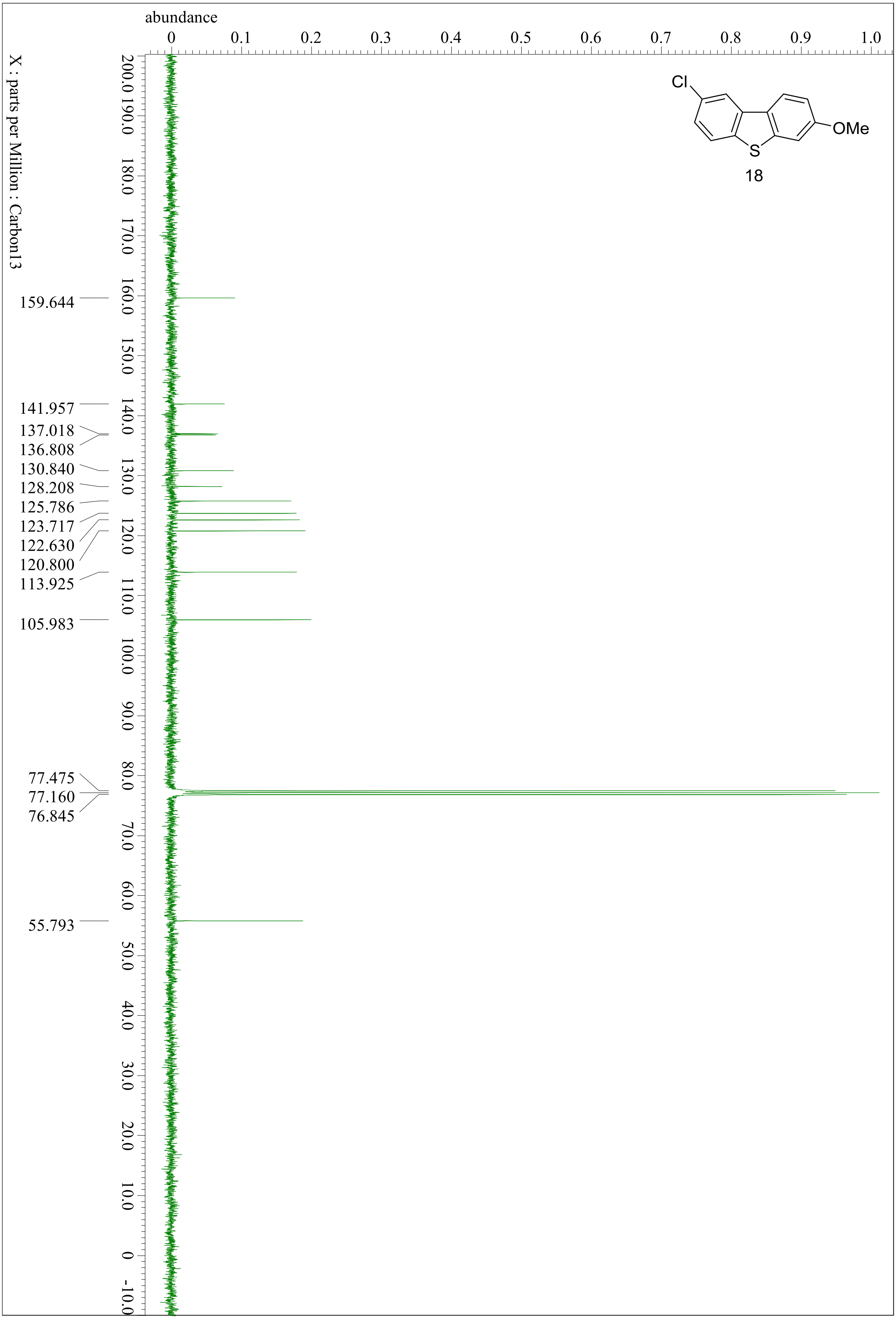
abundance
0 0.1 0.2 0.3 0.4 0.5 0.6 0.7 0.8 0.9 1.0
X : parts per Million : Carbon13
200.0 190.0 180.0 170.0 160.0 150.0 140.0 130.0 120.0 110.0 100.0 90.0 80.0 70.0 60.0 50.0 40.0 30.0 20.0 10.0 0 -10.0
159.644
141.957
137.018
136.808
130.840
128.208
125.786
123.717
122.630
120.800
113.925
105.983
77.475
77.160
76.845
55.793
Cl
OMe
S
18

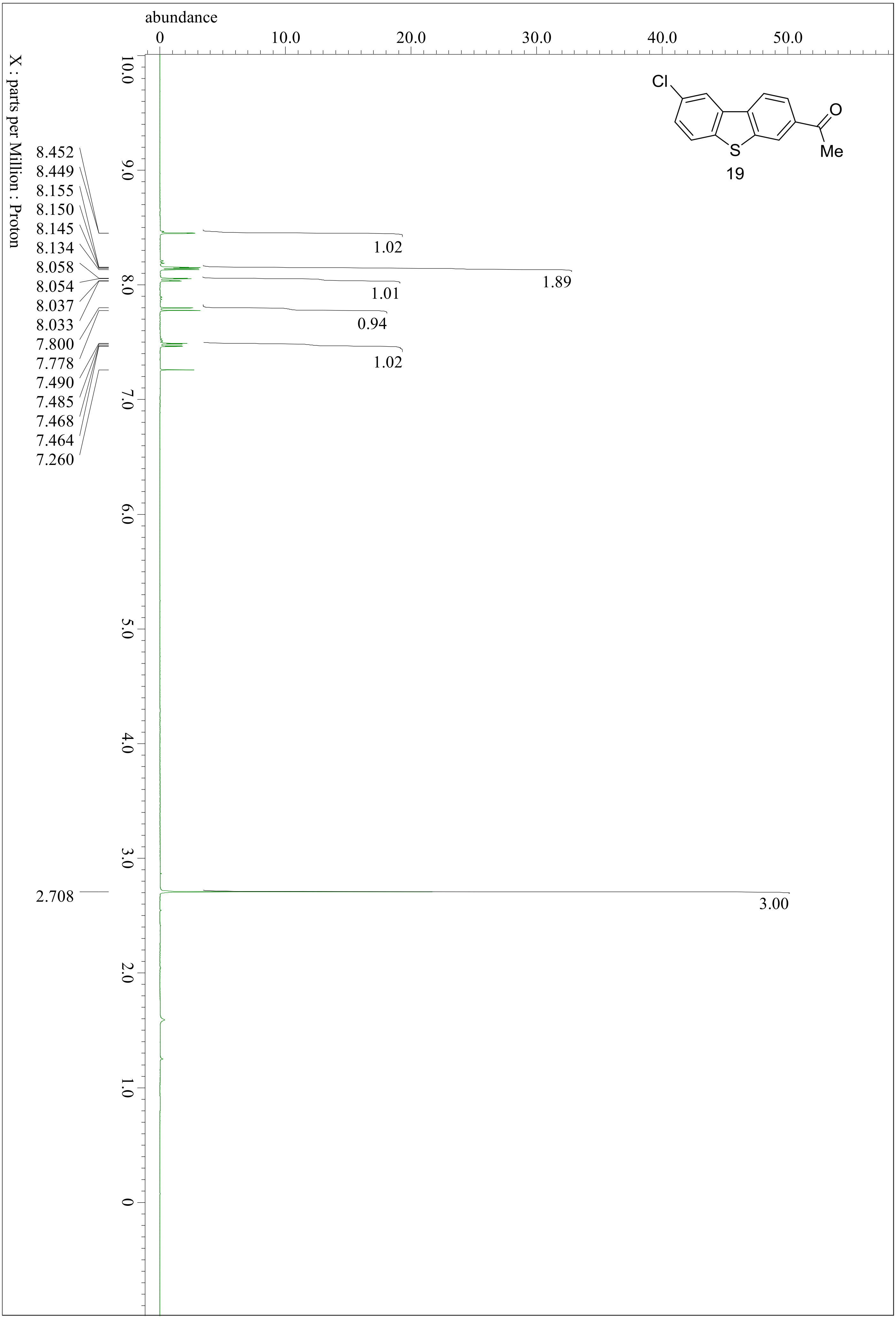

abundance
0
10.0
20.0
30.0
40.0
50.0
X : parts per Million : Proton
10.0
9.0
8.0
7.0
6.0
5.0
4.0
3.0
2.0
1.0
0
8.452
8.449
8.155
8.150
8.145
8.134
8.058
8.054
8.037
8.033
7.800
7.778
7.490
7.485
7.468
7.464
7.260
2.708
1.02
1.89
1.01
0.94
1.02
3.00
Cl
O
Me
S
19

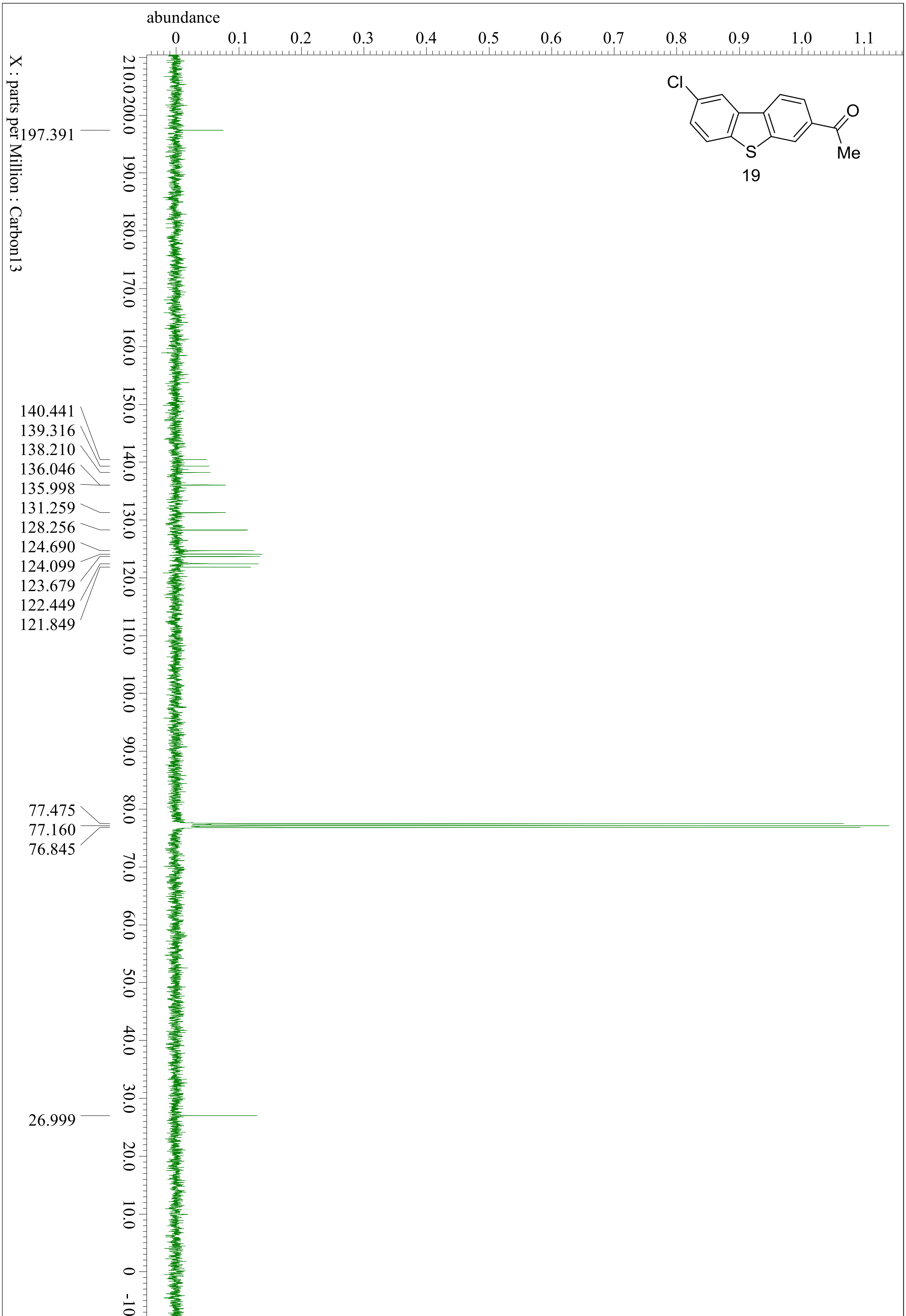
abundance
0
0.1
0.2
0.3
0.4
0.5
0.6
0.7
0.8
0.9
1.0
1.1
X : parts per Million : Carbon13
210.0
200.0
190.0
180.0
170.0
160.0
150.0
140.0
130.0
120.0
110.0
100.0
90.0
80.0
70.0
60.0
50.0
40.0
30.0
20.0
10.0
0
-10.0
197.391
140.441
139.316
138.210
136.046
135.998
131.259
128.256
124.690
124.099
123.679
122.449
121.849
77.475
77.160
76.845
26.999
Cl
O
S
Me
19

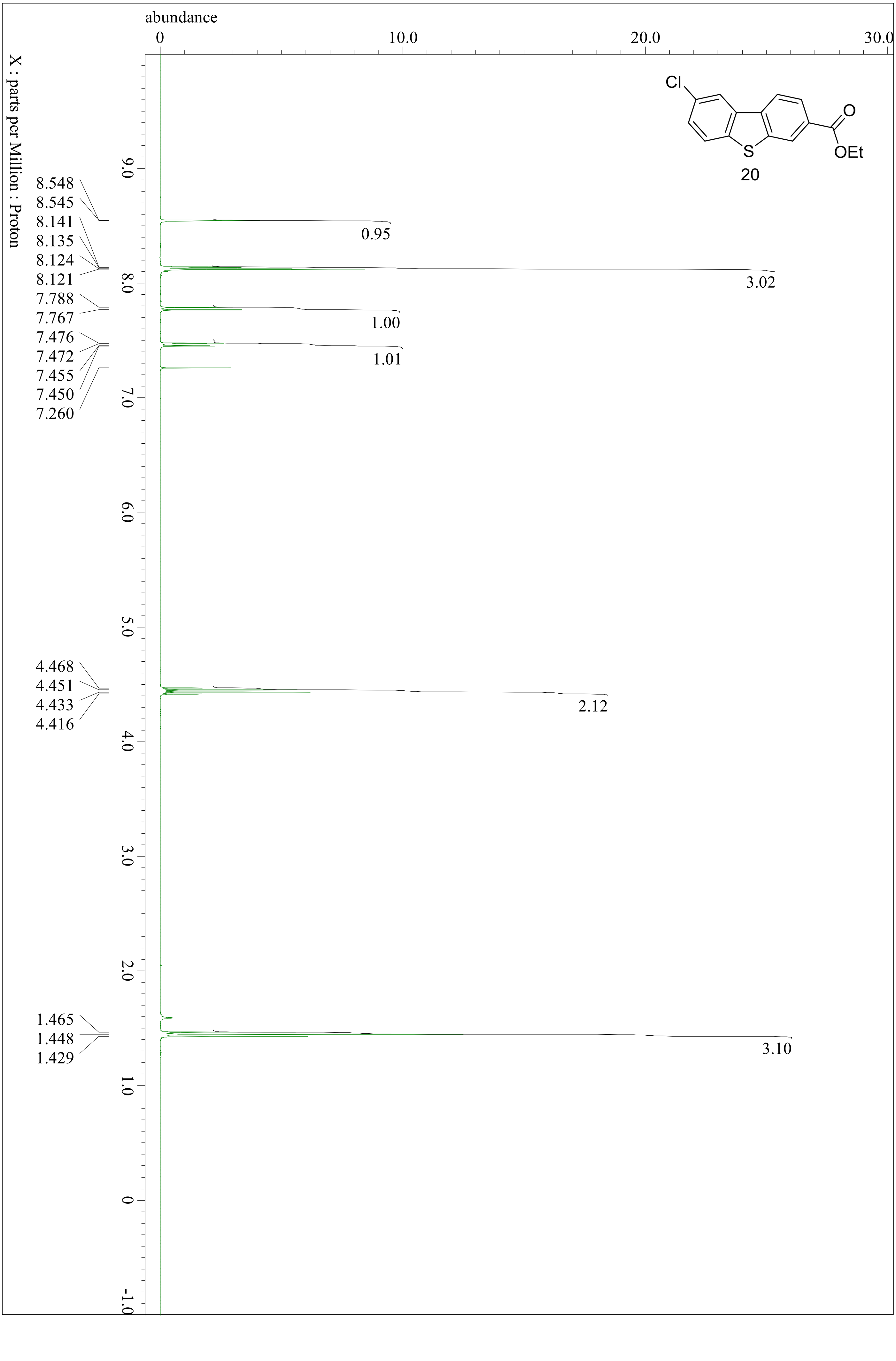

abundance
0
10.0
20.0
30.0
X : parts per Million : Proton
8.548
8.545
8.141
8.135
8.124
8.121
7.788
7.767
7.476
7.472
7.455
7.450
7.260
4.468
4.451
4.433
4.416
1.465
1.448
1.429
9.0
8.0
7.0
6.0
5.0
4.0
3.0
2.0
1.0
0
-1.0
0.95
3.02
1.00
1.01
2.12
3.10
Cl
O
OEt
S
20

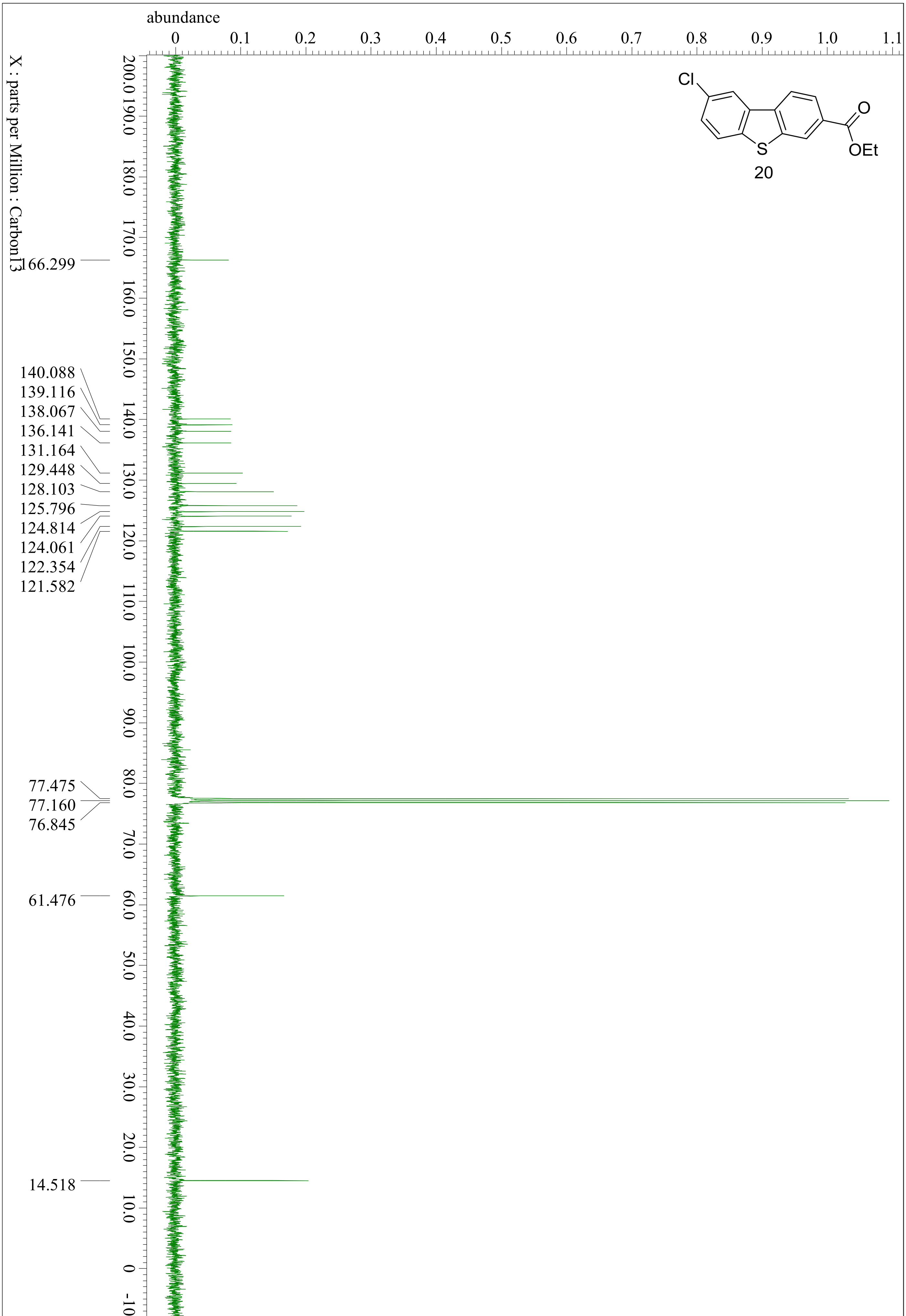
abundance
X : parts per Million : Carbon13
166.299
140.088
139.116
138.067
136.141
131.164
129.448
128.103
125.796
124.814
124.061
122.354
121.582
77.475
77.160
76.845
61.476
14.518
Cl
O
OEt
S
20

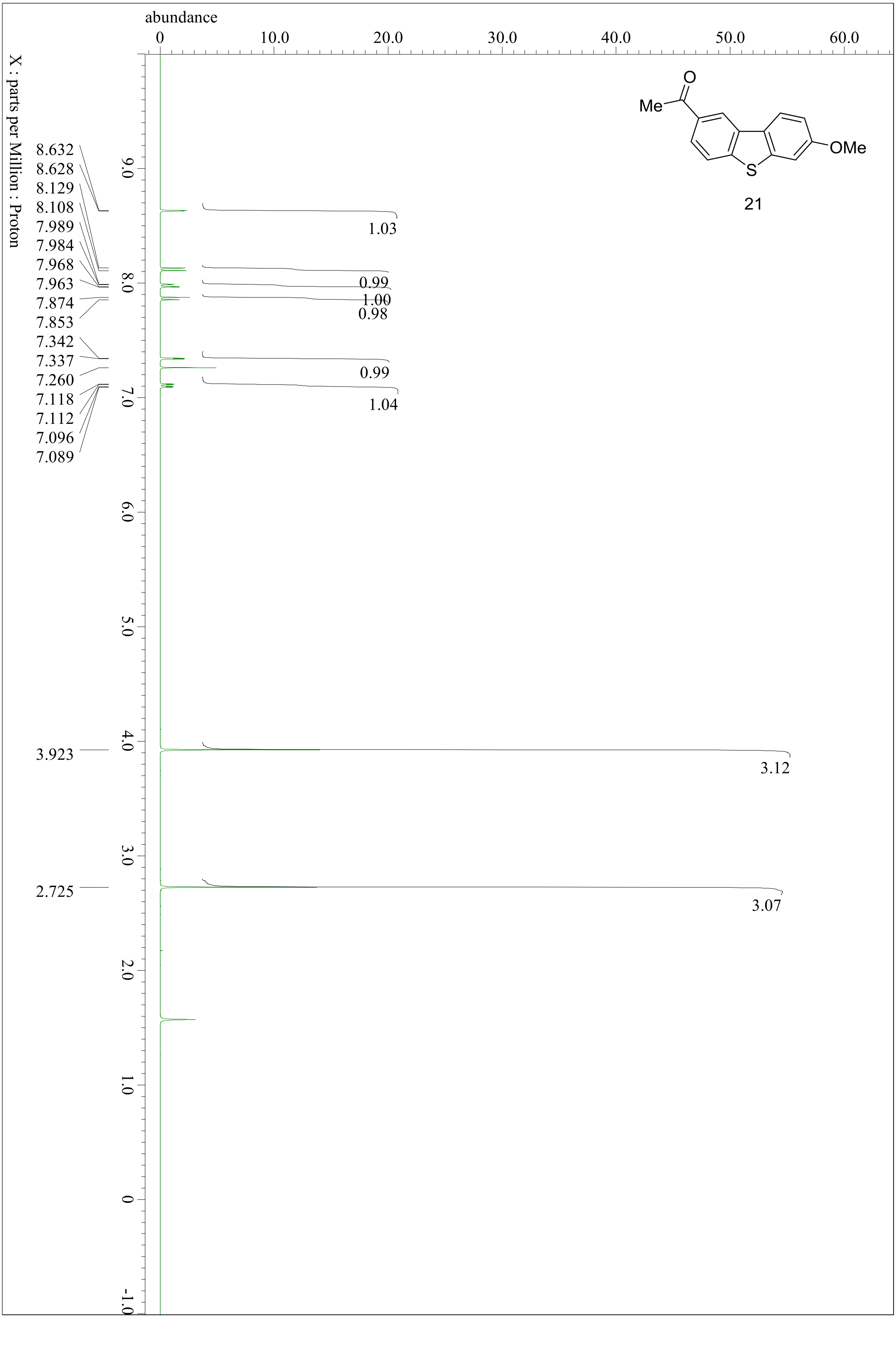
abundance
0
10.0
20.0
30.0
40.0
50.0
60.0
X : parts per Million : Proton
9.0
8.0
7.0
6.0
5.0
4.0
3.0
2.0
1.0
0
-1.0
8.632
8.628
8.129
8.108
7.989
7.984
7.968
7.963
7.874
7.853
7.342
7.337
7.260
7.118
7.112
7.096
7.089
3.923
2.725
1.03
0.99
1.00
0.98
0.99
1.04
3.12
3.07
O
Me
OMe
S
21

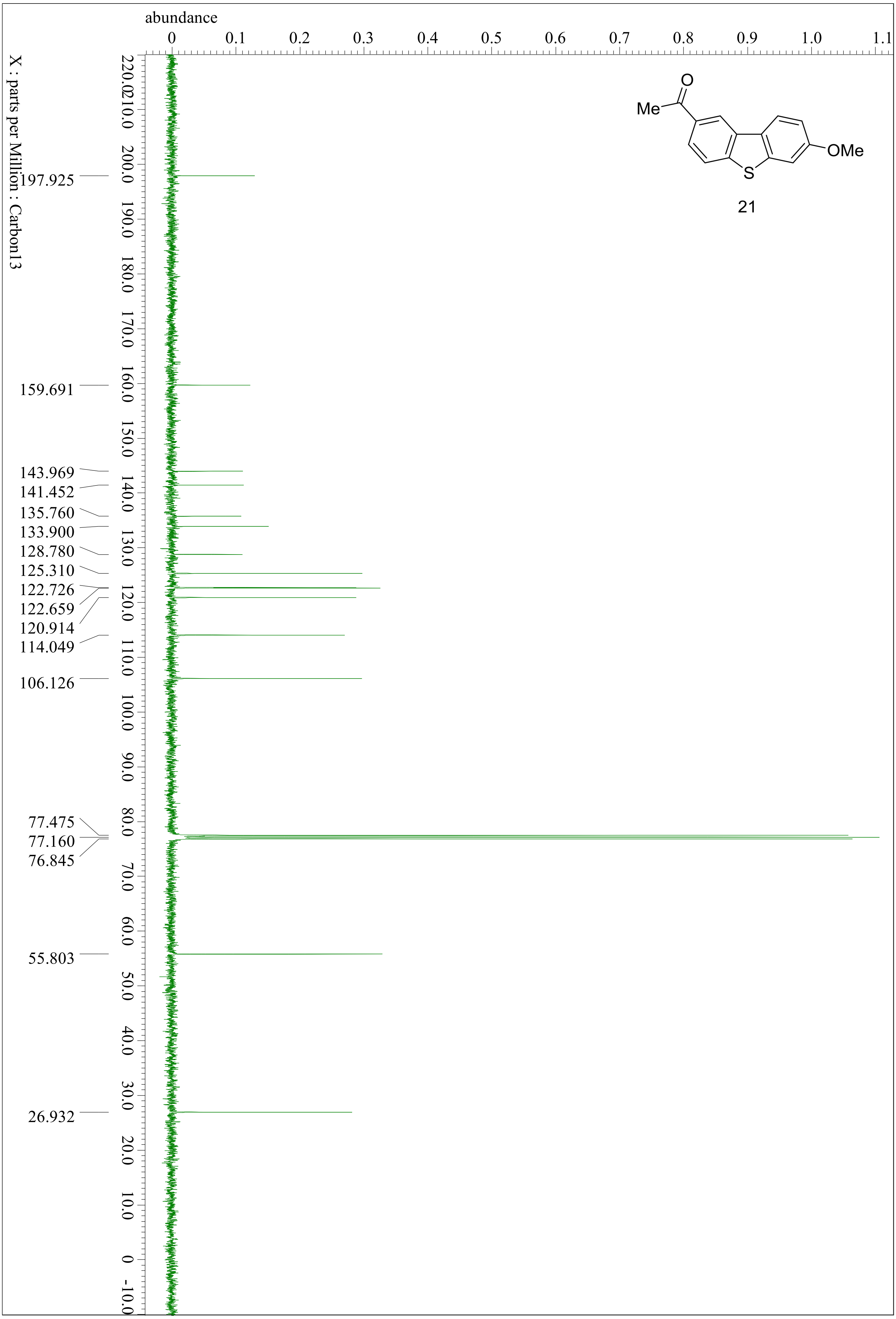
abundance
0 0.1 0.2 0.3 0.4 0.5 0.6 0.7 0.8 0.9 1.0 1.1
X : parts per Million : Carbon13
220.0 210.0 200.0 190.0 180.0 170.0 160.0 150.0 140.0 130.0 120.0 110.0 100.0 90.0 80.0 70.0 60.0 50.0 40.0 30.0 20.0 10.0 0 -10.0
197.925
159.691
143.969
141.452
135.760
133.900
128.780
125.310
122.726
122.659
120.914
114.049
106.126
77.475
77.160
76.845
55.803
26.932
O
Me
OMe
S
21

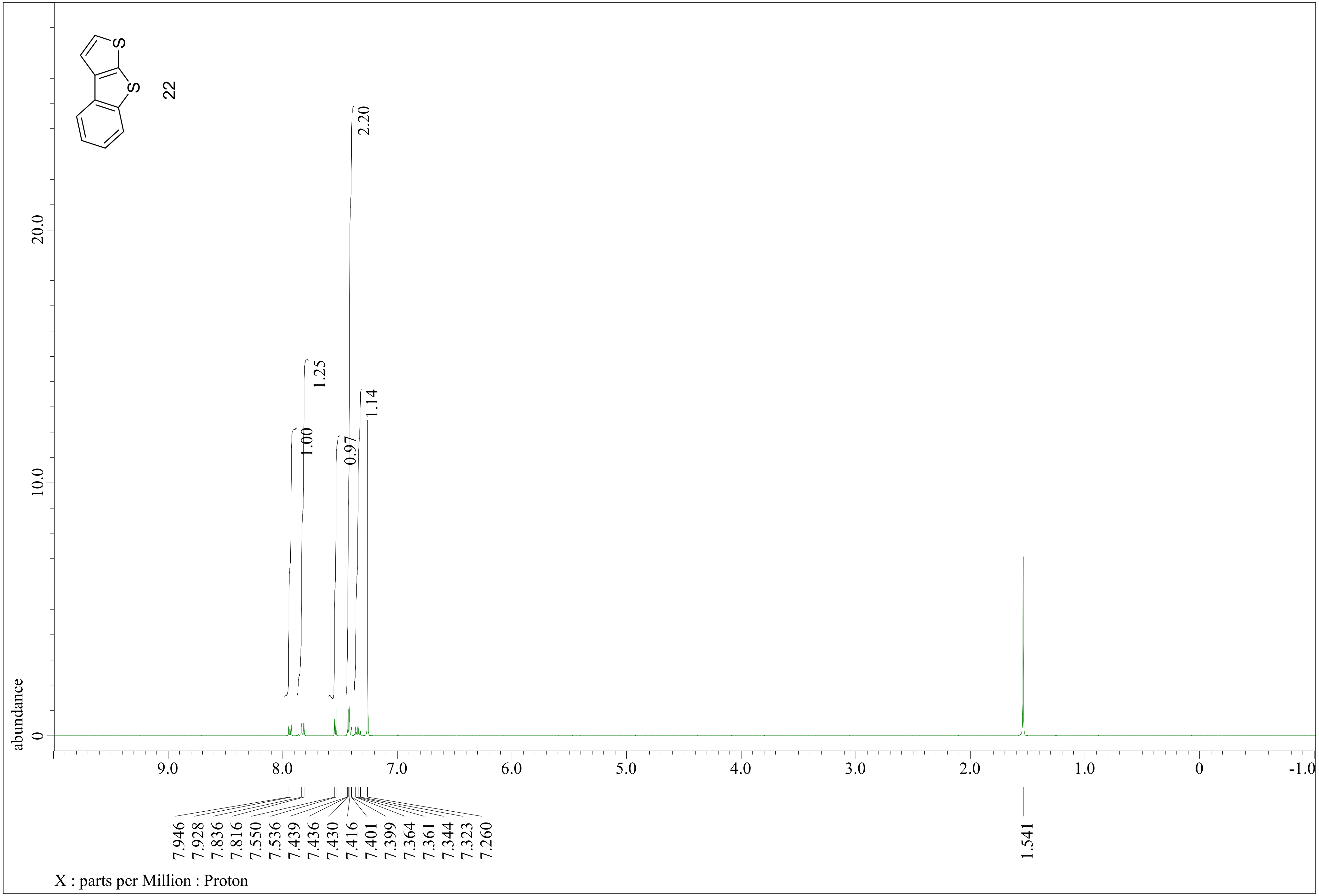
S
S
22
2.20
1.25
1.00
0.97
1.14
20.0
10.0
0
abundance
9.0
8.0
7.0
6.0
5.0
4.0
3.0
2.0
1.0
0
-1.0
7.946
7.928
7.836
7.816
7.550
7.536
7.439
7.436
7.430
7.416
7.401
7.399
7.364
7.361
7.344
7.323
7.260
1.541
X : parts per Million : Proton

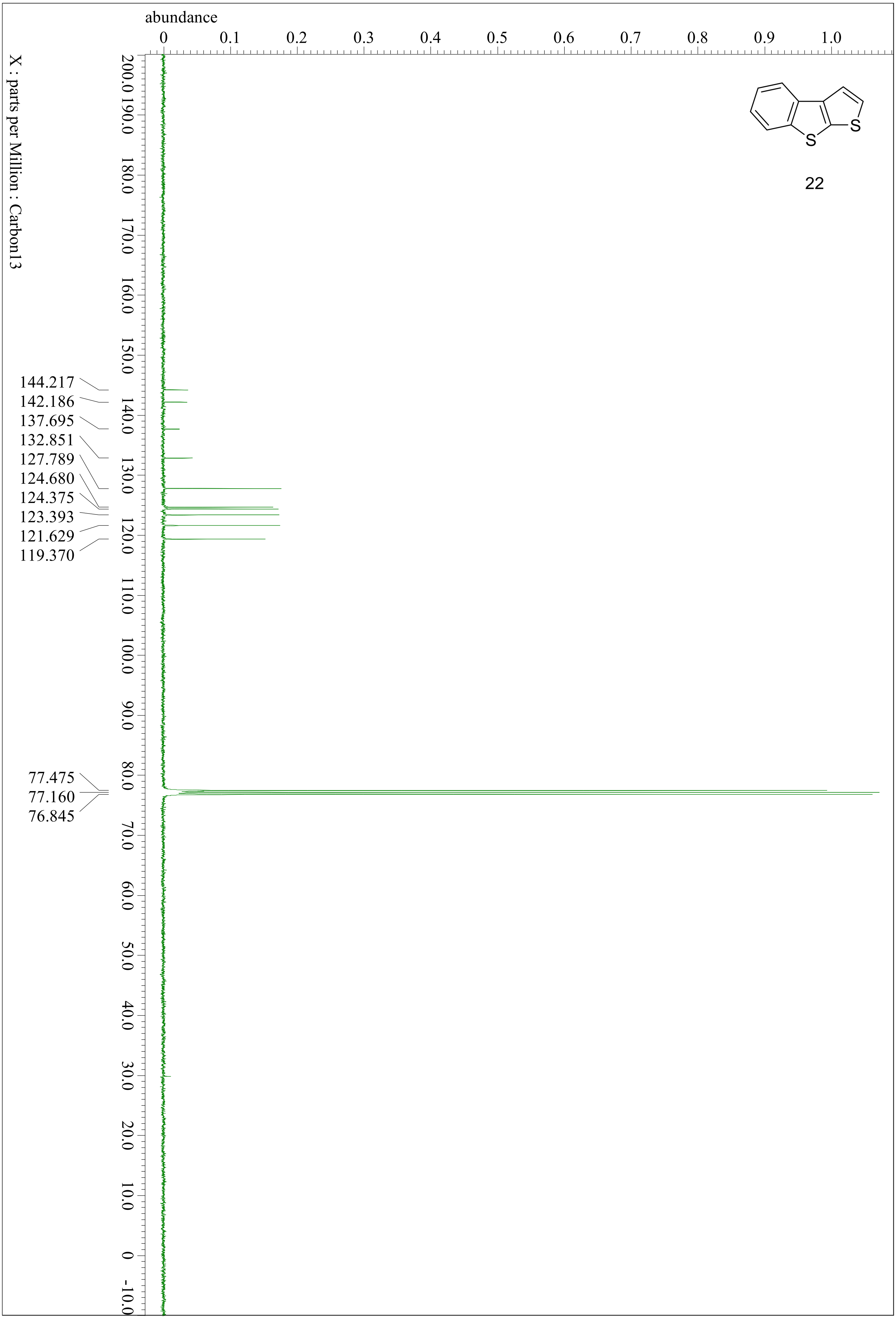
abundance
0 0.1 0.2 0.3 0.4 0.5 0.6 0.7 0.8 0.9 1.0
X : parts per Million : Carbon13
200.0 190.0 180.0 170.0 160.0 150.0 140.0 130.0 120.0 110.0 100.0 90.0 80.0 70.0 60.0 50.0 40.0 30.0 20.0 10.0 0 -10.0
144.217
142.186
137.695
132.851
127.789
124.680
124.375
123.393
121.629
119.370
77.475
77.160
76.845
S
S
22

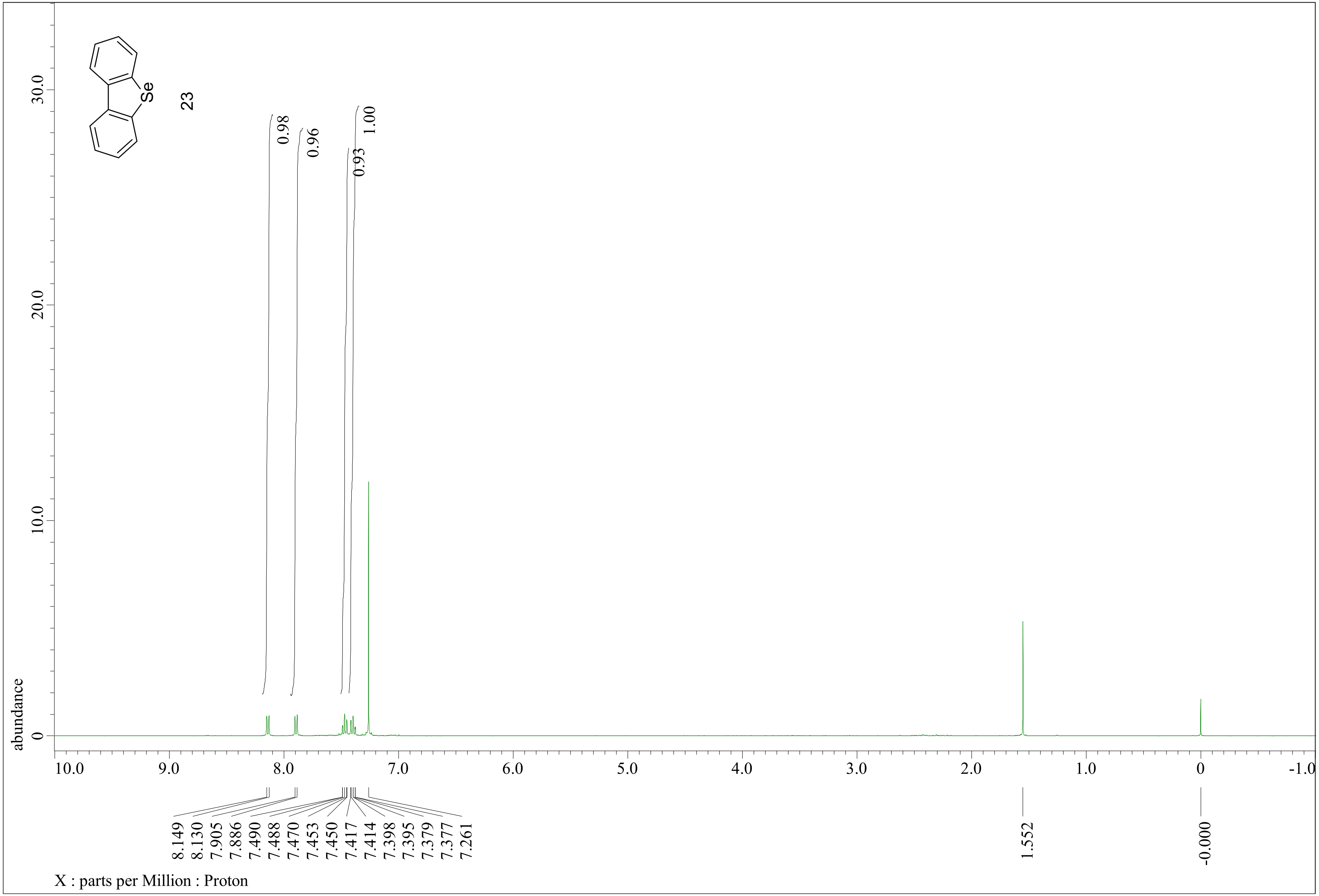

Se
23
0.98
0.96
0.93
1.00
abundance
0
10.0
20.0
30.0
10.0 9.0 8.0 7.0 6.0 5.0 4.0 3.0 2.0 1.0 0 -1.0
8.149
8.130
7.905
7.886
7.490
7.488
7.470
7.453
7.450
7.417
7.414
7.398
7.395
7.379
7.377
7.261
1.552
-0.000
X : parts per Million : Proton

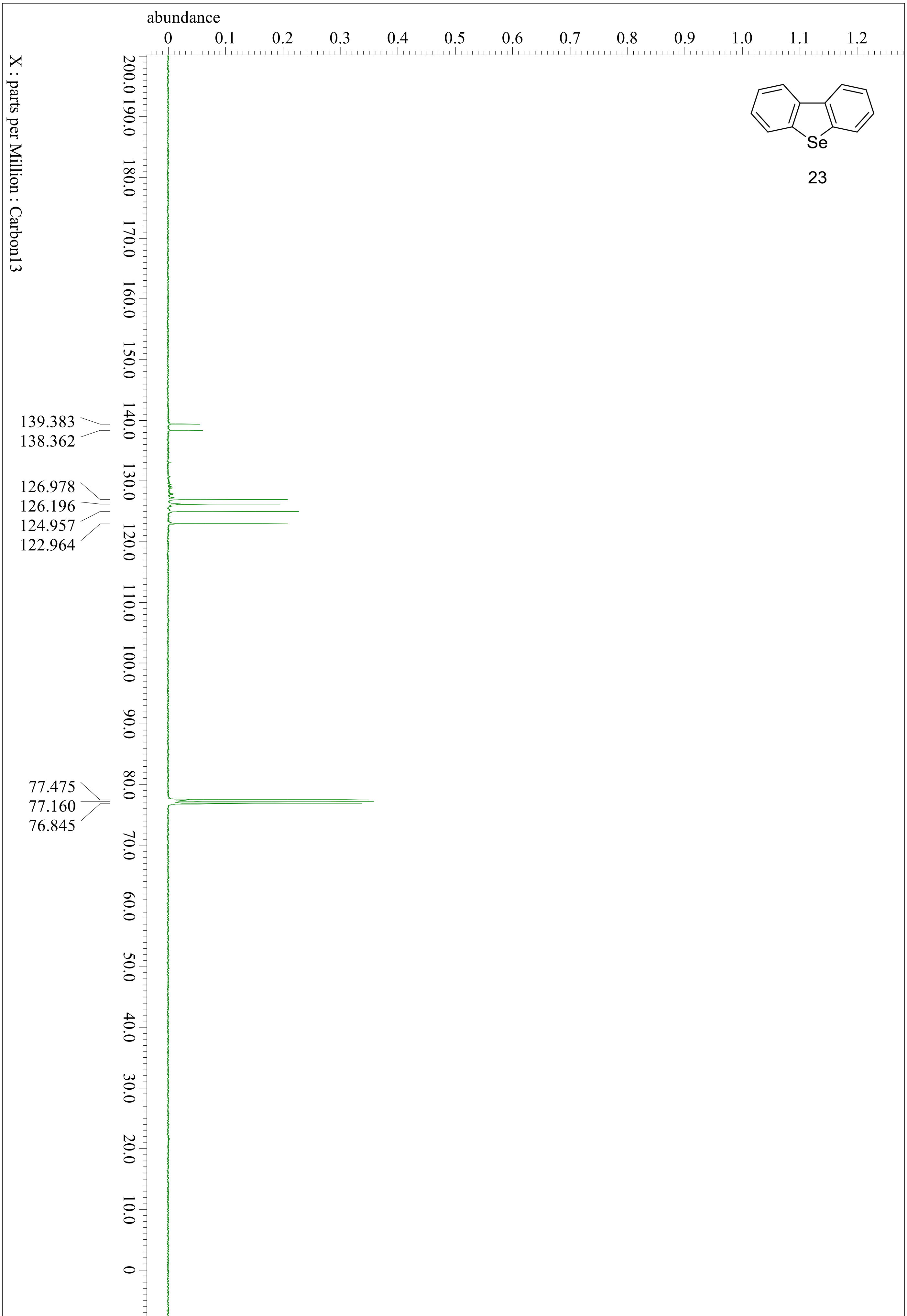

abundance
0
0.1
0.2
0.3
0.4
0.5
0.6
0.7
0.8
0.9
1.0
1.1
1.2
X : parts per Million : Carbon13
200.0
190.0
180.0
170.0
160.0
150.0
140.0
130.0
120.0
110.0
100.0
90.0
80.0
70.0
60.0
50.0
40.0
30.0
20.0
10.0
0
139.383
138.362
126.978
126.196
124.957
122.964
77.475
77.160
76.845
Se
23

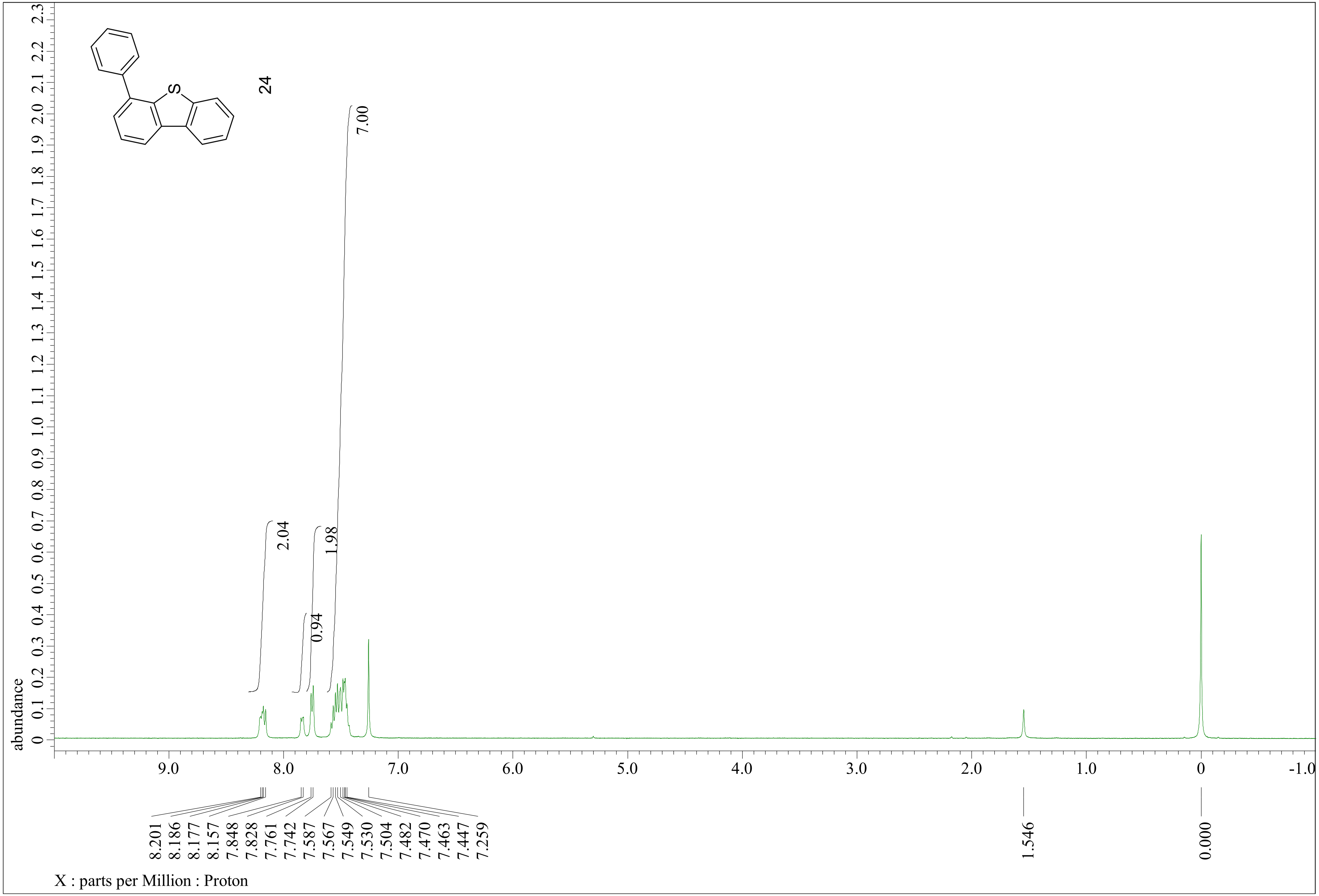

24
abundance
2.04
0.94
1.98
7.00
8.201
8.186
8.177
8.157
7.848
7.828
7.761
7.742
7.587
7.567
7.549
7.530
7.504
7.482
7.470
7.463
7.447
7.259
1.546
0.000
X : parts per Million : Proton

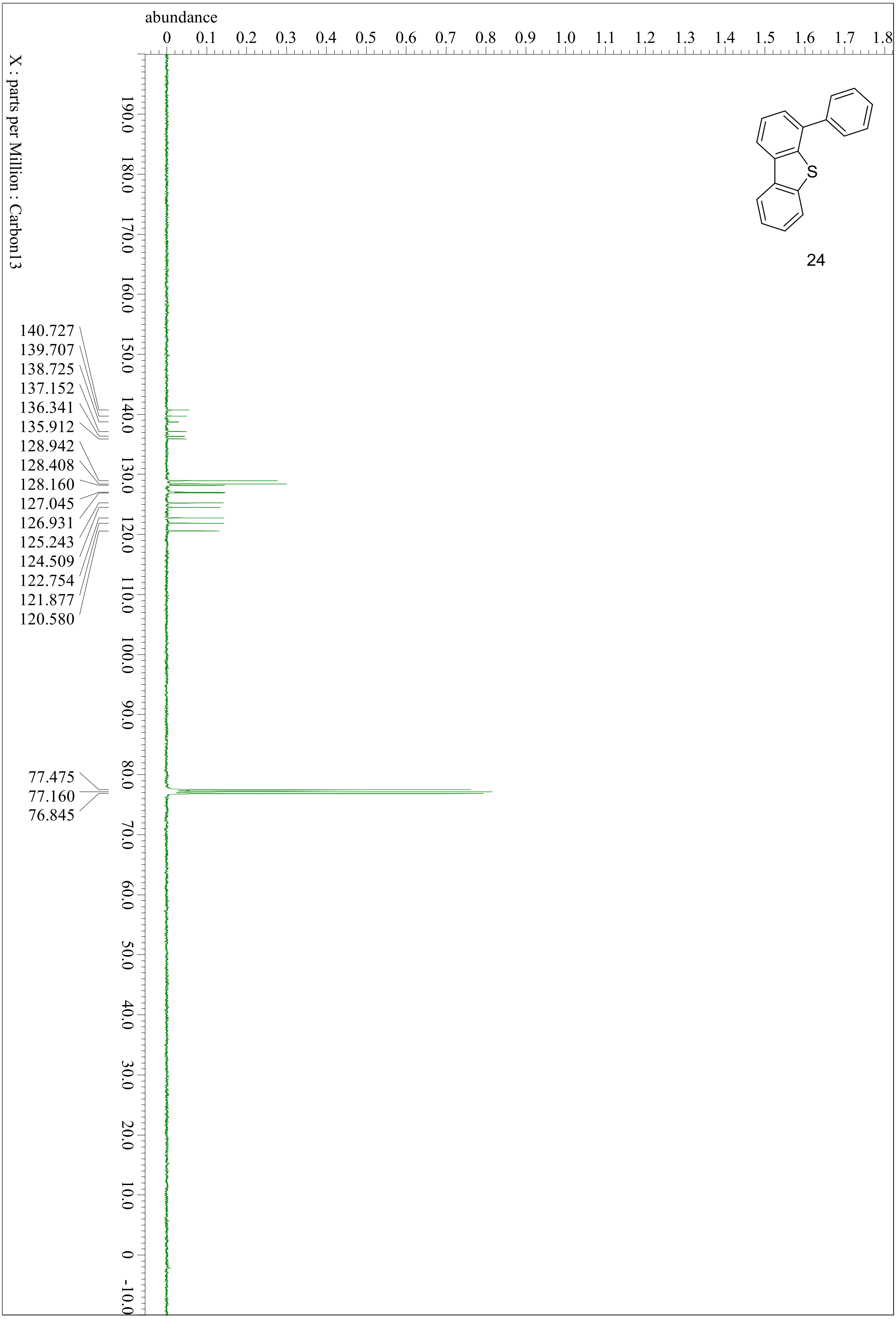
abundance
0 0.1 0.2 0.3 0.4 0.5 0.6 0.7 0.8 0.9 1.0 1.1 1.2 1.3 1.4 1.5 1.6 1.7 1.8
X : parts per Million : Carbon13
190.0 180.0 170.0 160.0 150.0 140.0 130.0 120.0 110.0 100.0 90.0 80.0 70.0 60.0 50.0 40.0 30.0 20.0 10.0 0 -10.0
140.727
139.707
138.725
137.152
136.341
135.912
128.942
128.408
128.160
127.045
126.931
125.243
124.509
122.754
121.877
120.580
77.475
77.160
76.845
S
24

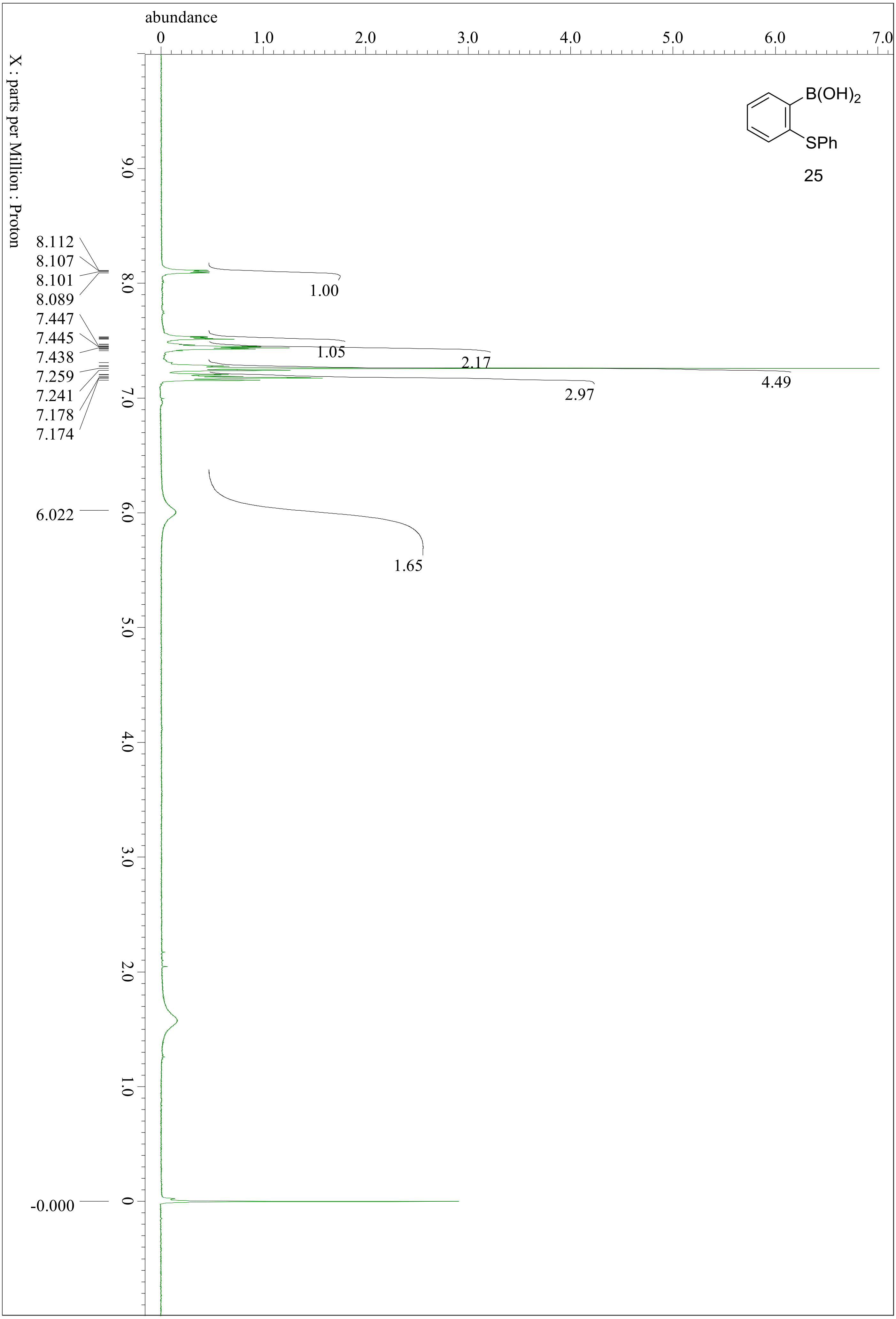
abundance
0
1.0
2.0
3.0
4.0
5.0
6.0
7.0
X : parts per Million : Proton
9.0
8.0
7.0
6.0
5.0
4.0
3.0
2.0
1.0
0
8.112
8.107
8.101
8.089
7.447
7.445
7.438
7.259
7.241
7.178
7.174
6.022
-0.000
1.00
1.05
2.17
4.49
2.97
1.65
B(OH)2
SPh
25

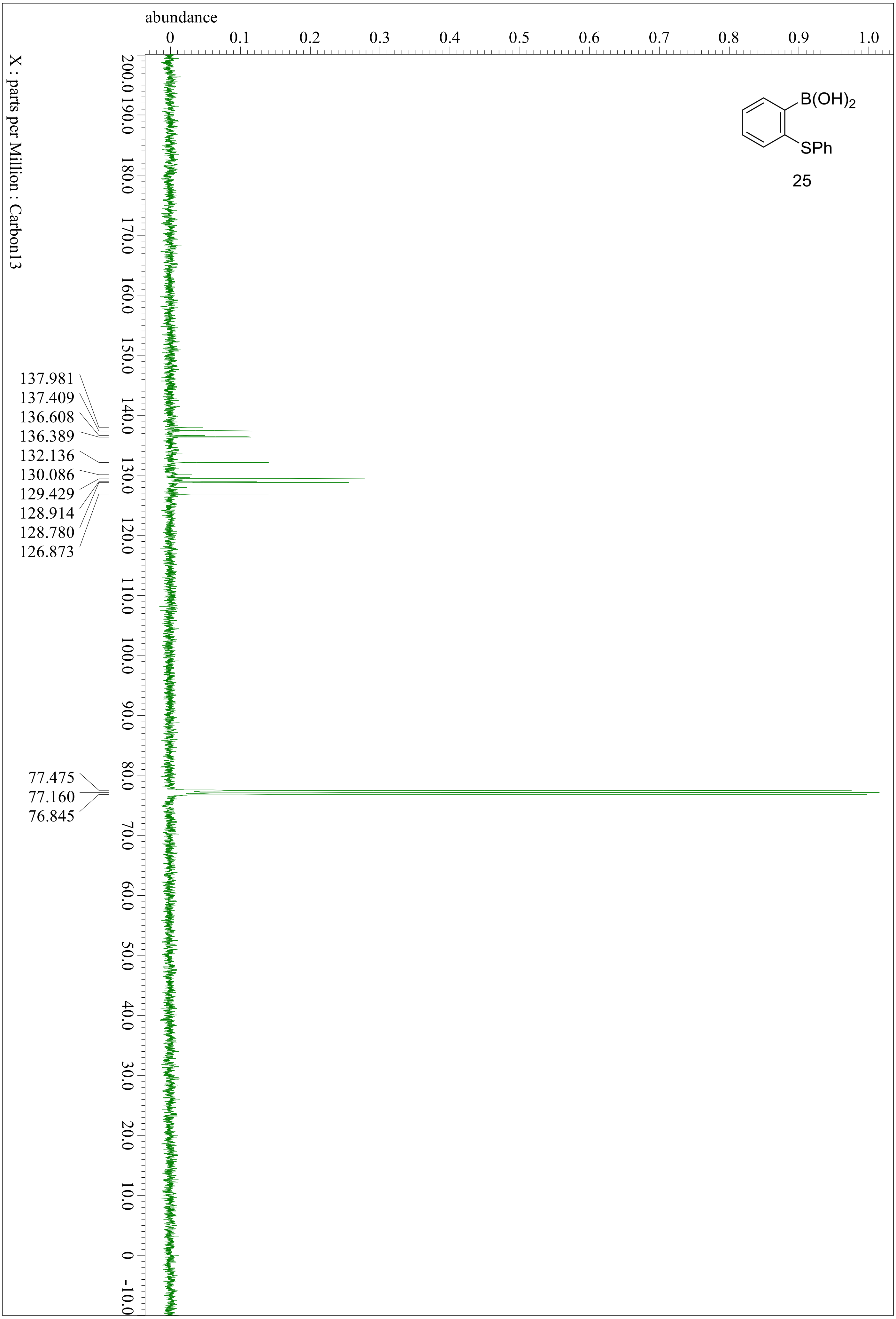
abundance
0 0.1 0.2 0.3 0.4 0.5 0.6 0.7 0.8 0.9 1.0
X : parts per Million : Carbon13
200.0 190.0 180.0 170.0 160.0 150.0 140.0 130.0 120.0 110.0 100.0 90.0 80.0 70.0 60.0 50.0 40.0 30.0 20.0 10.0 0 -10.0
137.981
137.409
136.608
136.389
132.136
130.086
129.429
128.914
128.780
126.873
77.475
77.160
76.845
B(OH)2
SPh
25

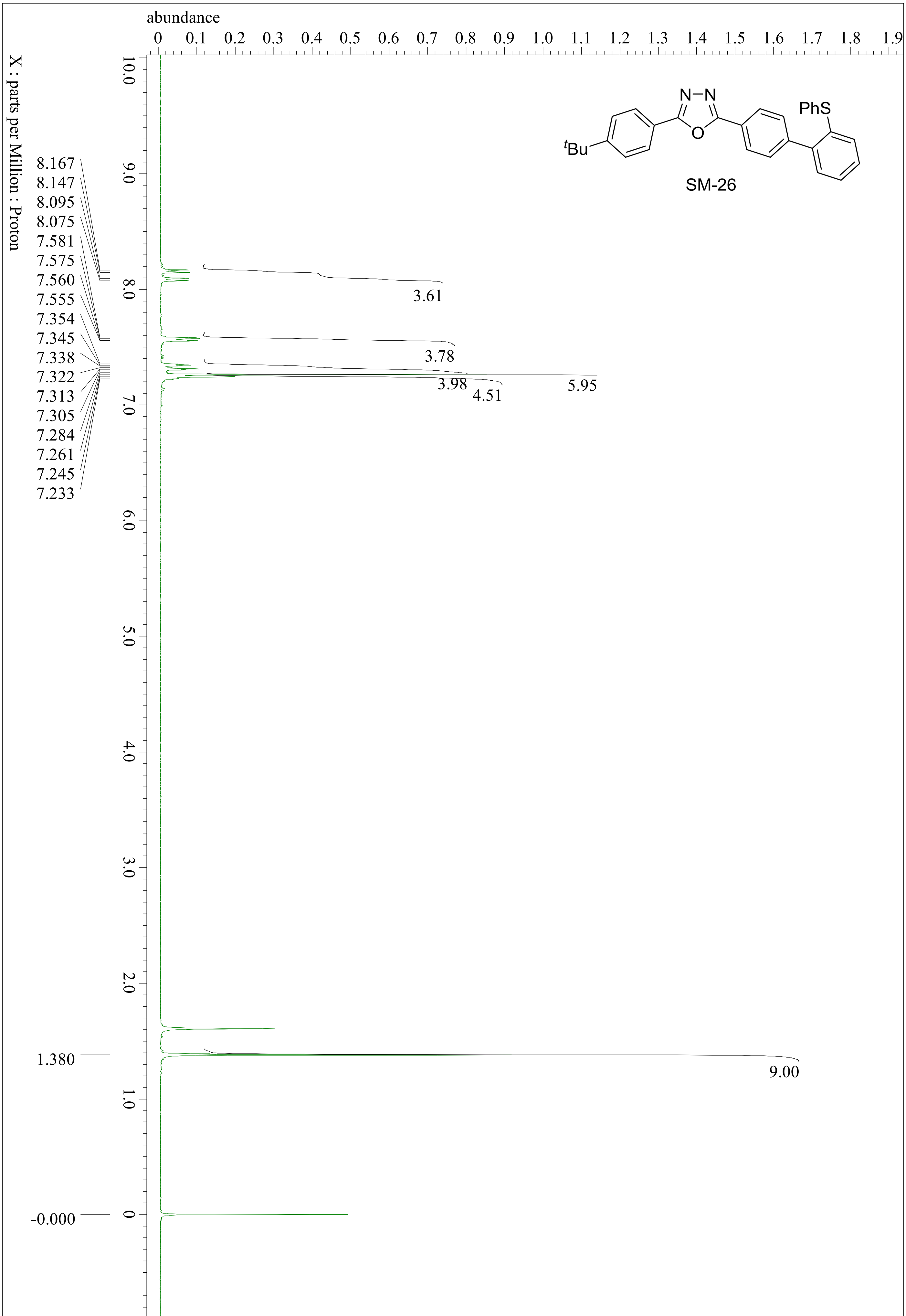
abundance
X : parts per Million : Proton
8.167
8.147
8.095
8.075
7.581
7.575
7.560
7.555
7.354
7.345
7.338
7.322
7.313
7.305
7.284
7.261
7.245
7.233
1.380
-0.000
3.61
3.78
3.98
4.51
5.95
9.00
N–N
O
PhS
tBu
SM-26

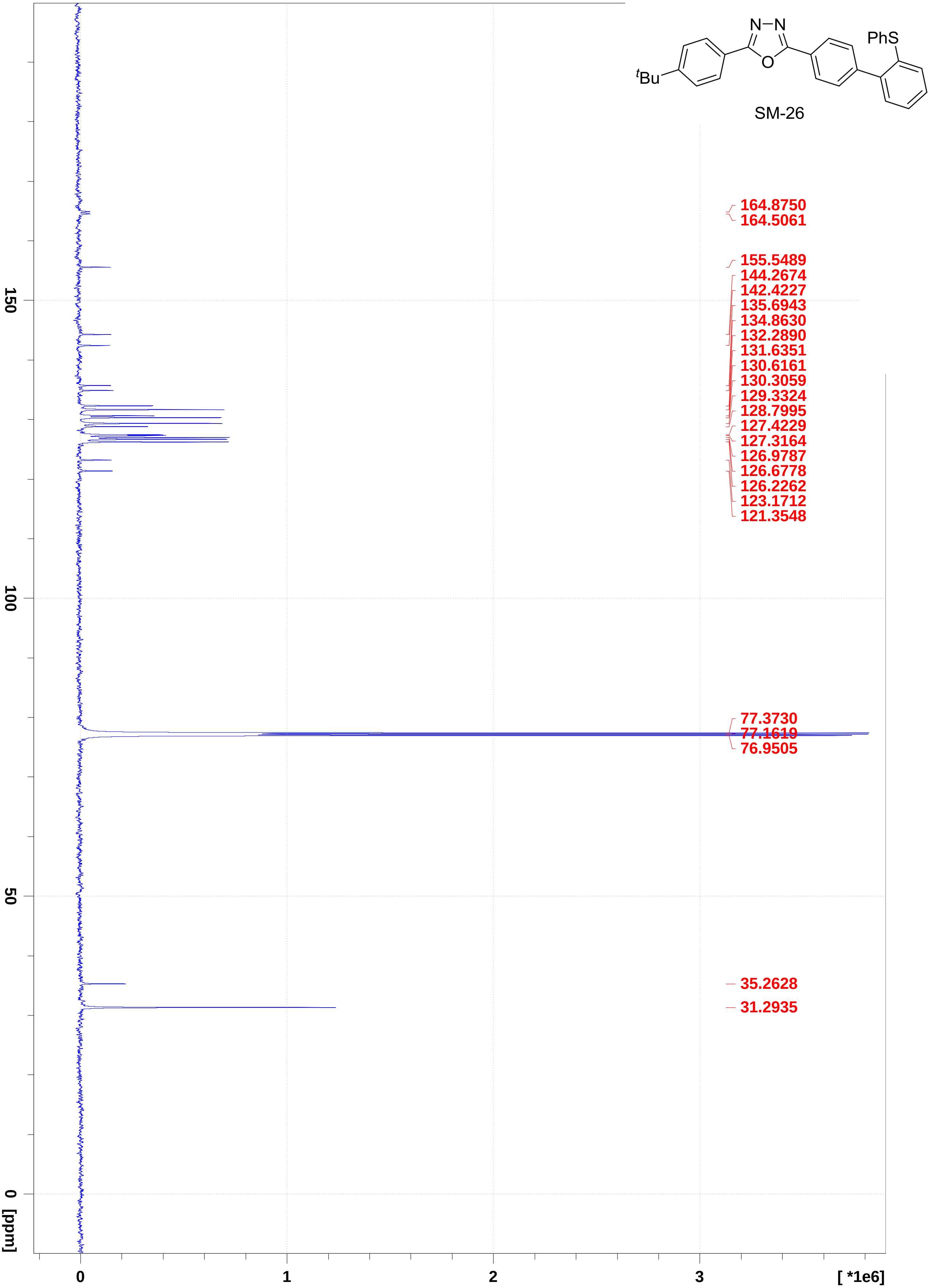
N–N
O
PhS
^{t}Bu
SM-26
164.8750
164.5061
155.5489
144.2674
142.4227
135.6943
134.8630
132.2890
131.6351
130.6161
130.3059
129.3324
128.7995
127.4229
127.3164
126.9787
126.6778
126.2262
123.1712
121.3548
77.3730
77.1619
76.9505
35.2628
31.2935
150
100
50
0
[ppm]
0
1
2
3
[*1e6]

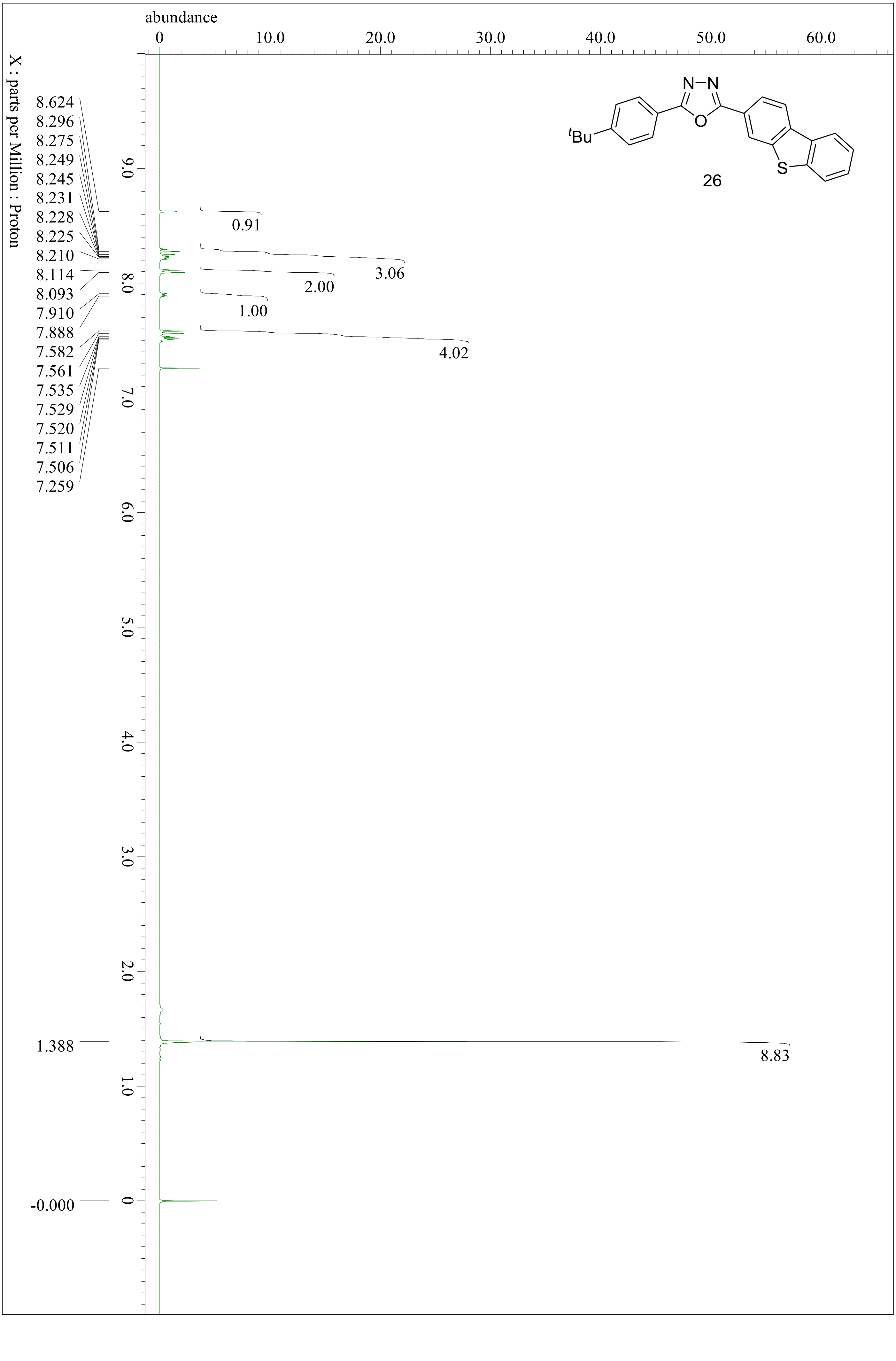
abundance
0
10.0
20.0
30.0
40.0
50.0
60.0
X : parts per Million : Proton
8.624
8.296
8.275
8.249
8.245
8.231
8.228
8.225
8.210
8.114
8.093
7.910
7.888
7.582
7.561
7.535
7.529
7.520
7.511
7.506
7.259
1.388
-0.000
9.0
8.0
7.0
6.0
5.0
4.0
3.0
2.0
1.0
0
0.91
3.06
2.00
1.00
4.02
8.83
N–N
O
S
tBu
26

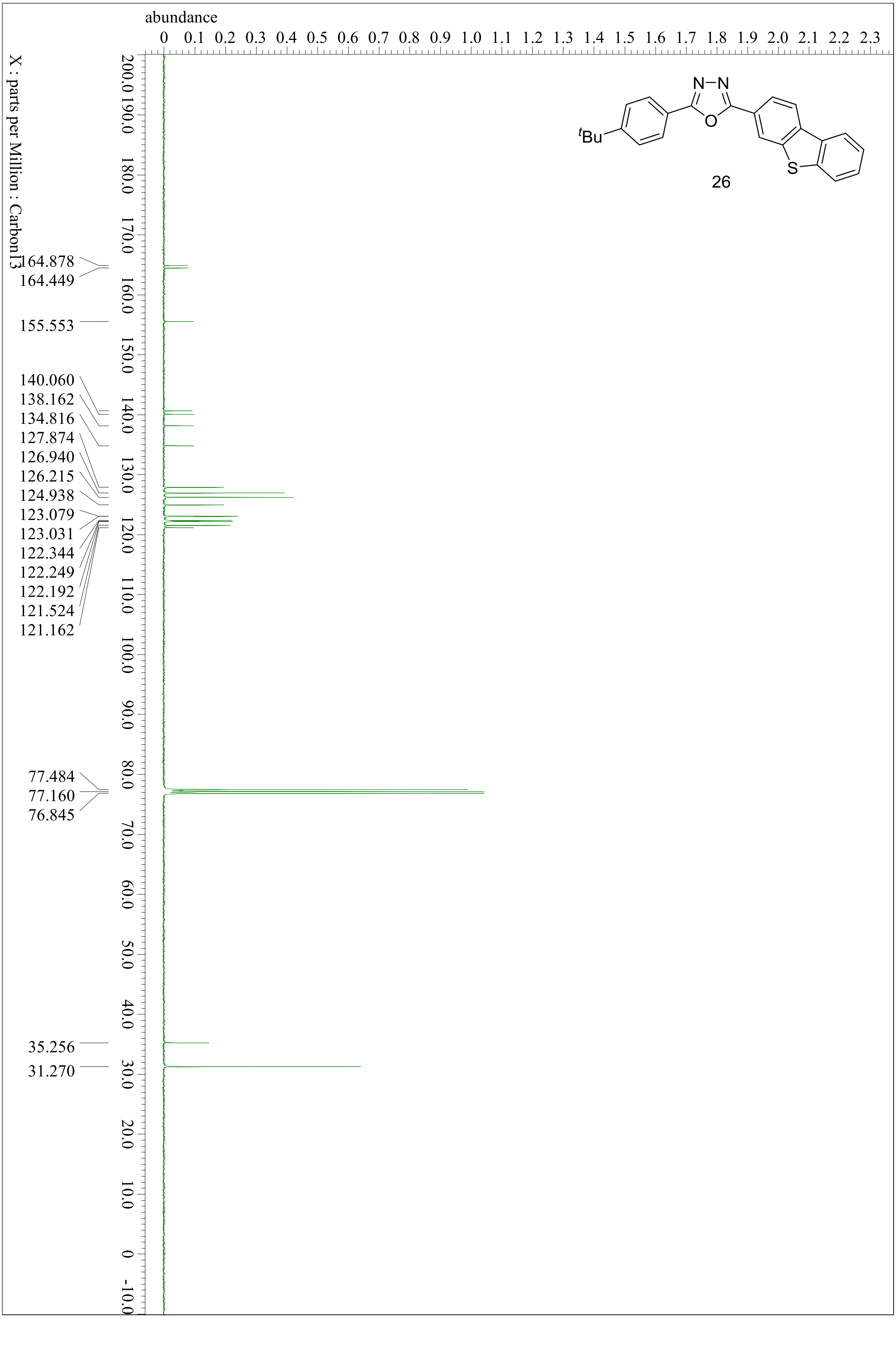
abundance
0 0.1 0.2 0.3 0.4 0.5 0.6 0.7 0.8 0.9 1.0 1.1 1.2 1.3 1.4 1.5 1.6 1.7 1.8 1.9 2.0 2.1 2.2 2.3
X : parts per Million : Carbon13
200.0 190.0 180.0 170.0 160.0 150.0 140.0 130.0 120.0 110.0 100.0 90.0 80.0 70.0 60.0 50.0 40.0 30.0 20.0 10.0 0 -10.0
164.878
164.449
155.553
140.060
138.162
134.816
127.874
126.940
126.215
124.938
123.079
123.031
122.344
122.249
122.192
121.524
121.162
77.484
77.160
76.845
35.256
31.270
N
N
O
S
^{t}Bu
26

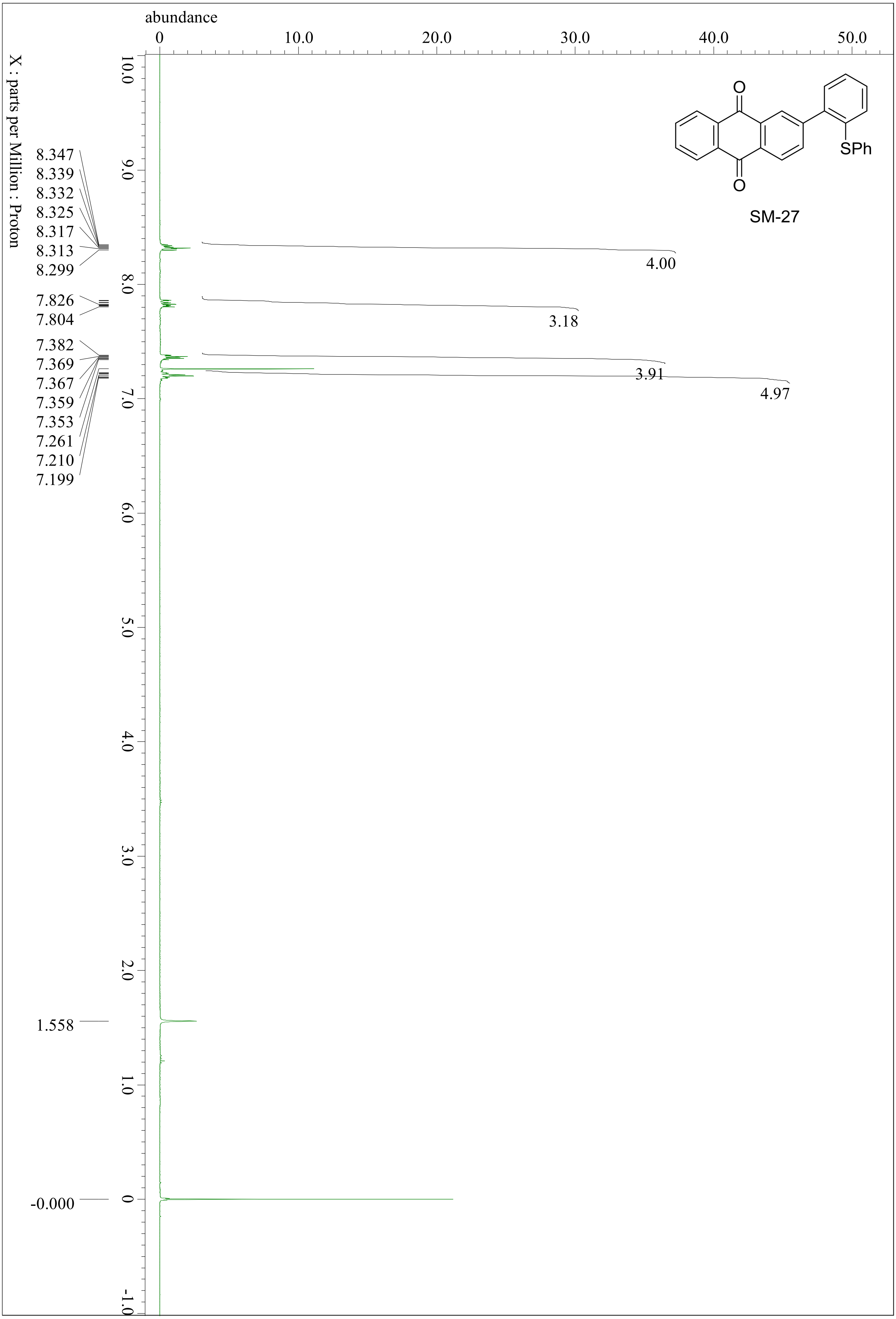
abundance
0
10.0
20.0
30.0
40.0
50.0
X : parts per Million : Proton
10.0
9.0
8.0
7.0
6.0
5.0
4.0
3.0
2.0
1.0
0
-1.0
8.347
8.339
8.332
8.325
8.317
8.313
8.299
7.826
7.804
7.382
7.369
7.367
7.359
7.353
7.261
7.210
7.199
1.558
-0.000
4.00
3.18
3.91
4.97
O
O
SPh
SM-27

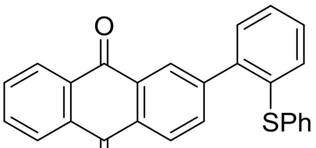
O
O
SPh

SM-27

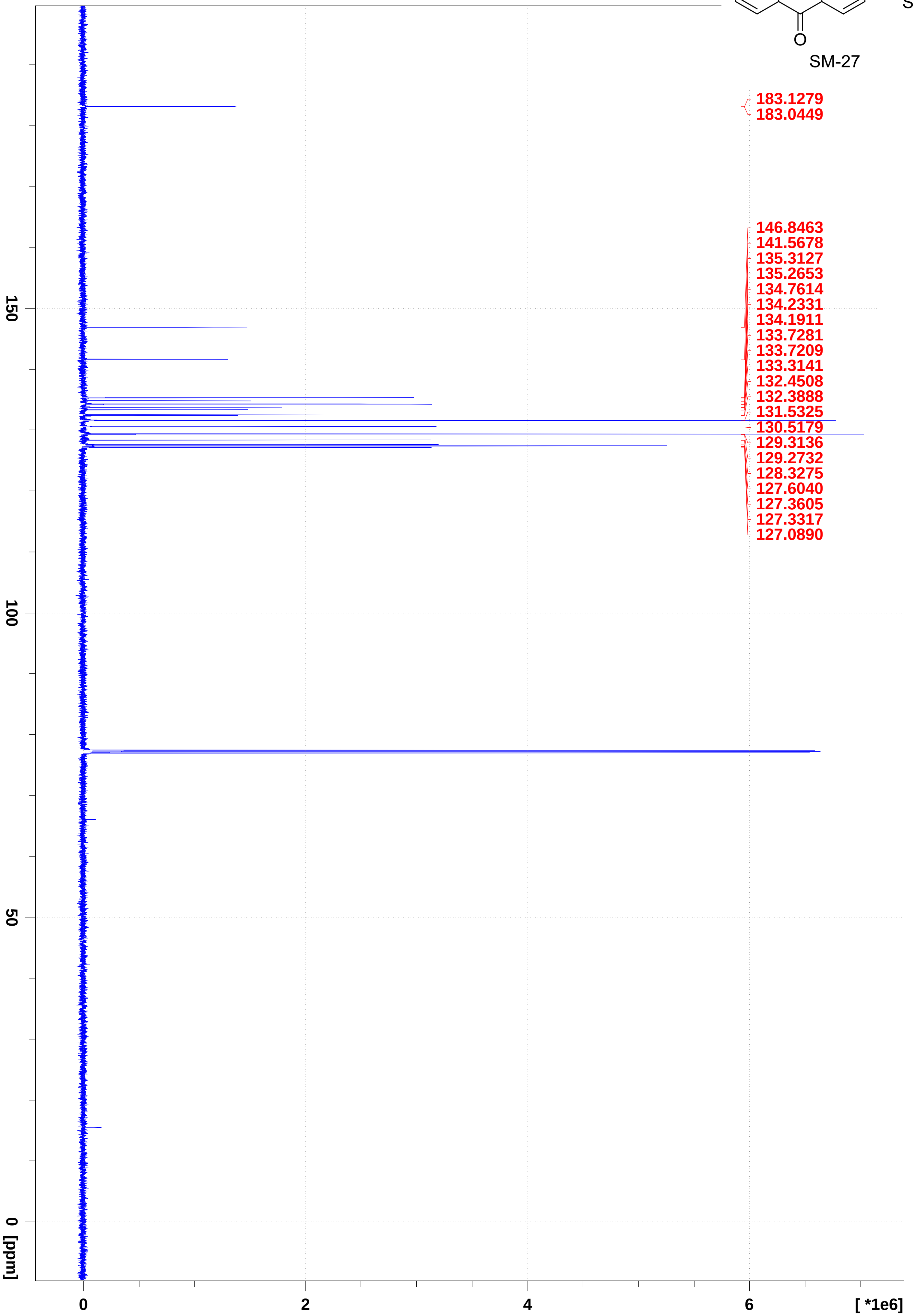
183.1279
183.0449
146.8463
141.5678
135.3127
135.2653
134.7614
134.2331
134.1911
133.7281
133.7209
133.3141
132.4508
132.3888
131.5325
130.5179
129.3136
129.2732
128.3275
127.6040
127.3605
127.3317
127.0890
150
100
50
0
[ppm]
0
2
4
6
[*1e6]

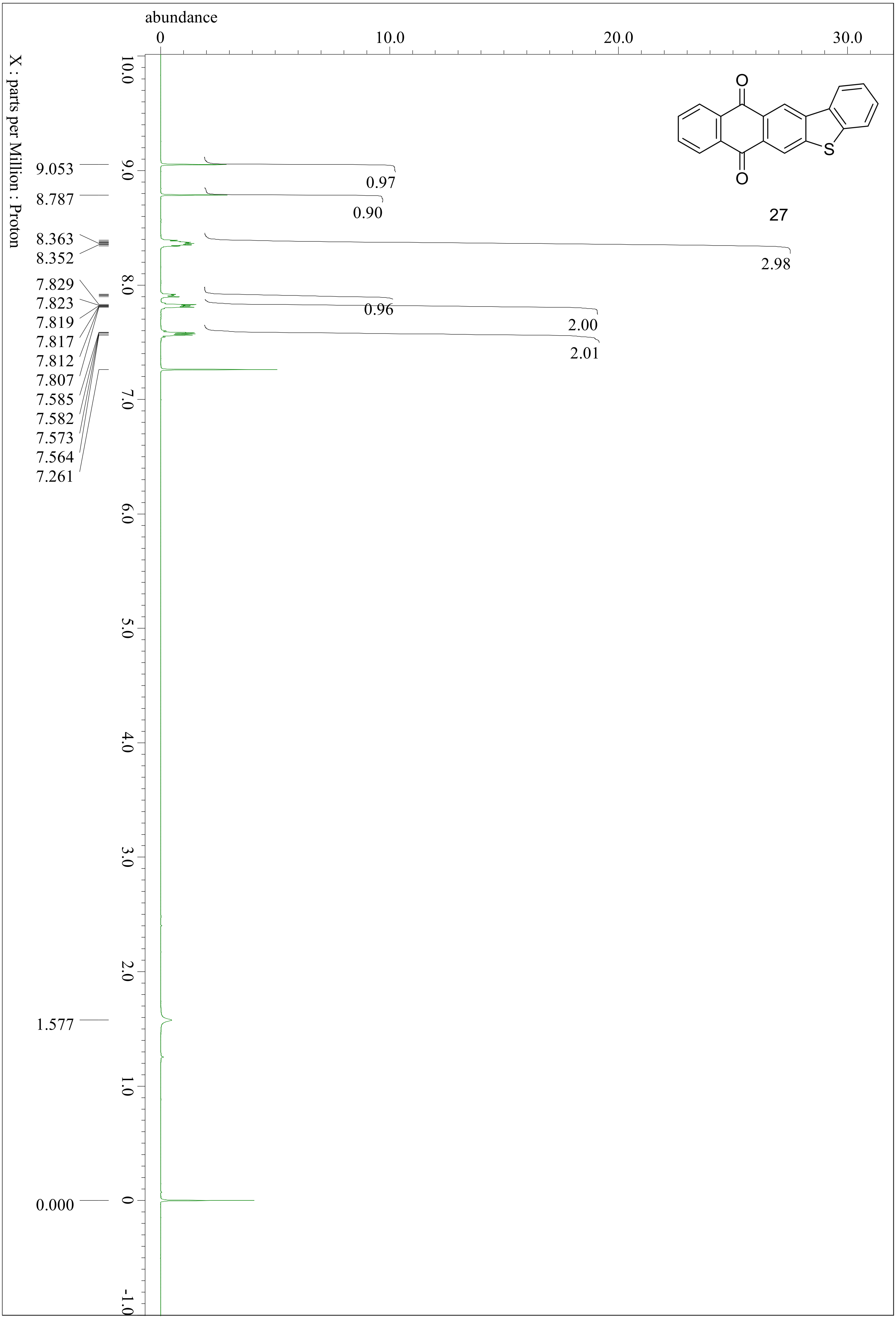

abundance
0
10.0
20.0
30.0
X : parts per Million : Proton
9.053
8.787
8.363
8.352
7.829
7.823
7.819
7.817
7.812
7.807
7.585
7.582
7.573
7.564
7.261
1.577
0.000
0.97
0.90
2.98
0.96
2.00
2.01
O
O
S
27

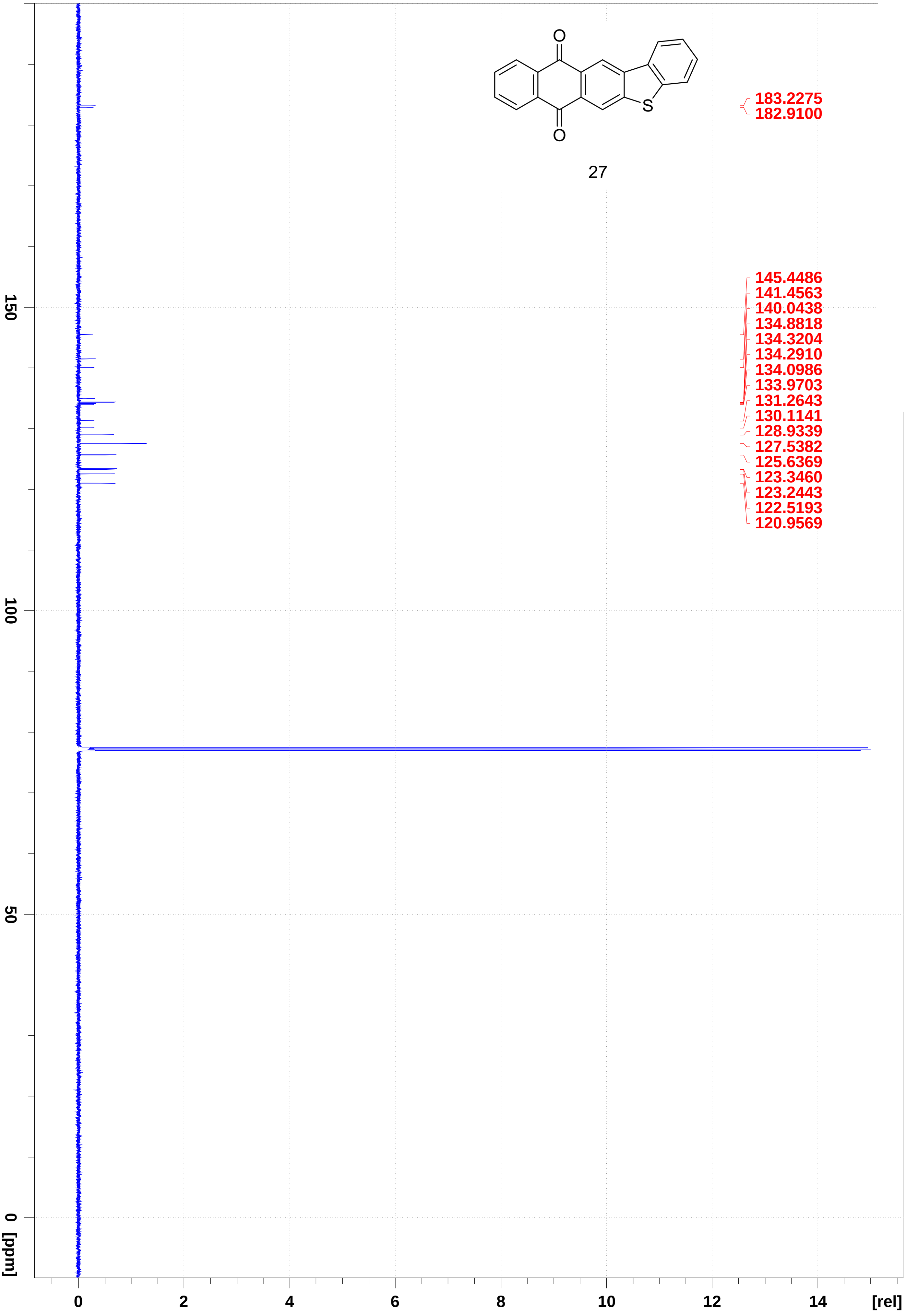
27
183.2275
182.9100
145.4486
141.4563
140.0438
134.8818
134.3204
134.2910
134.0986
133.9703
131.2643
130.1141
128.9339
127.5382
125.6369
123.3460
123.2443
122.5193
120.9569
150
100
50
0
[ppm]
0
2
4
6
8
10
12
14
[rel]

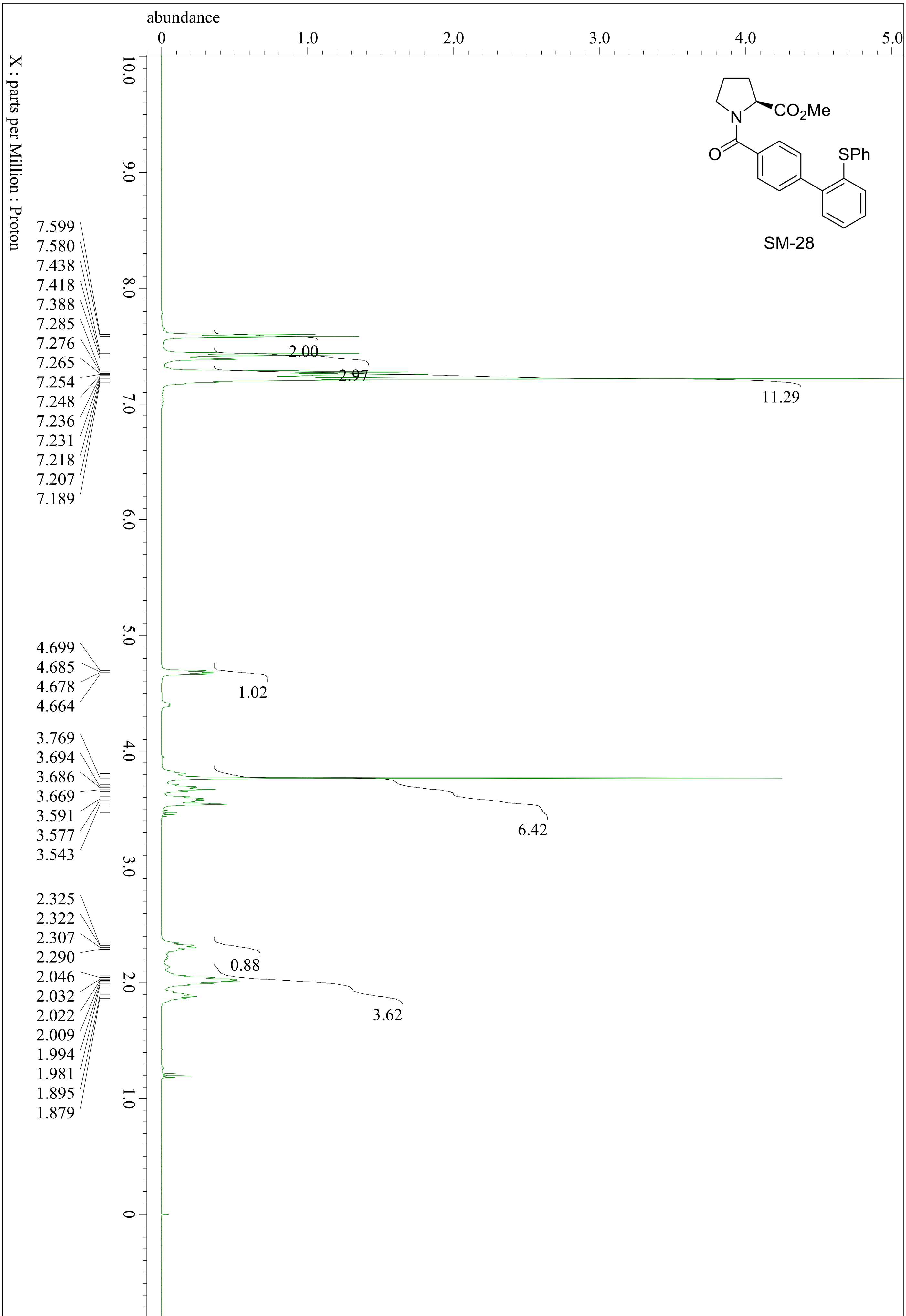

X : parts per Million : Proton
abundance
0
1.0
2.0
3.0
4.0
5.0
10.0
9.0
8.0
7.0
6.0
5.0
4.0
3.0
2.0
1.0
0
7.599
7.580
7.438
7.418
7.388
7.285
7.276
7.265
7.254
7.248
7.236
7.231
7.218
7.207
7.189
4.699
4.685
4.678
4.664
3.769
3.694
3.686
3.669
3.591
3.577
3.543
2.325
2.322
2.307
2.290
2.046
2.032
2.022
2.009
1.994
1.981
1.895
1.879
2.00
2.97
11.29
1.02
6.42
0.88
3.62
CO2Me
N
O
SPh
SM-28

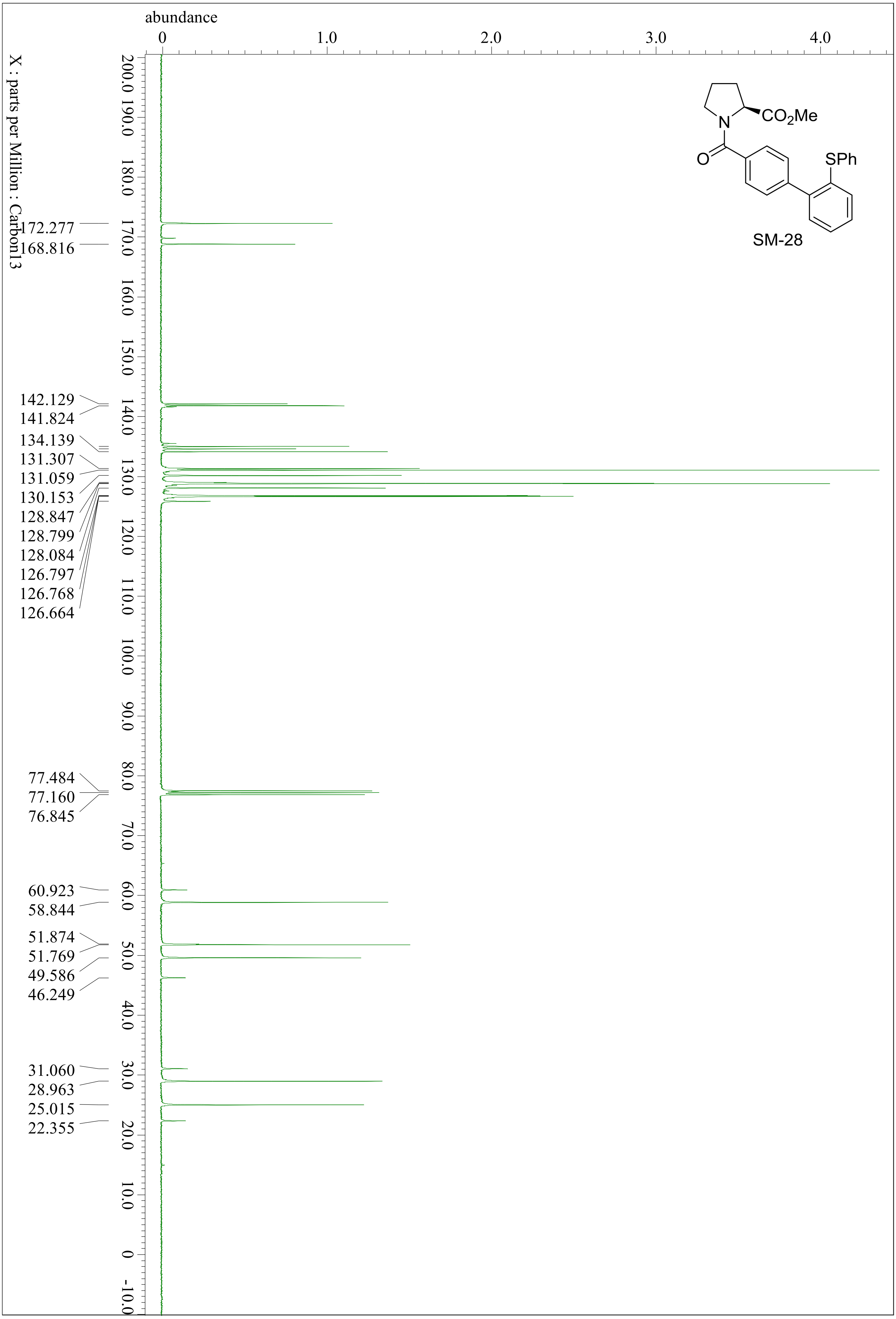

abundance
0
1.0
2.0
3.0
4.0
X : parts per Million : Carbon13
200.0
190.0
180.0
170.0
160.0
150.0
140.0
130.0
120.0
110.0
100.0
90.0
80.0
70.0
60.0
50.0
40.0
30.0
20.0
10.0
0
-10.0
172.277
168.816
142.129
141.824
134.139
131.307
131.059
130.153
128.847
128.799
128.084
126.797
126.768
126.664
77.484
77.160
76.845
60.923
58.844
51.874
51.769
49.586
46.249
31.060
28.963
25.015
22.355
CO2Me
N
O
SPh
SM-28

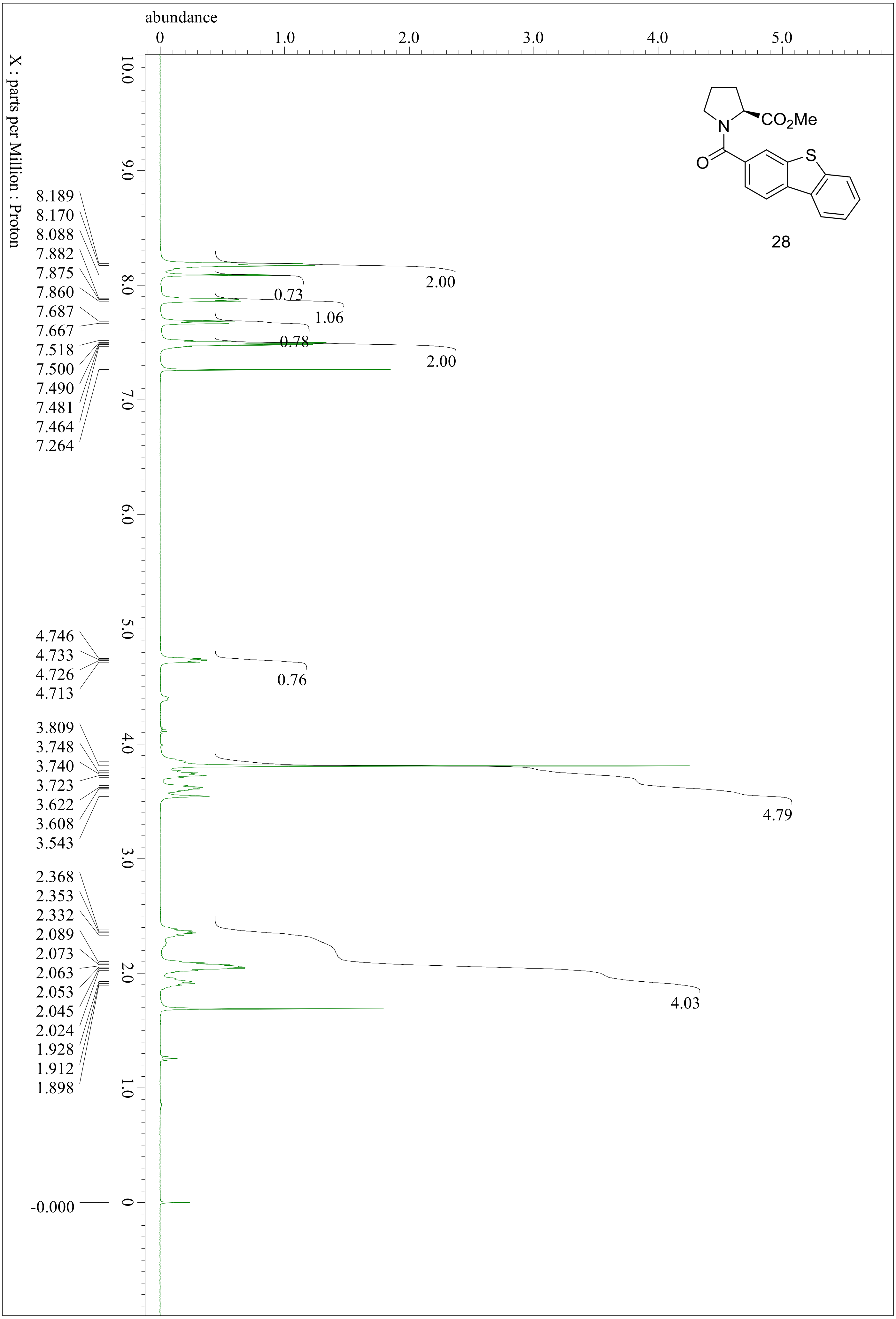
abundance
0
1.0
2.0
3.0
4.0
5.0
X : parts per Million : Proton
10.0
9.0
8.0
7.0
6.0
5.0
4.0
3.0
2.0
1.0
0
8.189
8.170
8.088
7.882
7.875
7.860
7.687
7.667
7.518
7.500
7.490
7.481
7.464
7.264
4.746
4.733
4.726
4.713
3.809
3.748
3.740
3.723
3.622
3.608
3.543
2.368
2.353
2.332
2.089
2.073
2.063
2.053
2.045
2.024
1.928
1.912
1.898
-0.000
2.00
0.73
1.06
0.78
2.00
0.76
4.79
4.03
CO_2Me
28

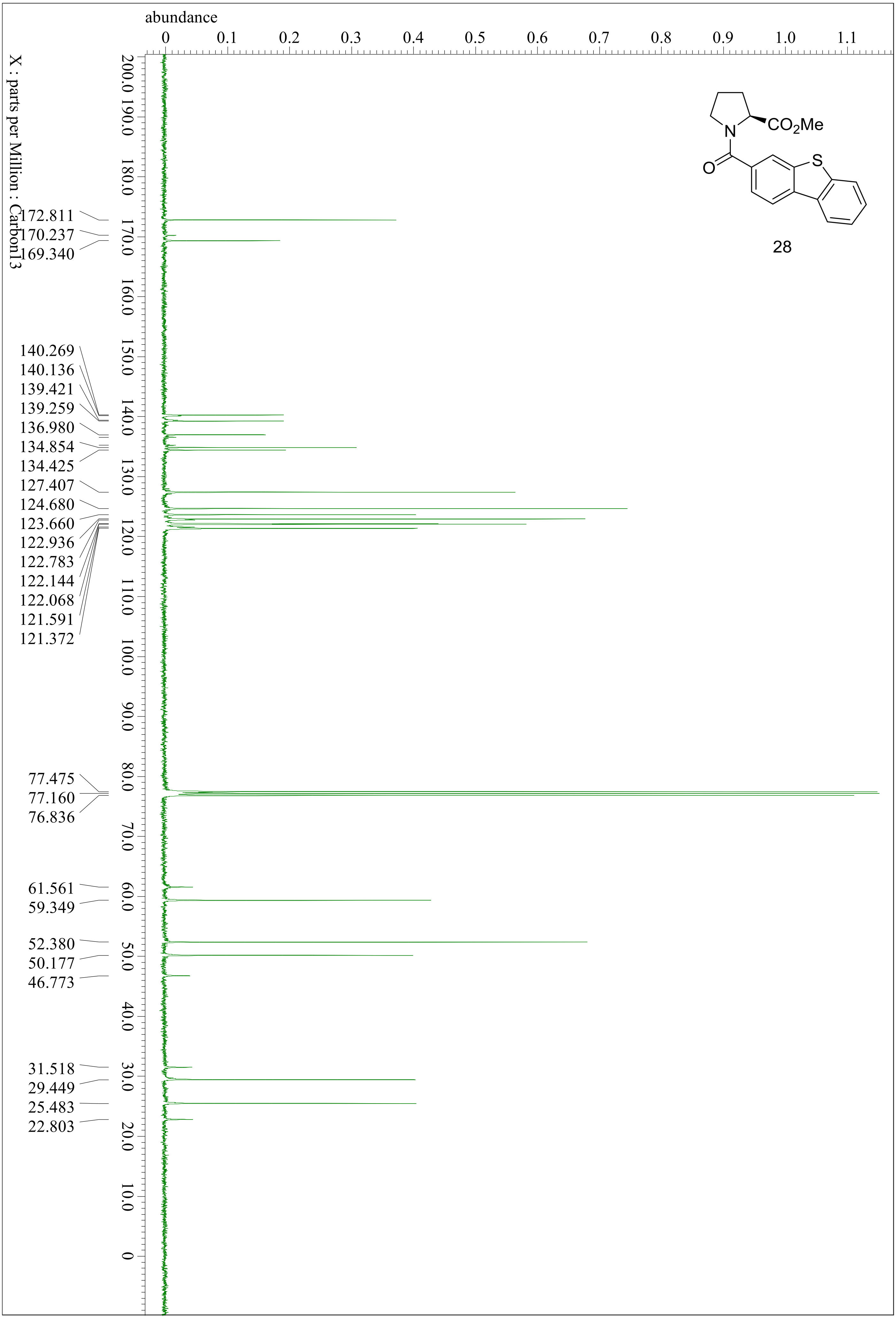

abundance
X : parts per Million : Carbon13
CO2Me
N
O
S
28
172.811
170.237
169.340
140.269
140.136
139.421
139.259
136.980
134.854
134.425
127.407
124.680
123.660
122.936
122.783
122.144
122.068
121.591
121.372
77.475
77.160
76.836
61.561
59.349
52.380
50.177
46.773
31.518
29.449
25.483
22.803

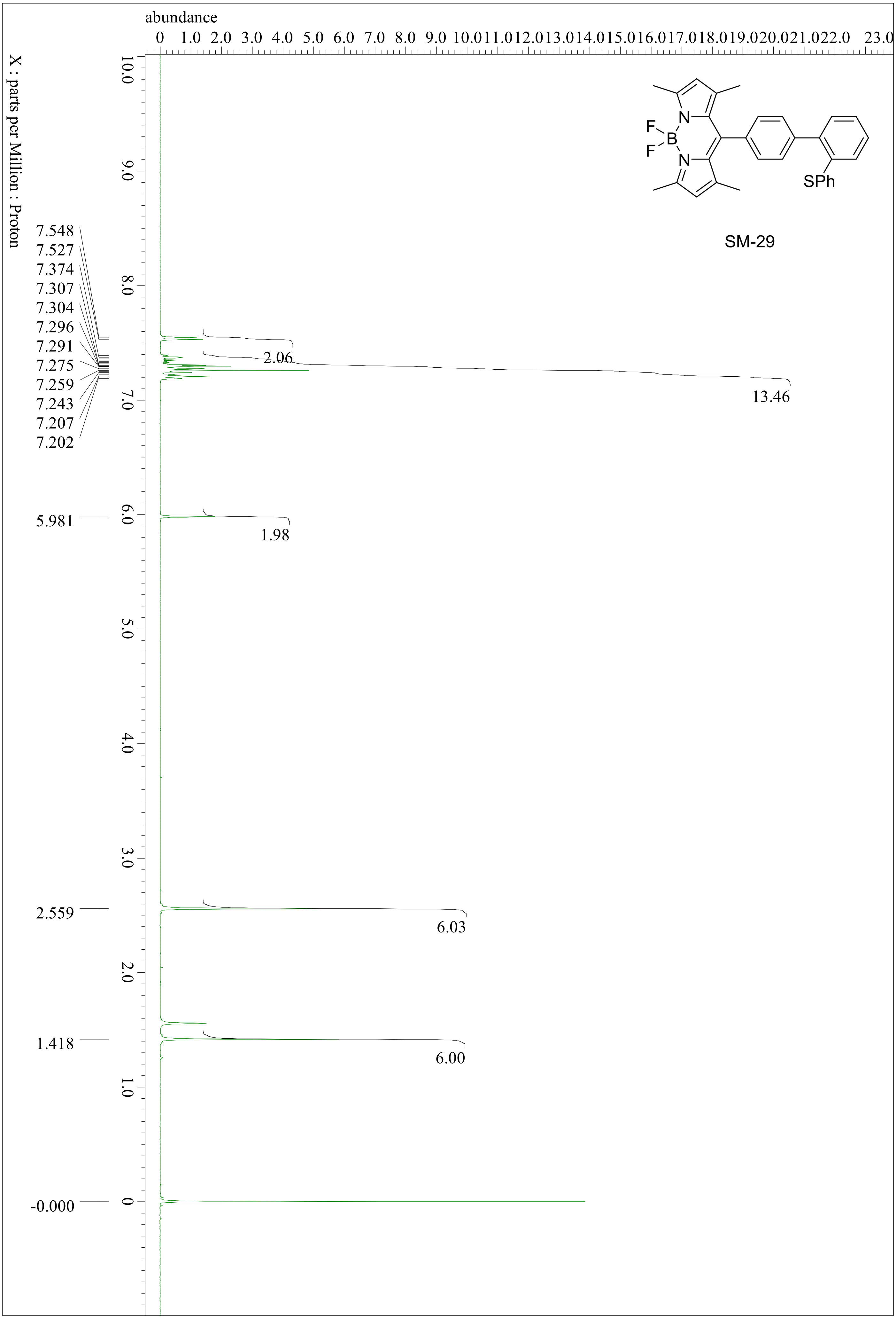
abundance
0 1.0 2.0 3.0 4.0 5.0 6.0 7.0 8.0 9.0 10.0 11.0 12.0 13.0 14.0 15.0 16.0 17.0 18.0 19.0 20.0 21.0 22.0 23.0
X : parts per Million : Proton
10.0 9.0 8.0 7.0 6.0 5.0 4.0 3.0 2.0 1.0 0
7.548
7.527
7.374
7.307
7.304
7.296
7.291
7.275
7.259
7.243
7.207
7.202
5.981
2.559
1.418
-0.000
2.06
13.46
1.98
6.03
6.00
F
B
F
N
N
SPh
SM-29

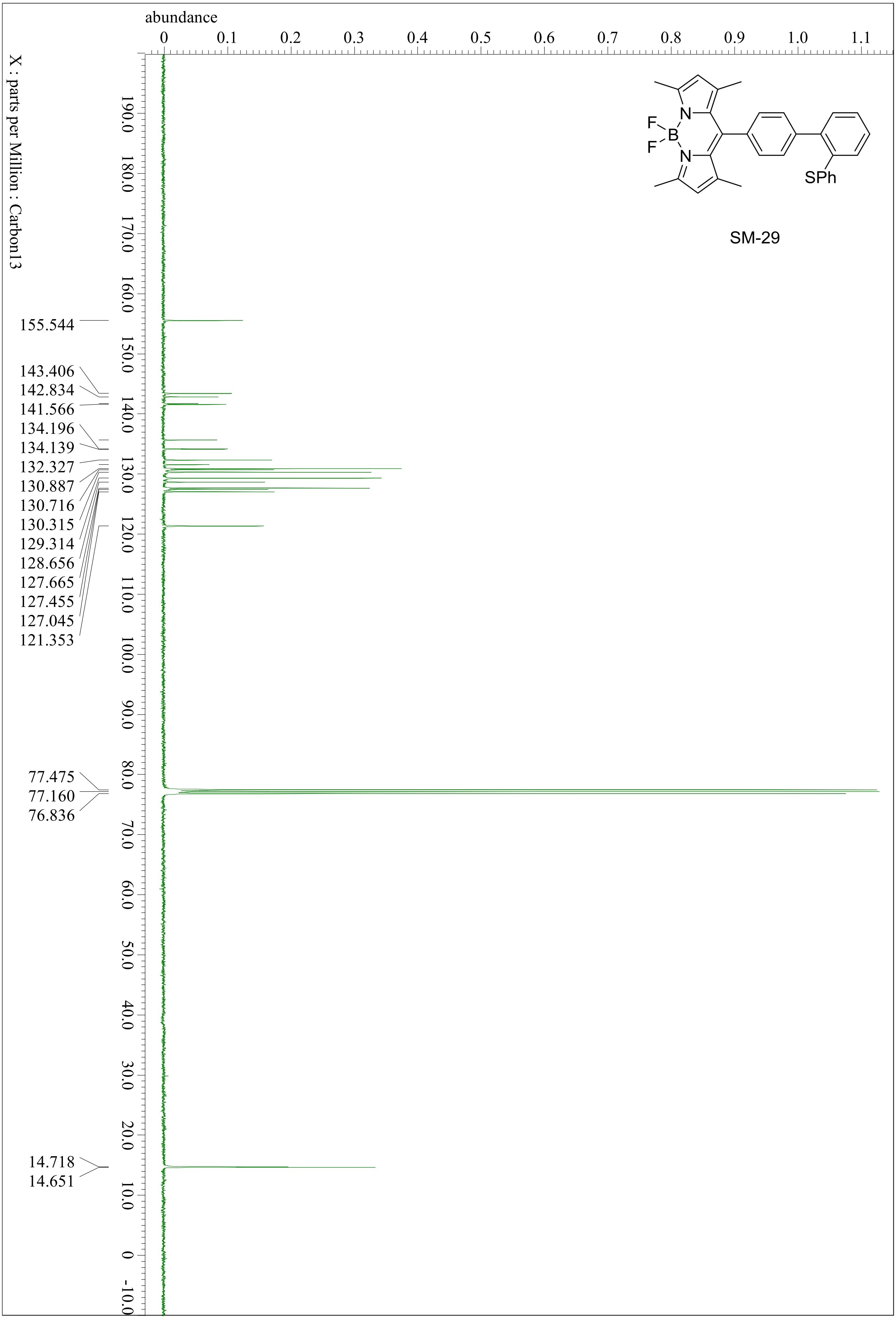

abundance
0
0.1
0.2
0.3
0.4
0.5
0.6
0.7
0.8
0.9
1.0
1.1
X : parts per Million : Carbon13
190.0
180.0
170.0
160.0
150.0
140.0
130.0
120.0
110.0
100.0
90.0
80.0
70.0
60.0
50.0
40.0
30.0
20.0
10.0
0
-10.0
155.544
143.406
142.834
141.566
134.196
134.139
132.327
130.887
130.716
130.315
129.314
128.656
127.665
127.455
127.045
121.353
77.475
77.160
76.836
14.718
14.651
F
F
B
N
N
SPh
SM-29

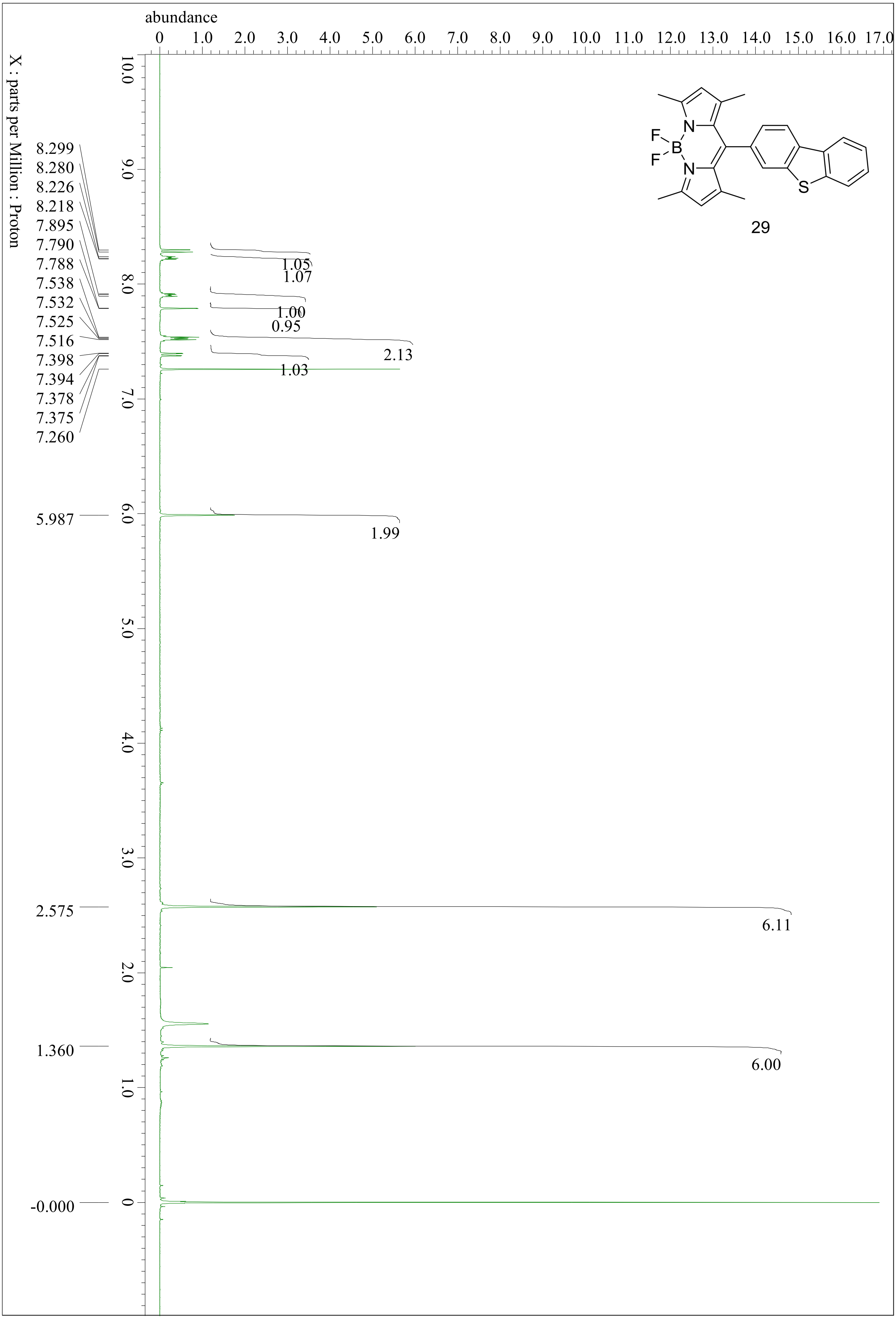

abundance
X : parts per Million : Proton
8.299
8.280
8.226
8.218
7.895
7.790
7.788
7.538
7.532
7.525
7.516
7.398
7.394
7.378
7.375
7.260
5.987
2.575
1.360
-0.000
1.05
1.07
1.00
0.95
2.13
1.03
1.99
6.11
6.00
F
B
N
S
29

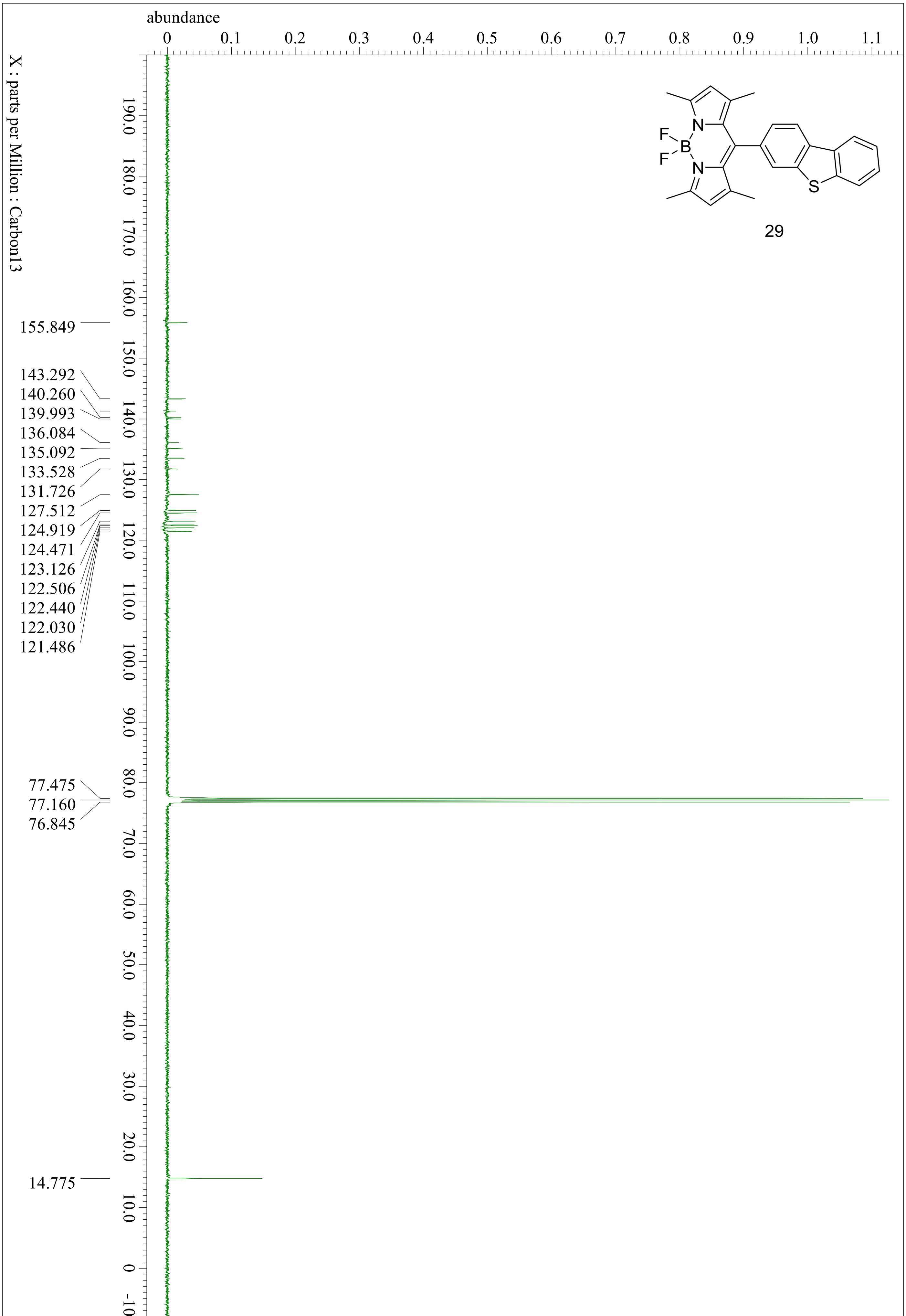

abundance
0 0.1 0.2 0.3 0.4 0.5 0.6 0.7 0.8 0.9 1.0 1.1
X : parts per Million : Carbon13
190.0 180.0 170.0 160.0 150.0 140.0 130.0 120.0 110.0 100.0 90.0 80.0 70.0 60.0 50.0 40.0 30.0 20.0 10.0 0 -10.0
155.849
143.292
140.260
139.993
136.084
135.092
133.528
131.726
127.512
124.919
124.471
123.126
122.506
122.440
122.030
121.486
77.475
77.160
76.845
14.775
F
B
F
N
N
S
29

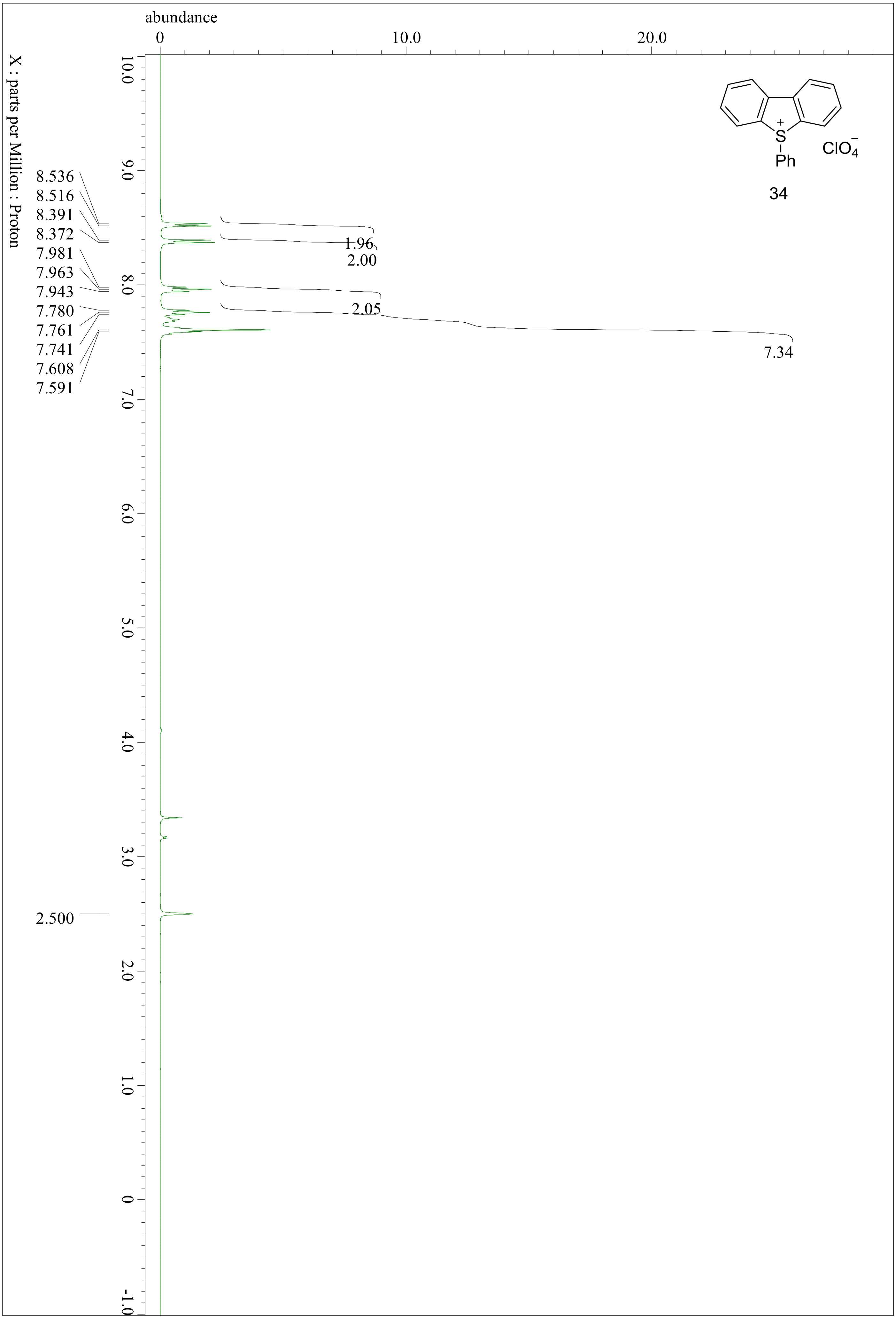
abundance
0
10.0
20.0
X : parts per Million : Proton
10.0
9.0
8.0
7.0
6.0
5.0
4.0
3.0
2.0
1.0
0
-1.0
8.536
8.516
8.391
8.372
7.981
7.963
7.943
7.780
7.761
7.741
7.608
7.591
2.500
1.96
2.00
2.05
7.34
S
Ph
ClO_4^-
34

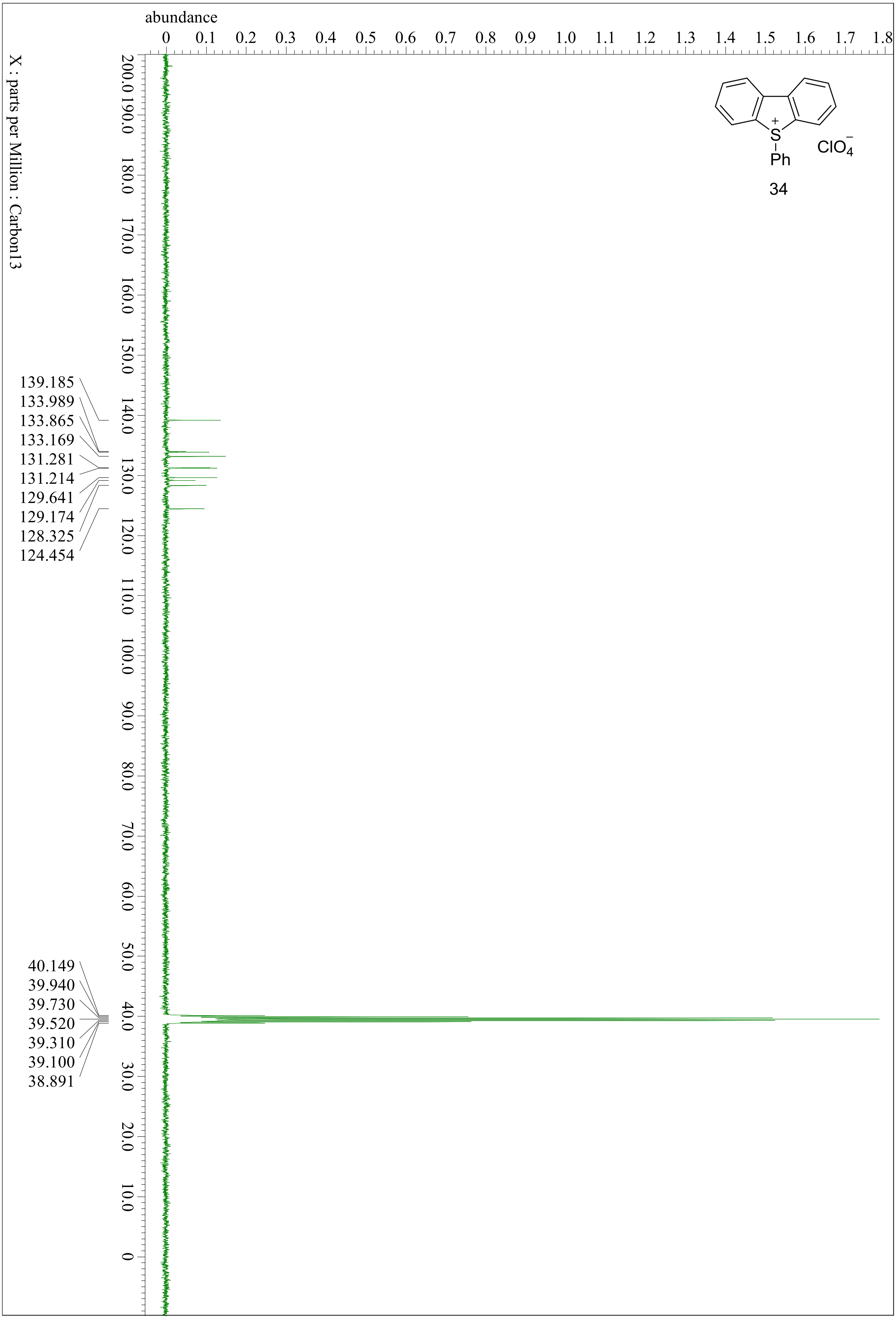

abundance
0 0.1 0.2 0.3 0.4 0.5 0.6 0.7 0.8 0.9 1.0 1.1 1.2 1.3 1.4 1.5 1.6 1.7 1.8
X : parts per Million : Carbon13
200.0 190.0 180.0 170.0 160.0 150.0 140.0 130.0 120.0 110.0 100.0 90.0 80.0 70.0 60.0 50.0 40.0 30.0 20.0 10.0 0
139.185
133.989
133.865
133.169
131.281
131.214
129.641
129.174
128.325
124.454
40.149
39.940
39.730
39.520
39.310
39.100
38.891
S
Ph
ClO_4^-
34

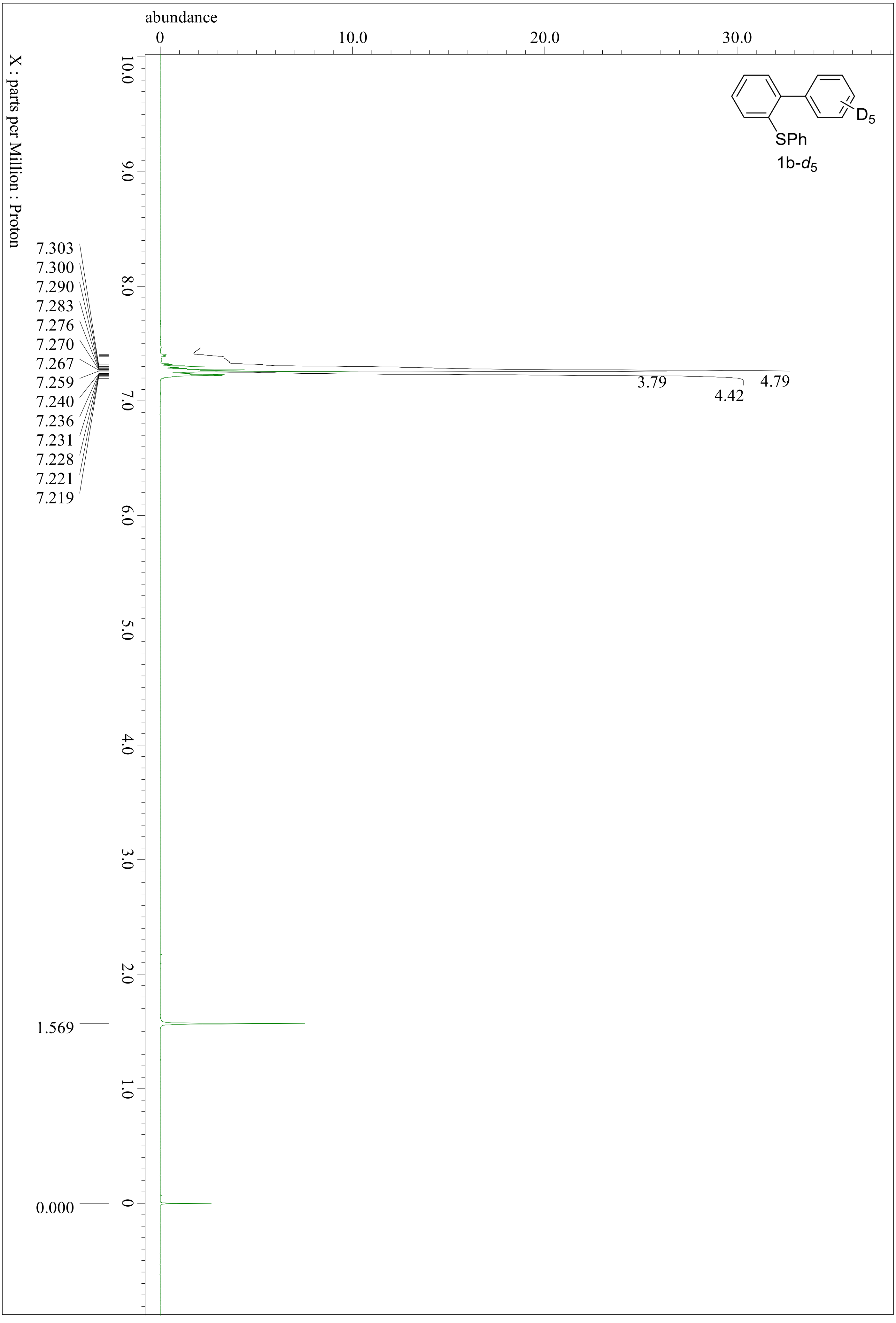
abundance
0
10.0
20.0
30.0
X : parts per Million : Proton
SPh
D5
1b-d5
7.303
7.300
7.290
7.283
7.276
7.270
7.267
7.259
7.240
7.236
7.231
7.228
7.221
7.219
1.569
0.000
3.79
4.42
4.79
10.0
9.0
8.0
7.0
6.0
5.0
4.0
3.0
2.0
1.0
0

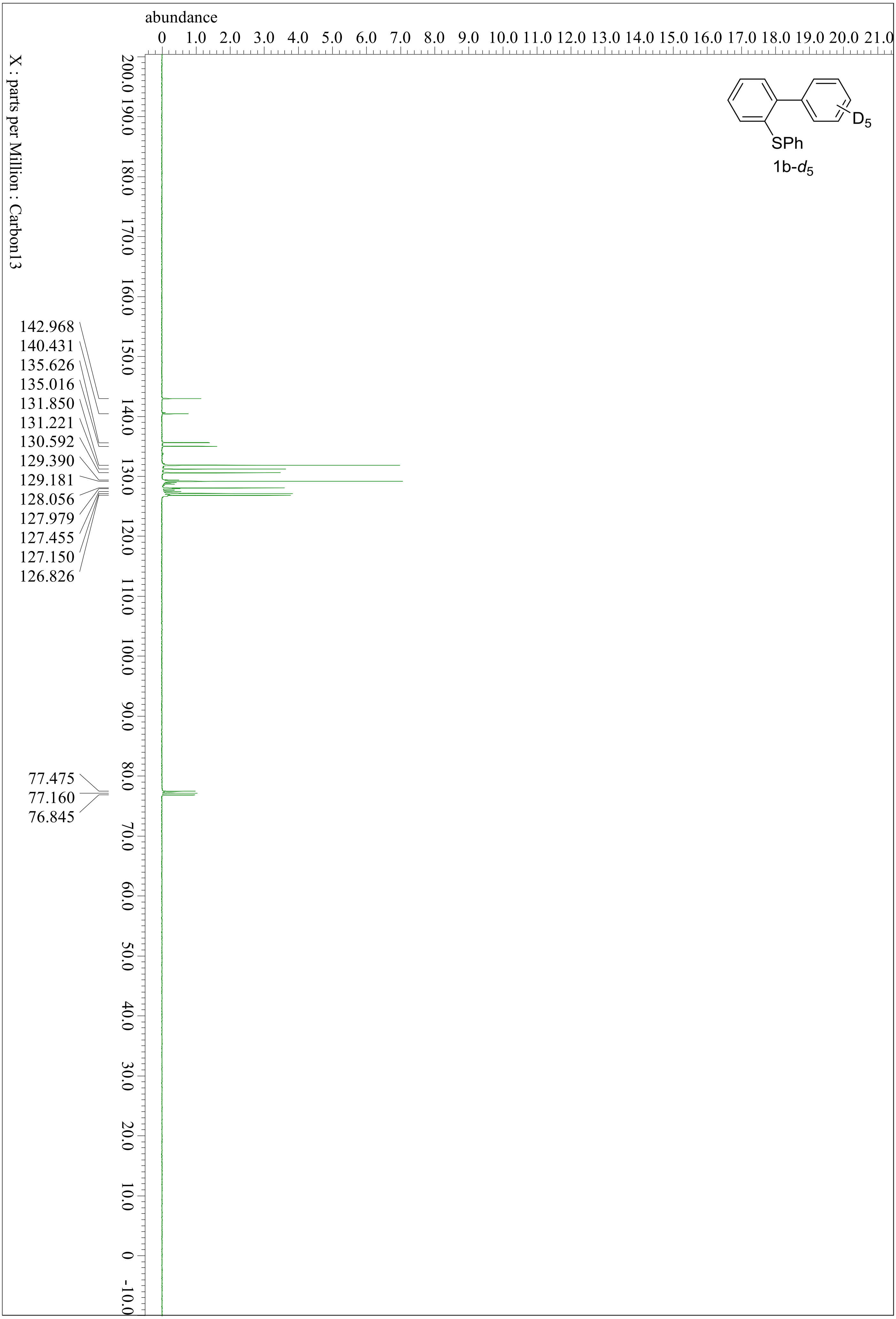
abundance
0 1.0 2.0 3.0 4.0 5.0 6.0 7.0 8.0 9.0 10.0 11.0 12.0 13.0 14.0 15.0 16.0 17.0 18.0 19.0 20.0 21.0
X : parts per Million : Carbon13
200.0 190.0 180.0 170.0 160.0 150.0 140.0 130.0 120.0 110.0 100.0 90.0 80.0 70.0 60.0 50.0 40.0 30.0 20.0 10.0 0 -10.0
142.968
140.431
135.626
135.016
131.850
131.221
130.592
129.390
129.181
128.056
127.979
127.455
127.150
126.826
77.475
77.160
76.845
D5
SPh
1b-d5

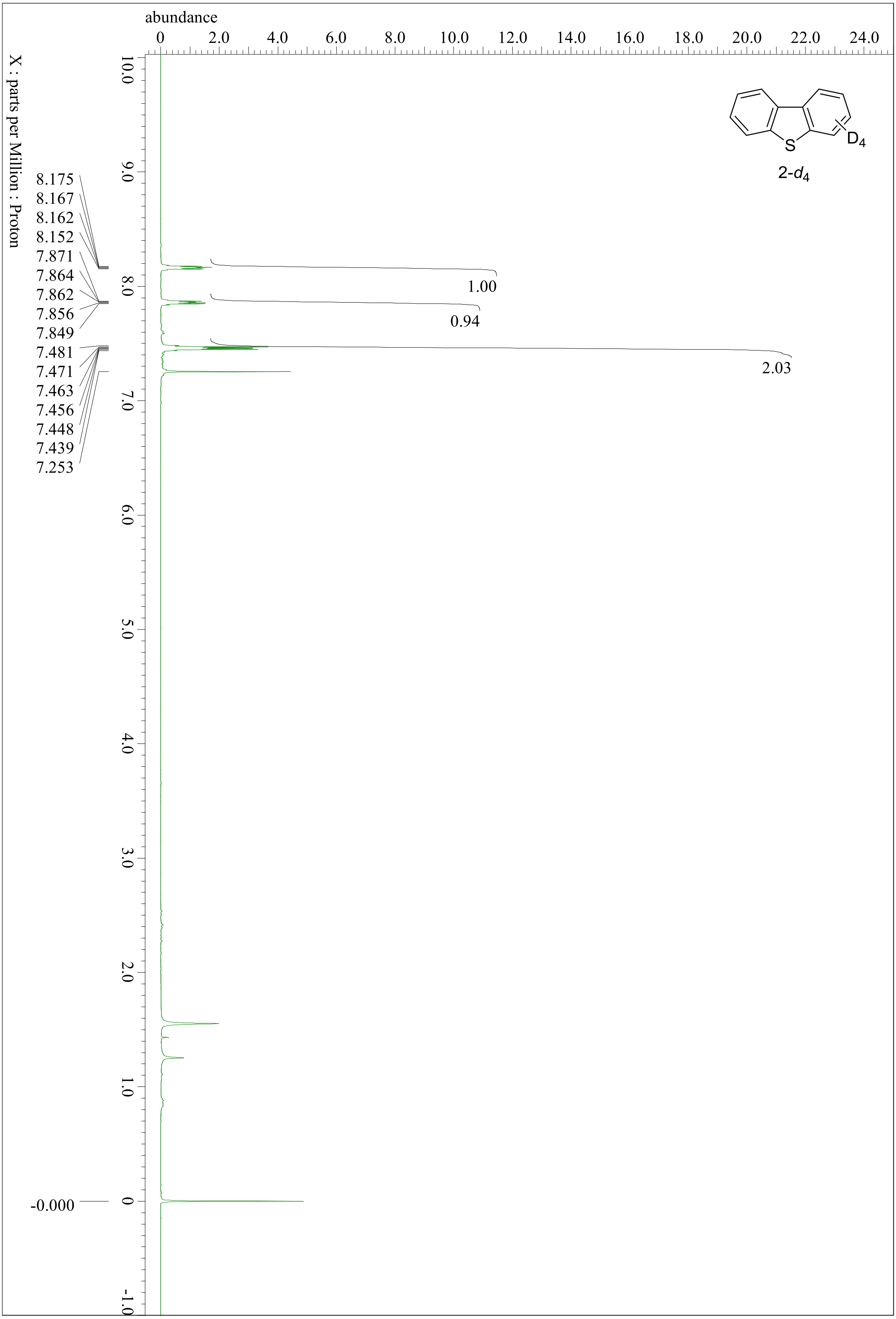
abundance
0 2.0 4.0 6.0 8.0 10.0 12.0 14.0 16.0 18.0 20.0 22.0 24.0
X : parts per Million : Proton
10.0 9.0 8.0 7.0 6.0 5.0 4.0 3.0 2.0 1.0 0 -1.0
8.175
8.167
8.162
8.152
7.871
7.864
7.862
7.856
7.849
7.481
7.471
7.463
7.456
7.448
7.439
7.253
-0.000
1.00
0.94
2.03
S
D4
2-d4

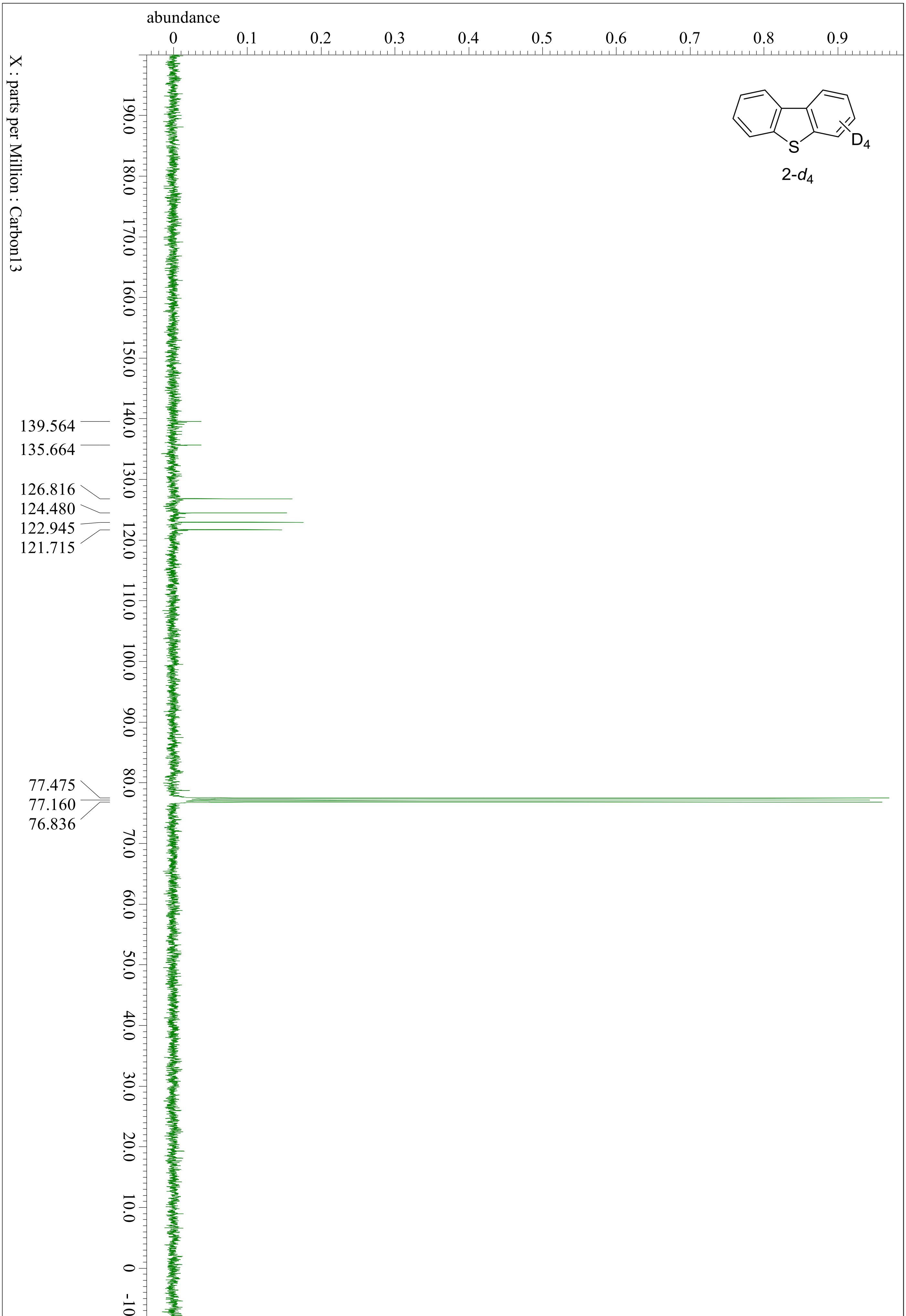

abundance
0
0.1
0.2
0.3
0.4
0.5
0.6
0.7
0.8
0.9
X : parts per Million : Carbon13
190.0
180.0
170.0
160.0
150.0
140.0
130.0
120.0
110.0
100.0
90.0
80.0
70.0
60.0
50.0
40.0
30.0
20.0
10.0
0
-10.0
139.564
135.664
126.816
124.480
122.945
121.715
77.475
77.160
76.836
S
D4
2-d4

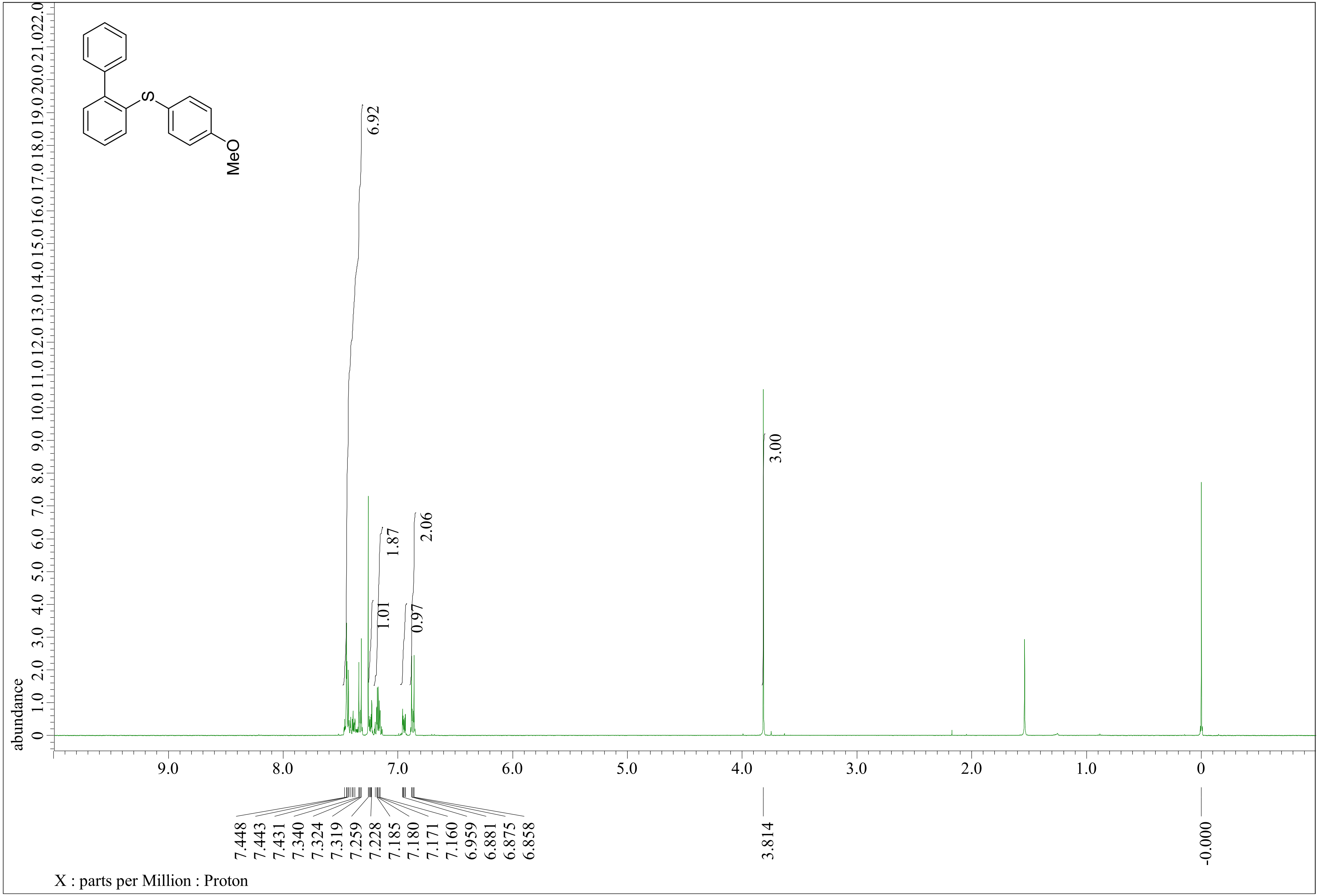

abundance
0
1.0
2.0
3.0
4.0
5.0
6.0
7.0
8.0
9.0
10.0
11.0
12.0
13.0
14.0
15.0
16.0
17.0
18.0
19.0
20.0
21.0
22.0
S
MeO
6.92
1.01
1.87
0.97
2.06
3.00
9.0
8.0
7.0
6.0
5.0
4.0
3.0
2.0
1.0
0
7.448
7.443
7.431
7.340
7.324
7.319
7.259
7.228
7.185
7.180
7.171
7.160
6.959
6.881
6.875
6.858
3.814
-0.000
X : parts per Million : Proton

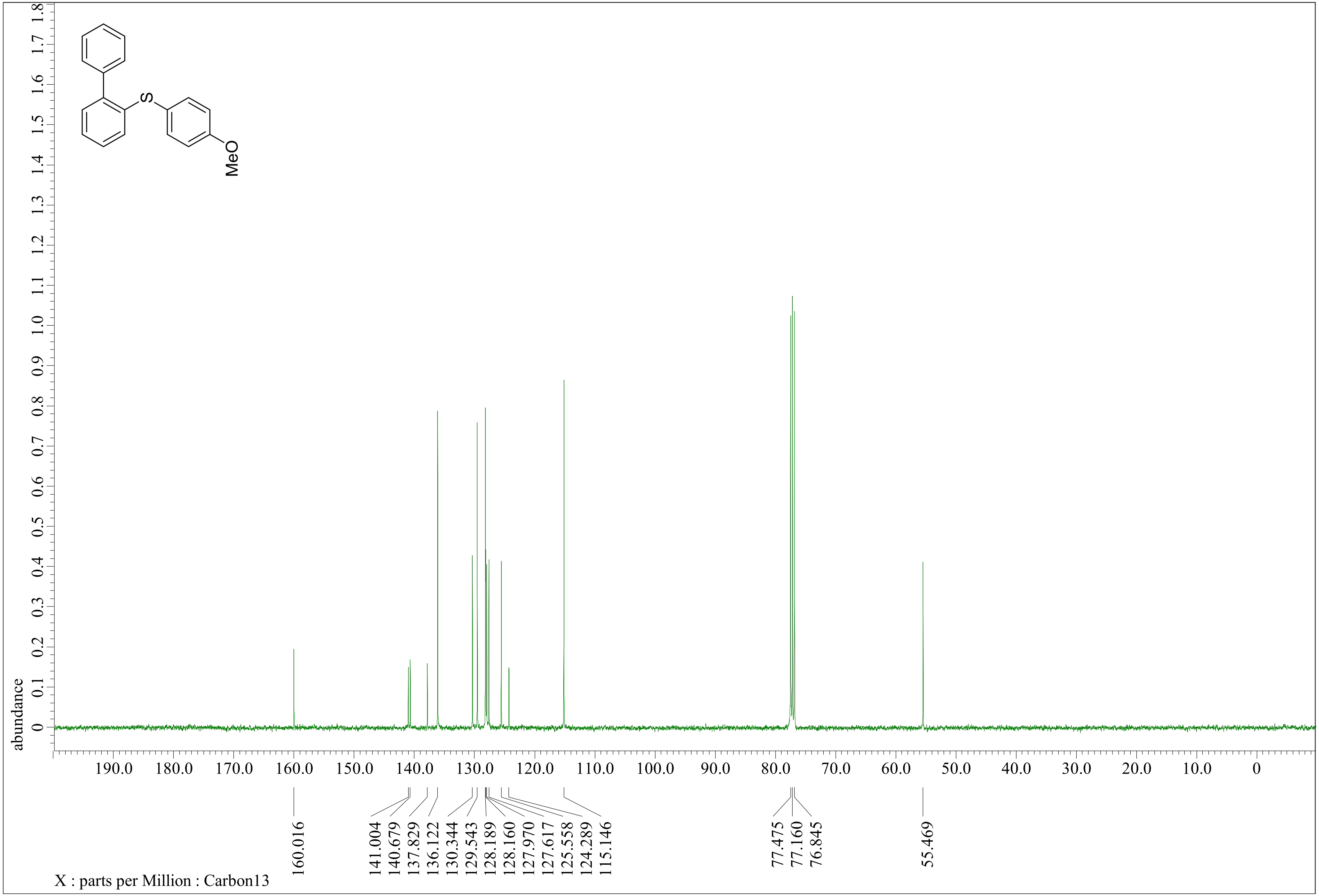
abundance
0
0.1
0.2
0.3
0.4
0.5
0.6
0.7
0.8
0.9
1.0
1.1
1.2
1.3
1.4
1.5
1.6
1.7
1.8
S
MeO
160.016
141.004
140.679
137.829
136.122
130.344
129.543
128.189
128.160
127.970
127.617
125.558
124.289
115.146
77.475
77.160
76.845
55.469
190.0
180.0
170.0
160.0
150.0
140.0
130.0
120.0
110.0
100.0
90.0
80.0
70.0
60.0
50.0
40.0
30.0
20.0
10.0
0
X : parts per Million : Carbon13

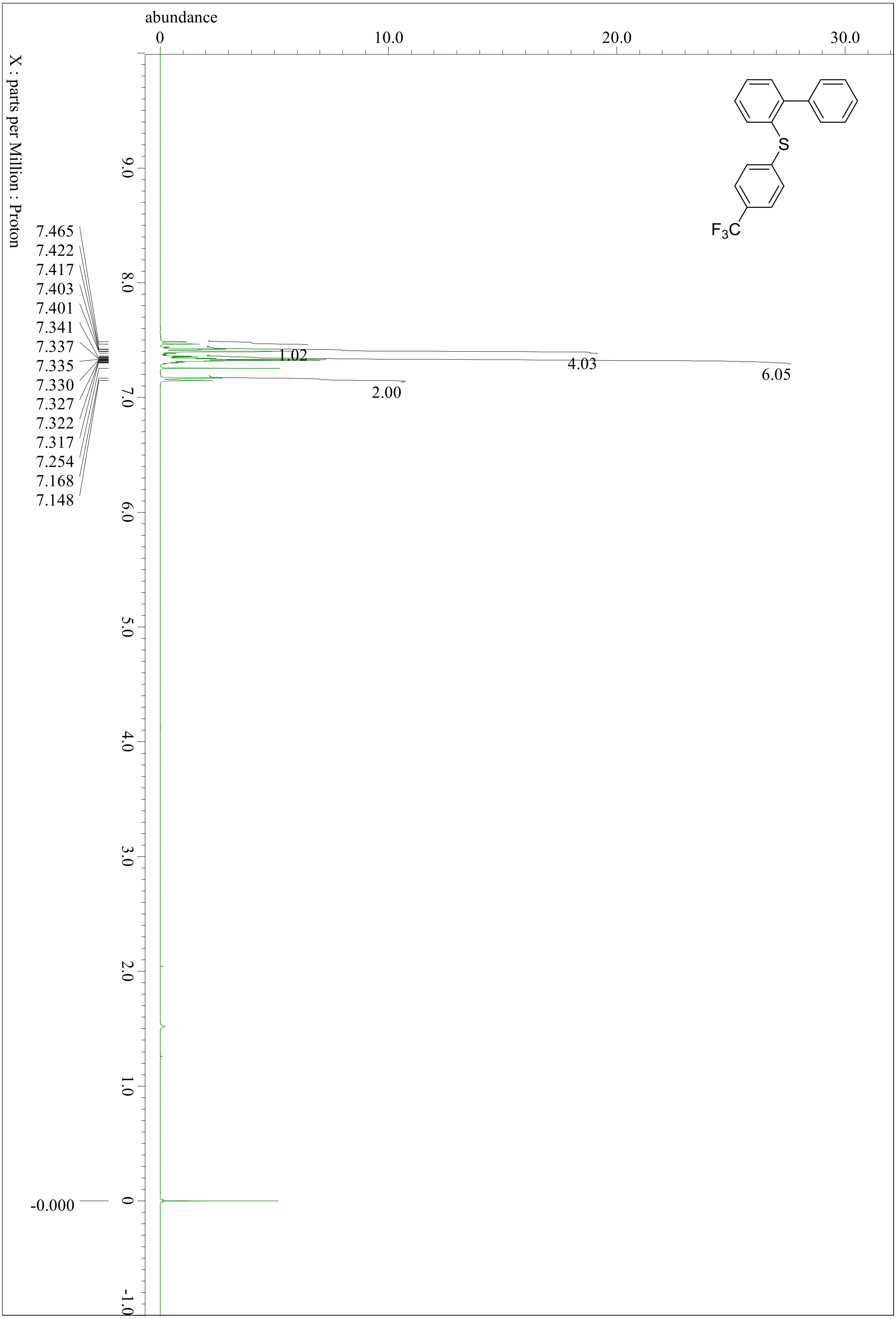
abundance
0
10.0
20.0
30.0
X : parts per Million : Proton
9.0
8.0
7.0
6.0
5.0
4.0
3.0
2.0
1.0
0
-1.0
7.465
7.422
7.417
7.403
7.401
7.341
7.337
7.335
7.330
7.327
7.322
7.317
7.254
7.168
7.148
-0.000
1.02
4.03
6.05
2.00
S
F3C

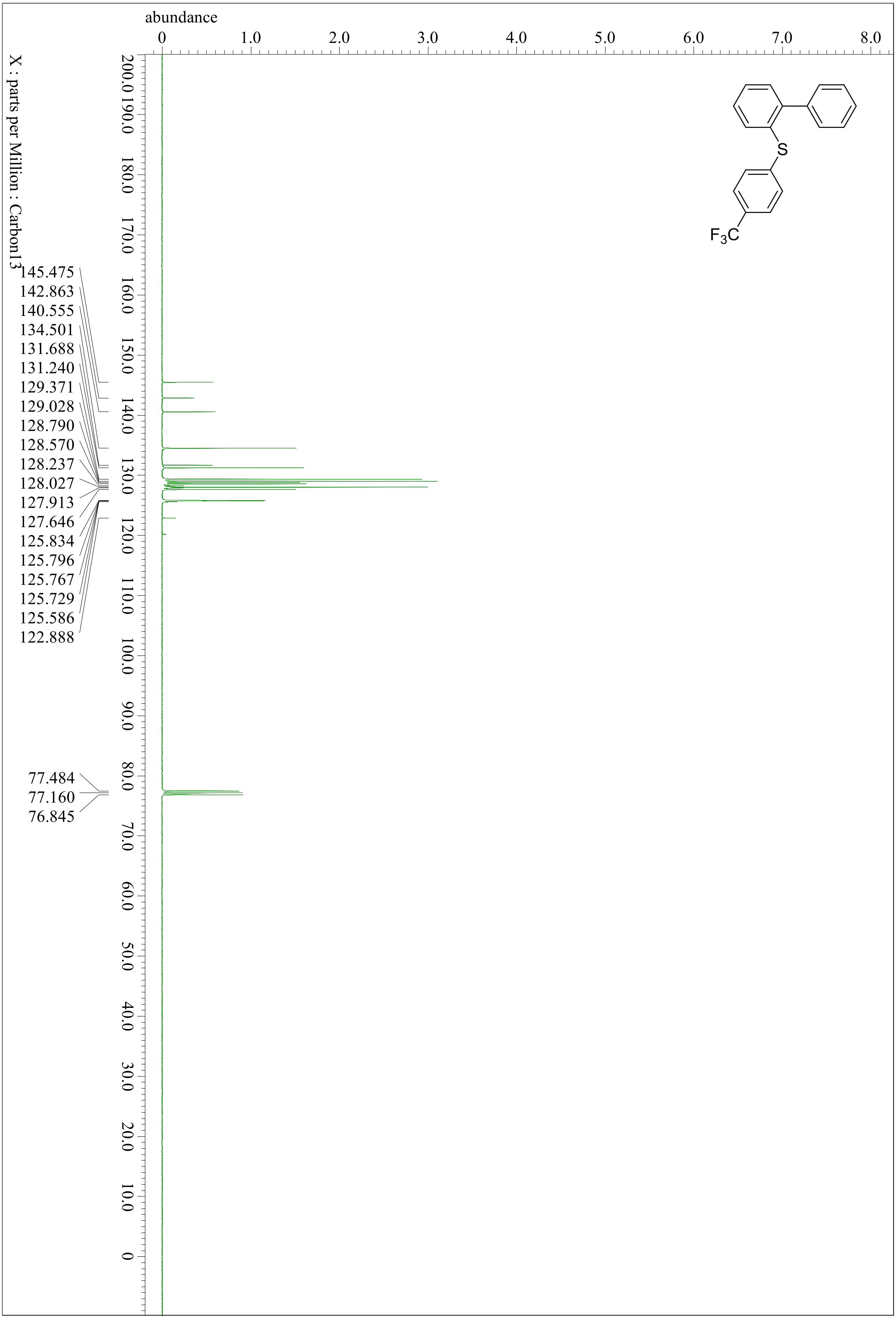
abundance
0
1.0
2.0
3.0
4.0
5.0
6.0
7.0
8.0
X : parts per Million : Carbon13
145.475
142.863
140.555
134.501
131.688
131.240
129.371
129.028
128.790
128.570
128.237
128.027
127.913
127.646
125.834
125.796
125.767
125.729
125.586
122.888
77.484
77.160
76.845
200.0
190.0
180.0
170.0
160.0
150.0
140.0
130.0
120.0
110.0
100.0
90.0
80.0
70.0
60.0
50.0
40.0
30.0
20.0
10.0
0
S
F_3C
